# Peripheral blood proteomic profiling of idiopathic pulmonary fibrosis biomarkers in the multicentre IPF-PRO Registry

**DOI:** 10.1186/s12931-019-1190-z

**Published:** 2019-10-22

**Authors:** Jamie L. Todd, Megan L. Neely, Robert Overton, Katey Durham, Mridu Gulati, Howard Huang, Jesse Roman, L. Kristin Newby, Kevin R. Flaherty, Richard Vinisko, Yi Liu, Janine Roy, Ramona Schmid, Benjamin Strobel, Christian Hesslinger, Thomas B. Leonard, Imre Noth, John A. Belperio, Scott M. Palmer, on behalf of the IPF-PRO Registry investigators, Wael Asi, Albert Baker, Scott Beegle, John A. Belperio, Rany Condos, Francis Cordova, Daniel A. Culver, Joao A. M. de Andrade, Daniel Dilling, Kevin R. Flaherty, Marilyn Glassberg, Mridu Gulati, Kalpalatha Guntupalli, Nishant Gupta, Amy Hajari Case, David Hotchkin, Tristan Huie, Robert Kaner, Hyun Kim, Maryl Kreider, Lisa Lancaster, Joseph Lasky, David Lederer, Doug Lee, Timothy Liesching, Randolph Lipchik, Jason Lobo, Yolanda Mageto, Prema Menon, Lake Morrison, Andrew Namen, Justin Oldham, Rishi Raj, Murali Ramaswamy, Tonya Russell, Paul Sachs, Zeenat Safdar, Barry Sigal, Leann Silhan, Mary Strek, Sally Suliman, Jeremy Tabak, Rajat Walia, Timothy P. Whelan

**Affiliations:** 1 Duke Clinical Research Institute, Durham, NC USA; 2 Duke University Medical Center, Durham, NC USA; 3 Boehringer Ingelheim Pharmaceuticals Inc., Ridgefield, CT USA; 4 Yale School of Medicine, New Haven, CT USA; 5 Houston Methodist Hospital, Houston, TX USA; 6 Jane and Leonard Korman Respiratory Institute, Philadelphia, PA USA; 7 Duke Clinical & Translational Science Institute, Durham, NC USA; 8 Division of Pulmonary and Critical Care Medicine, University of Michigan, Ann Arbor, MI USA; 9 Staburo GmbH, Munich, Germany; 10 Boehringer Ingelheim Pharma GmbH & Co. KG, Biberach, Germany; 11 University of Virginia, Charlottesville, Virginia, USA; 12 David Geffen School of Medicine at UCLA, California, Los Angeles USA

**Keywords:** Interstitial lung diseases, Observational study, Proteome, Registries

## Abstract

**Background:**

Idiopathic pulmonary fibrosis (IPF) is a progressive lung disease for which diagnosis and management remain challenging. Defining the circulating proteome in IPF may identify targets for biomarker development. We sought to quantify the circulating proteome in IPF, determine differential protein expression between subjects with IPF and controls, and examine relationships between protein expression and markers of disease severity.

**Methods:**

This study involved 300 patients with IPF from the IPF-PRO Registry and 100 participants without known lung disease. Plasma collected at enrolment was analysed using aptamer-based proteomics (1305 proteins). Linear regression was used to determine differential protein expression between participants with IPF and controls and associations between protein expression and disease severity measures (percent predicted values for forced vital capacity [FVC] and diffusion capacity of the lung for carbon monoxide [DLco]; composite physiologic index [CPI]). Multivariable models were fit to select proteins that best distinguished IPF from controls.

**Results:**

Five hundred fifty one proteins had significantly different levels between IPF and controls, of which 47 showed a |log_2_(fold-change)| > 0.585 (i.e. > 1.5-fold difference). Among the proteins with the greatest difference in levels in patients with IPF versus controls were the glycoproteins thrombospondin 1 and von Willebrand factor and immune-related proteins C-C motif chemokine ligand 17 and bactericidal permeability-increasing protein. Multivariable classification modelling identified nine proteins that, when considered together, distinguished IPF versus control status with high accuracy (area under receiver operating curve = 0.99). Among participants with IPF, 14 proteins were significantly associated with FVC % predicted, 23 with DLco % predicted, 14 with CPI. Four proteins (roundabout homolog-2, spondin-1, polymeric immunoglobulin receptor, intercellular adhesion molecule 5) demonstrated the expected relationship across all three disease severity measures. When considered in pathways analyses, proteins associated with the presence or severity of IPF were enriched in pathways involved in platelet and haemostatic responses, vascular or platelet derived growth factor signalling, immune activation, and extracellular matrix organisation.

**Conclusions:**

Patients with IPF have a distinct circulating proteome and can be distinguished using a nine-protein profile. Several proteins strongly associate with disease severity. The proteins identified may represent biomarker candidates and implicate pathways for further investigation.

**Trial registration:**

ClinicalTrials.gov (NCT01915511).

## Background

Idiopathic pulmonary fibrosis (IPF) is a progressive fibrotic interstitial lung disease of unknown cause [1]. Establishing a confident diagnosis of IPF remains a clinical challenge and relies on a multifaceted, multidisciplinary approach [1, 2]. Two anti-fibrotic drugs, nintedanib and pirfenidone, have been approved for the treatment of IPF and shown to slow the rate of lung function decline [3, 4]. However, the rate of disease progression in patients with IPF is variable, and there are no reliable predictors of disease progression or indicators of therapeutic response. The discovery and development of IPF-specific biomarkers for use as diagnostic adjuncts or measures of disease activity or treatment response remains a critical unmet need [5].

Most of the currently available clinical biomarkers are proteins. Proteomic profiling represents a highly translatable initiation point for biomarker discovery [6, 7]. Proteomics, the broad-scale, simultaneous quantification of a large number of proteins using high throughput technology, enables an understanding of the relationship between numerous potential protein biomarkers and disease-specific parameters. The results of such studies can be validated using targeted approaches such as enzyme-linked immunosorbent assays (ELISAs) where such assays exist. Given their relative methodological ease, protein-based assays are often more readily implemented in the clinical laboratory than other molecular assays.

Prior proteomics work has suggested that patients with IPF have a unique peripheral blood proteome [8, 9]. A study using aptamer-based methods showed that, compared with healthy controls, the blood of patients with IPF was enriched in proteins related to platelet activation and coagulation responses, complement activation, and cardiac muscle hypertrophy, while proteins related to host defence were under-represented [8]. This study identified a set of proteins that, when considered together, discriminated between patients with IPF and healthy controls. However, this work was limited by the small size of the cohort, thus the generalisability of the observations is uncertain.

In the current study, we leveraged a multicentre cohort of well-characterised patients with IPF to quantify the peripheral blood proteome, determine differential protein expression in patients with IPF versus controls of similar age, sex and smoking history distribution, and identify combinations of proteins that best distinguished patients with IPF from controls. We also examined whether circulating proteins associated with measures of IPF severity.

## Methods

### Cohorts

The IPF cohort consisted of 300 patients enrolled in the Idiopathic Pulmonary Fibrosis Prospective Outcomes (IPF-PRO) Registry (NCT01915511) [10] between June 2014 and February 2017. The IPF-PRO Registry is a multicentre observational US registry of patients with IPF that was diagnosed or confirmed at the enrolling centre in the past 6 months. IPF was determined by the site investigator according to the 2011 American Thoracic Society/European Respiratory Society/Japanese Respiratory Society/Latin American Thoracic Society diagnostic guidelines [11].

Controls were drawn from the Measurement to Understand the Reclassification of Disease of Cabarrus/Kannapolis (MURDOCK) Study, a longitudinal cohort study of adults in North Carolina [12]. Participants considered for inclusion as controls in our study were white and non-Hispanic, aged 60 to 80 years, with an enrolment blood (plasma) sample. Participants were excluded if they had self-reported respiratory disease, cancer, or autoimmune disease at enrolment or during follow-up, were active smokers, had second-hand tobacco exposure, or reported use of respiratory-targeted medication or immunomodulators. Stratified random sampling (stratification on sex and smoking status [ever/never]) was used to select 100 controls.

### Assays

Enrolment plasma samples were assayed using an aptamer-based platform encompassing 1305 proteins (SOMAscan, SOMALogic Inc., Boulder, CO). Data were reported in relative fluorescent units (RFU). No values were reported as below the limit of detection/quantification.

### Statistical analyses

Descriptive statistics were used to analyse patient characteristics and the expression of each protein in participants with IPF and controls. Linear regression was used to assess whether protein concentrations differed by IPF or control status when considered in a univariable fashion. Specifically, log_2_ transformed protein measurements were modelled as a function of group status (IPF versus control) such that the slope coefficient for group status estimated the fold-change (FC) in protein concentration between participants with IPF and controls. The group comparison was characterised by this estimate, its 95% confidence interval and corresponding *p* Value. *p* Values were corrected for multiple comparisons using the Benjamini-Hochberg procedure to control the false discovery rate (FDR) at 5%. Differences in protein concentrations between patients with IPF and controls were considered significant if the corrected p Value was < 0.05.

We then employed multivariable classification approaches to understand if a set of proteins could distinguish participants with IPF from controls. Considering all 1305 analytes, highly correlated proteins were identified using pairwise correlation analyses (Pearson correlation coefficient > 0.9) and proteins were removed such that those omitted were those correlated with the most other proteins, resulting in the fewest possible analytes removed (*n* = 143) [13]. The remaining data were Box-Cox transformed, centred and scaled. Prior to model fitting, the data on all 400 participants were randomly divided into training (75%) and test (25%) sets. Two linear and 6 nonlinear models were fit. Linear models were penalised logistic regression (GLMN) and partial least squares (PLS) [13]. Nonlinear models were flexible discriminant analysis (FDA), support vector machines (SVM), K-nearest neighbours (KNN), recursive partitioning - single tree (RPART), random forest (RF), and gradient boosted machine (GBM) [13]. While fitting each model using the training set, 10-fold cross validation was used to choose the optimal tuning parameter based on the area under the receiver operating curve. Operating characteristics including accuracy, kappa, specificity, and sensitivity, as well as positive and negative predictive values were computed in the training set. To evaluate model results, confusion matrices were calculated using a probability cut-off of 0.5 to convert model-predicted probabilities to IPF or control classifications. The model performance characteristics were then computed on the test set. Variable importance measures for each model were assessed and the most important proteins across the models were summarised. We also explored the discrimination of subjects with IPF from controls using a relatively simple linear discrimination function. This function was then refit to the entire 400-participant cohort.

In the IPF cohort, we used univariate linear regression models to determine if circulating proteins were associated with measures of disease severity. Three measures of disease severity were considered: forced vital capacity (FVC) % predicted, diffusion capacity of the lung for carbon monoxide (DLco) % predicted, and the composite physiologic index (CPI), which correlates with the amount of radiographic fibrosis [14]. Each measure was analysed as a continuous variable. As the use of antifibrotic treatment may be related to disease severity, the analyses were repeated adjusting for treatment at enrolment (nintedanib, pirfenidone, neither). Comparisons were considered significant if the FDR-corrected *p* Value was < 0.05 and there was a ≥ 5 point difference in the disease severity measure per unit change in the log_2_RFU for the protein (i.e. the protein had a statistically significant association and a doubling of the protein concentration was associated with a ≥ 5-point difference in the disease severity measure). All statistical analyses were completed in SAS version 9.4 or R version 3.4.2 (‘Short Summer’).

Pathways analyses were performed on proteins found to be significant in the analyses described above using EnrichR [15] based on the Reactome 2016 pathway database [16].

## Results

### Cohort characteristics

In the IPF cohort (*n* = 300), the median (Q1, Q3) age at enrolment was 70.0 (65.0, 75.0) years, 74% were men, 94% were white and 67% were former smokers (Table 1). The majority of participants (73%) were classified by the investigator as having definite IPF; 54% were recorded in their medical record as taking nintedanib or pirfenidone at the time of enrolment, when the blood sample was drawn. Median (Q1, Q3) FVC % predicted was 69.7 (61.0, 80.2), DLco % predicted was 40.6 (31.7, 49.4) and CPI was 53.5 (46.6, 60.5). In the control cohort (*n* = 100), the median (Q1, Q3) age at enrolment was 66.0 (63.0, 71.5) years, 74% were men, all were white, and 68% were former smokers.

**Table 1:** Characteristics of the IPF cohort (*N* = 300)

Age, years, median (Q1, Q3)	70 (65, 75)
Male, n (%)	223 (74.3%)
Race, n (%)	
White	281 (93.7%)
Black/African-American	8 (2.7%)
Asian	6 (2.0%)
Other	5 (1.7%)
Ethnicity (Hispanic or Latino), n (%)	8 (2.7%)
Smoking status, n (%)	
Past	202 (67.3%)
Never	96 (32.0%)
Current	2 (0.7%)
Diagnostic criteria^a^, n (%)	
Definite IPF	220 (73.3%)
Probable IPF	63 (21.0%)
Possible IPF	17 (5.7%)
Emphysema on CT, n (%)	31 (10.3%)
Supplemental oxygen use at rest, n (%)	59 (20.0%)^b^
Pulmonary function measures, median (Q1, Q3)	
FEV_1_ (L)	2.2 (1.8, 2.7)
FEV_1_ (% predicted)	77.4 (68.0, 89.1)
FVC (L)	2.7 (2.2, 3.2)
FVC (% predicted)	69.7 (61.0, 80.2)
FEV_1_/FVC ratio	74.1 (72.8, 89.6)
DLco (mL/min/kPa)	12.0 (8.6, 14.7)
DLco (% predicted)	40.6 (31.7, 49.4)
CPI, median (Q1, Q3)	53.5 (46.6, 60.5)
Antifibrotic drug use, n (%)	
Pirfenidone	106 (35.3%)
Nintedanib	56 (18.7%)
Neither pirfenidone or nintedanib	138 (46.0%)

*Definition of abbreviations: CT* Computed tomography, *CPI* Composite physiologic index, *DLco* Diffusing capacity of the lungs for carbon monoxide, *FEV*_*1*_ Forced expiratory volume in 1 s, *FVC* Forced vital capacity

^a^Determined by the investigator according to 2011 ATS/ERS/JRS/ALAT diagnostic guidelines [11]

^b^Information available for 295 patients

### Circulating proteome in patients with IPF versus controls

The concentrations of the 1305 measured proteins are described in Additional file 1: Table S1. Linear regression analyses identified 551 proteins with a level that was significantly different (corrected *p* Value < 0.05) between patients with IPF and controls. Forty-seven of these proteins had a |log_2_FC| > 0.585 (i.e. a 1.5-fold difference in protein concentration between groups), of which 37 occurred at higher levels in patients with IPF than controls (Table 2, Additional file 1: Fig. S1). A total of nine proteins had a |log_2_FC| > 1 (Table 2, Additional file 1: Fig. S1).

**Table 2:** Top proteins with higher or lower levels in participants with IPF versus controls. Proteins with a |log_2_FC| > 0.585 (i.e. a > 1.5-fold difference in protein concentration between groups) and a false discovery rate (FDR)-corrected *p* Value < 0.05 are shown

Gene	Aptamer ID	Protein	log_2_FC	*p* Value	FDR adjusted *p* Value
Higher levels in participants with IPF versus controls					
PGAM1	3896–5	Phosphoglycerate mutase 1	1.260	9.00E-08	9.24E-07
GPD1	11,081–1	Glycerol-3-phosphate dehydrogenase [NAD(+)], cytoplasmic	1.224	4.95E-19	3.08E-17
THBS1	3474–19	Thrombospondin-1	1.203	1.46E-16	6.55E-15
VWF	3050–7	von Willebrand factor	1.136	3.68E-32	1.20E-29
CCL17	3519–3	C-C motif chemokine 17	1.053	2.38E-16	1.03E-14
BPI	4126–22	Bactericidal permeability-increasing protein	1.031	7.26E-15	2.37E-13
OLR1	3636–37	Oxidised low-density lipoprotein receptor 1	0.929	4.84E-32	1.26E-29
CAPG	4968–50	Macrophage-capping protein	0.882	1.09E-28	1.43E-26
SPARC	3043–49	SPARC	0.869	3.13E-15	1.13E-13
FN1	4131–72	Fibronectin	0.864	1.19E-30	2.59E-28
PF4	2697–7	Platelet factor 4	0.853	7.84E-08	8.32E-07
PPBP	4544–4	Connective tissue-activating peptide III	0.850	2.74E-08	3.26E-07
PPBP	2790–54	Neutrophil-activating peptide 2	0.844	3.23E-08	3.73E-07
ICAM5	5124–69	Intercellular adhesion molecule 5	0.841	4.10E-34	1.78E-31
CXCL13	3487–32	C-X-C motif chemokine 13	0.834	1.63E-15	6.27E-14
PGD	4187–49	6-phosphogluconate dehydrogenase, decarboxylating	0.833	7.64E-07	6.39E-06
C1R	3285–23	Complement C1r subcomponent	0.825	3.39E-29	5.49E-27
HIST1H1C	2987–37	Histone H1.2	0.808	1.58E-07	1.56E-06
MMP9	2579–17	Matrix metalloproteinase-9	0.792	4.49E-16	1.83E-14
SFTPD	4414–69	Pulmonary surfactant-associated protein D	0.789	1.75E-08	2.20E-07
GDF15	4374–45	Growth/differentiation factor 15	0.782	2.32E-28	2.75E-26
S100A9	5339–49	Protein S100-A9	0.778	9.08E-18	4.74E-16
FN1	3434–34	Fibronectin fragment 3	0.770	3.78E-29	5.49E-27
PDGFB	4149–8	Platelet-derived growth factor subunit B	0.768	7.67E-08	8.25E-07
CCL18	3044–3	C-C motif chemokine 18	0.734	2.31E-29	4.31E-27
ANXA6	5335–73	Annexin A6	0.725	3.36E-09	4.82E-08
MMP1	4924–32	Matrix metalloproteinase-1	0.712	3.92E-11	8.25E-10
TIMP3	2480–58	Metalloproteinase inhibitor 3	0.690	4.04E-10	7.32E-09
VAV1	5275–28	Proto-oncogene vav	0.687	8.31E-05	4.15E-04
HNRNPA2B1	5351–52	Heterogeneous nuclear ribonucleoproteins A2/B1	0.683	2.27E-12	5.69E-11
PDGFA	4499–21	Platelet-derived growth factor subunit A	0.665	3.05E-08	3.55E-07
APP	3171–57	Amyloid-beta A4 protein	0.662	1.34E-11	2.97E-10
S100A6	13,090–17	Protein S100-A6	0.625	4.47E-14	1.33E-12
CCL5	5480–49	C-C motif chemokine 5	0.614	3.43E-07	3.15E-06
C4A C4B	2182–54	Complement C4A and C4B	0.604	1.13E-08	1.49E-07
CCL22	3508–78	C-C motif chemokine 22	0.599	6.96E-18	3.95E-16
HK2	13,130–150	Hexokinase-2	0.592	2.22E-09	3.35E-08
Lower levels in participants with IPF versus controls					
MMP3	2788–55	Stromelysin-1	−1.343	2.77E-36	1.81E-33
CKB CKM	3714–49	Creatine kinase B-type; Creatine kinase M-type	−1.325	4.95E-22	4.04E-20
ADSL	5023–23	Adenylosuccinate lyase	−1.126	2.70E-26	2.93E-24
SHH	2743–5	Sonic hedgehog protein	−0.852	7.35E-26	7.38E-24
CA6	3352–80	Carbonic anhydrase 6	−0.778	2.15E-15	8.01E-14
AGER	4125–52	Advanced glycosylation end product-specific receptor	−0.770	5.74E-20	3.74E-18
HSPB1	11,103–24	Heat shock protein beta-1	−0.734	1.46E-05	9.18E-05
TFF1	9185–15	Trefoil factor 1	−0.708	1.20E-12	3.20E-11
PRKCA	2644–11	Protein kinase C alpha type	−0.695	3.68E-04	1.54E-03
PRKACA	3466–8	cAMP-dependent protein kinase catalytic subunit alpha	−0.590	7.02E-05	3.63E-04

*p* Values are shown as exponentiated values

Among the top proteins with higher circulating levels in the IPF cohort than in controls were several immune-related proteins including chemokine (CC motif) ligand (CCL) 5, 17, 18, 22; chemokine (C-X-C motif) ligand 13 (CXCL13); and complement components C1R, C4A and C4B; as well as extracellular matrix components (including fibronectins), matrix remodelling proteins (including matrix metalloproteinases [MMPs] 1 and 9 and tissue inhibitor of metalloproteinase [TIMP] 3), and proteins important in cell proliferation, adhesion, or motility (such as platelet-derived growth factor [PDGF] subunits A and B, intracellular adhesion molecule 5 [ICAM5)] and secreted protein, acidic and rich in cysteine [SPARC]). Among the top proteins that were observed at lower levels in patients with IPF relative to controls were the matrix remodelling protein stromelysin-1 (MMP3), creatine kinase enzymes B and M, and the advanced glycosylation end products receptor (AGER).

### Multiprotein classification approaches to distinguish patients with IPF from controls

**Fig. 1:**
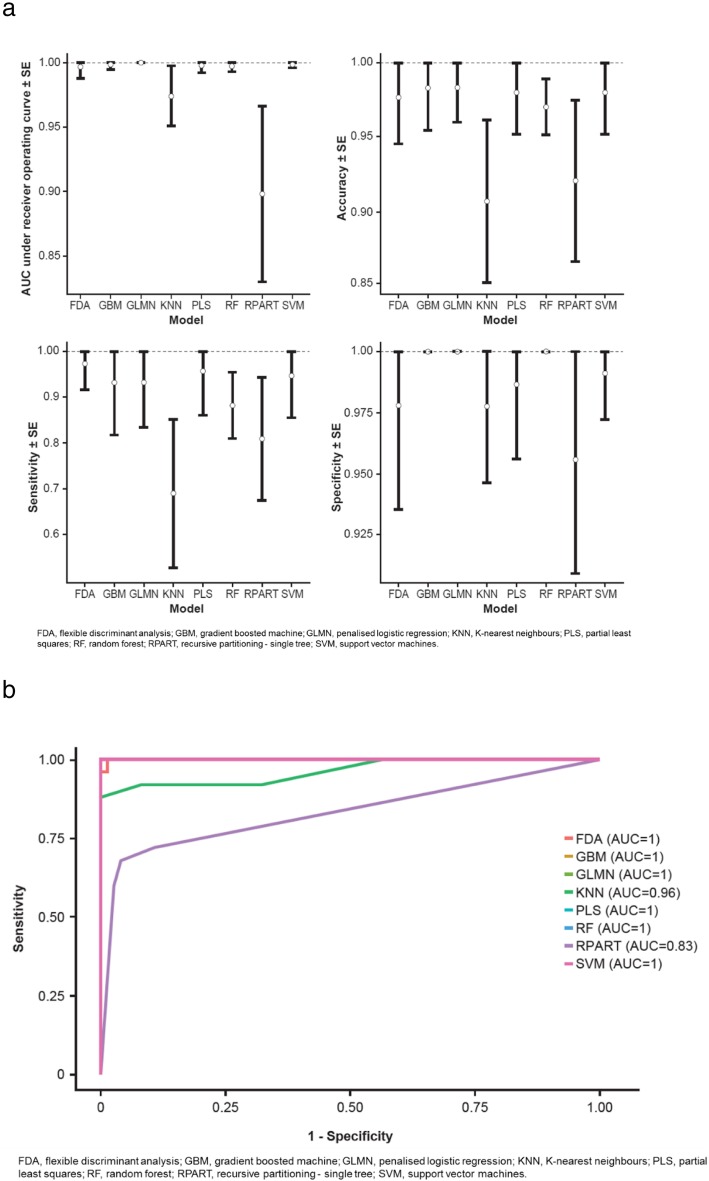
Operating characteristics of linear and non-linear models to differentiate patients with IPF from controls in training set (**a**) and receiver operating curve for the test set (**b**)

We sought to identify a set of proteins that optimally differentiated patients with IPF from controls by fitting models on a training set and a test set. Select performance measures by model in the training set are illustrated in Fig. 1. Six of the eight multivariable classification models evaluated (both linear models [GLMN, PLS] and four non-linear models [FDA, SVM, RF, GBM]) had a good overall ability to distinguish between participants with IPF from controls. Several models made no or minimal classification errors for all iterations of the cross-validation procedure, as indicated by models with an area under the curve (AUC) of 1 with no or minimal variation (Fig. 1A). When the models were applied to the test set, we observed similar results (Fig. 1B). Computed operating characteristics for all models in the test set are shown in Additional file 1: Table S2.

**Fig. 2:**
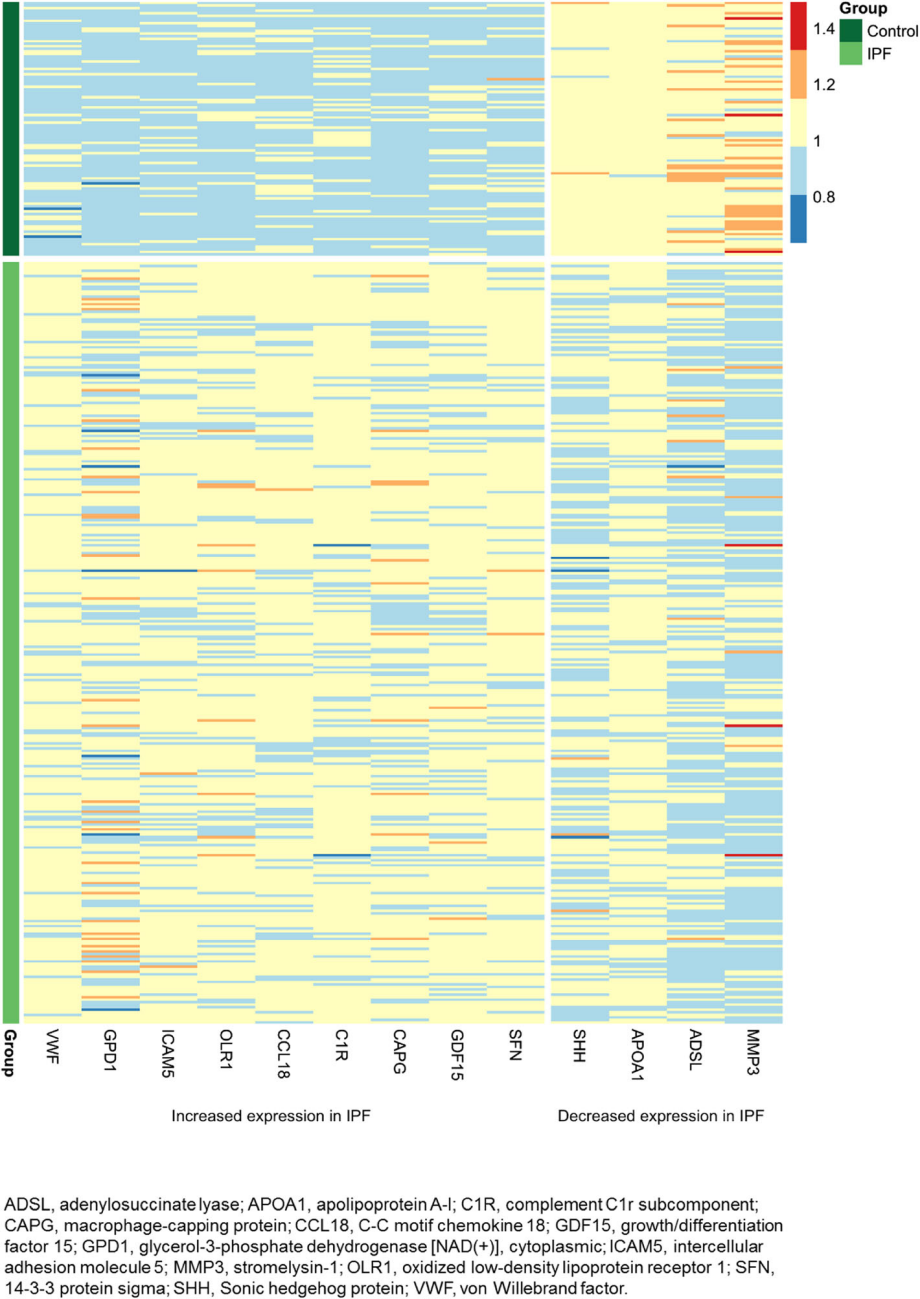
Heat map indicating expression of most frequently observed proteins of importance across the linear and non-linear models in patients with IPF versus controls

To understand the proteins of importance in distinguishing patients with IPF from controls, we determined the variable importance measures of proteins selected by each multivariable model. Thirteen proteins were designated as among the 10 most influential proteins in at least two of the eight models (Additional file 1: Table S3). A heat map of the expression of these proteins in participants with IPF versus controls is shown in Fig. 2.

As the performance of the linear models was equivalent to that of the more complex non-linear models, we explored the discrimination of IPF using a linear discriminant function with recursive feature elimination. This indicated that the optimal number of proteins to differentiate participants with IPF from controls was nine (Table 3). The linear discriminant analysis considering these nine proteins had an AUC of 0.99. Linear discriminant scores for every participant were calculated by multiplying the protein values for each selected protein by the respective model coefficient (Table 3) and plotted by IPF versus control status. As illustrated in Additional file 1: Fig. S2, the linear discriminant analysis based on these nine proteins distinguished patients with IPF from control subjects with very little overlap.

**Table 3:** Nine proteins that optimally differentiated patients with IPF from control participants. Proteins selected by the linear discriminant function with recursive feature elimination and the respective model coefficient

Gene	Aptamer ID	Protein	Model coefficient^a^
APOA1	2750–3	Apolipoprotein A-I	0.38
C1R	3285–23	Complement C1r subcomponent	−0.41
MMP3	2788–55	Stromelysin-1	−0.30
SFN	4829–43	14–3-3 protein sigma	0.43
CCL18	3044–3	C-C motif chemokine 18	0.36
ICAM5	5124–69	Intercellular adhesion molecule 5	−0.06
SHH	2743–5	Sonic hedgehog protein	0.46
OLR1	3636–37	Oxidised low-density ipoprotein receptor 1	−0.32
CAPG	4968–50	Macrophage-capping protein	0.28

^a^The sign of the coefficient indicates whether the subject’s linear discriminant analysis score increases or decreases as values of the protein change. Higher scores are associated with IPF, as opposed to control, status

### Association between circulating proteome and measures of disease severity in patients with IPF

**Fig. 3:**
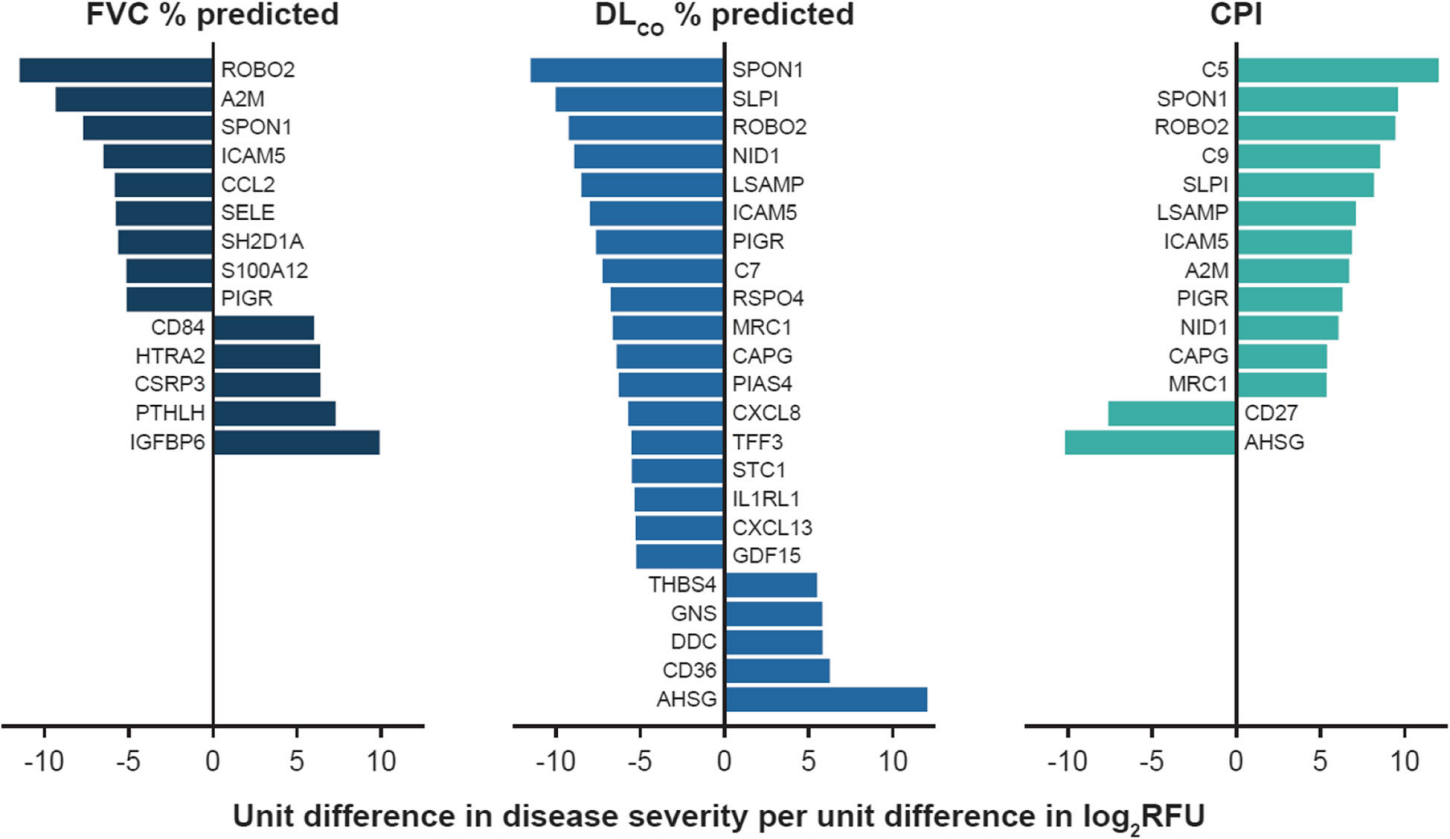
Proteins significantly associated with measures of disease severity in patients with IPF. All proteins presented had an FDR-corrected *p* Value < 0.05 and a > 5-unit difference in the respective disease severity measure per unit change in log_2_RFU (i.e., doubling of protein concentration)

Using significance criteria of a corrected *p* Value < 0.05 and a ≥ 5-unit difference in disease severity measure per doubling in protein concentration, we identified 14 proteins that were associated with FVC % predicted, 23 with DLco % predicted, and 14 with CPI (Fig. 3). These associations were largely unchanged after adjustment for treatment (nintedanib, pirfenidone, neither) at enrolment (Additional file 1: Tables S4-S6). Four proteins, roundabout homolog-2 (ROBO2), spondin-1 (SPON1), polymeric immunoglobulin receptor (PIGR) and ICAM 5, satisfied both analytic criteria for all three disease severity measures. Each of these proteins were observed at higher levels in patients with more severe disease.

### Pathways analysis of proteins associated with presence or severity of IPF

**Fig. 4:**
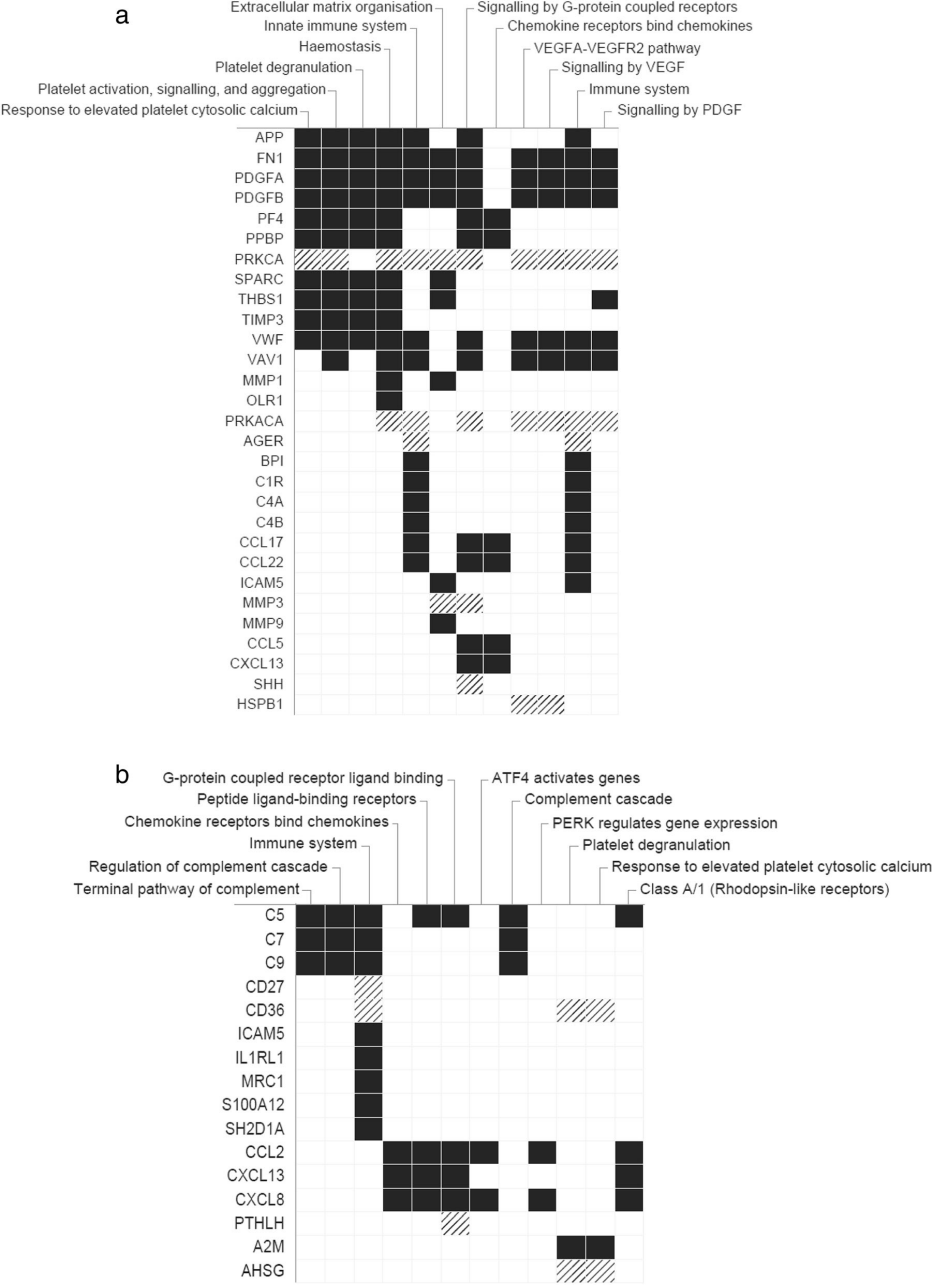
Top 12 pathways/gene sets related to proteins observed at higher (black) or lower (hatched) levels in patients with IPF versus controls (Benjamini-Hochberg corrected *p* Value for enrichment in respective pathway using Fisher’s exact test < 4.40E-5) (**a**) or observed at higher levels in more severe disease (black) or less severe disease (hatched) in patients with IPF (corrected *p *Value for enrichment *<* 0.029) (**b**) as identified by EnrichR, sorted according to the combined score^15^

To elucidate potential pathways related to the presence or severity of IPF, we performed a pathways analysis on proteins demonstrated to be significant in the previous analyses. In analyses of the 47 proteins that occurred at different levels in patients with IPF versus controls with an absolute > 1.5-fold change and a corrected *p* Value < 0.05, we observed a significant enrichment of proteins in pathways related to platelet activation, innate immunity, extracellular matrix organisation, and vascular growth factor signalling (Fig. 4A). The same pathways, plus mechanistically-related pathways and processes, were identified in analyses of the 36 proteins that were significantly correlated with measures of disease severity (Fig. 4B). Additionally, activation and regulation of the complement cascade appeared to be prominent pathways of importance in disease severity.

## Discussion

In this comprehensive study using a targeted platform of over 1300 proteins, we identified a distinct circulating proteome associated with IPF. When considered together, nine proteins accurately distinguished patients with IPF from controls who had a similar distribution of age, sex, and smoking status. Further, several proteins were associated with clinical measures of disease severity. When proteins associated with the presence or severity of IPF were considered in pathways analyses, they tended to be found in pathways involved in platelet and haemostatic responses, including vascular growth factor signalling, immune activation (including innate immunity and the complement cascade), and extracellular matrix organisation.

The majority of proteomic studies in IPF have focussed on the characterisation of protein expression in lung tissue or bronchoalveolar lavage fluid (BALF) [17–21], with only a few studies having quantified the circulating proteome [8, 9]. An additional novel aspect of our analysis was the identification of proteins associated with clinical measures of disease severity, as well as proteins associated with the presence of IPF. In general, the proteins associated with disease severity were distinct from those that distinguished patients with IPF from controls. Though it was expected that proteins associated with CPI would also be associated with DLco or FVC, given that these measures are used in the CPI calculation, we observed that only four proteins (ROBO2, SPON1, PIGR, ICAM5) were associated with all three disease severity measures.

Our observation related to expression of circulating PIGR, a transmembrane glycoprotein important in immunoglobulin A transport across mucosal epithelial cells, is particularly intriguing, as prior work has demonstrated that the lungs of patients with IPF have ectopic expression of PIGR within areas of type 2 alveolar cell hyperplasia [22]. Moreover, PIGR-deficient mice demonstrated attenuated lung fibrosis after bleomycin treatment compared with wild-type mice [22]. Others have demonstrated that PIGR is upregulated by cytokines induced by innate immune activation and have implicated PIGR as a bridge between innate and adaptive immune responses [23], responses which we found to be enriched in pathways analyses of proteins associated with disease severity. While the other three proteins associated with all three disease severity measures have not been well characterised in lung fibrosis, ROBO2 has been demonstrated to be overexpressed in a murine model of toxin-induced liver fibrosis, where it localised on the surface of hepatic stellate cells within fibrotic septae. Moreover, the interaction between ROBO2 and its ligand (slit guidance ligand 2) promoted fibrogenic activity within stellate cells [24].

In prior work, an aptamer-based proteomic approach similar to that used in our analysis was used to quantify 1129 circulating proteins in 60 patients with IPF versus 21 healthy controls of older mean age who were lifetime non-smokers. Consistent with our observations, higher levels of complement C1r subcomponent, complement C4, fibronectin, ICAM 5, thrombospondin 1, and MMP1 were observed in the IPF cohort [8]. However, many of the proteins found to have lower levels in patients with IPF than in controls in this previous study were observed at higher levels in patients with IPF than controls in our study, including MMP9, S100A9, and surfactant protein D, for which other literature supports increased expression in IPF [8, 25–29]. The factors accounting for these divergent observations are likely multifactorial, and may include the types of assays used, technical aspects of the aptamer-based assay, differences in disease severity between the groups with IPF, or differences between the control groups.

While the peripheral blood proteome may not fully reflect intrapulmonary changes, several of our findings are consistent with those of proteomic studies of BALF or lung tissue. A study using mass spectrometry-based proteomics of BALF demonstrated a 3-fold increase in CCL18 and protein S100A9 in patients with IPF compared with controls [18]. Another proteomic study of BALF from patients with fibrotic diseases, including IPF, demonstrated increased expression of S100A6 [20]. Several proteins observed at higher or lower levels in patients with IPF in our study were consistent with observations from a study that performed unbiased proteomics on lung tissue samples from patients with fibrosing lung disease. For example, both studies demonstrated higher levels of CCL13 and lower levels of AGER compared with controls [17]. These observations suggest that blood-based protein analysis may be a useful tool to phenotype patients with IPF and facilitate monitoring of disease progression. Consistent with this idea, Maher et al. quantified 123 circulating proteins in patients with IPF and identified a new IPF-associated protein, cancer antigen-125 protein, rising levels of which were associated with the risk of disease progression and mortality [29]. The newly identified IPF-associated circulating proteins identified in our analyses expand the pool of candidate biomarkers for further evaluation in relation to clinically relevant outcomes.

Our results support the importance of circulating proteins relevant to extracellular matrix remodelling in patients with IPF. Notably several extracellular matrix glycoproteins, MMPs 1 and 9, and the MMP inhibitor TIMP3 were present at higher levels in patients with IPF relative to controls. These data are of interest in view of prior work by Jenkins et al. demonstrating that circulating levels of protein fragments generated by MMP activity are increased in patients with IPF relative to healthy controls and may associate with disease progression [30]. Although the majority of our data with regard to extracellular matrix remodelling protein expression are consistent with prior work, we note a particular discordance between our results and those of previous studies related to MMP3. High MMP3 levels have been reported in lung tissue from patients with IPF, and genetic deletion of MMP3 in mice abrogates bleomycin-induced pulmonary fibrosis [31, 32]. In contrast to these observations, in our cohort, of all the proteins with lower levels in patients with IPF than in controls, MMP3 showed the strongest association. Given that MMP3 was selected as a protein of importance in multivariable models distinguishing patients with IPF from controls, including the linear discriminant analysis, we examined the sensitivity of this model to the exclusion of MMP3. When the analysis was performed without MMP3 in the pool of analytes available for model selection, the optimal number of proteins to differentiate participants with IPF from controls was also nine, with adenylosuccinate lyase filling the final position and the remaining markers chosen in the same order. The linear discriminant analysis considering these nine proteins also had an AUC of 0.99 (data not shown).

Our study has several strengths, including the multicentre nature of the IPF cohort and the inclusion of control participants of comparable age, sex and smoking distribution. However, we acknowledge some inherent limitations. First, we acknowledge that our cohort is a US-based population of predominantly white patients, thus broader generalisability to other populations of patients with IPF is uncertain. Additionally, although we characterised a broad array of proteins, our approach was targeted rather than discovery-based, so proteins of potential importance could have been missed if not included on our platform. Finally, we acknowledge that an aptamer-based approach to protein detection and quantification does not always yield results that are reproducible when using ELISA-based approaches. This may in fact explain the differences between previous studies and our results with regard to MMP3. Thus, the proteins we identified as of interest in our study need to be validated, both from a technical and a clinical viewpoint. In particular, the association of the circulating proteins identified herein with clinical measures of IPF severity warrants validation.

## Conclusion

The results of this study add to the evidence suggesting that circulating proteins are likely to hold value in the diagnostic approach to IPF. Additionally, these data indicate that profiling of circulating proteins may provide insights into biological pathways underlying the development of IPF or contributing to disease severity. Validation of candidate proteins will be necessary, as will extension of these analyses to examine the association of the circulating proteome with clinical outcomes. Rich longitudinal data collection through the IPF-PRO Registry, including serial pulmonary function measures, hospitalisation data, and information on vital status, will support these analyses and further the goal of improving the diagnosis and management of IPF.

## Supplementary information

**Additional file 1: Figure S1.** Differential levels of circulating proteins in participants with IPF versus controls. Volcano plot of the Log2fold change in means by log10 of the corrected p Value for each protein. The horizontal line indicates the threshold for statistical significance. **Figure S2.** Histogram of the linear discriminant scores for each participant in the IPF and control cohort. **Table S1.** Summary statistics for all 1305 proteins assayed across the IPF and control cohorts. Protein data are reported in relative fluorescent units. **Table S2.** Operating characteristics of all models in the test set for the IPF versus control multivariable modelling. **Table S3.** Proteins designated as among the most influential in at least two of the eight multivariable models. **Table S4.** Proteins significantly associated with FVC % predicted (unadjusted and adjusted for anti-fibrotic treatment). **Table S5.** Proteins significantly associated with DLco % predicted (unadjusted and adjusted for anti-fibrotic treatment). **Table S6.** Proteins significantly associated with composite physiologic index (unadjusted and adjusted for anti-fibrotic treatment).

## Data Availability

All data relevant to the study are included in the article or uploaded as supplementary information.

## Abbreviations

AGER: Advanced glycosylation end products receptor
AUC: Area under the curve
BALF: Bronchoalveolar lavage fluid
CCL: Chemokine ligand
CPI: Composite physiologic index
DLco: Diffusion capacity of the lung for carbon monoxide
ELISA: Enzyme-linked immunosorbent assay
FC: Fold-change
FDA: Flexible discriminant analysis
FDR: False discovery rate
FVC: Forced vital capacity
GBM: Gradient boosted machine
GLMN: Penalised logistic regression
ICAM5: Intracellular adhesion molecule 5
IPF: Idiopathic pulmonary fibrosis
IPF-PRO Registry: Idiopathic Pulmonary Fibrosis Prospective Outcomes Registry
KNN: K-nearest neighbour
MMP: Matrix metalloproteinase
MMP3: Matrix remodelling protein stromelysin-1
MURDOCK: Measurement to Understand the Reclassification of Disease of Cabarrus/Kannapolis Study
PDGF: Platelet-derived growth factor
PIGR: Polymeric immunoglobulin receptor
PLS: Partial least squares
RF: Random forest
RFU: Relative fluorescent unit
ROBO2: Roundabout homolog-2
RPART: Recursive partitioning - single tree
SPARC: Secreted protein acidic and rich in cysteine
SPON1: Spondin-1
SVM: Support vector machine
TIMP: Tissue inhibitor of metalloproteinase
