# Supplementary material for: Peripheral blood proteomic profiling of idiopathic pulmonary fibrosis biomarkers in the multicentre IPF-PRO Registry
Source: Respir Res. 2019 Oct 22;20:227. doi: 10.1186/s12931-019-1190-z (PMC6805665; doi:10.1186/s12931-019-1190-z)
Supplement: Supplementary file 1 — Additional file 1: Figure S1. Differential levels of circulating proteins in participants with IPF versus controls. Volcano plot of the Log2fold change in means by log10 of the corrected p Value for each protein. The horizontal line indicates the threshold for statistical significance. Figure S2. Histogram of the linear discriminant scores for each participant in the IPF and control cohort. Table S1. Summary statistics for all 1305 proteins assayed across the IPF and control cohorts. Protein data are reported in relative fluorescent units. Table S2. Operating characteristics of all models in the test set for the IPF versus control multivariable modelling. Table S3. Proteins designated as among the most influential in at least two of the eight multivariable models. Table S4. Proteins significantly associated with FVC % predicted (unadjusted and adjusted for anti-fibrotic treatment). Table S5. Proteins significantly associated with DLco % predicted (unadjusted and adjusted for anti-fibrotic treatment). Table S6. Proteins significantly associated with composite physiologic index (unadjusted and adjusted for anti-fibrotic treatment). [file 12931_2019_1190_MOESM1_ESM.docx]

**Additional File**

**Figure S1.** Differential levels of circulating proteins in participants with IPF versus controls. Volcano plot of the Log_2_fold change in means by log10 of the corrected p Value for each protein. The horizontal line indicates the threshold for statistical significance.


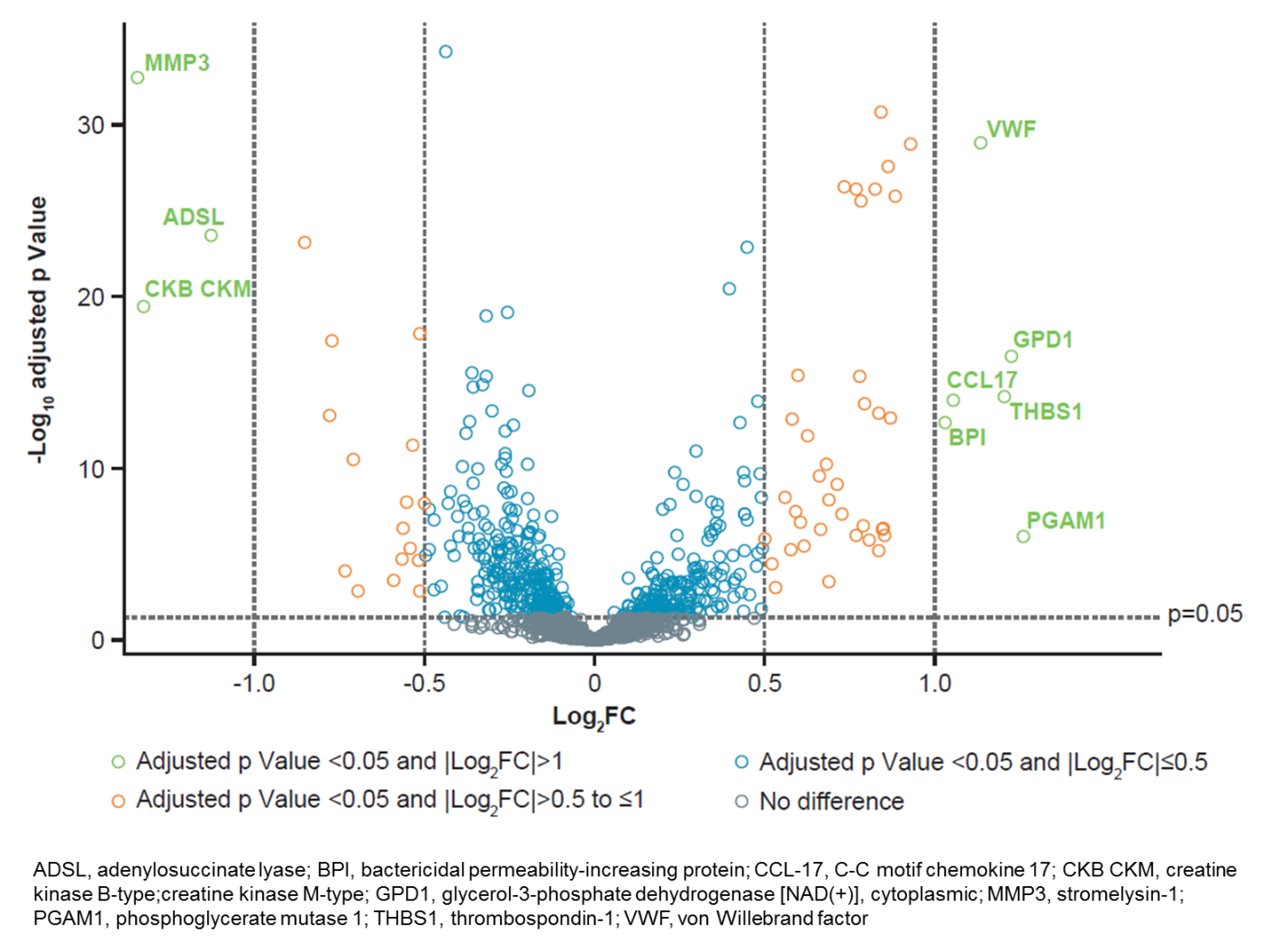


**Figure S2.** Histogram of the linear discriminant scores for each participant in the IPF and control cohort.


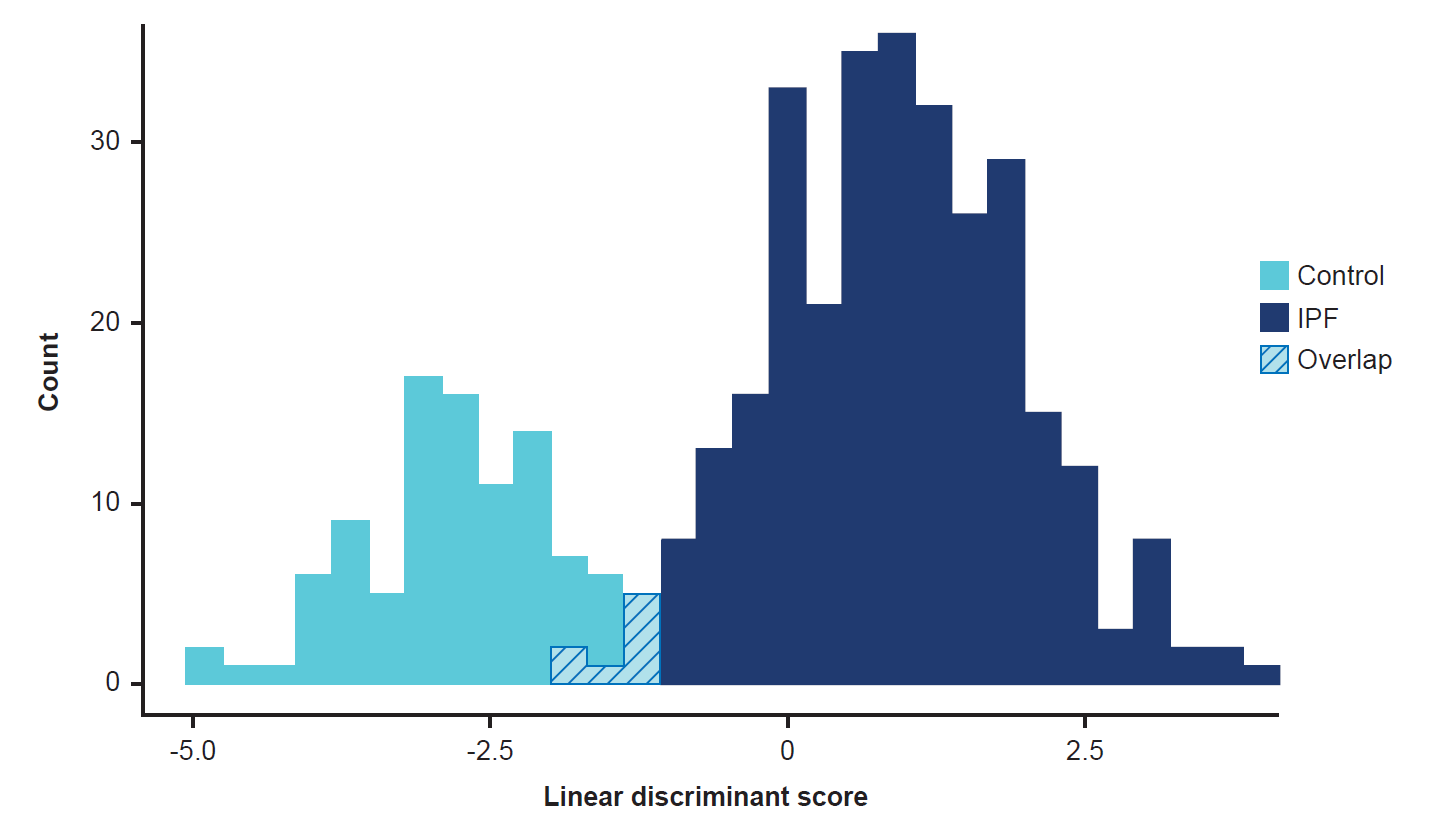


**Table S1.** Summary statistics for all 1305 proteins assayed across the IPF and control cohorts. Protein data are reported in relative fluorescent units.

| **APTAMER** | **GENE** | **UNIPROT** | **IPF population flag** | **Mean** | **Std dev** | **Minimum** | **Maximum** | **log_2_FC** | **Uncorrected p Value** | **FDR corrected p Value** |
| --- | --- | --- | --- | --- | --- | --- | --- | --- | --- | --- |
| 2788-55 | MMP3 | P08254 | 0 | 2301.03 | 1823.92 | 491.60 | 11012.90 | -1.343 | 2.77E-36 | 1.81E-33 |
|  |  |  | 1 | 974.95 | 2168.63 | 276.80 | 26125.80 |  |  |  |
| 3050-7 | VWF | P04275 | 0 | 16672.27 | 14068.43 | 1897.00 | 71743.00 | 1.136 | 3.68E-32 | 1.20E-29 |
|  |  |  | 1 | 30640.32 | 12953.38 | 5030.00 | 77830.60 |  |  |  |
| 5023-23 | ADSL | P30566 | 0 | 4469.45 | 2931.43 | 797.20 | 15243.60 | -1.126 | 2.70E-26 | 2.93E-24 |
|  |  |  | 1 | 2139.27 | 2147.93 | 471.80 | 21150.00 |  |  |  |
| 3714-49 | CKB CKM | P12277 P06732 | 0 | 1157.84 | 1864.73 | 172.60 | 16469.30 | -1.325 | 4.95E-22 | 4.04E-20 |
|  |  |  | 1 | 437.32 | 520.85 | 51.60 | 4653.40 |  |  |  |
| 11081-1 | GPD1 | P21695 | 0 | 2126.56 | 1122.81 | 555.00 | 7667.50 | 1.224 | 4.95E-19 | 3.08E-17 |
|  |  |  | 1 | 6164.64 | 4725.87 | 336.50 | 25464.30 |  |  |  |
| 3474-19 | THBS1 | P07996 | 0 | 3301.64 | 3024.62 | 359.60 | 11368.20 | 1.203 | 1.46E-16 | 6.55E-15 |
|  |  |  | 1 | 7151.51 | 6365.84 | 489.00 | 69156.10 |  |  |  |
| 3519-3 | CCL17 | Q92583 | 0 | 1914.73 | 1177.88 | 568.50 | 6277.90 | 1.053 | 2.38E-16 | 1.03E-14 |
|  |  |  | 1 | 4768.82 | 4738.29 | 554.60 | 32583.80 |  |  |  |
| 4126-22 | BPI | P17213 | 0 | 1035.01 | 990.27 | 404.20 | 8900.40 | 1.031 | 7.26E-15 | 2.37E-13 |
|  |  |  | 1 | 3229.92 | 8353.06 | 423.50 | 87079.30 |  |  |  |
| 3896-5 | PGAM1 | P18669 | 0 | 1925.14 | 2388.30 | 124.50 | 13658.50 | 1.260 | 9.00E-08 | 9.24E-07 |
|  |  |  | 1 | 6149.71 | 9522.18 | 103.40 | 80922.50 |  |  |  |
| 2750-3 | APOA1 | P02647 | 0 | 20538.22 | 3567.91 | 11922.50 | 28177.30 | -0.438 | 4.06E-38 | 5.30E-35 |
|  |  |  | 1 | 15183.25 | 2784.52 | 7962.90 | 29079.10 |  |  |  |
| 5124-69 | ICAM5 | Q9UMF0 | 0 | 1058.34 | 316.36 | 554.30 | 2114.50 | 0.841 | 4.10E-34 | 1.78E-31 |
|  |  |  | 1 | 1963.81 | 814.22 | 323.30 | 6908.40 |  |  |  |
| 3636-37 | OLR1 | P78380 | 0 | 1954.60 | 743.83 | 902.50 | 4844.60 | 0.929 | 4.84E-32 | 1.26E-29 |
|  |  |  | 1 | 3950.21 | 2657.80 | 916.10 | 33031.30 |  |  |  |
| 4131-72 | FN1 | P02751 | 0 | 17688.97 | 11840.25 | 4154.80 | 65905.70 | 0.864 | 1.19E-30 | 2.59E-28 |
|  |  |  | 1 | 28207.90 | 8643.17 | 6418.20 | 55646.10 |  |  |  |
| 3044-3 | CCL18 | P55774 | 0 | 8027.16 | 2804.41 | 2713.80 | 17278.90 | 0.734 | 2.31E-29 | 4.31E-27 |
|  |  |  | 1 | 13499.75 | 5934.37 | 3665.80 | 63782.40 |  |  |  |
| 3285-23 | C1R | P00736 | 0 | 1691.02 | 639.60 | 606.80 | 3181.50 | 0.825 | 3.39E-29 | 5.49E-27 |
|  |  |  | 1 | 2965.27 | 921.29 | 139.40 | 5844.90 |  |  |  |
| 3434-34 | FN1 | P02751 | 0 | 3326.46 | 2103.36 | 1164.80 | 11523.00 | 0.770 | 3.78E-29 | 5.49E-27 |
|  |  |  | 1 | 5120.73 | 1563.10 | 1435.80 | 9659.50 |  |  |  |
| 4968-50 | CAPG | P40121 | 0 | 974.88 | 422.84 | 455.40 | 3961.70 | 0.882 | 1.09E-28 | 1.43E-26 |
|  |  |  | 1 | 1912.32 | 1159.15 | 630.50 | 9190.20 |  |  |  |
| 4374-45 | GDF15 | Q99988 | 0 | 1787.91 | 843.59 | 714.60 | 5697.90 | 0.782 | 2.32E-28 | 2.75E-26 |
|  |  |  | 1 | 3033.57 | 1372.15 | 921.60 | 12165.10 |  |  |  |
| 2743-5 | SHH | Q15465 | 0 | 2126.81 | 696.33 | 911.40 | 4856.60 | -0.852 | 7.35E-26 | 7.38E-24 |
|  |  |  | 1 | 1260.45 | 664.40 | 198.70 | 5972.70 |  |  |  |
| 4829-43 | SFN | P31947 | 0 | 787.23 | 315.06 | 542.80 | 3731.70 | 0.447 | 1.35E-25 | 1.26E-23 |
|  |  |  | 1 | 1076.99 | 350.52 | 645.50 | 4083.20 |  |  |  |
| 3633-70 | NID2 | Q14112 | 0 | 2498.24 | 554.45 | 1587.90 | 4605.50 | 0.398 | 3.85E-23 | 3.35E-21 |
|  |  |  | 1 | 3308.24 | 811.83 | 1785.80 | 7299.70 |  |  |  |
| 3581-53 | AHSG | P02765 | 0 | 2146.22 | 314.45 | 1513.00 | 2904.60 | -0.256 | 1.13E-21 | 8.70E-20 |
|  |  |  | 1 | 1799.30 | 269.95 | 1035.80 | 2821.70 |  |  |  |
| 2677-1 | EGFR | P00533 | 0 | 30327.25 | 5075.64 | 21622.00 | 42607.70 | -0.318 | 1.72E-21 | 1.25E-19 |
|  |  |  | 1 | 24487.21 | 5679.68 | 13001.20 | 86687.70 |  |  |  |
| 4880-21 | GDF2 | Q9UK05 | 0 | 3225.93 | 1317.23 | 1433.80 | 8769.40 | -0.514 | 2.19E-20 | 1.51E-18 |
|  |  |  | 1 | 2193.81 | 634.58 | 576.60 | 4291.50 |  |  |  |
| 4125-52 | AGER | Q15109 | 0 | 632.54 | 335.67 | 173.20 | 1768.30 | -0.770 | 5.74E-20 | 3.74E-18 |
|  |  |  | 1 | 365.49 | 200.47 | 125.40 | 1622.80 |  |  |  |
| 4866-59 | NTRK2 | Q16620 | 0 | 1259.73 | 224.82 | 883.40 | 1899.30 | -0.359 | 4.36E-18 | 2.59E-16 |
|  |  |  | 1 | 1006.01 | 441.97 | 272.70 | 7713.20 |  |  |  |
| 3508-78 | CCL22 | O00626 | 0 | 1501.19 | 520.15 | 362.80 | 3398.40 | 0.599 | 6.96E-18 | 3.95E-16 |
|  |  |  | 1 | 2290.59 | 810.41 | 330.20 | 5437.30 |  |  |  |
| 5339-49 | S100A9 | P06702 | 0 | 4483.64 | 2350.77 | 1718.80 | 16359.80 | 0.778 | 9.08E-18 | 4.74E-16 |
|  |  |  | 1 | 8261.81 | 5579.03 | 875.90 | 38076.90 |  |  |  |
| 4152-58 | KLKB1 | P03952 | 0 | 25999.67 | 5941.14 | 10672.80 | 46102.00 | -0.318 | 8.90E-18 | 4.74E-16 |
|  |  |  | 1 | 20770.09 | 4387.02 | 8935.20 | 40820.60 |  |  |  |
| 13109-82 | NEGR1 | Q7Z3B1 | 0 | 5242.86 | 984.60 | 3197.40 | 8078.50 | -0.330 | 2.86E-17 | 1.44E-15 |
|  |  |  | 1 | 4201.58 | 897.25 | 805.40 | 7017.60 |  |  |  |
| 3035-80 | IL19 | Q9UHD0 | 0 | 7787.13 | 1636.15 | 3754.60 | 12818.10 | -0.356 | 3.68E-17 | 1.78E-15 |
|  |  |  | 1 | 6134.36 | 1514.92 | 1326.00 | 11957.10 |  |  |  |
| 3024-18 | SERPINF2 | P08697 | 0 | 2044.45 | 271.45 | 1361.10 | 2885.20 | -0.193 | 6.36E-17 | 2.97E-15 |
|  |  |  | 1 | 1788.81 | 244.04 | 1191.30 | 2896.80 |  |  |  |
| 2212-69 | PLAT | P00750 | 0 | 673.00 | 241.63 | 265.30 | 1857.00 | 0.478 | 2.79E-16 | 1.18E-14 |
|  |  |  | 1 | 938.08 | 355.87 | 328.90 | 4183.20 |  |  |  |
| 2579-17 | MMP9 | P14780 | 0 | 13985.20 | 7151.70 | 4161.70 | 35774.30 | 0.792 | 4.49E-16 | 1.83E-14 |
|  |  |  | 1 | 25635.45 | 15912.16 | 5019.50 | 88094.30 |  |  |  |
| 3298-52 | CNTN4 | Q8IWV2 | 0 | 4712.62 | 900.56 | 3089.40 | 7429.00 | -0.302 | 1.12E-15 | 4.43E-14 |
|  |  |  | 1 | 3849.57 | 848.20 | 1300.90 | 7300.00 |  |  |  |
| 3487-32 | CXCL13 | O43927 | 0 | 3615.73 | 1295.54 | 1494.20 | 10672.10 | 0.834 | 1.63E-15 | 6.27E-14 |
|  |  |  | 1 | 8067.23 | 9663.59 | 731.10 | 116887.80 |  |  |  |
| 3352-80 | CA6 | P23280 | 0 | 10064.30 | 5473.11 | 2043.20 | 33597.30 | -0.778 | 2.15E-15 | 8.01E-14 |
|  |  |  | 1 | 5958.36 | 3473.64 | 566.90 | 21976.00 |  |  |  |
| 3043-49 | SPARC | P09486 | 0 | 19391.18 | 14320.61 | 2749.50 | 71022.70 | 0.869 | 3.13E-15 | 1.13E-13 |
|  |  |  | 1 | 32913.96 | 17935.38 | 3677.60 | 99740.00 |  |  |  |
| 5852-6 | S100A12 | P80511 | 0 | 692.53 | 227.64 | 438.20 | 2299.50 | 0.580 | 3.86E-15 | 1.36E-13 |
|  |  |  | 1 | 1178.35 | 1260.96 | 443.10 | 18289.00 |  |  |  |
| 2826-53 | EDA | Q92838 | 0 | 2010.01 | 635.00 | 1116.10 | 5714.20 | -0.366 | 5.32E-15 | 1.83E-13 |
|  |  |  | 1 | 1564.38 | 518.28 | 486.40 | 6302.30 |  |  |  |
| 3580-25 | SERPINA1 | P01009 | 0 | 1055.13 | 341.65 | 565.20 | 2940.60 | 0.426 | 7.21E-15 | 2.37E-13 |
|  |  |  | 1 | 1465.40 | 934.33 | 695.30 | 14618.30 |  |  |  |
| 3449-58 | SERPINA4 | P29622 | 0 | 40778.70 | 5480.41 | 28917.00 | 57496.30 | -0.238 | 1.03E-14 | 3.27E-13 |
|  |  |  | 1 | 34871.33 | 6679.54 | 16945.10 | 83673.70 |  |  |  |
| 2643-57 | CDH3 | P22223 | 0 | 31541.07 | 5289.21 | 21369.10 | 45382.20 | -0.261 | 2.09E-14 | 6.51E-13 |
|  |  |  | 1 | 26521.56 | 5713.62 | 14152.60 | 52692.90 |  |  |  |
| 3322-52 | LRIG3 | Q6UXM1 | 0 | 7795.32 | 1746.45 | 4279.00 | 11878.10 | -0.378 | 3.00E-14 | 9.12E-13 |
|  |  |  | 1 | 6100.13 | 1695.70 | 1557.60 | 11502.00 |  |  |  |
| 13090-17 | S100A6 | P06703 | 0 | 3577.67 | 1720.25 | 1104.30 | 11279.70 | 0.625 | 4.47E-14 | 1.33E-12 |
|  |  |  | 1 | 5695.24 | 4095.80 | 1532.40 | 57889.50 |  |  |  |
| 2797-56 | APOB | P04114 | 0 | 14292.64 | 6215.64 | 5159.20 | 35485.60 | -0.536 | 1.53E-13 | 4.44E-12 |
|  |  |  | 1 | 9789.79 | 4055.10 | 1211.70 | 28398.50 |  |  |  |
| 3435-53 | FN1 | P02751 | 0 | 71592.52 | 20241.15 | 38971.50 | 133788.00 | 0.298 | 3.57E-13 | 1.01E-11 |
|  |  |  | 1 | 86805.84 | 19126.41 | 36246.10 | 158158.90 |  |  |  |
| 3009-3 | TGFBR3 | Q03167 | 0 | 34906.98 | 6849.38 | 20038.20 | 59718.20 | -0.263 | 4.73E-13 | 1.31E-11 |
|  |  |  | 1 | 29195.87 | 6280.91 | 10881.20 | 52709.90 |  |  |  |
| 3651-50 | KDR | P35968 | 0 | 7958.62 | 1682.25 | 4924.90 | 12877.00 | -0.264 | 8.96E-13 | 2.44E-11 |
|  |  |  | 1 | 6633.27 | 1407.90 | 3055.10 | 11131.90 |  |  |  |
| 9185-15 | TFF1 | P04155 | 0 | 8081.40 | 4400.60 | 2586.50 | 30322.20 | -0.708 | 1.20E-12 | 3.20E-11 |
|  |  |  | 1 | 5247.53 | 3406.12 | 291.60 | 27805.40 |  |  |  |
| 5483-1 | RGMA | Q96B86 | 0 | 20951.76 | 2862.22 | 14816.80 | 29139.90 | -0.196 | 2.09E-12 | 5.44E-11 |
|  |  |  | 1 | 18385.03 | 3153.67 | 10325.00 | 28923.90 |  |  |  |
| 4498-62 | NCAM1 | P13591 | 0 | 13269.26 | 3086.49 | 8116.70 | 24209.00 | -0.273 | 2.13E-12 | 5.44E-11 |
|  |  |  | 1 | 10972.34 | 2487.09 | 5744.80 | 21672.40 |  |  |  |
| 5351-52 | HNRNPA2B1 | P22626 | 0 | 3739.06 | 1862.40 | 1829.50 | 16389.00 | 0.683 | 2.27E-12 | 5.69E-11 |
|  |  |  | 1 | 7498.18 | 9958.08 | 1902.60 | 76434.20 |  |  |  |
| 3235-50 | WFIKKN2 | Q8TEU8 | 0 | 8366.33 | 2976.48 | 3590.80 | 27692.30 | -0.389 | 3.24E-12 | 7.98E-11 |
|  |  |  | 1 | 6414.74 | 2147.92 | 2111.40 | 16088.90 |  |  |  |
| 3175-51 | ADAMTS13 | Q76LX8 | 0 | 4718.53 | 1166.27 | 2177.30 | 7223.00 | -0.341 | 4.49E-12 | 1.08E-10 |
|  |  |  | 1 | 3748.18 | 1023.68 | 1020.00 | 7100.20 |  |  |  |
| 3325-2 | MATN2 | O00339 | 0 | 10491.38 | 1910.08 | 6760.50 | 14905.00 | -0.258 | 5.99E-12 | 1.42E-10 |
|  |  |  | 1 | 8836.36 | 1835.60 | 1836.40 | 14022.10 |  |  |  |
| 5116-62 | ROBO2 | Q9HCK4 | 0 | 3875.72 | 682.96 | 2646.40 | 6261.40 | 0.235 | 7.28E-12 | 1.70E-10 |
|  |  |  | 1 | 4591.98 | 958.39 | 2462.50 | 7879.60 |  |  |  |
| 8464-31 | RSPO4 | Q2I0M5 | 0 | 381.53 | 119.93 | 205.50 | 959.60 | 0.437 | 7.77E-12 | 1.78E-10 |
|  |  |  | 1 | 540.91 | 296.07 | 181.60 | 3868.20 |  |  |  |
| 4567-82 | SH2D1A | O60880 | 0 | 5866.81 | 4732.71 | 2999.20 | 44982.70 | 0.488 | 9.06E-12 | 2.04E-10 |
|  |  |  | 1 | 8131.95 | 4715.85 | 3441.80 | 44853.00 |  |  |  |
| 3171-57 | APP | P05067 | 0 | 22175.59 | 13943.90 | 6149.70 | 73743.70 | 0.662 | 1.34E-11 | 2.97E-10 |
|  |  |  | 1 | 34781.23 | 19372.87 | 7233.30 | 121629.30 |  |  |  |
| 2811-27 | ANGPT1 | Q15389 | 0 | 626.98 | 232.46 | 374.30 | 1379.00 | 0.442 | 2.61E-11 | 5.69E-10 |
|  |  |  | 1 | 881.98 | 433.87 | 418.10 | 4001.20 |  |  |  |
| 2711-6 | CNTFR | P26992 | 0 | 8085.95 | 1775.35 | 4774.00 | 12833.70 | -0.356 | 3.46E-11 | 7.39E-10 |
|  |  |  | 1 | 6508.93 | 2131.32 | 1428.60 | 16158.10 |  |  |  |
| 4924-32 | MMP1 | P03956 | 0 | 1750.89 | 1250.42 | 396.60 | 7137.70 | 0.712 | 3.92E-11 | 8.25E-10 |
|  |  |  | 1 | 2911.59 | 2063.24 | 394.20 | 14309.60 |  |  |  |
| 3213-65 | NID1 | P14543 | 0 | 5350.56 | 1090.23 | 3055.10 | 8252.90 | 0.260 | 4.20E-11 | 8.70E-10 |
|  |  |  | 1 | 6456.98 | 1556.37 | 2870.80 | 11965.80 |  |  |  |
| 2819-23 | CDH5 | P33151 | 0 | 14602.31 | 3021.10 | 6907.10 | 23257.60 | -0.265 | 7.12E-11 | 1.45E-09 |
|  |  |  | 1 | 12269.29 | 3416.58 | 5112.60 | 46618.60 |  |  |  |
| 3331-8 | RGMB | Q6NW40 | 0 | 2443.76 | 447.32 | 1471.80 | 3464.90 | -0.247 | 1.08E-10 | 2.17E-09 |
|  |  |  | 1 | 2085.17 | 597.90 | 808.10 | 8989.20 |  |  |  |
| 4546-27 | ADGRE2 | Q9UHX3 | 0 | 1331.03 | 401.27 | 446.30 | 2906.30 | -0.422 | 1.17E-10 | 2.31E-09 |
|  |  |  | 1 | 1469.11 | 8572.82 | 387.80 | 149374.00 |  |  |  |
| 4541-49 | CDON | Q4KMG0 | 0 | 7506.31 | 1604.85 | 3990.10 | 11073.40 | -0.256 | 1.34E-10 | 2.61E-09 |
|  |  |  | 1 | 6308.36 | 1466.44 | 2645.90 | 11301.00 |  |  |  |
| 5534-49 | TNFRSF10B | O14763 | 0 | 404.94 | 74.89 | 307.00 | 632.20 | 0.297 | 2.18E-10 | 4.19E-09 |
|  |  |  | 1 | 520.62 | 276.14 | 314.80 | 3468.20 |  |  |  |
| 3864-5 | RPS7 | P62081 | 0 | 1192.16 | 400.68 | 647.40 | 2691.70 | 0.559 | 2.60E-10 | 4.91E-09 |
|  |  |  | 1 | 2134.76 | 2571.33 | 688.40 | 26936.90 |  |  |  |
| 3040-59 | CCL3 | P10147 | 0 | 1180.76 | 682.41 | 657.70 | 6355.20 | 0.491 | 2.70E-10 | 5.03E-09 |
|  |  |  | 1 | 2065.51 | 6850.10 | 748.30 | 118772.50 |  |  |  |
| 5107-7 | NOTCH1 | P46531 | 0 | 24932.87 | 3373.45 | 17754.30 | 33144.40 | -0.197 | 3.24E-10 | 5.96E-09 |
|  |  |  | 1 | 21931.42 | 3823.82 | 4261.40 | 32665.60 |  |  |  |
| 2480-58 | TIMP3 | P35625 | 0 | 1958.10 | 1508.40 | 574.00 | 7298.30 | 0.690 | 4.04E-10 | 7.32E-09 |
|  |  |  | 1 | 3265.14 | 2684.75 | 586.80 | 19032.30 |  |  |  |
| 4328-2 | BOC | Q9BWV1 | 0 | 1553.42 | 406.09 | 679.70 | 2581.40 | -0.385 | 4.79E-10 | 8.56E-09 |
|  |  |  | 1 | 1222.16 | 399.19 | 205.50 | 2450.80 |  |  |  |
| 2670-67 | CKM | P06732 | 0 | 1216.13 | 1858.78 | 350.90 | 14581.10 | -0.552 | 5.70E-10 | 1.01E-08 |
|  |  |  | 1 | 700.83 | 437.73 | 248.50 | 3745.00 |  |  |  |
| 2681-23 | HGF | P14210 | 0 | 1643.43 | 755.07 | 700.00 | 7048.70 | 0.345 | 5.83E-10 | 1.01E-08 |
|  |  |  | 1 | 2124.86 | 1274.84 | 959.70 | 19109.40 |  |  |  |
| 4568-17 | SLITRK5 | O94991 | 0 | 2306.84 | 684.72 | 495.70 | 3978.70 | -0.500 | 6.55E-10 | 1.12E-08 |
|  |  |  | 1 | 1704.24 | 624.84 | 134.30 | 3922.10 |  |  |  |
| 14125-5 | APOM | O95445 | 0 | 13086.89 | 5152.86 | 5176.90 | 28421.90 | -0.429 | 6.82E-10 | 1.16E-08 |
|  |  |  | 1 | 9809.30 | 3909.09 | 935.40 | 29497.20 |  |  |  |
| 4562-1 | NXPH1 | P58417 | 0 | 4403.46 | 1124.89 | 2303.40 | 7256.00 | 0.359 | 7.59E-10 | 1.27E-08 |
|  |  |  | 1 | 5824.56 | 2123.74 | 1205.10 | 16638.10 |  |  |  |
| 4194-26 | ANP32B | Q92688 | 0 | 191.78 | 59.34 | 126.20 | 726.10 | 0.223 | 8.26E-10 | 1.36E-08 |
|  |  |  | 1 | 224.83 | 64.96 | 146.30 | 690.10 |  |  |  |
| 2948-58 | GHR | P10912 | 0 | 1625.05 | 540.48 | 713.80 | 3342.30 | -0.378 | 1.17E-09 | 1.91E-08 |
|  |  |  | 1 | 1267.66 | 459.26 | 389.20 | 3766.80 |  |  |  |
| 14136-234 | CD93 | Q9NPY3 | 0 | 13014.87 | 2861.60 | 5802.90 | 20484.50 | -0.253 | 1.30E-09 | 2.10E-08 |
|  |  |  | 1 | 10982.06 | 2621.08 | 1790.50 | 22329.20 |  |  |  |
| 3727-35 | PYY | P10082 | 0 | 3307.70 | 1556.15 | 1128.70 | 7489.80 | -0.485 | 1.43E-09 | 2.28E-08 |
|  |  |  | 1 | 2419.46 | 1384.23 | 630.00 | 12024.70 |  |  |  |
| 3060-43 | C9 | P02748 | 0 | 76347.23 | 16902.24 | 37731.80 | 148314.10 | 0.201 | 1.52E-09 | 2.39E-08 |
|  |  |  | 1 | 86978.12 | 15873.32 | 42731.20 | 163284.30 |  |  |  |
| 2617-56 | ERBB3 | P21860 | 0 | 379.30 | 232.59 | 236.90 | 2444.20 | -0.245 | 1.75E-09 | 2.72E-08 |
|  |  |  | 1 | 308.29 | 94.86 | 185.20 | 1551.90 |  |  |  |
| 5470-69 | MED1 | Q15648 | 0 | 85491.57 | 15135.79 | 53698.40 | 127523.50 | -0.231 | 1.85E-09 | 2.84E-08 |
|  |  |  | 1 | 73752.18 | 17345.61 | 29665.60 | 146004.80 |  |  |  |
| 13130-150 | HK2 | P52789 | 0 | 799.85 | 339.70 | 448.50 | 3240.50 | 0.592 | 2.22E-09 | 3.35E-08 |
|  |  |  | 1 | 1605.49 | 2477.19 | 357.10 | 24674.70 |  |  |  |
| 3177-49 | CA4 | P22748 | 0 | 2167.19 | 472.92 | 965.90 | 3236.70 | -0.329 | 2.24E-09 | 3.35E-08 |
|  |  |  | 1 | 1774.46 | 549.84 | 358.90 | 5088.30 |  |  |  |
| 4771-10 | SMPDL3A | Q92484 | 0 | 3814.38 | 1248.75 | 2155.10 | 11453.00 | 0.360 | 2.33E-09 | 3.45E-08 |
|  |  |  | 1 | 5166.12 | 3125.45 | 2486.80 | 33235.00 |  |  |  |
| 3302-58 | CST7 | O76096 | 0 | 1053.98 | 2613.49 | 467.10 | 26836.80 | 0.441 | 2.97E-09 | 4.31E-08 |
|  |  |  | 1 | 1239.26 | 1207.14 | 528.70 | 18148.80 |  |  |  |
| 4284-18 | RBM39 | Q14498 | 0 | 426.69 | 127.71 | 284.50 | 1272.60 | 0.441 | 2.96E-09 | 4.31E-08 |
|  |  |  | 1 | 669.50 | 655.97 | 286.90 | 5990.00 |  |  |  |
| 5335-73 | ANXA6 | P08133 | 0 | 6499.20 | 4782.08 | 831.80 | 17980.90 | 0.725 | 3.36E-09 | 4.82E-08 |
|  |  |  | 1 | 9476.10 | 4556.87 | 748.70 | 22774.00 |  |  |  |
| 2683-1 | C3 | P01024 | 0 | 12050.63 | 3356.30 | 4420.00 | 21563.90 | -0.355 | 3.44E-09 | 4.88E-08 |
|  |  |  | 1 | 9689.60 | 3789.74 | 1843.40 | 41143.30 |  |  |  |
| 4775-34 | GSN | P06396 | 0 | 936.42 | 137.91 | 582.30 | 1250.40 | -0.178 | 3.88E-09 | 5.44E-08 |
|  |  |  | 1 | 832.93 | 157.79 | 493.40 | 1513.70 |  |  |  |
| 3290-50 | CD109 | Q6YHK3 | 0 | 4346.76 | 1179.62 | 1147.10 | 7219.70 | -0.404 | 4.24E-09 | 5.88E-08 |
|  |  |  | 1 | 3431.94 | 1364.78 | 688.70 | 9294.10 |  |  |  |
| 3344-60 | SERPINC1 | P01008 | 0 | 125511.73 | 13774.39 | 93881.40 | 165761.20 | -0.127 | 4.68E-09 | 6.43E-08 |
|  |  |  | 1 | 115192.83 | 14491.10 | 48274.30 | 158768.30 |  |  |  |
| 4246-40 | L1CAM | P32004 | 0 | 541.42 | 141.96 | 302.00 | 1430.50 | -0.243 | 6.49E-09 | 8.82E-08 |
|  |  |  | 1 | 459.02 | 116.68 | 184.60 | 926.60 |  |  |  |
| 4867-15 | VEGFA | P15692 | 0 | 778.89 | 281.40 | 377.00 | 1837.00 | 0.448 | 7.66E-09 | 1.03E-07 |
|  |  |  | 1 | 1234.34 | 1863.21 | 417.30 | 26927.30 |  |  |  |
| 5140-56 | UNC5D | Q6UXZ4 | 0 | 6182.91 | 1978.97 | 2906.60 | 11544.30 | -0.472 | 7.83E-09 | 1.04E-07 |
|  |  |  | 1 | 4699.04 | 1892.32 | 348.60 | 12416.80 |  |  |  |
| 2182-54 | C4A C4B | P0C0L4 P0C0L5 | 0 | 173.41 | 187.35 | 31.80 | 1088.20 | 0.604 | 1.13E-08 | 1.49E-07 |
|  |  |  | 1 | 220.94 | 134.65 | 48.60 | 857.10 |  |  |  |
| 3603-60 | CLEC7A | Q9BXN2 | 0 | 904.83 | 288.49 | 603.80 | 2313.40 | 0.357 | 1.28E-08 | 1.67E-07 |
|  |  |  | 1 | 1255.17 | 1124.66 | 642.80 | 13524.30 |  |  |  |
| 14133-93 | IL1R2 | P27930 | 0 | 13240.58 | 2849.49 | 5676.00 | 18628.80 | -0.264 | 1.33E-08 | 1.72E-07 |
|  |  |  | 1 | 11184.64 | 3088.48 | 1813.10 | 22991.40 |  |  |  |
| 13124-20 | ISLR2 | Q6UXK2 | 0 | 7652.49 | 2112.81 | 3790.00 | 14591.70 | -0.321 | 1.56E-08 | 2.00E-07 |
|  |  |  | 1 | 6219.23 | 1893.38 | 522.80 | 12112.90 |  |  |  |
| 2421-7 | BDNF | P23560 | 0 | 1085.57 | 431.17 | 541.10 | 2466.10 | 0.368 | 1.71E-08 | 2.17E-07 |
|  |  |  | 1 | 1415.29 | 629.54 | 480.40 | 4680.40 |  |  |  |
| 4414-69 | SFTPD | P35247 | 0 | 7300.31 | 6880.82 | 1870.00 | 63722.70 | 0.789 | 1.75E-08 | 2.20E-07 |
|  |  |  | 1 | 18612.72 | 33367.75 | 1907.20 | 213186.50 |  |  |  |
| 4831-4 | SELL | P14151 | 0 | 4784.77 | 891.91 | 3327.90 | 7103.40 | -0.182 | 2.20E-08 | 2.74E-07 |
|  |  |  | 1 | 4225.14 | 808.64 | 2262.30 | 6985.30 |  |  |  |
| 2526-53 | TNFRSF11B | O00300 | 0 | 15365.90 | 3019.61 | 7662.30 | 22305.10 | -0.252 | 2.29E-08 | 2.82E-07 |
|  |  |  | 1 | 13106.62 | 3327.17 | 3718.90 | 21596.00 |  |  |  |
| 2658-27 | NTRK3 | Q16288 | 0 | 5469.75 | 1470.89 | 1368.90 | 8721.50 | -0.369 | 2.42E-08 | 2.95E-07 |
|  |  |  | 1 | 4370.23 | 1539.77 | 815.60 | 11924.50 |  |  |  |
| 10339-48 | ENO2 | P09104 | 0 | 44532.86 | 18091.81 | 14892.20 | 121807.20 | -0.563 | 2.69E-08 | 3.25E-07 |
|  |  |  | 1 | 32483.18 | 16397.39 | 959.50 | 110968.80 |  |  |  |
| 4544-4 | PPBP | P02775 | 0 | 28603.27 | 26479.38 | 1186.10 | 129278.00 | 0.850 | 2.74E-08 | 3.26E-07 |
|  |  |  | 1 | 46479.34 | 34300.03 | 1820.20 | 178013.00 |  |  |  |
| 14134-49 | AMIGO2 | Q86SJ2 | 0 | 2763.69 | 612.45 | 1286.80 | 4975.30 | -0.312 | 2.75E-08 | 3.26E-07 |
|  |  |  | 1 | 2298.08 | 724.82 | 411.50 | 6391.60 |  |  |  |
| 4499-21 | PDGFA | P04085 | 0 | 16034.58 | 11813.59 | 1103.10 | 50164.00 | 0.665 | 3.05E-08 | 3.55E-07 |
|  |  |  | 1 | 22906.21 | 12278.67 | 1418.60 | 85863.10 |  |  |  |
| 3894-15 | NAGK | Q9UJ70 | 0 | 1173.01 | 357.38 | 502.60 | 2271.60 | 0.354 | 3.04E-08 | 3.55E-07 |
|  |  |  | 1 | 1606.75 | 1682.09 | 586.50 | 28677.90 |  |  |  |
| 2790-54 | PPBP | P02775 | 0 | 9854.43 | 9393.28 | 430.20 | 50740.70 | 0.844 | 3.23E-08 | 3.73E-07 |
|  |  |  | 1 | 15972.31 | 12289.37 | 672.40 | 68404.70 |  |  |  |
| 4467-49 | SPARCL1 | Q14515 | 0 | 14316.35 | 3474.01 | 8636.10 | 25025.30 | -0.229 | 3.59E-08 | 4.11E-07 |
|  |  |  | 1 | 12226.33 | 2851.54 | 2015.10 | 25165.00 |  |  |  |
| 3707-12 | ALB | P02768 | 0 | 10477.46 | 1846.50 | 6254.00 | 16473.50 | -0.188 | 4.13E-08 | 4.69E-07 |
|  |  |  | 1 | 9255.81 | 1984.72 | 1928.20 | 22675.70 |  |  |  |
| 3179-51 | CTSA | P10619 | 0 | 9896.83 | 4844.42 | 2305.20 | 32451.10 | 0.339 | 4.61E-08 | 5.19E-07 |
|  |  |  | 1 | 12090.05 | 4093.55 | 3140.00 | 28197.70 |  |  |  |
| 14124-6 | EFNA2 | O43921 | 0 | 1290.05 | 341.58 | 778.30 | 4007.10 | -0.192 | 4.88E-08 | 5.44E-07 |
|  |  |  | 1 | 1127.88 | 244.43 | 540.20 | 2230.00 |  |  |  |
| 5482-61 | RBP4 | P02753 | 0 | 281.74 | 54.95 | 186.60 | 610.50 | -0.193 | 5.44E-08 | 6.02E-07 |
|  |  |  | 1 | 250.45 | 104.18 | 136.00 | 1867.50 |  |  |  |
| 5069-9 | CD55 | P08174 | 0 | 19647.97 | 4049.76 | 10233.00 | 33065.30 | -0.198 | 6.64E-08 | 7.28E-07 |
|  |  |  | 1 | 17188.21 | 3876.22 | 7881.20 | 35997.20 |  |  |  |
| 3037-62 | IL1B | P01584 | 0 | 2370.83 | 1177.34 | 1186.10 | 11480.00 | 0.343 | 7.09E-08 | 7.71E-07 |
|  |  |  | 1 | 3122.21 | 2226.40 | 1258.20 | 30436.80 |  |  |  |
| 4149-8 | PDGFB | P01127 | 0 | 44009.19 | 34460.80 | 1072.40 | 124783.50 | 0.768 | 7.67E-08 | 8.25E-07 |
|  |  |  | 1 | 65895.86 | 36771.27 | 893.70 | 184370.90 |  |  |  |
| 5412-53 | CD27 | P26842 | 0 | 7848.80 | 1129.68 | 5624.20 | 11263.90 | -0.162 | 7.71E-08 | 8.25E-07 |
|  |  |  | 1 | 7066.57 | 1382.45 | 1890.00 | 18925.90 |  |  |  |
| 2697-7 | PF4 | P02776 | 0 | 50937.80 | 52543.82 | 3056.20 | 211148.30 | 0.853 | 7.84E-08 | 8.32E-07 |
|  |  |  | 1 | 80940.73 | 60647.42 | 3323.70 | 238806.50 |  |  |  |
| 2475-1 | KIT | P10721 | 0 | 18017.86 | 5113.85 | 8892.30 | 30839.80 | -0.288 | 8.21E-08 | 8.64E-07 |
|  |  |  | 1 | 14891.04 | 4763.09 | 4245.00 | 31609.30 |  |  |  |
| 4297-62 | SPON1 | Q9HCB6 | 0 | 2420.70 | 482.40 | 1565.00 | 4405.20 | 0.244 | 8.30E-08 | 8.67E-07 |
|  |  |  | 1 | 2935.64 | 912.76 | 1068.30 | 8051.50 |  |  |  |
| 5122-92 | SEMA6A | Q9H2E6 | 0 | 11969.14 | 2764.16 | 4497.00 | 19095.00 | -0.399 | 8.69E-08 | 9.01E-07 |
|  |  |  | 1 | 9640.33 | 3437.16 | 1027.40 | 19498.30 |  |  |  |
| 2834-54 | KLK8 | O60259 | 0 | 3205.26 | 1057.83 | 1019.70 | 6374.40 | -0.372 | 1.02E-07 | 1.04E-06 |
|  |  |  | 1 | 2552.41 | 1036.86 | 542.60 | 6875.30 |  |  |  |
| 2190-55 | F11 | P03951 | 0 | 1852.59 | 302.18 | 1260.80 | 2725.80 | -0.167 | 1.17E-07 | 1.18E-06 |
|  |  |  | 1 | 1663.80 | 420.26 | 911.70 | 6784.70 |  |  |  |
| 3364-76 | CTSV | O60911 | 0 | 1526.27 | 504.44 | 683.90 | 3555.20 | -0.339 | 1.25E-07 | 1.26E-06 |
|  |  |  | 1 | 1243.37 | 523.92 | 221.40 | 4802.20 |  |  |  |
| 4450-26 | HNRNPAB | Q99729 | 0 | 2044.95 | 788.95 | 1101.80 | 4259.90 | 0.500 | 1.38E-07 | 1.37E-06 |
|  |  |  | 1 | 3637.81 | 5604.49 | 1015.10 | 74620.00 |  |  |  |
| 2987-37 | HIST1H1C | P16403 | 0 | 2082.72 | 3602.00 | 565.40 | 33430.70 | 0.808 | 1.58E-07 | 1.56E-06 |
|  |  |  | 1 | 5107.08 | 8848.02 | 598.70 | 53723.00 |  |  |  |
| 4859-6 | BMPR1A | P36894 | 0 | 880.86 | 237.39 | 398.10 | 1602.80 | -0.279 | 1.59E-07 | 1.56E-06 |
|  |  |  | 1 | 739.36 | 248.10 | 194.60 | 1756.10 |  |  |  |
| 2835-1 | XRCC6 | P12956 | 0 | 313.81 | 81.51 | 226.40 | 768.50 | 0.334 | 1.68E-07 | 1.64E-06 |
|  |  |  | 1 | 442.62 | 408.53 | 162.20 | 4409.70 |  |  |  |
| 4556-10 | IL34 | Q6ZMJ4 | 0 | 811.59 | 250.79 | 525.70 | 2622.40 | -0.227 | 1.77E-07 | 1.71E-06 |
|  |  |  | 1 | 694.52 | 167.42 | 222.10 | 1293.90 |  |  |  |
| 4232-19 | IGF1R | P08069 | 0 | 8294.54 | 1757.77 | 4592.70 | 12802.40 | -0.252 | 2.08E-07 | 2.00E-06 |
|  |  |  | 1 | 7101.73 | 1989.10 | 1686.50 | 13541.50 |  |  |  |
| 5383-14 | TNFRSF13C | Q96RJ3 | 0 | 3224.24 | 2965.08 | 1662.30 | 28252.20 | -0.291 | 2.13E-07 | 2.03E-06 |
|  |  |  | 1 | 2512.59 | 1317.33 | 1222.80 | 17187.70 |  |  |  |
| 3773-15 | TEK | Q02763 | 0 | 2307.65 | 717.09 | 1611.30 | 8441.30 | -0.201 | 2.63E-07 | 2.48E-06 |
|  |  |  | 1 | 2023.45 | 739.56 | 1031.00 | 11673.70 |  |  |  |
| 2429-27 | C8A C8B C8G | P07357 P07358 P07360 | 0 | 4040.33 | 1030.11 | 2021.90 | 8546.00 | -0.245 | 2.82E-07 | 2.65E-06 |
|  |  |  | 1 | 3453.64 | 1087.41 | 1015.60 | 13169.80 |  |  |  |
| 4842-62 | GPC3 | P51654 | 0 | 1058.58 | 358.50 | 502.90 | 2439.10 | -0.327 | 3.12E-07 | 2.91E-06 |
|  |  |  | 1 | 871.86 | 448.94 | 262.90 | 5442.90 |  |  |  |
| 2813-11 | AGRP | O00253 | 0 | 2099.85 | 568.04 | 774.40 | 3571.90 | -0.330 | 3.32E-07 | 3.08E-06 |
|  |  |  | 1 | 1741.54 | 691.99 | 371.50 | 4408.00 |  |  |  |
| 5480-49 | CCL5 | P13501 | 0 | 42431.45 | 28873.72 | 1111.60 | 137687.00 | 0.614 | 3.43E-07 | 3.15E-06 |
|  |  |  | 1 | 60339.86 | 30659.05 | 1448.60 | 163569.60 |  |  |  |
| 4962-52 | CDNF | Q49AH0 | 0 | 4318.90 | 860.63 | 2630.40 | 8600.50 | -0.241 | 3.45E-07 | 3.15E-06 |
|  |  |  | 1 | 3816.76 | 2309.30 | 542.30 | 33410.60 |  |  |  |
| 4931-59 | F3 | P13726 | 0 | 1923.72 | 4511.30 | 615.70 | 46302.70 | -0.425 | 3.74E-07 | 3.39E-06 |
|  |  |  | 1 | 1409.09 | 3974.78 | 245.70 | 68796.00 |  |  |  |
| 3220-40 | RET | P07949 | 0 | 1761.00 | 866.04 | 877.30 | 7100.70 | -0.339 | 4.47E-07 | 4.02E-06 |
|  |  |  | 1 | 1393.77 | 634.03 | 410.40 | 6277.50 |  |  |  |
| 3000-66 | MBL2 | P11226 | 0 | 11656.49 | 6059.08 | 2415.90 | 30469.00 | 0.493 | 5.10E-07 | 4.56E-06 |
|  |  |  | 1 | 16434.24 | 8070.84 | 1781.00 | 38640.10 |  |  |  |
| 2611-72 | TYRO3 | Q06418 | 0 | 632.37 | 217.59 | 309.20 | 1913.50 | -0.248 | 5.29E-07 | 4.70E-06 |
|  |  |  | 1 | 569.09 | 870.74 | 269.70 | 15441.50 |  |  |  |
| 4891-50 | GCG | P01275 | 0 | 5854.88 | 3576.03 | 905.30 | 17594.60 | -0.542 | 5.41E-07 | 4.77E-06 |
|  |  |  | 1 | 4091.02 | 2904.60 | 691.70 | 19148.50 |  |  |  |
| 8474-6 | ROR1 | Q01973 | 0 | 663.97 | 132.09 | 413.30 | 999.00 | -0.188 | 5.56E-07 | 4.87E-06 |
|  |  |  | 1 | 586.50 | 132.18 | 149.80 | 1043.60 |  |  |  |
| 5954-62 | PTH | P01270 | 0 | 4259.14 | 5455.12 | 1542.90 | 55647.10 | -0.352 | 5.61E-07 | 4.88E-06 |
|  |  |  | 1 | 3021.14 | 1688.53 | 718.70 | 23978.80 |  |  |  |
| 3029-52 | CD209 | Q9NNX6 | 0 | 1778.92 | 653.51 | 884.90 | 4232.20 | -0.285 | 6.08E-07 | 5.22E-06 |
|  |  |  | 1 | 1461.26 | 540.41 | 661.20 | 3674.00 |  |  |  |
| 11516-7 | FABP1 | P07148 | 0 | 5336.78 | 2374.09 | 861.90 | 13458.10 | -0.487 | 6.05E-07 | 5.22E-06 |
|  |  |  | 1 | 4082.55 | 2510.75 | 404.60 | 15807.40 |  |  |  |
| 5026-66 | RPS3 | P23396 | 0 | 690.41 | 322.03 | 352.70 | 2227.40 | 0.576 | 6.46E-07 | 5.51E-06 |
|  |  |  | 1 | 2070.07 | 12905.73 | 303.00 | 221968.10 |  |  |  |
| 5837-49 | LIFR | P42702 | 0 | 5474.79 | 1200.30 | 2570.20 | 8332.60 | -0.274 | 6.68E-07 | 5.66E-06 |
|  |  |  | 1 | 4670.46 | 1544.30 | 527.30 | 11159.70 |  |  |  |
| 4706-17 | EPB41 | P11171 | 0 | 10551.11 | 5384.96 | 3219.30 | 39891.50 | 0.442 | 7.57E-07 | 6.37E-06 |
|  |  |  | 1 | 15060.19 | 10783.35 | 3335.00 | 113616.90 |  |  |  |
| 4187-49 | PGD | P52209 | 0 | 23779.37 | 22391.00 | 2047.50 | 102158.50 | 0.833 | 7.64E-07 | 6.39E-06 |
|  |  |  | 1 | 44130.46 | 40008.91 | 1510.50 | 229990.20 |  |  |  |
| 3299-29 | CNTN5 | O94779 | 0 | 669.38 | 134.53 | 515.40 | 1681.10 | -0.136 | 7.93E-07 | 6.59E-06 |
|  |  |  | 1 | 609.56 | 122.59 | 363.20 | 1635.30 |  |  |  |
| 2973-15 | CD36 | P16671 | 0 | 14770.77 | 4501.20 | 7201.60 | 29427.90 | -0.274 | 7.98E-07 | 6.59E-06 |
|  |  |  | 1 | 12330.97 | 4040.68 | 4123.50 | 27964.00 |  |  |  |
| 2633-52 | IL13RA1 | P78552 | 0 | 3472.58 | 4648.71 | 1577.80 | 48873.20 | -0.285 | 8.79E-07 | 7.21E-06 |
|  |  |  | 1 | 2600.52 | 861.69 | 591.60 | 7161.90 |  |  |  |
| 2630-12 | IL1RAP | Q9NPH3 | 0 | 32837.06 | 12056.72 | 17253.50 | 88093.90 | -0.285 | 1.01E-06 | 8.21E-06 |
|  |  |  | 1 | 27159.77 | 10330.96 | 10829.30 | 72338.00 |  |  |  |
| 2925-9 | SERPINE1 | P05121 | 0 | 1302.88 | 688.76 | 282.90 | 3211.90 | 0.480 | 1.17E-06 | 9.47E-06 |
|  |  |  | 1 | 1847.22 | 1109.61 | 296.60 | 6509.30 |  |  |  |
| 14116-129 | S100A4 | P26447 | 0 | 1792.85 | 369.61 | 659.60 | 3224.90 | 0.248 | 1.19E-06 | 9.51E-06 |
|  |  |  | 1 | 2227.06 | 1288.26 | 1056.90 | 20575.90 |  |  |  |
| 3030-3 | CLEC4M | Q9H2X3 | 0 | 53570.92 | 7460.83 | 35801.30 | 72289.50 | -0.107 | 1.18E-06 | 9.51E-06 |
|  |  |  | 1 | 49660.96 | 6378.24 | 35449.20 | 71820.50 |  |  |  |
| 9199-6 | UBE2G2 | P60604 | 0 | 7199.77 | 1869.88 | 4235.20 | 13456.30 | -0.208 | 1.20E-06 | 9.56E-06 |
|  |  |  | 1 | 6256.48 | 1801.71 | 3155.90 | 19681.60 |  |  |  |
| 2789-26 | MMP7 | P09237 | 0 | 6218.68 | 2464.37 | 2730.80 | 17127.30 | 0.405 | 1.44E-06 | 1.14E-05 |
|  |  |  | 1 | 8801.34 | 4287.95 | 385.40 | 30486.10 |  |  |  |
| 4495-33 | KNG1 | P01042 | 0 | 34360.93 | 9683.52 | 10648.90 | 79202.50 | -0.216 | 1.49E-06 | 1.18E-05 |
|  |  |  | 1 | 29458.35 | 8418.54 | 9747.80 | 90559.00 |  |  |  |
| 14131-37 | EFNB2 | P52799 | 0 | 14131.72 | 4106.81 | 3837.00 | 24842.10 | -0.497 | 1.51E-06 | 1.18E-05 |
|  |  |  | 1 | 11078.51 | 5023.26 | 317.70 | 40522.90 |  |  |  |
| 5532-53 | FGFR1 | P11362 | 0 | 834.17 | 152.52 | 526.90 | 1455.60 | -0.159 | 1.53E-06 | 1.18E-05 |
|  |  |  | 1 | 751.05 | 167.16 | 393.80 | 1908.00 |  |  |  |
| 4459-68 | PCSK7 | Q16549 | 0 | 2731.81 | 812.88 | 874.60 | 6071.20 | -0.341 | 1.53E-06 | 1.18E-05 |
|  |  |  | 1 | 2387.31 | 3495.39 | 429.30 | 61258.80 |  |  |  |
| 3358-51 | CDK5 CDK5R1 | Q00535 Q15078 | 0 | 1461.01 | 528.79 | 895.60 | 4372.90 | -0.197 | 1.58E-06 | 1.22E-05 |
|  |  |  | 1 | 1264.50 | 444.34 | 468.60 | 7563.00 |  |  |  |
| 2991-9 | IL1R1 | P14778 | 0 | 9869.02 | 2745.34 | 2878.70 | 17847.90 | -0.412 | 1.64E-06 | 1.25E-05 |
|  |  |  | 1 | 7989.38 | 3271.86 | 344.90 | 18444.20 |  |  |  |
| 13132-14 | SEMA5A | Q13591 | 0 | 7779.46 | 1944.01 | 4732.50 | 14829.70 | -0.277 | 1.69E-06 | 1.28E-05 |
|  |  |  | 1 | 6648.86 | 2383.78 | 820.90 | 18072.10 |  |  |  |
| 3514-49 | PRTN3 | P24158 | 0 | 4832.19 | 1670.54 | 2065.60 | 10575.20 | 0.369 | 1.77E-06 | 1.33E-05 |
|  |  |  | 1 | 6909.67 | 6191.97 | 2329.50 | 65146.40 |  |  |  |
| 2418-55 | APOE | P02649 | 0 | 63249.24 | 23571.19 | 18025.90 | 130179.40 | -0.327 | 2.17E-06 | 1.63E-05 |
|  |  |  | 1 | 51454.77 | 23890.56 | 12463.00 | 271249.80 |  |  |  |
| 4472-5 | TPM2 | P07951 | 0 | 2298.54 | 604.45 | 1256.80 | 6631.80 | -0.229 | 2.20E-06 | 1.64E-05 |
|  |  |  | 1 | 2013.68 | 949.32 | 529.20 | 15653.00 |  |  |  |
| 2211-9 | TIMP1 | P01033 | 0 | 78.83 | 13.02 | 53.50 | 125.30 | 0.185 | 2.39E-06 | 1.77E-05 |
|  |  |  | 1 | 91.31 | 25.62 | 23.30 | 300.00 |  |  |  |
| 5353-89 | IL1RN | P18510 | 0 | 4875.39 | 2134.69 | 2195.10 | 15392.30 | 0.290 | 2.53E-06 | 1.87E-05 |
|  |  |  | 1 | 5989.38 | 2659.62 | 2264.10 | 20591.90 |  |  |  |
| 4588-1 | PPY | P01298 | 0 | 7034.68 | 5613.70 | 1279.20 | 43716.60 | -0.566 | 2.58E-06 | 1.89E-05 |
|  |  |  | 1 | 4880.79 | 3926.68 | 385.10 | 29501.70 |  |  |  |
| 3004-67 | PDCD1LG2 | Q9BQ51 | 0 | 4261.88 | 735.87 | 2830.00 | 6472.10 | -0.216 | 2.67E-06 | 1.95E-05 |
|  |  |  | 1 | 3833.78 | 2055.24 | 1724.40 | 30287.30 |  |  |  |
| 3326-58 | CADM1 | Q9BY67 | 0 | 2552.62 | 692.72 | 704.60 | 4350.10 | -0.322 | 2.72E-06 | 1.97E-05 |
|  |  |  | 1 | 2121.64 | 762.38 | 376.10 | 5978.80 |  |  |  |
| 3431-54 | EPHA1 | P21709 | 0 | 8448.58 | 3587.10 | 1136.00 | 20239.80 | -0.517 | 3.40E-06 | 2.45E-05 |
|  |  |  | 1 | 6429.37 | 4054.00 | 386.80 | 45696.30 |  |  |  |
| 2837-3 | MET | P08581 | 0 | 9899.01 | 2267.80 | 5856.10 | 18151.90 | -0.188 | 3.64E-06 | 2.61E-05 |
|  |  |  | 1 | 8714.19 | 1938.62 | 1332.90 | 15161.20 |  |  |  |
| 3042-7 | MB | P02144 | 0 | 1551.00 | 626.14 | 486.50 | 5528.10 | -0.294 | 3.67E-06 | 2.62E-05 |
|  |  |  | 1 | 1286.68 | 648.44 | 204.30 | 7806.80 |  |  |  |
| 5103-30 | CD200R1 | Q8TD46 | 0 | 1182.74 | 219.48 | 814.30 | 1985.70 | -0.211 | 4.30E-06 | 3.03E-05 |
|  |  |  | 1 | 1061.65 | 481.91 | 496.10 | 5421.00 |  |  |  |
| 14114-18 | PIANP | Q8IYJ0 | 0 | 5834.76 | 2399.39 | 2707.70 | 23274.20 | -0.278 | 4.28E-06 | 3.03E-05 |
|  |  |  | 1 | 4848.95 | 1738.48 | 660.30 | 11595.00 |  |  |  |
| 3810-50 | FGR | P09769 | 0 | 690.55 | 574.95 | 277.00 | 5075.70 | 0.521 | 4.91E-06 | 3.44E-05 |
|  |  |  | 1 | 1276.85 | 2738.15 | 291.60 | 38987.30 |  |  |  |
| 4878-3 | F10 | P00742 | 0 | 6472.76 | 1254.15 | 2417.10 | 9182.60 | -0.152 | 5.24E-06 | 3.66E-05 |
|  |  |  | 1 | 5772.57 | 929.71 | 1953.70 | 8598.30 |  |  |  |
| 9182-3 | LRP1 | Q07954 | 0 | 2090.22 | 1216.17 | 685.50 | 12036.30 | -0.262 | 5.63E-06 | 3.91E-05 |
|  |  |  | 1 | 1701.40 | 573.38 | 670.30 | 4422.90 |  |  |  |
| 4996-66 | HRG | P04196 | 0 | 4917.31 | 1223.86 | 2985.60 | 7987.50 | -0.211 | 6.82E-06 | 4.69E-05 |
|  |  |  | 1 | 4290.62 | 1204.82 | 1285.70 | 8678.10 |  |  |  |
| 2615-60 | EFNA5 | P52803 | 0 | 3598.88 | 855.56 | 1521.20 | 5295.40 | -0.317 | 6.79E-06 | 4.69E-05 |
|  |  |  | 1 | 3171.60 | 3275.39 | 321.10 | 56504.10 |  |  |  |
| 5509-7 | EGF | P01133 | 0 | 908.50 | 306.76 | 356.10 | 1838.20 | 0.315 | 6.91E-06 | 4.72E-05 |
|  |  |  | 1 | 1316.81 | 3159.96 | 360.60 | 55222.70 |  |  |  |
| 4337-49 | CRP | P02741 | 0 | 63773.73 | 38658.60 | 7842.20 | 198971.20 | 0.475 | 7.13E-06 | 4.85E-05 |
|  |  |  | 1 | 84526.16 | 42214.52 | 10258.70 | 202632.70 |  |  |  |
| 2578-67 | CCL2 | P13500 | 0 | 1161.15 | 372.33 | 721.70 | 3318.30 | 0.298 | 8.52E-06 | 5.76E-05 |
|  |  |  | 1 | 1549.10 | 1097.14 | 654.90 | 13234.30 |  |  |  |
| 4997-19 | EIF4A3 | P38919 | 0 | 649.35 | 245.88 | 418.70 | 1883.60 | 0.339 | 8.64E-06 | 5.81E-05 |
|  |  |  | 1 | 997.91 | 1736.88 | 423.00 | 23847.00 |  |  |  |
| 8480-29 | EFEMP1 | Q12805 | 0 | 1846.54 | 367.90 | 1041.30 | 3135.40 | 0.156 | 9.15E-06 | 6.09E-05 |
|  |  |  | 1 | 2067.42 | 472.78 | 1143.40 | 4569.70 |  |  |  |
| 3348-49 | BMP1 | P13497 | 0 | 5166.58 | 1344.98 | 2482.40 | 9556.50 | -0.232 | 9.14E-06 | 6.09E-05 |
|  |  |  | 1 | 4474.59 | 1396.51 | 890.10 | 10993.90 |  |  |  |
| 2571-12 | IGFBP3 | P17936 | 0 | 1641.63 | 751.10 | 455.40 | 5967.50 | -0.277 | 1.03E-05 | 6.86E-05 |
|  |  |  | 1 | 1334.83 | 462.64 | 414.40 | 2621.20 |  |  |  |
| 3535-84 | DKK1 | O94907 | 0 | 28324.48 | 13634.74 | 10899.70 | 70923.10 | 0.379 | 1.08E-05 | 7.06E-05 |
|  |  |  | 1 | 37369.43 | 17719.10 | 5587.00 | 105488.80 |  |  |  |
| 2706-69 | SERPINA7 | P05543 | 0 | 48249.70 | 6438.22 | 35123.30 | 70714.40 | -0.114 | 1.08E-05 | 7.06E-05 |
|  |  |  | 1 | 44753.65 | 7133.19 | 25735.60 | 72732.80 |  |  |  |
| 2620-4 | IL6ST | P40189 | 0 | 8267.77 | 1389.13 | 5051.50 | 11980.60 | -0.157 | 1.08E-05 | 7.06E-05 |
|  |  |  | 1 | 7474.35 | 1501.75 | 1323.70 | 12140.20 |  |  |  |
| 3312-64 | FCGR1A | P12314 | 0 | 381.77 | 115.92 | 266.00 | 979.60 | 0.291 | 1.11E-05 | 7.18E-05 |
|  |  |  | 1 | 615.78 | 2401.93 | 270.80 | 41867.80 |  |  |  |
| 3378-49 | KLK7 | P49862 | 0 | 1663.74 | 555.48 | 597.40 | 4910.80 | -0.248 | 1.12E-05 | 7.23E-05 |
|  |  |  | 1 | 1411.48 | 471.05 | 343.30 | 3215.50 |  |  |  |
| 3280-49 | ACAN | P16112 | 0 | 559.09 | 216.25 | 326.20 | 1975.60 | -0.184 | 1.15E-05 | 7.37E-05 |
|  |  |  | 1 | 490.34 | 192.29 | 287.80 | 2781.40 |  |  |  |
| 5088-175 | IL23R | Q5VWK5 | 0 | 4155.89 | 1129.82 | 1692.90 | 7137.80 | -0.287 | 1.21E-05 | 7.72E-05 |
|  |  |  | 1 | 3605.33 | 1989.61 | 900.00 | 28442.80 |  |  |  |
| 2723-9 | IL37 | Q9NZH6 | 0 | 2595.00 | 576.25 | 1703.30 | 5366.40 | -0.164 | 1.35E-05 | 8.60E-05 |
|  |  |  | 1 | 2344.79 | 755.22 | 1192.00 | 8959.10 |  |  |  |
| 4153-11 | SERPINA3 | P01011 | 0 | 8150.87 | 1940.72 | 4938.60 | 16086.50 | 0.302 | 1.44E-05 | 9.14E-05 |
|  |  |  | 1 | 11766.43 | 14708.69 | 4641.90 | 168839.90 |  |  |  |
| 11103-24 | HSPB1 | P04792 | 0 | 15700.73 | 10140.02 | 1475.90 | 36575.00 | -0.734 | 1.46E-05 | 9.18E-05 |
|  |  |  | 1 | 11433.21 | 8789.83 | 515.20 | 38563.90 |  |  |  |
| 5456-59 | CNDP1 | Q96KN2 | 0 | 6975.58 | 2322.32 | 2304.90 | 13980.20 | -0.296 | 1.57E-05 | 9.83E-05 |
|  |  |  | 1 | 5941.05 | 4813.89 | 1663.10 | 80875.90 |  |  |  |
| 5029-3 | FAP | Q12884 | 0 | 941.47 | 200.59 | 548.30 | 1518.20 | -0.226 | 1.63E-05 | 1.02E-04 |
|  |  |  | 1 | 889.96 | 1428.16 | 369.90 | 25192.80 |  |  |  |
| 3585-54 | BSG | P35613 | 0 | 430.10 | 73.68 | 310.70 | 774.10 | 0.169 | 1.97E-05 | 1.22E-04 |
|  |  |  | 1 | 497.02 | 183.24 | 335.20 | 2300.30 |  |  |  |
| 2828-82 | SPINT1 | O43278 | 0 | 6655.27 | 2880.87 | 2705.30 | 28055.90 | -0.282 | 2.04E-05 | 1.26E-04 |
|  |  |  | 1 | 5539.96 | 2059.28 | 1299.50 | 13121.00 |  |  |  |
| 4908-6 | ENG | P17813 | 0 | 1654.19 | 469.91 | 362.90 | 2894.70 | -0.323 | 2.09E-05 | 1.28E-04 |
|  |  |  | 1 | 1388.12 | 563.43 | 153.80 | 4031.60 |  |  |  |
| 5260-80 | TYK2 | P29597 | 0 | 3412.48 | 1177.50 | 1590.10 | 6500.90 | -0.279 | 2.10E-05 | 1.29E-04 |
|  |  |  | 1 | 2890.41 | 1261.85 | 988.40 | 8595.00 |  |  |  |
| 4969-2 | CA1 | P00915 | 0 | 19005.93 | 9413.35 | 4536.50 | 60866.50 | 0.358 | 2.12E-05 | 1.29E-04 |
|  |  |  | 1 | 24729.41 | 14662.35 | 6149.00 | 127152.90 |  |  |  |
| 5099-14 | LAG3 | P18627 | 0 | 8932.17 | 3175.45 | 2739.10 | 20430.70 | -0.320 | 2.14E-05 | 1.30E-04 |
|  |  |  | 1 | 7377.83 | 3132.05 | 330.80 | 22999.60 |  |  |  |
| 4476-22 | ZAP70 | P43403 | 0 | 14143.82 | 3220.77 | 7242.50 | 24611.10 | -0.218 | 2.15E-05 | 1.30E-04 |
|  |  |  | 1 | 12603.96 | 7291.67 | 2750.60 | 124817.20 |  |  |  |
| 5465-32 | HS6ST1 | O60243 | 0 | 2950.87 | 520.56 | 1850.70 | 4289.80 | -0.146 | 2.17E-05 | 1.30E-04 |
|  |  |  | 1 | 2685.01 | 568.97 | 817.80 | 5691.20 |  |  |  |
| 3605-77 | MASP1 | P48740 | 0 | 5399.45 | 960.28 | 3481.70 | 9863.50 | -0.147 | 2.26E-05 | 1.35E-04 |
|  |  |  | 1 | 4912.91 | 1024.29 | 1838.10 | 8840.30 |  |  |  |
| 2937-10 | APOE | P02649 | 0 | 271544.33 | 49523.40 | 123393.20 | 381552.20 | -0.175 | 2.41E-05 | 1.44E-04 |
|  |  |  | 1 | 243988.89 | 59611.14 | 73931.10 | 526945.90 |  |  |  |
| 3855-56 | PRDX1 | Q06830 | 0 | 2936.70 | 1444.59 | 944.20 | 8048.70 | 0.426 | 2.49E-05 | 1.48E-04 |
|  |  |  | 1 | 4544.54 | 6177.87 | 886.80 | 99413.30 |  |  |  |
| 9211-19 | SERPINF1 | P36955 | 0 | 61342.40 | 9411.10 | 38070.00 | 93159.50 | -0.112 | 2.52E-05 | 1.49E-04 |
|  |  |  | 1 | 56855.57 | 9687.72 | 36260.20 | 129292.70 |  |  |  |
| 3217-74 | SERPINE2 | P07093 | 0 | 3149.84 | 9858.05 | 1254.30 | 100322.60 | 0.310 | 2.63E-05 | 1.55E-04 |
|  |  |  | 1 | 2890.88 | 1611.81 | 1162.70 | 14859.70 |  |  |  |
| 4474-19 | RPS27A | P62979 | 0 | 1467.23 | 480.11 | 803.80 | 2706.60 | 0.306 | 2.72E-05 | 1.59E-04 |
|  |  |  | 1 | 2261.80 | 6931.97 | 797.90 | 120637.90 |  |  |  |
| 5463-22 | GAS1 | P54826 | 0 | 779.30 | 132.21 | 526.70 | 1367.40 | -0.136 | 2.77E-05 | 1.61E-04 |
|  |  |  | 1 | 714.79 | 162.01 | 429.30 | 1933.30 |  |  |  |
| 2515-14 | GFRA2 | O00451 | 0 | 6168.18 | 1216.82 | 3651.40 | 9622.60 | -0.144 | 2.93E-05 | 1.70E-04 |
|  |  |  | 1 | 5592.07 | 1164.37 | 3011.40 | 9485.90 |  |  |  |
| 5109-24 | NRCAM | Q92823 | 0 | 18190.14 | 4924.17 | 9159.30 | 34896.10 | -0.268 | 2.99E-05 | 1.73E-04 |
|  |  |  | 1 | 15712.42 | 5953.74 | 1290.20 | 34690.80 |  |  |  |
| 3077-66 | F10 | P00742 | 0 | 5347.51 | 1072.62 | 2109.30 | 7873.70 | -0.141 | 3.02E-05 | 1.74E-04 |
|  |  |  | 1 | 4803.84 | 797.92 | 1636.90 | 7304.90 |  |  |  |
| 3293-2 | CD5L | O43866 | 0 | 2266.84 | 1187.93 | 594.40 | 7620.00 | 0.370 | 3.25E-05 | 1.85E-04 |
|  |  |  | 1 | 3159.28 | 2983.77 | 927.50 | 37211.60 |  |  |  |
| 2645-54 | PRKCZ | Q05513 | 0 | 1030.10 | 390.59 | 602.40 | 2702.30 | 0.294 | 3.24E-05 | 1.85E-04 |
|  |  |  | 1 | 1367.00 | 1117.46 | 593.60 | 14048.80 |  |  |  |
| 2622-18 | HMOX2 | P30519 | 0 | 502.67 | 190.72 | 326.40 | 1563.10 | 0.214 | 3.26E-05 | 1.85E-04 |
|  |  |  | 1 | 591.96 | 252.43 | 339.50 | 2579.50 |  |  |  |
| 3296-92 | CNTN2 | Q02246 | 0 | 1197.01 | 366.58 | 525.20 | 2423.20 | -0.254 | 3.31E-05 | 1.87E-04 |
|  |  |  | 1 | 1029.50 | 388.22 | 312.30 | 2364.90 |  |  |  |
| 3612-6 | EPHB4 | P54760 | 0 | 3256.94 | 514.48 | 2483.50 | 5045.50 | 0.171 | 3.46E-05 | 1.95E-04 |
|  |  |  | 1 | 3782.87 | 1324.45 | 2222.70 | 12461.20 |  |  |  |
| 13126-52 | DSC2 | Q02487 | 0 | 3501.24 | 749.80 | 1872.70 | 5648.70 | -0.195 | 3.48E-05 | 1.95E-04 |
|  |  |  | 1 | 3116.93 | 929.27 | 443.90 | 6851.10 |  |  |  |
| 5404-53 | TNFRSF21 | O75509 | 0 | 9581.78 | 1855.79 | 5895.40 | 16714.10 | -0.157 | 3.58E-05 | 1.99E-04 |
|  |  |  | 1 | 8659.17 | 1890.90 | 1458.00 | 18473.70 |  |  |  |
| 2770-51 | CCL1 | P22362 | 0 | 3680.77 | 920.09 | 2167.80 | 8270.60 | -0.178 | 3.70E-05 | 2.06E-04 |
|  |  |  | 1 | 3292.82 | 997.30 | 1344.40 | 11654.70 |  |  |  |
| 4990-87 | GP1BA | P07359 | 0 | 1535.34 | 288.34 | 966.60 | 2611.20 | -0.170 | 4.09E-05 | 2.26E-04 |
|  |  |  | 1 | 1412.38 | 834.67 | 580.80 | 14375.00 |  |  |  |
| 4801-13 | LPO | P22079 | 0 | 1038.33 | 411.86 | 403.00 | 2841.20 | -0.294 | 4.13E-05 | 2.27E-04 |
|  |  |  | 1 | 887.56 | 563.53 | 255.50 | 7389.90 |  |  |  |
| 5803-24 | C3 | P01024 | 0 | 12838.15 | 3718.33 | 3495.40 | 25281.00 | -0.296 | 4.22E-05 | 2.31E-04 |
|  |  |  | 1 | 10810.34 | 3670.73 | 883.10 | 21397.30 |  |  |  |
| 3283-21 | TGFBI | Q15582 | 0 | 47663.51 | 9080.79 | 29589.50 | 79501.30 | -0.158 | 4.30E-05 | 2.35E-04 |
|  |  |  | 1 | 43166.29 | 10156.58 | 14269.00 | 78195.10 |  |  |  |
| 9177-6 | FAM3B | P58499 | 0 | 1382.67 | 365.21 | 626.60 | 2276.50 | -0.210 | 4.40E-05 | 2.40E-04 |
|  |  |  | 1 | 1209.91 | 398.33 | 292.30 | 3490.60 |  |  |  |
| 2890-59 | CCL28 | Q9NRJ3 | 0 | 8718.07 | 3499.46 | 2896.10 | 24627.10 | 0.297 | 4.47E-05 | 2.42E-04 |
|  |  |  | 1 | 11195.31 | 6409.48 | 1850.20 | 56676.70 |  |  |  |
| 4811-33 | ITIH4 | Q14624 | 0 | 41182.40 | 6116.38 | 13895.30 | 53329.30 | 0.098 | 4.86E-05 | 2.62E-04 |
|  |  |  | 1 | 43870.11 | 5757.57 | 27350.20 | 64428.50 |  |  |  |
| 2844-53 | TIE1 | P35590 | 0 | 9236.38 | 1996.86 | 5464.20 | 14725.90 | -0.180 | 4.96E-05 | 2.66E-04 |
|  |  |  | 1 | 8266.05 | 2242.21 | 1536.50 | 16217.40 |  |  |  |
| 14144-3 | HIST3H2A | Q7L7L0 | 0 | 886.28 | 630.30 | 426.20 | 6256.30 | 0.412 | 5.05E-05 | 2.70E-04 |
|  |  |  | 1 | 1501.66 | 2166.29 | 342.20 | 19455.20 |  |  |  |
| 2976-58 | DSG1 | Q02413 | 0 | 3030.81 | 1882.99 | 1910.30 | 15859.20 | -0.192 | 5.17E-05 | 2.75E-04 |
|  |  |  | 1 | 2642.62 | 1665.99 | 967.30 | 22261.50 |  |  |  |
| 13118-5 | SMOC1 | Q9H4F8 | 0 | 3990.26 | 1550.24 | 2692.40 | 17947.80 | -0.139 | 5.37E-05 | 2.85E-04 |
|  |  |  | 1 | 3580.91 | 819.70 | 2145.00 | 9792.80 |  |  |  |
| 4703-87 | LTA | P01374 | 0 | 496.53 | 97.05 | 363.30 | 941.30 | 0.204 | 5.58E-05 | 2.95E-04 |
|  |  |  | 1 | 616.08 | 526.56 | 367.80 | 7357.70 |  |  |  |
| 3448-13 | INSR | P06213 | 0 | 10641.02 | 3248.30 | 4930.20 | 20027.80 | -0.249 | 5.72E-05 | 3.01E-04 |
|  |  |  | 1 | 9161.62 | 3354.06 | 1773.10 | 25332.60 |  |  |  |
| 2636-10 | LTBR | P36941 | 0 | 6845.09 | 1402.02 | 3483.40 | 11964.20 | -0.203 | 5.88E-05 | 3.08E-04 |
|  |  |  | 1 | 6200.04 | 3241.45 | 1713.90 | 46105.00 |  |  |  |
| 3521-16 | CGA TSHB | P01215 P01222 | 0 | 877.12 | 514.63 | 277.20 | 4617.60 | -0.310 | 6.20E-05 | 3.24E-04 |
|  |  |  | 1 | 712.83 | 413.34 | 176.40 | 4718.70 |  |  |  |
| 3067-67 | GDF9 | O60383 | 0 | 520.54 | 295.90 | 292.90 | 3059.50 | 0.339 | 6.44E-05 | 3.35E-04 |
|  |  |  | 1 | 953.54 | 4074.43 | 339.40 | 69974.80 |  |  |  |
| 3823-9 | MATK | P42679 | 0 | 424.52 | 112.29 | 304.00 | 884.20 | 0.187 | 7.04E-05 | 3.63E-04 |
|  |  |  | 1 | 500.55 | 297.30 | 297.10 | 4541.20 |  |  |  |
| 3466-8 | PRKACA | P17612 | 0 | 1570.88 | 1286.27 | 142.50 | 9253.50 | -0.590 | 7.02E-05 | 3.63E-04 |
|  |  |  | 1 | 1183.65 | 1030.31 | 113.80 | 6207.40 |  |  |  |
| 13098-93 | FIGF | O43915 | 0 | 611.37 | 144.34 | 350.20 | 1215.20 | -0.181 | 7.11E-05 | 3.65E-04 |
|  |  |  | 1 | 549.36 | 181.66 | 263.90 | 1850.20 |  |  |  |
| 3191-50 | WFIKKN1 | Q96NZ8 | 0 | 1191.62 | 392.11 | 410.60 | 2415.70 | -0.249 | 7.29E-05 | 3.73E-04 |
|  |  |  | 1 | 1023.44 | 429.10 | 171.20 | 4057.30 |  |  |  |
| 3685-53 | MFRP | Q9BY79 | 0 | 2554.83 | 917.63 | 1339.70 | 5097.20 | 0.275 | 7.37E-05 | 3.75E-04 |
|  |  |  | 1 | 3281.54 | 2272.02 | 1243.40 | 20436.50 |  |  |  |
| 4267-81 | PES1 | O00541 | 0 | 280.62 | 97.23 | 191.60 | 706.90 | 0.253 | 7.40E-05 | 3.75E-04 |
|  |  |  | 1 | 366.25 | 387.88 | 205.10 | 5564.60 |  |  |  |
| 5106-52 | NOTCH2 | Q04721 | 0 | 1144.71 | 1070.39 | 432.20 | 11118.30 | -0.342 | 7.38E-05 | 3.75E-04 |
|  |  |  | 1 | 1087.45 | 2418.63 | 223.50 | 34540.70 |  |  |  |
| 2468-62 | CCL20 | P78556 | 0 | 195.94 | 130.40 | 126.50 | 1389.30 | 0.229 | 7.65E-05 | 3.85E-04 |
|  |  |  | 1 | 235.23 | 170.33 | 125.20 | 1803.90 |  |  |  |
| 2871-73 | RAD51 | Q06609 | 0 | 2723.93 | 1972.08 | 1393.70 | 21685.00 | -0.219 | 8.21E-05 | 4.12E-04 |
|  |  |  | 1 | 2392.64 | 2258.12 | 864.50 | 38433.00 |  |  |  |
| 5275-28 | VAV1 | P15498 | 0 | 13062.09 | 12548.85 | 1483.20 | 49786.40 | 0.687 | 8.31E-05 | 4.15E-04 |
|  |  |  | 1 | 23337.88 | 20756.55 | 1356.10 | 103490.70 |  |  |  |
| 4673-13 | IL6 | P05231 | 0 | 642.72 | 293.63 | 271.50 | 1626.50 | 0.316 | 8.46E-05 | 4.22E-04 |
|  |  |  | 1 | 887.49 | 1523.14 | 303.90 | 26163.70 |  |  |  |
| 5009-11 | MSN | P26038 | 0 | 1014.35 | 413.73 | 474.90 | 3037.50 | 0.321 | 8.76E-05 | 4.34E-04 |
|  |  |  | 1 | 1429.27 | 1341.09 | 571.20 | 14595.90 |  |  |  |
| 3587-53 | BMP10 | O95393 | 0 | 2943.54 | 12028.51 | 888.20 | 121945.80 | -0.243 | 9.11E-05 | 4.50E-04 |
|  |  |  | 1 | 1584.20 | 703.18 | 662.90 | 7765.30 |  |  |  |
| 4983-6 | ERP29 | P30040 | 0 | 9797.28 | 2212.31 | 4813.10 | 18072.80 | -0.161 | 9.44E-05 | 4.65E-04 |
|  |  |  | 1 | 8829.97 | 2382.24 | 4507.60 | 23018.20 |  |  |  |
| 3477-63 | NTRK1 | P04629 | 0 | 3599.50 | 1728.17 | 2201.60 | 18071.40 | 0.250 | 9.53E-05 | 4.67E-04 |
|  |  |  | 1 | 4558.41 | 3687.43 | 1938.50 | 49731.00 |  |  |  |
| 3620-67 | IL22RA1 | Q8N6P7 | 0 | 962.56 | 568.74 | 602.50 | 5467.60 | 0.225 | 9.70E-05 | 4.73E-04 |
|  |  |  | 1 | 1155.11 | 834.06 | 468.50 | 10610.30 |  |  |  |
| 4437-56 | ENTPD5 | O75356 | 0 | 4914.58 | 1200.68 | 2621.20 | 8966.90 | -0.145 | 9.68E-05 | 4.73E-04 |
|  |  |  | 1 | 4413.29 | 941.49 | 2258.60 | 7171.60 |  |  |  |
| 3038-9 | CXCL11 | O14625 | 0 | 4062.16 | 2952.71 | 575.20 | 17136.00 | 0.409 | 1.02E-04 | 4.94E-04 |
|  |  |  | 1 | 5498.46 | 4733.68 | 949.70 | 42850.60 |  |  |  |
| 4907-56 | FGA FGB FGG | P02671 P02675 P02679 | 0 | 13889.37 | 2899.12 | 8824.00 | 23974.20 | 0.230 | 1.02E-04 | 4.94E-04 |
|  |  |  | 1 | 16542.30 | 2989.34 | 171.80 | 26778.30 |  |  |  |
| 4708-3 | HSD17B1 | P14061 | 0 | 2192.83 | 566.43 | 1676.50 | 6143.00 | 0.252 | 1.03E-04 | 4.98E-04 |
|  |  |  | 1 | 2883.77 | 1986.12 | 996.70 | 16804.70 |  |  |  |
| 2822-56 | CD97 | P48960 | 0 | 1928.58 | 349.26 | 1155.30 | 3163.60 | -0.143 | 1.04E-04 | 4.99E-04 |
|  |  |  | 1 | 1766.82 | 464.28 | 697.80 | 5819.60 |  |  |  |
| 2742-68 | SIGLEC7 | Q9Y286 | 0 | 1593.06 | 374.39 | 913.20 | 3705.50 | -0.149 | 1.10E-04 | 5.26E-04 |
|  |  |  | 1 | 1440.37 | 344.62 | 636.40 | 3501.10 |  |  |  |
| 5008-51 | SOD2 | P04179 | 0 | 49808.79 | 12616.25 | 31923.90 | 106775.80 | -0.163 | 1.13E-04 | 5.38E-04 |
|  |  |  | 1 | 44789.65 | 12710.75 | 25110.00 | 104108.40 |  |  |  |
| 3367-8 | FETUB | Q9UGM5 | 0 | 6880.47 | 1528.03 | 3744.80 | 13876.40 | -0.216 | 1.14E-04 | 5.39E-04 |
|  |  |  | 1 | 6034.62 | 1438.99 | 50.80 | 12801.90 |  |  |  |
| 5080-131 | GPNMB | Q14956 | 0 | 6366.30 | 1301.19 | 3647.70 | 9789.40 | -0.169 | 1.14E-04 | 5.39E-04 |
|  |  |  | 1 | 5757.09 | 1612.51 | 860.30 | 15902.50 |  |  |  |
| 9196-8 | LGALS7 | P47929 | 0 | 785.31 | 447.44 | 501.20 | 4792.90 | 0.210 | 1.16E-04 | 5.45E-04 |
|  |  |  | 1 | 930.55 | 834.35 | 463.60 | 14306.20 |  |  |  |
| 2783-18 | CCL3L1 | P16619 | 0 | 1985.22 | 436.68 | 1105.60 | 3497.60 | 0.183 | 1.16E-04 | 5.45E-04 |
|  |  |  | 1 | 2309.30 | 798.07 | 884.70 | 8521.40 |  |  |  |
| 3656-9 | CDH12 | P55289 | 0 | 969.44 | 387.07 | 548.10 | 2234.50 | 0.262 | 1.22E-04 | 5.70E-04 |
|  |  |  | 1 | 1249.21 | 1380.79 | 554.60 | 21809.70 |  |  |  |
| 3303-23 | CST6 | Q15828 | 0 | 9302.38 | 3061.24 | 3815.70 | 20196.60 | -0.246 | 1.24E-04 | 5.79E-04 |
|  |  |  | 1 | 8048.06 | 3418.09 | 1122.70 | 32557.30 |  |  |  |
| 2333-72 | TGFB1 | P01137 | 0 | 2206.00 | 2424.85 | 1244.60 | 24691.40 | 0.230 | 1.30E-04 | 6.03E-04 |
|  |  |  | 1 | 2369.56 | 861.60 | 1096.00 | 6407.60 |  |  |  |
| 7628-40 | CRELD1 | Q96HD1 | 0 | 3915.02 | 1044.90 | 1845.40 | 8132.60 | -0.189 | 1.37E-04 | 6.35E-04 |
|  |  |  | 1 | 3462.70 | 1017.08 | 1047.40 | 7988.80 |  |  |  |
| 5105-2 | RTN4R | Q9BZR6 | 0 | 2200.52 | 682.54 | 931.00 | 4370.00 | -0.211 | 1.40E-04 | 6.44E-04 |
|  |  |  | 1 | 1917.67 | 623.35 | 325.60 | 4588.70 |  |  |  |
| 3041-55 | MRC2 | Q9UBG0 | 0 | 4306.13 | 882.59 | 2508.60 | 6863.00 | -0.157 | 1.44E-04 | 6.62E-04 |
|  |  |  | 1 | 3904.38 | 961.49 | 993.90 | 7544.50 |  |  |  |
| 3196-6 | HAPLN1 | P10915 | 0 | 1756.78 | 840.10 | 883.80 | 7429.30 | -0.242 | 1.49E-04 | 6.83E-04 |
|  |  |  | 1 | 1564.47 | 1518.70 | 722.10 | 19522.00 |  |  |  |
| 5357-60 | NLGN4X | Q8N0W4 | 0 | 1051.10 | 1311.24 | 518.70 | 13490.50 | -0.236 | 1.64E-04 | 7.48E-04 |
|  |  |  | 1 | 843.97 | 758.59 | 326.60 | 10635.20 |  |  |  |
| 3216-2 | PIGR | P01833 | 0 | 4017.75 | 1689.91 | 1530.40 | 11740.30 | 0.270 | 1.65E-04 | 7.49E-04 |
|  |  |  | 1 | 4982.42 | 2406.11 | 1082.30 | 16638.50 |  |  |  |
| 2994-71 | IL1RL2 | Q9HB29 | 0 | 957.79 | 155.71 | 619.50 | 1593.70 | -0.140 | 1.65E-04 | 7.49E-04 |
|  |  |  | 1 | 890.04 | 341.06 | 454.50 | 4915.20 |  |  |  |
| 7648-9 | MYBPC1 | Q00872 | 0 | 4725.47 | 5864.46 | 841.10 | 40763.40 | -0.451 | 1.71E-04 | 7.72E-04 |
|  |  |  | 1 | 3256.82 | 4031.18 | 439.00 | 52701.10 |  |  |  |
| 3212-30 | ASAH2 | Q9NR71 | 0 | 5034.70 | 2180.07 | 1761.00 | 12324.00 | -0.282 | 1.73E-04 | 7.79E-04 |
|  |  |  | 1 | 4158.02 | 1793.36 | 593.30 | 10889.70 |  |  |  |
| 3758-68 | PROC | P04070 | 0 | 897.83 | 248.77 | 576.00 | 2173.20 | 0.214 | 1.77E-04 | 7.92E-04 |
|  |  |  | 1 | 1149.49 | 1470.38 | 559.20 | 23398.10 |  |  |  |
| 2846-24 | RPS27A | P62979 | 0 | 3828.93 | 1999.11 | 1587.00 | 8891.00 | 0.382 | 1.83E-04 | 8.16E-04 |
|  |  |  | 1 | 5580.94 | 4785.07 | 1369.60 | 37412.20 |  |  |  |
| 4469-78 | CHST15 | Q7LFX5 | 0 | 5969.99 | 1174.21 | 3567.40 | 9935.90 | -0.143 | 1.83E-04 | 8.16E-04 |
|  |  |  | 1 | 5443.85 | 1191.91 | 1063.80 | 8683.90 |  |  |  |
| 3198-4 | IDS | P22304 | 0 | 3091.63 | 1013.21 | 776.10 | 7827.70 | -0.265 | 1.84E-04 | 8.16E-04 |
|  |  |  | 1 | 2776.94 | 2962.63 | 509.50 | 50401.00 |  |  |  |
| 3186-2 | C2 | P06681 | 0 | 3274.86 | 412.36 | 1409.30 | 4289.80 | -0.097 | 1.88E-04 | 8.30E-04 |
|  |  |  | 1 | 3072.23 | 490.80 | 1388.40 | 6842.10 |  |  |  |
| 3025-50 | FGF2 | P09038 | 0 | 3029.00 | 4157.58 | 752.50 | 34126.20 | 0.530 | 2.00E-04 | 8.83E-04 |
|  |  |  | 1 | 5602.92 | 13178.47 | 671.90 | 164234.30 |  |  |  |
| 2693-20 | OSM | P13725 | 0 | 2220.54 | 443.06 | 1448.70 | 4369.30 | -0.135 | 2.01E-04 | 8.84E-04 |
|  |  |  | 1 | 2052.03 | 698.31 | 1084.70 | 9195.80 |  |  |  |
| 3461-58 | BCAN | Q96GW7 | 0 | 1759.01 | 1364.07 | 910.30 | 14585.80 | -0.191 | 2.30E-04 | 1.01E-03 |
|  |  |  | 1 | 1493.96 | 667.83 | 528.70 | 8682.20 |  |  |  |
| 11089-7 | IGHA1 IGHA2 | P01876 P01877 | 0 | 4888.80 | 2253.92 | 137.60 | 13708.00 | 0.306 | 2.38E-04 | 1.04E-03 |
|  |  |  | 1 | 6233.97 | 6795.45 | 900.60 | 114685.60 |  |  |  |
| 3083-71 | EDA2R | Q9HAV5 | 0 | 1048.05 | 627.58 | 672.50 | 5317.00 | 0.281 | 2.39E-04 | 1.04E-03 |
|  |  |  | 1 | 1617.43 | 4477.52 | 682.40 | 70500.00 |  |  |  |
| 3481-87 | XPNPEP1 | Q9NQW7 | 0 | 7504.81 | 4914.81 | 680.70 | 25074.80 | -0.471 | 2.80E-04 | 1.22E-03 |
|  |  |  | 1 | 5873.53 | 4210.25 | 256.00 | 31181.30 |  |  |  |
| 3281-19 | ANGPTL3 | Q9Y5C1 | 0 | 446.26 | 93.35 | 345.80 | 1101.50 | 0.175 | 2.88E-04 | 1.24E-03 |
|  |  |  | 1 | 558.17 | 883.62 | 341.50 | 15517.20 |  |  |  |
| 13122-19 | FLRT2 | O43155 | 0 | 22214.85 | 5326.49 | 550.60 | 34633.00 | -0.227 | 2.91E-04 | 1.25E-03 |
|  |  |  | 1 | 19002.50 | 5835.82 | 1179.80 | 47085.60 |  |  |  |
| 4133-54 | GZMB | P10144 | 0 | 247.74 | 59.49 | 181.80 | 560.20 | 0.214 | 2.97E-04 | 1.27E-03 |
|  |  |  | 1 | 313.62 | 228.77 | 152.80 | 2533.70 |  |  |  |
| 4496-60 | MMP12 | P39900 | 0 | 1870.50 | 872.36 | 611.50 | 5170.70 | 0.343 | 3.04E-04 | 1.30E-03 |
|  |  |  | 1 | 2582.46 | 1737.46 | 268.40 | 15384.30 |  |  |  |
| 5111-15 | NRXN3 | Q9HDB5 | 0 | 1084.58 | 231.73 | 678.10 | 2113.30 | -0.158 | 3.05E-04 | 1.30E-03 |
|  |  |  | 1 | 992.04 | 335.11 | 413.50 | 4361.50 |  |  |  |
| 3459-49 | PDGFRB | P09619 | 0 | 6306.08 | 2874.55 | 776.00 | 12589.80 | -0.342 | 3.07E-04 | 1.31E-03 |
|  |  |  | 1 | 4978.57 | 2332.30 | 490.50 | 12916.10 |  |  |  |
| 3647-49 | TLR4 LY96 | O00206 Q9Y6Y9 | 0 | 5509.04 | 1399.07 | 2623.90 | 10891.00 | -0.199 | 3.20E-04 | 1.36E-03 |
|  |  |  | 1 | 5195.06 | 6542.35 | 1676.40 | 110482.80 |  |  |  |
| 9202-309 | ATP5O | P48047 | 0 | 12142.84 | 13778.15 | 1628.70 | 115701.50 | -0.513 | 3.31E-04 | 1.40E-03 |
|  |  |  | 1 | 8290.86 | 6533.80 | 626.90 | 44858.40 |  |  |  |
| 3666-17 | CFHR5 | Q9BXR6 | 0 | 1289.30 | 304.19 | 686.40 | 2371.20 | 0.195 | 3.37E-04 | 1.42E-03 |
|  |  |  | 1 | 1587.87 | 1795.78 | 638.40 | 31578.10 |  |  |  |
| 4455-89 | MFGE8 | Q08431 | 0 | 1340.94 | 1532.17 | 426.50 | 15549.60 | -0.339 | 3.64E-04 | 1.53E-03 |
|  |  |  | 1 | 1047.17 | 706.63 | 92.10 | 5443.50 |  |  |  |
| 2644-11 | PRKCA | P17252 | 0 | 71767.59 | 45902.03 | 3310.50 | 163954.30 | -0.695 | 3.68E-04 | 1.54E-03 |
|  |  |  | 1 | 59794.00 | 47156.07 | 1751.60 | 205074.90 |  |  |  |
| 2524-56 | HMGB1 | P09429 | 0 | 6970.33 | 2445.43 | 4493.30 | 24589.90 | 0.278 | 3.72E-04 | 1.55E-03 |
|  |  |  | 1 | 9788.66 | 9174.67 | 2564.50 | 66201.30 |  |  |  |
| 2624-31 | Human-virus | P06788 | 0 | 2288.71 | 588.65 | 1464.80 | 5740.10 | -0.139 | 3.95E-04 | 1.64E-03 |
|  |  |  | 1 | 2110.99 | 949.92 | 1188.40 | 15301.20 |  |  |  |
| 5201-50 | PDE9A | O76083 | 0 | 653.46 | 656.03 | 302.80 | 6810.70 | -0.263 | 4.02E-04 | 1.67E-03 |
|  |  |  | 1 | 546.29 | 466.91 | 159.90 | 5442.80 |  |  |  |
| 2431-17 | CTSG | P08311 | 0 | 1173.78 | 861.66 | 705.40 | 8431.00 | 0.238 | 4.15E-04 | 1.71E-03 |
|  |  |  | 1 | 1410.11 | 959.18 | 735.90 | 9590.60 |  |  |  |
| 2876-74 | TOP1 | P11387 | 0 | 731.98 | 481.43 | 452.60 | 4966.90 | 0.352 | 4.22E-04 | 1.74E-03 |
|  |  |  | 1 | 1248.49 | 1863.80 | 237.70 | 13654.00 |  |  |  |
| 8476-11 | CHGA | P10645 | 0 | 12170.05 | 11301.35 | 335.40 | 74123.80 | 0.432 | 4.35E-04 | 1.79E-03 |
|  |  |  | 1 | 16887.89 | 13904.97 | 2074.80 | 103134.70 |  |  |  |
| 4237-70 | LCMT1 | Q9UIC8 | 0 | 1673.98 | 324.49 | 1080.00 | 3013.90 | 0.153 | 4.37E-04 | 1.79E-03 |
|  |  |  | 1 | 1909.37 | 641.68 | 972.20 | 7532.80 |  |  |  |
| 14122-132 | ZNRF3 | Q9ULT6 | 0 | 779.75 | 1903.78 | 282.70 | 19238.60 | -0.298 | 4.53E-04 | 1.85E-03 |
|  |  |  | 1 | 548.67 | 738.30 | 260.40 | 7947.00 |  |  |  |
| 5078-82 | EPHB6 | O15197 | 0 | 1759.11 | 329.74 | 1158.30 | 2593.70 | -0.128 | 4.61E-04 | 1.87E-03 |
|  |  |  | 1 | 1632.87 | 549.37 | 947.00 | 8176.40 |  |  |  |
| 2816-50 | BCAM | P50895 | 0 | 1971.76 | 811.84 | 1187.10 | 9168.70 | -0.156 | 4.67E-04 | 1.89E-03 |
|  |  |  | 1 | 1764.07 | 484.40 | 725.80 | 3979.80 |  |  |  |
| 2981-9 | ESAM | Q96AP7 | 0 | 4771.86 | 1009.23 | 2690.10 | 8234.40 | -0.132 | 4.76E-04 | 1.92E-03 |
|  |  |  | 1 | 4383.16 | 1079.29 | 2537.40 | 8259.90 |  |  |  |
| 14583-49 | MSTN | O14793 | 0 | 5836.34 | 2206.72 | 2339.00 | 12415.80 | -0.247 | 4.84E-04 | 1.95E-03 |
|  |  |  | 1 | 5059.35 | 2291.16 | 1392.80 | 15142.60 |  |  |  |
| 5032-64 | SSRP1 | Q08945 | 0 | 556.50 | 297.04 | 404.20 | 2669.40 | 0.254 | 4.90E-04 | 1.97E-03 |
|  |  |  | 1 | 779.40 | 1517.30 | 385.20 | 25513.30 |  |  |  |
| 5001-6 | SCGB2A1 | O75556 | 0 | 8588.72 | 1725.71 | 6231.80 | 13991.40 | 0.127 | 5.18E-04 | 2.07E-03 |
|  |  |  | 1 | 9484.63 | 2688.38 | 5697.00 | 32318.80 |  |  |  |
| 3607-71 | DKK3 | Q9UBP4 | 0 | 6482.50 | 1322.91 | 3458.30 | 10690.10 | -0.138 | 5.21E-04 | 2.07E-03 |
|  |  |  | 1 | 5952.44 | 1493.29 | 2447.40 | 11266.60 |  |  |  |
| 2942-50 | CYCS | P99999 | 0 | 2298.31 | 3348.85 | 1111.90 | 35232.70 | -0.183 | 5.20E-04 | 2.07E-03 |
|  |  |  | 1 | 1826.02 | 563.14 | 549.60 | 6344.30 |  |  |  |
| 3327-27 | NTN4 | Q9HB63 | 0 | 883.50 | 435.25 | 562.20 | 4972.40 | 0.127 | 5.52E-04 | 2.19E-03 |
|  |  |  | 1 | 945.36 | 214.77 | 561.90 | 1936.50 |  |  |  |
| 3624-3 | PRSS27 | Q9BQR3 | 0 | 8531.18 | 2273.54 | 5914.70 | 22699.10 | -0.131 | 5.54E-04 | 2.19E-03 |
|  |  |  | 1 | 7838.25 | 2178.21 | 3807.20 | 21177.90 |  |  |  |
| 3294-55 | CFC1 | P0CG37 | 0 | 2578.26 | 895.28 | 1735.60 | 8605.80 | 0.194 | 5.67E-04 | 2.24E-03 |
|  |  |  | 1 | 3074.02 | 1779.51 | 1371.60 | 22775.40 |  |  |  |
| 3890-8 | LDHB | P07195 | 0 | 1418.86 | 606.52 | 738.30 | 3229.80 | 0.282 | 5.79E-04 | 2.27E-03 |
|  |  |  | 1 | 2135.62 | 6536.75 | 477.80 | 113848.30 |  |  |  |
| 3197-70 | IDE | P14735 | 0 | 2943.48 | 1113.67 | 1446.20 | 6155.30 | -0.261 | 5.82E-04 | 2.28E-03 |
|  |  |  | 1 | 2758.70 | 4099.12 | 493.50 | 66762.80 |  |  |  |
| 3601-54 | CHL1 | O00533 | 0 | 13679.26 | 2992.14 | 8201.00 | 24608.80 | -0.149 | 5.99E-04 | 2.34E-03 |
|  |  |  | 1 | 12510.11 | 3423.97 | 2747.10 | 34622.40 |  |  |  |
| 2719-3 | CSF3R | Q99062 | 0 | 1028.52 | 423.55 | 728.30 | 4610.10 | 0.181 | 6.07E-04 | 2.37E-03 |
|  |  |  | 1 | 1219.88 | 953.52 | 627.10 | 14909.60 |  |  |  |
| 5096-51 | KIR3DL2 | P43630 | 0 | 468.70 | 416.84 | 256.80 | 4134.30 | 0.453 | 6.13E-04 | 2.37E-03 |
|  |  |  | 1 | 2099.91 | 15566.19 | 256.60 | 257842.00 |  |  |  |
| 4718-5 | PPIB | P23284 | 0 | 3435.83 | 1151.24 | 2438.60 | 12530.20 | 0.177 | 6.12E-04 | 2.37E-03 |
|  |  |  | 1 | 4080.70 | 2720.42 | 2219.30 | 32594.70 |  |  |  |
| 9207-60 | CRYZL1 | O95825 | 0 | 3245.12 | 1448.61 | 1174.90 | 10206.80 | -0.240 | 6.20E-04 | 2.39E-03 |
|  |  |  | 1 | 2810.42 | 1953.00 | 794.70 | 28455.40 |  |  |  |
| 2763-66 | FGF20 | Q9NP95 | 0 | 434.48 | 125.49 | 267.00 | 1210.20 | -0.151 | 6.37E-04 | 2.45E-03 |
|  |  |  | 1 | 396.46 | 166.72 | 181.00 | 2375.00 |  |  |  |
| 13117-232 | CHKB | Q9Y259 | 0 | 4160.30 | 1348.39 | 2253.30 | 9887.10 | -0.217 | 6.48E-04 | 2.49E-03 |
|  |  |  | 1 | 3826.33 | 2999.41 | 1152.40 | 35555.90 |  |  |  |
| 2692-74 | PLA2G2A | P14555 | 0 | 3571.98 | 1861.61 | 1731.10 | 16144.10 | 0.284 | 6.57E-04 | 2.51E-03 |
|  |  |  | 1 | 4771.28 | 4589.69 | 1169.20 | 58795.10 |  |  |  |
| 11510-31 | APOL1 | O14791 | 0 | 39506.43 | 8218.31 | 16192.70 | 64683.60 | -0.120 | 6.65E-04 | 2.54E-03 |
|  |  |  | 1 | 36294.66 | 7362.26 | 17230.30 | 64305.60 |  |  |  |
| 9212-22 | CTSF | Q9UBX1 | 0 | 3491.86 | 891.88 | 1706.90 | 5991.90 | -0.162 | 6.72E-04 | 2.56E-03 |
|  |  |  | 1 | 3180.56 | 1469.11 | 1467.60 | 23533.60 |  |  |  |
| 3365-7 | DKK4 | Q9UBT3 | 0 | 7488.85 | 2142.30 | 3962.60 | 12863.60 | 0.197 | 6.97E-04 | 2.64E-03 |
|  |  |  | 1 | 8801.22 | 3166.59 | 3128.00 | 21896.40 |  |  |  |
| 5087-5 | IL22RA2 | Q969J5 | 0 | 2452.05 | 756.66 | 954.20 | 5515.90 | -0.260 | 7.05E-04 | 2.67E-03 |
|  |  |  | 1 | 2202.55 | 1222.61 | 372.70 | 16362.70 |  |  |  |
| 7625-27 | YWHAQ | P27348 | 0 | 1273.10 | 495.91 | 858.90 | 5207.80 | 0.176 | 7.20E-04 | 2.72E-03 |
|  |  |  | 1 | 1463.08 | 586.78 | 786.40 | 5523.50 |  |  |  |
| 13104-32 | EFNB1 | P98172 | 0 | 2021.83 | 478.20 | 1359.10 | 3799.80 | -0.120 | 7.78E-04 | 2.93E-03 |
|  |  |  | 1 | 1857.40 | 420.84 | 1212.30 | 3361.60 |  |  |  |
| 2705-5 | CCL25 | O15444 | 0 | 2510.99 | 1312.15 | 238.40 | 8266.30 | -0.313 | 7.90E-04 | 2.96E-03 |
|  |  |  | 1 | 2037.42 | 1286.33 | 207.70 | 15104.20 |  |  |  |
| 3351-1 | CAMK2B | Q13554 | 0 | 2333.82 | 1981.00 | 751.00 | 10354.80 | 0.411 | 8.02E-04 | 3.00E-03 |
|  |  |  | 1 | 3301.78 | 3013.40 | 654.30 | 22291.60 |  |  |  |
| 4763-31 | AFM | P43652 | 0 | 35381.67 | 6474.81 | 17832.00 | 53112.80 | -0.116 | 8.23E-04 | 3.07E-03 |
|  |  |  | 1 | 32818.67 | 6702.46 | 14894.50 | 52186.20 |  |  |  |
| 3221-54 | SFRP1 | Q8N474 | 0 | 6183.28 | 1676.51 | 1607.00 | 11210.80 | -0.249 | 8.35E-04 | 3.10E-03 |
|  |  |  | 1 | 5638.05 | 4774.88 | 686.10 | 79983.60 |  |  |  |
| 2765-4 | GDF11 MSTN | O95390 O14793 | 0 | 1037.91 | 259.37 | 519.80 | 1991.10 | -0.160 | 9.18E-04 | 3.40E-03 |
|  |  |  | 1 | 963.59 | 696.60 | 440.50 | 12185.60 |  |  |  |
| 2700-56 | PROS1 | P07225 | 0 | 5391.33 | 838.92 | 2895.40 | 6979.50 | -0.087 | 9.81E-04 | 3.63E-03 |
|  |  |  | 1 | 5054.41 | 682.33 | 2254.80 | 7420.20 |  |  |  |
| 5112-73 | CD200 | P41217 | 0 | 2880.13 | 833.68 | 1751.80 | 9060.80 | -0.179 | 1.06E-03 | 3.92E-03 |
|  |  |  | 1 | 2989.30 | 7715.52 | 1399.80 | 135149.50 |  |  |  |
| 3471-49 | STK16 | O75716 | 0 | 2200.10 | 1405.59 | 1497.00 | 15470.00 | -0.143 | 1.07E-03 | 3.94E-03 |
|  |  |  | 1 | 1956.12 | 739.93 | 858.60 | 8598.10 |  |  |  |
| 4143-74 | XCL1 | P47992 | 0 | 493.67 | 81.75 | 359.70 | 826.40 | 0.145 | 1.08E-03 | 3.96E-03 |
|  |  |  | 1 | 580.89 | 492.07 | 352.10 | 8126.00 |  |  |  |
| 3484-60 | AGT | P01019 | 0 | 5862.20 | 1607.83 | 1708.40 | 15932.50 | -0.162 | 1.11E-03 | 4.06E-03 |
|  |  |  | 1 | 5277.76 | 1428.38 | 327.00 | 14156.50 |  |  |  |
| 5424-55 | TNFRSF11A | Q9Y6Q6 | 0 | 1464.39 | 304.86 | 985.70 | 2779.20 | 0.155 | 1.14E-03 | 4.15E-03 |
|  |  |  | 1 | 1721.77 | 1153.54 | 563.70 | 15536.20 |  |  |  |
| 4154-57 | SELP | P16109 | 0 | 22875.70 | 6954.67 | 11752.40 | 45750.50 | 0.169 | 1.14E-03 | 4.16E-03 |
|  |  |  | 1 | 25928.90 | 8280.54 | 9643.30 | 59311.30 |  |  |  |
| 4991-12 | GPC5 | P78333 | 0 | 1069.34 | 560.56 | 522.40 | 5401.70 | -0.186 | 1.15E-03 | 4.17E-03 |
|  |  |  | 1 | 945.31 | 616.55 | 393.60 | 9425.40 |  |  |  |
| 2858-29 | HAT1 | O14929 | 0 | 423.71 | 262.56 | 197.50 | 2439.00 | 0.269 | 1.19E-03 | 4.29E-03 |
|  |  |  | 1 | 580.69 | 870.49 | 212.70 | 11916.10 |  |  |  |
| 3616-3 | GNS | P15586 | 0 | 3077.83 | 1139.58 | 1399.60 | 8127.00 | -0.189 | 1.19E-03 | 4.30E-03 |
|  |  |  | 1 | 2753.18 | 1472.25 | 864.60 | 22136.50 |  |  |  |
| 5092-51 | JAG1 | P78504 | 0 | 630.99 | 101.48 | 421.60 | 934.60 | -0.111 | 1.23E-03 | 4.43E-03 |
|  |  |  | 1 | 592.78 | 172.96 | 358.70 | 2361.40 |  |  |  |
| 3642-4 | CD84 | Q9UIB8 | 0 | 7923.58 | 2123.56 | 2688.50 | 17604.00 | -0.198 | 1.25E-03 | 4.48E-03 |
|  |  |  | 1 | 7122.60 | 2453.14 | 1426.30 | 20661.30 |  |  |  |
| 3802-50 | CST4 | P01036 | 0 | 160.77 | 76.18 | 103.30 | 710.60 | 0.252 | 1.26E-03 | 4.49E-03 |
|  |  |  | 1 | 227.90 | 348.87 | 94.80 | 4475.40 |  |  |  |
| 4157-2 | F2 | P00734 | 0 | 1426.63 | 2963.65 | 314.20 | 28592.10 | -0.348 | 1.27E-03 | 4.52E-03 |
|  |  |  | 1 | 1331.75 | 5438.19 | 268.50 | 80941.80 |  |  |  |
| 3640-14 | LRPAP1 | P30533 | 0 | 858.30 | 269.95 | 592.30 | 2668.80 | 0.175 | 1.28E-03 | 4.54E-03 |
|  |  |  | 1 | 1011.44 | 559.45 | 471.80 | 6742.60 |  |  |  |
| 9187-2 | HMGN1 | P05114 | 0 | 861.16 | 280.36 | 447.00 | 2926.50 | 0.239 | 1.34E-03 | 4.77E-03 |
|  |  |  | 1 | 1180.48 | 1229.66 | 471.10 | 11686.30 |  |  |  |
| 3329-14 | PGLYRP1 | O75594 | 0 | 773.24 | 668.90 | 254.90 | 4642.00 | 0.309 | 1.37E-03 | 4.83E-03 |
|  |  |  | 1 | 1043.66 | 1915.58 | 293.60 | 29598.40 |  |  |  |
| 3488-64 | CAT | P04040 | 0 | 17821.05 | 7414.06 | 6259.50 | 63694.70 | 0.197 | 1.40E-03 | 4.94E-03 |
|  |  |  | 1 | 20892.86 | 10464.22 | 7436.80 | 107851.00 |  |  |  |
| 4184-43 | EIF4EBP2 | Q13542 | 0 | 375.05 | 503.84 | 203.20 | 5191.60 | 0.191 | 1.43E-03 | 5.02E-03 |
|  |  |  | 1 | 393.64 | 304.04 | 205.60 | 5180.80 |  |  |  |
| 4890-10 | POMC | P01189 | 0 | 484.87 | 150.50 | 320.50 | 1490.20 | 0.158 | 1.45E-03 | 5.09E-03 |
|  |  |  | 1 | 562.06 | 352.01 | 327.60 | 5544.90 |  |  |  |
| 5268-49 | MMP16 | P51512 | 0 | 792.40 | 152.11 | 596.10 | 1369.20 | 0.157 | 1.48E-03 | 5.19E-03 |
|  |  |  | 1 | 935.00 | 528.69 | 559.00 | 6438.40 |  |  |  |
| 5493-17 | WNK3 | Q9BYP7 | 0 | 1430.38 | 889.00 | 586.10 | 4822.90 | 0.322 | 1.49E-03 | 5.19E-03 |
|  |  |  | 1 | 2099.35 | 4464.55 | 450.40 | 75997.90 |  |  |  |
| 14153-8 | EFNA3 | P52797 | 0 | 2332.22 | 545.96 | 1187.80 | 3562.20 | -0.164 | 1.51E-03 | 5.25E-03 |
|  |  |  | 1 | 2144.96 | 861.49 | 560.80 | 9737.70 |  |  |  |
| 7638-30 | LMAN2 | Q12907 | 0 | 14230.92 | 2847.11 | 9195.10 | 20683.70 | -0.139 | 1.58E-03 | 5.49E-03 |
|  |  |  | 1 | 13459.98 | 8750.96 | 4542.30 | 124340.80 |  |  |  |
| 9216-100 | PLXNB2 | O15031 | 0 | 5900.16 | 1211.88 | 3515.20 | 10943.10 | -0.123 | 1.69E-03 | 5.87E-03 |
|  |  |  | 1 | 5467.52 | 1333.53 | 1201.80 | 11898.80 |  |  |  |
| 4163-5 | H2AFZ | P0C0S5 | 0 | 1917.81 | 1091.60 | 612.10 | 7771.50 | 0.331 | 1.76E-03 | 6.06E-03 |
|  |  |  | 1 | 2895.82 | 3758.29 | 514.90 | 48472.30 |  |  |  |
| 3446-7 | IL18R1 | Q13478 | 0 | 13264.60 | 3592.69 | 6530.70 | 23207.60 | -0.183 | 1.78E-03 | 6.14E-03 |
|  |  |  | 1 | 12029.91 | 4321.11 | 1314.70 | 31718.40 |  |  |  |
| 4413-3 | SLPI | P03973 | 0 | 37569.19 | 7941.97 | 11139.70 | 61654.50 | 0.139 | 1.79E-03 | 6.15E-03 |
|  |  |  | 1 | 41736.68 | 10259.49 | 5916.50 | 74206.40 |  |  |  |
| 4906-35 | F5 | P12259 | 0 | 82781.33 | 13045.90 | 33456.50 | 115076.80 | -0.124 | 1.88E-03 | 6.42E-03 |
|  |  |  | 1 | 76995.95 | 15964.52 | 12639.50 | 129684.80 |  |  |  |
| 3506-49 | LTA LTB | P01374 Q06643 | 0 | 2604.59 | 545.13 | 1464.40 | 3867.60 | -0.128 | 1.88E-03 | 6.42E-03 |
|  |  |  | 1 | 2411.05 | 644.82 | 1009.90 | 5764.30 |  |  |  |
| 4923-79 | AMH | P03971 | 0 | 10137.88 | 1486.99 | 6924.00 | 14140.30 | -0.086 | 1.89E-03 | 6.44E-03 |
|  |  |  | 1 | 9583.42 | 1599.78 | 3997.30 | 13631.70 |  |  |  |
| 2974-61 | CNTN1 | Q12860 | 0 | 355.70 | 74.51 | 241.40 | 622.90 | -0.160 | 1.89E-03 | 6.44E-03 |
|  |  |  | 1 | 360.42 | 660.62 | 138.60 | 11307.20 |  |  |  |
| 3332-57 | HFE2 | Q6ZVN8 | 0 | 1180.91 | 194.07 | 813.00 | 2169.40 | -0.104 | 1.91E-03 | 6.46E-03 |
|  |  |  | 1 | 1114.05 | 333.90 | 614.20 | 5391.10 |  |  |  |
| 4989-7 | FGG | P02679 | 0 | 64917.19 | 13056.02 | 43422.30 | 110407.60 | 0.202 | 1.94E-03 | 6.56E-03 |
|  |  |  | 1 | 76141.80 | 13427.58 | 469.10 | 115733.10 |  |  |  |
| 3592-4 | CAMK1 | Q14012 | 0 | 7398.44 | 1780.70 | 2657.10 | 12560.80 | -0.161 | 1.96E-03 | 6.59E-03 |
|  |  |  | 1 | 6740.80 | 2301.46 | 2310.40 | 24867.20 |  |  |  |
| 3877-67 | CAMKK1 | Q8N5S9 | 0 | 461.68 | 229.32 | 212.40 | 1194.00 | 0.277 | 2.01E-03 | 6.75E-03 |
|  |  |  | 1 | 647.01 | 1265.47 | 204.20 | 21633.70 |  |  |  |
| 3166-92 | CD33 | P20138 | 0 | 4955.47 | 2293.34 | 747.10 | 18076.90 | -0.261 | 2.14E-03 | 7.19E-03 |
|  |  |  | 1 | 4226.52 | 1913.78 | 274.10 | 11764.10 |  |  |  |
| 2500-2 | ANGPT4 | Q9Y264 | 0 | 881.45 | 269.18 | 536.50 | 1673.20 | 0.187 | 2.22E-03 | 7.41E-03 |
|  |  |  | 1 | 1053.38 | 589.81 | 546.30 | 6276.70 |  |  |  |
| 3423-59 | CMA1 | P23946 | 0 | 659.27 | 247.95 | 431.70 | 2291.40 | 0.237 | 2.24E-03 | 7.47E-03 |
|  |  |  | 1 | 1010.58 | 2509.34 | 386.80 | 40049.30 |  |  |  |
| 3518-54 | CPB2 | Q96IY4 | 0 | 13801.85 | 1870.68 | 9701.50 | 19303.40 | -0.078 | 2.26E-03 | 7.54E-03 |
|  |  |  | 1 | 13133.93 | 2390.34 | 9146.30 | 35752.40 |  |  |  |
| 14158-17 | ANXA5 | P08758 | 0 | 1105.65 | 934.73 | 540.10 | 8382.00 | -0.244 | 2.41E-03 | 8.01E-03 |
|  |  |  | 1 | 1470.52 | 9849.15 | 362.30 | 170796.30 |  |  |  |
| 3891-56 | MBD4 | O95243 | 0 | 609.63 | 329.77 | 417.30 | 3301.10 | 0.198 | 2.53E-03 | 8.39E-03 |
|  |  |  | 1 | 759.34 | 804.70 | 392.00 | 8610.90 |  |  |  |
| 4985-11 | FABP5 | Q01469 | 0 | 990.55 | 593.74 | 519.40 | 5643.60 | 0.201 | 2.55E-03 | 8.43E-03 |
|  |  |  | 1 | 1242.12 | 2013.83 | 318.40 | 33596.80 |  |  |  |
| 3054-3 | HP | P00738 | 0 | 30140.25 | 16225.04 | 131.10 | 108099.40 | 0.372 | 2.59E-03 | 8.54E-03 |
|  |  |  | 1 | 36138.63 | 16967.23 | 146.30 | 90821.40 |  |  |  |
| 2580-83 | MPO | P05164 | 0 | 19867.37 | 6627.34 | 7822.80 | 47083.30 | 0.205 | 2.65E-03 | 8.69E-03 |
|  |  |  | 1 | 24244.18 | 14737.34 | 4629.30 | 140840.80 |  |  |  |
| 10358-33 | MCL1 | Q07820 | 0 | 1488.11 | 1642.71 | 835.70 | 16547.30 | 0.202 | 2.65E-03 | 8.69E-03 |
|  |  |  | 1 | 1958.42 | 6997.39 | 611.00 | 121969.80 |  |  |  |
| 2192-63 | CCL27 | Q9Y4X3 | 0 | 1632.66 | 781.66 | 1056.30 | 8948.60 | -0.122 | 2.66E-03 | 8.71E-03 |
|  |  |  | 1 | 1494.95 | 575.54 | 756.80 | 7602.30 |  |  |  |
| 5110-84 | NRXN1 | P58400 | 0 | 868.34 | 611.71 | 506.00 | 5660.90 | -0.152 | 2.68E-03 | 8.74E-03 |
|  |  |  | 1 | 754.46 | 282.90 | 305.80 | 2822.10 |  |  |  |
| 3611-70 | ECE1 | P42892 | 0 | 21417.21 | 4464.97 | 10168.50 | 37491.60 | -0.126 | 2.74E-03 | 8.92E-03 |
|  |  |  | 1 | 19828.29 | 4980.55 | 5589.30 | 47526.40 |  |  |  |
| 2829-19 | IL27 EBI3 | Q8NEV9 Q14213 | 0 | 926.83 | 867.58 | 589.70 | 7146.90 | -0.170 | 2.75E-03 | 8.92E-03 |
|  |  |  | 1 | 794.38 | 682.27 | 297.30 | 11163.20 |  |  |  |
| 5183-53 | PRKAA1 PRKAB1 PRKAG1 | Q13131 Q9Y478 P54619 | 0 | 470.36 | 255.48 | 338.80 | 2582.10 | 0.204 | 2.80E-03 | 9.06E-03 |
|  |  |  | 1 | 659.84 | 1898.53 | 316.80 | 32839.40 |  |  |  |
| 4917-62 | ITGAV ITGB5 | P06756 P18084 | 0 | 744.29 | 1303.75 | 343.80 | 13049.30 | 0.212 | 2.82E-03 | 9.11E-03 |
|  |  |  | 1 | 813.78 | 1106.81 | 231.50 | 16678.60 |  |  |  |
| 3174-2 | ADAMTS1 | Q9UHI8 | 0 | 451.26 | 117.43 | 309.80 | 965.60 | 0.229 | 2.88E-03 | 9.27E-03 |
|  |  |  | 1 | 948.12 | 6128.95 | 295.60 | 105616.80 |  |  |  |
| 3169-70 | IDUA | P35475 | 0 | 1856.43 | 742.27 | 707.20 | 5848.50 | 0.171 | 2.89E-03 | 9.29E-03 |
|  |  |  | 1 | 2082.52 | 743.31 | 513.10 | 5665.10 |  |  |  |
| 2501-51 | CDH1 | P12830 | 0 | 83234.22 | 15635.47 | 31879.40 | 130513.40 | -0.120 | 2.90E-03 | 9.31E-03 |
|  |  |  | 1 | 77489.62 | 19355.79 | 17180.30 | 228073.70 |  |  |  |
| 4150-75 | PLG | P00747 | 0 | 785.53 | 157.66 | 638.10 | 1957.20 | 0.099 | 2.95E-03 | 9.43E-03 |
|  |  |  | 1 | 852.59 | 259.80 | 576.00 | 3814.20 |  |  |  |
| 9168-31 | CCL26 | Q9Y258 | 0 | 1249.66 | 612.14 | 753.00 | 5190.30 | 0.236 | 3.06E-03 | 9.77E-03 |
|  |  |  | 1 | 1927.65 | 5120.81 | 420.10 | 71932.50 |  |  |  |
| 3470-1 | SELE | P16581 | 0 | 32855.22 | 11400.52 | 13310.60 | 77707.00 | 0.189 | 3.15E-03 | 1.00E-02 |
|  |  |  | 1 | 37926.24 | 14048.06 | 9435.30 | 92248.30 |  |  |  |
| 2686-67 | IGFBP6 | P24592 | 0 | 812.48 | 196.82 | 472.50 | 1537.30 | -0.110 | 3.16E-03 | 1.00E-02 |
|  |  |  | 1 | 751.18 | 173.39 | 376.80 | 1594.30 |  |  |  |
| 5337-64 | CD86 | P42081 | 0 | 1133.73 | 262.43 | 564.40 | 2088.40 | -0.209 | 3.20E-03 | 1.01E-02 |
|  |  |  | 1 | 1128.44 | 1294.27 | 274.20 | 16388.70 |  |  |  |
| 2988-57 | ICOS | Q9Y6W8 | 0 | 881.76 | 416.80 | 637.20 | 4614.00 | 0.186 | 3.27E-03 | 1.03E-02 |
|  |  |  | 1 | 1089.41 | 955.91 | 537.20 | 11473.80 |  |  |  |
| 4449-67 | GREM1 | O60565 | 0 | 533.13 | 334.51 | 353.70 | 3257.00 | 0.172 | 3.30E-03 | 1.04E-02 |
|  |  |  | 1 | 618.77 | 598.95 | 334.60 | 9848.50 |  |  |  |
| 5100-53 | SCARB2 | Q14108 | 0 | 510.18 | 199.47 | 391.80 | 2055.90 | 0.141 | 3.36E-03 | 1.06E-02 |
|  |  |  | 1 | 574.69 | 289.51 | 363.60 | 3379.50 |  |  |  |
| 2744-57 | IGHG1 IGHG2 IGHG3 IGHG4 IGK@ IGL@ | P01857 | 0 | 201404.74 | 33433.52 | 137390.20 | 326313.90 | 0.098 | 3.38E-03 | 1.06E-02 |
|  |  |  | 1 | 216770.50 | 37563.90 | 23739.70 | 342436.70 |  |  |  |
| 5121-3 | SEMA6B | Q9H3T3 | 0 | 3833.90 | 1143.91 | 887.00 | 7461.00 | -0.227 | 3.54E-03 | 1.11E-02 |
|  |  |  | 1 | 3452.17 | 1400.08 | 445.50 | 8337.50 |  |  |  |
| 3710-49 | PLG | P00747 | 0 | 22658.28 | 4522.64 | 9692.50 | 33631.00 | -0.118 | 3.56E-03 | 1.11E-02 |
|  |  |  | 1 | 21111.30 | 5668.81 | 8394.80 | 63810.00 |  |  |  |
| 4254-6 | NUDCD3 | Q8IVD9 | 0 | 1352.23 | 8603.56 | 220.60 | 86473.00 | -0.244 | 3.59E-03 | 1.12E-02 |
|  |  |  | 1 | 435.61 | 298.14 | 145.30 | 3772.30 |  |  |  |
| 3839-60 | AIP | O00170 | 0 | 10755.17 | 7904.48 | 3121.50 | 35043.70 | 0.362 | 3.65E-03 | 1.13E-02 |
|  |  |  | 1 | 14767.41 | 10869.79 | 2657.50 | 46329.40 |  |  |  |
| 5254-69 | PDE3A | Q14432 | 0 | 1193.73 | 865.46 | 772.00 | 8882.10 | 0.186 | 3.65E-03 | 1.13E-02 |
|  |  |  | 1 | 1419.16 | 1977.20 | 696.40 | 34085.30 |  |  |  |
| 5139-32 | UNC5C | O95185 | 0 | 4631.37 | 944.19 | 2799.00 | 7121.90 | -0.121 | 3.67E-03 | 1.14E-02 |
|  |  |  | 1 | 4308.83 | 1086.65 | 790.20 | 8180.50 |  |  |  |
| 3852-19 | DNAJB1 | P25685 | 0 | 840.42 | 572.98 | 213.70 | 2652.10 | 0.375 | 3.73E-03 | 1.15E-02 |
|  |  |  | 1 | 1325.51 | 2056.50 | 246.60 | 32235.00 |  |  |  |
| 5343-74 | CPE | P16870 | 0 | 2175.19 | 453.40 | 1203.30 | 4130.20 | -0.129 | 3.75E-03 | 1.15E-02 |
|  |  |  | 1 | 2053.31 | 922.47 | 1038.20 | 10646.30 |  |  |  |
| 8479-4 | MMP10 | P09238 | 0 | 914.26 | 426.74 | 344.40 | 3481.10 | 0.261 | 3.88E-03 | 1.19E-02 |
|  |  |  | 1 | 1259.30 | 1562.32 | 274.30 | 24448.60 |  |  |  |
| 4124-24 | HSPA1A | P0DMV8 | 0 | 9443.28 | 2547.95 | 4180.70 | 19762.20 | -0.151 | 3.95E-03 | 1.21E-02 |
|  |  |  | 1 | 8663.40 | 2822.61 | 2486.00 | 23787.10 |  |  |  |
| 3069-52 | IGHM IGJ IGK@ IGL@ | P01871 | 0 | 3021.00 | 1767.69 | 836.30 | 10030.80 | 0.301 | 4.06E-03 | 1.24E-02 |
|  |  |  | 1 | 4089.31 | 3791.41 | 671.00 | 44059.60 |  |  |  |
| 5011-11 | NAMPT | P43490 | 0 | 759.61 | 243.83 | 405.40 | 1967.20 | -0.173 | 4.06E-03 | 1.24E-02 |
|  |  |  | 1 | 735.81 | 851.38 | 320.80 | 10558.00 |  |  |  |
| 5354-11 | KRT18 | P05783 | 0 | 1179.12 | 361.57 | 729.30 | 2581.30 | 0.166 | 4.18E-03 | 1.27E-02 |
|  |  |  | 1 | 1392.16 | 1092.33 | 676.00 | 16540.90 |  |  |  |
| 4479-14 | SERPING1 | P05155 | 0 | 7432.26 | 2069.78 | 3998.80 | 15337.10 | -0.140 | 4.19E-03 | 1.27E-02 |
|  |  |  | 1 | 6800.94 | 2100.03 | 2383.90 | 16085.60 |  |  |  |
| 5843-60 | EHMT2 | Q96KQ7 | 0 | 594.34 | 299.69 | 302.60 | 3034.90 | -0.136 | 4.21E-03 | 1.28E-02 |
|  |  |  | 1 | 527.46 | 167.38 | 283.20 | 1710.30 |  |  |  |
| 9172-69 | MMP8 | P22894 | 0 | 2543.45 | 1531.56 | 914.50 | 9475.80 | 0.277 | 4.46E-03 | 1.35E-02 |
|  |  |  | 1 | 3838.11 | 9013.60 | 908.20 | 134833.80 |  |  |  |
| 2827-23 | CX3CL1 | P78423 | 0 | 3781.11 | 1052.77 | 1758.50 | 8812.60 | -0.161 | 4.50E-03 | 1.36E-02 |
|  |  |  | 1 | 3463.23 | 1166.99 | 649.00 | 8315.90 |  |  |  |
| 3315-15 | GPC2 | Q8N158 | 0 | 194.06 | 75.64 | 136.60 | 607.80 | 0.190 | 4.72E-03 | 1.41E-02 |
|  |  |  | 1 | 249.88 | 321.36 | 134.10 | 4852.40 |  |  |  |
| 4717-55 | IL3 | P08700 | 0 | 1611.62 | 440.78 | 1119.00 | 4611.30 | 0.114 | 4.71E-03 | 1.41E-02 |
|  |  |  | 1 | 1771.03 | 633.33 | 1079.40 | 6262.20 |  |  |  |
| 11514-196 | CD59 | P13987 | 0 | 2026.78 | 373.44 | 1268.60 | 3045.40 | -0.098 | 4.72E-03 | 1.41E-02 |
|  |  |  | 1 | 1903.94 | 418.21 | 768.70 | 3910.60 |  |  |  |
| 5236-2 | NR1D1 | P20393 | 0 | 587.78 | 229.78 | 410.40 | 2211.20 | 0.152 | 4.77E-03 | 1.42E-02 |
|  |  |  | 1 | 681.37 | 521.55 | 362.40 | 8179.40 |  |  |  |
| 3210-1 | METAP1 | P53582 | 0 | 3674.90 | 3141.25 | 498.00 | 14531.50 | 0.490 | 4.81E-03 | 1.43E-02 |
|  |  |  | 1 | 6337.55 | 6463.22 | 479.50 | 58558.50 |  |  |  |
| 3323-37 | LRP8 | Q14114 | 0 | 2286.14 | 817.87 | 1295.00 | 8598.80 | -0.126 | 4.84E-03 | 1.44E-02 |
|  |  |  | 1 | 2093.96 | 706.28 | 861.80 | 9655.60 |  |  |  |
| 4904-7 | CASP2 | P42575 | 0 | 491.56 | 655.28 | 286.90 | 6872.10 | 0.250 | 4.85E-03 | 1.44E-02 |
|  |  |  | 1 | 795.48 | 2842.15 | 278.90 | 46300.80 |  |  |  |
| 5457-5 | COLEC12 | Q5KU26 | 0 | 3270.03 | 971.74 | 1673.80 | 6575.80 | -0.190 | 4.92E-03 | 1.46E-02 |
|  |  |  | 1 | 3308.17 | 7083.63 | 693.70 | 124199.70 |  |  |  |
| 4144-13 | CCL13 | Q99616 | 0 | 608.73 | 615.62 | 360.70 | 5273.30 | 0.243 | 5.05E-03 | 1.49E-02 |
|  |  |  | 1 | 1174.22 | 8149.48 | 379.80 | 141450.50 |  |  |  |
| 3400-49 | TBK1 | Q9UHD2 | 0 | 328.36 | 115.14 | 195.40 | 1138.50 | 0.137 | 5.15E-03 | 1.52E-02 |
|  |  |  | 1 | 366.29 | 171.28 | 210.90 | 2475.00 |  |  |  |
| 2441-2 | FGF10 | O15520 | 0 | 302.49 | 178.68 | 173.80 | 1464.30 | 0.158 | 5.30E-03 | 1.56E-02 |
|  |  |  | 1 | 334.08 | 183.51 | 175.70 | 1867.20 |  |  |  |
| 4127-75 | C6 | P13671 | 0 | 92085.64 | 15904.19 | 45234.80 | 135191.20 | -0.136 | 5.30E-03 | 1.56E-02 |
|  |  |  | 1 | 85933.81 | 20742.99 | 15535.10 | 150897.00 |  |  |  |
| 3200-49 | KLK13 | Q9UKR3 | 0 | 1198.99 | 182.44 | 880.00 | 1870.50 | -0.087 | 5.45E-03 | 1.59E-02 |
|  |  |  | 1 | 1141.05 | 261.87 | 629.70 | 2733.70 |  |  |  |
| 3879-50 | CDC37 | Q16543 | 0 | 636.67 | 250.79 | 338.00 | 1473.00 | 0.203 | 5.50E-03 | 1.60E-02 |
|  |  |  | 1 | 789.31 | 692.61 | 323.90 | 8604.40 |  |  |  |
| 2614-28 | EFNA4 | P52798 | 0 | 2239.71 | 486.68 | 1258.80 | 3687.90 | -0.137 | 5.54E-03 | 1.61E-02 |
|  |  |  | 1 | 2081.17 | 621.73 | 304.10 | 4817.60 |  |  |  |
| 3350-53 | CAMK2A | Q9UQM7 | 0 | 965.11 | 697.96 | 371.50 | 3738.80 | 0.298 | 5.67E-03 | 1.65E-02 |
|  |  |  | 1 | 1268.20 | 1111.14 | 338.40 | 9397.60 |  |  |  |
| 3046-31 | RETN | Q9HD89 | 0 | 1199.69 | 381.74 | 550.50 | 2796.80 | 0.180 | 5.69E-03 | 1.65E-02 |
|  |  |  | 1 | 1586.54 | 3772.57 | 610.50 | 65953.30 |  |  |  |
| 9201-13 | TAGLN2 | P37802 | 0 | 7469.86 | 4629.09 | 2318.40 | 22491.90 | -0.292 | 5.69E-03 | 1.65E-02 |
|  |  |  | 1 | 6416.01 | 4386.65 | 1644.80 | 24932.20 |  |  |  |
| 3320-49 | IGFBP7 | Q16270 | 0 | 59103.85 | 11623.19 | 27458.60 | 91824.70 | -0.113 | 5.83E-03 | 1.68E-02 |
|  |  |  | 1 | 55285.36 | 14164.37 | 8474.00 | 133427.30 |  |  |  |
| 4158-54 | PLAU | P00749 | 0 | 2373.46 | 527.15 | 1374.70 | 3614.60 | -0.160 | 5.96E-03 | 1.72E-02 |
|  |  |  | 1 | 2317.41 | 2685.85 | 471.00 | 46675.60 |  |  |  |
| 3821-28 | MAPKAPK5 | Q8IW41 | 0 | 789.45 | 425.51 | 440.70 | 2649.80 | 0.231 | 6.00E-03 | 1.73E-02 |
|  |  |  | 1 | 1304.72 | 5453.02 | 465.10 | 92338.90 |  |  |  |
| 7640-29 | LRP1B | Q9NZR2 | 0 | 43753.11 | 8544.54 | 27680.60 | 66795.80 | -0.088 | 6.05E-03 | 1.74E-02 |
|  |  |  | 1 | 41117.68 | 7865.88 | 26310.10 | 63657.60 |  |  |  |
| 10366-11 | PDGFRA | P16234 | 0 | 1610.24 | 502.07 | 551.20 | 3283.00 | -0.201 | 6.07E-03 | 1.74E-02 |
|  |  |  | 1 | 1550.62 | 1441.99 | 409.90 | 16343.40 |  |  |  |
| 13102-1 | FAM3D | Q96BQ1 | 0 | 15815.93 | 12081.29 | 4033.10 | 53515.60 | -0.309 | 6.20E-03 | 1.77E-02 |
|  |  |  | 1 | 12256.76 | 9146.75 | 485.60 | 74853.40 |  |  |  |
| 3189-61 | TMPRSS15 | P98073 | 0 | 329.79 | 90.41 | 229.10 | 1116.60 | 0.129 | 6.33E-03 | 1.80E-02 |
|  |  |  | 1 | 397.84 | 643.24 | 227.60 | 11311.40 |  |  |  |
| 5443-62 | NPPA | P01160 | 0 | 942.47 | 474.65 | 512.20 | 3437.70 | 0.183 | 6.46E-03 | 1.84E-02 |
|  |  |  | 1 | 1148.77 | 1579.46 | 543.00 | 22263.30 |  |  |  |
| 2762-30 | FGF19 | O95750 | 0 | 12108.86 | 8060.93 | 1272.70 | 38459.50 | -0.311 | 6.57E-03 | 1.86E-02 |
|  |  |  | 1 | 9950.14 | 6927.66 | 884.30 | 39067.40 |  |  |  |
| 3376-49 | IL17RD | Q8NFM7 | 0 | 570.66 | 155.38 | 391.40 | 1671.10 | -0.095 | 6.80E-03 | 1.92E-02 |
|  |  |  | 1 | 535.01 | 162.15 | 243.00 | 2682.60 |  |  |  |
| 3028-36 | CCL23 | P55773 | 0 | 74252.35 | 20596.83 | 25350.20 | 140597.90 | -0.168 | 6.79E-03 | 1.92E-02 |
|  |  |  | 1 | 67748.80 | 23370.38 | 5620.80 | 161396.90 |  |  |  |
| 3178-5 | CTSC | P53634 | 0 | 702.43 | 622.27 | 487.00 | 6650.40 | -0.141 | 6.91E-03 | 1.95E-02 |
|  |  |  | 1 | 650.31 | 848.14 | 364.90 | 14135.10 |  |  |  |
| 5484-63 | RPS3A | P61247 | 0 | 1033.43 | 1254.84 | 471.60 | 12647.50 | 0.203 | 6.95E-03 | 1.95E-02 |
|  |  |  | 1 | 1167.68 | 1183.85 | 499.90 | 15690.50 |  |  |  |
| 11104-13 | CHI3L1 | P36222 | 0 | 22794.54 | 25469.81 | 2746.40 | 190896.90 | 0.439 | 7.00E-03 | 1.96E-02 |
|  |  |  | 1 | 31194.92 | 32308.72 | 1568.30 | 244620.70 |  |  |  |
| 8462-18 | GH1 | P01241 | 0 | 582.36 | 1731.33 | 239.70 | 17271.10 | 0.271 | 7.03E-03 | 1.97E-02 |
|  |  |  | 1 | 749.31 | 3582.48 | 227.90 | 61738.30 |  |  |  |
| 2938-55 | APOE | P02649 | 0 | 276652.90 | 44013.11 | 192224.90 | 381427.30 | -0.090 | 7.12E-03 | 1.99E-02 |
|  |  |  | 1 | 262352.86 | 55336.15 | 118116.20 | 531158.70 |  |  |  |
| 3199-54 | KLK12 | Q9UKR0 | 0 | 6090.43 | 1546.36 | 3252.20 | 10869.60 | -0.154 | 7.17E-03 | 2.00E-02 |
|  |  |  | 1 | 5653.83 | 1999.42 | 1567.30 | 14389.90 |  |  |  |
| 3419-49 | CAMK2D | Q13557 | 0 | 7343.07 | 6375.06 | 2107.30 | 28192.10 | 0.343 | 7.60E-03 | 2.11E-02 |
|  |  |  | 1 | 9753.09 | 8318.06 | 1452.30 | 51061.50 |  |  |  |
| 5481-16 | RASA1 | P20936 | 0 | 1843.55 | 4704.50 | 534.50 | 45425.20 | -0.231 | 7.70E-03 | 2.14E-02 |
|  |  |  | 1 | 1332.22 | 1844.40 | 402.00 | 19903.10 |  |  |  |
| 4245-80 | MDM2 | Q00987 | 0 | 294.86 | 112.13 | 182.50 | 879.70 | 0.155 | 7.74E-03 | 2.14E-02 |
|  |  |  | 1 | 335.95 | 162.61 | 178.10 | 2179.80 |  |  |  |
| 5825-49 | IFNGR1 | P15260 | 0 | 502.54 | 170.61 | 199.70 | 1239.10 | -0.179 | 7.80E-03 | 2.16E-02 |
|  |  |  | 1 | 471.65 | 292.16 | 161.70 | 2694.40 |  |  |  |
| 4324-33 | CST2 | P09228 | 0 | 26278.90 | 10776.52 | 4499.80 | 59900.90 | -0.234 | 7.85E-03 | 2.17E-02 |
|  |  |  | 1 | 23576.03 | 12332.52 | 1811.00 | 73106.40 |  |  |  |
| 3761-4 | PTGS2 | P35354 | 0 | 706.71 | 351.92 | 503.00 | 3140.30 | 0.181 | 7.93E-03 | 2.18E-02 |
|  |  |  | 1 | 866.70 | 761.68 | 394.80 | 9934.00 |  |  |  |
| 5095-21 | KIR2DL4 | Q99706 | 0 | 1681.44 | 615.96 | 508.50 | 3595.10 | 0.208 | 8.09E-03 | 2.22E-02 |
|  |  |  | 1 | 2070.81 | 1406.50 | 450.90 | 14643.40 |  |  |  |
| 5476-66 | PRKCG | P05129 | 0 | 804.55 | 1116.01 | 423.80 | 10487.60 | -0.142 | 8.12E-03 | 2.23E-02 |
|  |  |  | 1 | 633.76 | 301.13 | 407.30 | 3551.00 |  |  |  |
| 9183-7 | IFNAR1 | P17181 | 0 | 1709.02 | 748.57 | 453.10 | 7539.10 | -0.189 | 8.17E-03 | 2.24E-02 |
|  |  |  | 1 | 1551.60 | 773.64 | 337.80 | 8837.00 |  |  |  |
| 2751-16 | AZU1 | P20160 | 0 | 325.72 | 132.41 | 164.40 | 901.00 | 0.198 | 8.29E-03 | 2.26E-02 |
|  |  |  | 1 | 403.06 | 351.15 | 165.90 | 5107.00 |  |  |  |
| 3321-2 | IL24 | Q13007 | 0 | 380.69 | 584.38 | 165.70 | 5985.60 | 0.200 | 8.54E-03 | 2.32E-02 |
|  |  |  | 1 | 405.05 | 299.56 | 174.60 | 3556.00 |  |  |  |
| 3079-62 | RARRES2 | Q99969 | 0 | 3402.78 | 758.92 | 1833.80 | 6726.80 | 0.112 | 8.56E-03 | 2.32E-02 |
|  |  |  | 1 | 3810.95 | 2981.78 | 1441.40 | 53258.90 |  |  |  |
| 2879-9 | SERPINA3 | P01011 | 0 | 267843.46 | 35286.77 | 193234.30 | 365652.30 | 0.057 | 8.58E-03 | 2.32E-02 |
|  |  |  | 1 | 278468.13 | 35773.80 | 176942.40 | 436563.90 |  |  |  |
| 5345-51 | CKAP2 | Q8WWK9 | 0 | 16084.94 | 9048.95 | 10118.90 | 102907.20 | -0.126 | 8.58E-03 | 2.32E-02 |
|  |  |  | 1 | 14777.44 | 6364.33 | 4495.70 | 84514.10 |  |  |  |
| 3893-64 | MSLN | Q13421 | 0 | 448.93 | 160.19 | 235.20 | 897.50 | 0.241 | 8.67E-03 | 2.34E-02 |
|  |  |  | 1 | 894.76 | 4576.57 | 219.40 | 76199.50 |  |  |  |
| 5909-51 | NME1 | P15531 | 0 | 694.01 | 241.29 | 449.30 | 1389.90 | 0.212 | 8.69E-03 | 2.34E-02 |
|  |  |  | 1 | 1464.37 | 10986.82 | 380.60 | 190965.60 |  |  |  |
| 2514-65 | EFNB3 | Q15768 | 0 | 1096.44 | 281.22 | 750.40 | 2563.90 | 0.132 | 8.90E-03 | 2.40E-02 |
|  |  |  | 1 | 1254.23 | 700.40 | 662.10 | 9400.90 |  |  |  |
| 6641-60 | UBB | P0CG47 | 0 | 2058.37 | 681.93 | 1090.40 | 6145.90 | 0.160 | 9.05E-03 | 2.43E-02 |
|  |  |  | 1 | 2615.56 | 5185.89 | 1098.40 | 89930.50 |  |  |  |
| 4960-72 | ANXA1 | P04083 | 0 | 6793.76 | 4871.35 | 2529.40 | 40252.40 | 0.310 | 9.16E-03 | 2.46E-02 |
|  |  |  | 1 | 10592.53 | 12774.29 | 418.30 | 90794.30 |  |  |  |
| 4332-6 | CLEC1B | Q9P126 | 0 | 18155.65 | 11572.67 | 5838.70 | 52384.30 | 0.276 | 9.38E-03 | 2.51E-02 |
|  |  |  | 1 | 22326.77 | 13392.09 | 3775.70 | 88232.50 |  |  |  |
| 3132-1 | VEGFC | P49767 | 0 | 1582.09 | 542.71 | 898.00 | 3026.30 | 0.172 | 9.48E-03 | 2.52E-02 |
|  |  |  | 1 | 1912.70 | 1630.31 | 870.20 | 18146.20 |  |  |  |
| 4712-28 | APOD | P05090 | 0 | 5393.38 | 1057.55 | 2637.80 | 8058.40 | -0.132 | 9.46E-03 | 2.52E-02 |
|  |  |  | 1 | 5044.88 | 1339.35 | 732.40 | 9043.20 |  |  |  |
| 5062-60 | CD226 | Q15762 | 0 | 1019.29 | 267.09 | 668.70 | 2225.30 | 0.127 | 9.76E-03 | 2.59E-02 |
|  |  |  | 1 | 1170.54 | 819.76 | 693.40 | 11253.30 |  |  |  |
| 4697-59 | CSF2 | P04141 | 0 | 296.70 | 80.17 | 202.00 | 831.20 | 0.165 | 9.81E-03 | 2.60E-02 |
|  |  |  | 1 | 376.45 | 394.11 | 198.20 | 4739.40 |  |  |  |
| 5854-60 | MAPT | P10636 | 0 | 255.61 | 230.37 | 160.10 | 2219.90 | 0.242 | 1.02E-02 | 2.71E-02 |
|  |  |  | 1 | 465.08 | 2069.65 | 156.30 | 33931.40 |  |  |  |
| 3503-4 | ITGA1 ITGB1 | P56199 P05556 | 0 | 1369.69 | 978.44 | 544.70 | 5428.60 | 0.219 | 1.03E-02 | 2.73E-02 |
|  |  |  | 1 | 1580.51 | 1028.49 | 466.50 | 9908.50 |  |  |  |
| 5016-61 | PARK7 | Q99497 | 0 | 527.80 | 304.02 | 269.50 | 2633.60 | 0.179 | 1.04E-02 | 2.74E-02 |
|  |  |  | 1 | 719.20 | 1759.64 | 259.40 | 26071.00 |  |  |  |
| 2780-35 | LTF | P02788 | 0 | 46506.01 | 18294.51 | 16230.50 | 151402.80 | 0.162 | 1.04E-02 | 2.75E-02 |
|  |  |  | 1 | 52770.33 | 24600.65 | 16833.30 | 234500.70 |  |  |  |
| 2864-2 | MAP2K1 | Q02750 | 0 | 453.19 | 75.64 | 339.60 | 931.90 | 0.091 | 1.08E-02 | 2.82E-02 |
|  |  |  | 1 | 494.88 | 207.19 | 281.80 | 3424.80 |  |  |  |
| 2516-57 | CCL21 | O00585 | 0 | 34042.53 | 8688.03 | 16179.90 | 59786.50 | 0.129 | 1.09E-02 | 2.85E-02 |
|  |  |  | 1 | 37892.34 | 12263.47 | 8537.10 | 93713.70 |  |  |  |
| 3234-23 | CCDC80 | Q76M96 | 0 | 2623.09 | 491.24 | 1395.10 | 4075.80 | -0.108 | 1.09E-02 | 2.85E-02 |
|  |  |  | 1 | 2487.14 | 762.92 | 958.80 | 9669.70 |  |  |  |
| 5242-37 | MAP2K4 | P45985 | 0 | 49310.16 | 15635.95 | 18772.10 | 104886.30 | -0.146 | 1.09E-02 | 2.85E-02 |
|  |  |  | 1 | 45012.84 | 15001.58 | 10523.70 | 109943.70 |  |  |  |
| 5097-14 | KIR3DS1 | Q14943 | 0 | 505.01 | 186.87 | 336.60 | 1484.40 | 0.169 | 1.11E-02 | 2.88E-02 |
|  |  |  | 1 | 631.57 | 712.04 | 326.50 | 8661.40 |  |  |  |
| 2358-19 | FLT4 | P35916 | 0 | 8370.73 | 2061.86 | 3737.40 | 13010.90 | -0.116 | 1.11E-02 | 2.88E-02 |
|  |  |  | 1 | 7770.81 | 2030.58 | 2289.60 | 15200.60 |  |  |  |
| 4430-44 | COLEC11 | Q9BWP8 | 0 | 16121.10 | 5550.52 | 5891.90 | 34714.70 | -0.199 | 1.11E-02 | 2.88E-02 |
|  |  |  | 1 | 15150.57 | 9684.58 | 3645.00 | 81538.00 |  |  |  |
| 5012-67 | AK1 | P00568 | 0 | 33964.41 | 15215.91 | 12037.50 | 87947.60 | 0.223 | 1.12E-02 | 2.90E-02 |
|  |  |  | 1 | 41594.39 | 23077.69 | 6965.60 | 188715.30 |  |  |  |
| 5900-11 | HINT1 | P49773 | 0 | 1625.22 | 603.83 | 782.70 | 3134.80 | 0.169 | 1.13E-02 | 2.91E-02 |
|  |  |  | 1 | 1895.09 | 1161.33 | 676.90 | 16175.20 |  |  |  |
| 2967-8 | VCAM1 | P19320 | 0 | 15110.73 | 3791.51 | 7612.90 | 27539.20 | -0.127 | 1.15E-02 | 2.96E-02 |
|  |  |  | 1 | 14178.48 | 6228.21 | 4695.40 | 94921.10 |  |  |  |
| 3450-4 | KLK6 | Q92876 | 0 | 2264.91 | 415.24 | 1292.20 | 3167.60 | -0.142 | 1.17E-02 | 3.01E-02 |
|  |  |  | 1 | 2538.47 | 7924.86 | 884.10 | 138451.10 |  |  |  |
| 5227-60 | PDK1 | Q15118 | 0 | 3158.87 | 3131.66 | 1039.10 | 23338.20 | 0.261 | 1.23E-02 | 3.15E-02 |
|  |  |  | 1 | 3789.93 | 3993.80 | 872.50 | 49548.90 |  |  |  |
| 4500-50 | CLEC11A | Q9Y240 | 0 | 2743.29 | 873.46 | 1256.50 | 6526.20 | 0.153 | 1.23E-02 | 3.15E-02 |
|  |  |  | 1 | 3141.47 | 1313.57 | 748.30 | 10738.30 |  |  |  |
| 2857-70 | NR3C1 | P04150 | 0 | 122.70 | 60.70 | 62.90 | 428.60 | 0.178 | 1.24E-02 | 3.17E-02 |
|  |  |  | 1 | 150.72 | 198.99 | 59.20 | 3177.00 |  |  |  |
| 13119-26 | SERPINA10 | Q9UK55 | 0 | 5990.16 | 1824.76 | 2702.90 | 12415.70 | -0.131 | 1.24E-02 | 3.18E-02 |
|  |  |  | 1 | 5501.36 | 1832.19 | 1496.50 | 13440.90 |  |  |  |
| 5000-52 | LGALS3BP | Q08380 | 0 | 1486.77 | 579.37 | 576.10 | 4034.70 | 0.152 | 1.27E-02 | 3.24E-02 |
|  |  |  | 1 | 1654.78 | 608.30 | 428.90 | 3951.80 |  |  |  |
| 3709-4 | GPT | P24298 | 0 | 4578.59 | 1433.33 | 2827.60 | 9829.30 | -0.127 | 1.27E-02 | 3.24E-02 |
|  |  |  | 1 | 4251.93 | 1728.92 | 2168.20 | 18845.30 |  |  |  |
| 3859-50 | PSMA1 | P25786 | 0 | 722.35 | 183.90 | 440.60 | 2071.90 | 0.123 | 1.28E-02 | 3.25E-02 |
|  |  |  | 1 | 841.21 | 789.85 | 398.90 | 12926.70 |  |  |  |
| 3889-64 | LMNB1 | P20700 | 0 | 844.03 | 211.33 | 527.30 | 1731.80 | 0.130 | 1.36E-02 | 3.44E-02 |
|  |  |  | 1 | 983.11 | 859.84 | 511.90 | 13716.40 |  |  |  |
| 3314-74 | GFRA1 | P56159 | 0 | 1484.33 | 388.26 | 776.70 | 2378.70 | -0.124 | 1.37E-02 | 3.47E-02 |
|  |  |  | 1 | 1380.96 | 435.36 | 288.80 | 3847.40 |  |  |  |
| 3387-1 | PAK3 | O75914 | 0 | 1196.28 | 279.93 | 819.20 | 3308.10 | -0.107 | 1.38E-02 | 3.48E-02 |
|  |  |  | 1 | 1151.11 | 653.76 | 456.00 | 10399.80 |  |  |  |
| 2911-27 | MDK | P21741 | 0 | 30080.27 | 8393.59 | 3072.00 | 51207.40 | 0.158 | 1.39E-02 | 3.50E-02 |
|  |  |  | 1 | 34605.26 | 15517.19 | 5188.30 | 169904.80 |  |  |  |
| 4971-1 | CTSZ | Q9UBR2 | 0 | 6553.72 | 2213.54 | 2887.20 | 17791.20 | -0.125 | 1.40E-02 | 3.51E-02 |
|  |  |  | 1 | 5961.12 | 1752.54 | 1763.00 | 11243.50 |  |  |  |
| 5253-1 | PDE1A | P54750 | 0 | 1063.11 | 1170.55 | 511.20 | 10802.50 | 0.190 | 1.44E-02 | 3.62E-02 |
|  |  |  | 1 | 1193.06 | 1096.14 | 469.60 | 11086.30 |  |  |  |
| 3902-21 | SKP1 | P63208 | 0 | 5453.87 | 2482.36 | 2412.20 | 12420.40 | 0.185 | 1.46E-02 | 3.64E-02 |
|  |  |  | 1 | 6360.56 | 3639.16 | 2300.60 | 41269.10 |  |  |  |
| 4123-60 | FGF4 | P08620 | 0 | 286.60 | 141.79 | 177.30 | 1468.20 | 0.153 | 1.46E-02 | 3.64E-02 |
|  |  |  | 1 | 337.37 | 315.27 | 175.60 | 4570.00 |  |  |  |
| 2875-15 | TBP | P20226 | 0 | 576.10 | 141.91 | 430.90 | 1535.60 | 0.130 | 1.47E-02 | 3.66E-02 |
|  |  |  | 1 | 670.24 | 379.83 | 244.80 | 3770.20 |  |  |  |
| 8467-9 | INHBA INHBB | P08476 P09529 | 0 | 993.93 | 457.89 | 426.80 | 3742.30 | -0.162 | 1.48E-02 | 3.67E-02 |
|  |  |  | 1 | 944.32 | 1335.31 | 268.80 | 23337.80 |  |  |  |
| 4249-64 | NME2 | P22392 | 0 | 28476.18 | 18675.43 | 2913.40 | 73450.90 | -0.394 | 1.49E-02 | 3.71E-02 |
|  |  |  | 1 | 26520.66 | 20480.23 | 470.50 | 104619.00 |  |  |  |
| 3505-6 | LTA LTB | P01374 Q06643 | 0 | 126.77 | 30.78 | 62.00 | 201.80 | -0.140 | 1.53E-02 | 3.80E-02 |
|  |  |  | 1 | 122.79 | 100.46 | 42.30 | 1628.40 |  |  |  |
| 2558-51 | POMC | P01189 | 0 | 1064.11 | 423.75 | 479.00 | 3124.60 | -0.184 | 1.53E-02 | 3.80E-02 |
|  |  |  | 1 | 1433.34 | 8373.68 | 465.00 | 145635.70 |  |  |  |
| 3289-19 | CA10 | Q9NS85 | 0 | 769.27 | 141.28 | 490.30 | 1317.00 | -0.105 | 1.55E-02 | 3.84E-02 |
|  |  |  | 1 | 744.12 | 387.13 | 340.00 | 5203.40 |  |  |  |
| 3805-16 | ESM1 | Q9NQ30 | 0 | 1289.70 | 368.78 | 763.20 | 2889.00 | -0.114 | 1.60E-02 | 3.94E-02 |
|  |  |  | 1 | 1216.17 | 647.88 | 581.00 | 10808.10 |  |  |  |
| 4911-49 | GSTP1 | P09211 | 0 | 5261.05 | 1616.57 | 1974.20 | 10306.70 | -0.151 | 1.65E-02 | 4.07E-02 |
|  |  |  | 1 | 4851.93 | 1744.87 | 872.70 | 13664.70 |  |  |  |
| 5358-3 | OMD | Q99983 | 0 | 7664.82 | 3030.82 | 2684.90 | 19988.70 | -0.151 | 1.66E-02 | 4.09E-02 |
|  |  |  | 1 | 6883.18 | 2687.56 | 1436.70 | 22333.70 |  |  |  |
| 3606-2 | DDR2 | Q16832 | 0 | 1814.53 | 374.97 | 1291.00 | 3543.60 | 0.081 | 1.67E-02 | 4.10E-02 |
|  |  |  | 1 | 1940.27 | 612.03 | 1288.70 | 9887.10 |  |  |  |
| 2966-65 | CLEC11A | Q9Y240 | 0 | 1172.12 | 328.59 | 655.40 | 2474.40 | 0.126 | 1.72E-02 | 4.21E-02 |
|  |  |  | 1 | 1307.71 | 477.35 | 484.60 | 3906.30 |  |  |  |
| 3738-54 | CSF1 | P09603 | 0 | 1427.33 | 332.20 | 1065.70 | 3687.90 | 0.093 | 1.74E-02 | 4.26E-02 |
|  |  |  | 1 | 1546.40 | 495.69 | 928.30 | 5534.20 |  |  |  |
| 4397-26 | IFNL2 | Q8IZJ0 | 0 | 182.94 | 68.95 | 124.70 | 634.90 | 0.127 | 1.81E-02 | 4.42E-02 |
|  |  |  | 1 | 206.34 | 108.57 | 121.90 | 964.30 |  |  |  |
| 13112-179 | FSTL1 | Q12841 | 0 | 35050.93 | 4700.05 | 25392.30 | 48552.80 | -0.067 | 1.83E-02 | 4.47E-02 |
|  |  |  | 1 | 33656.58 | 5549.81 | 6795.20 | 57782.20 |  |  |  |
| 4548-4 | FUT3 | P21217 | 0 | 635.02 | 394.03 | 205.30 | 3376.20 | -0.176 | 1.84E-02 | 4.47E-02 |
|  |  |  | 1 | 558.08 | 365.81 | 192.00 | 5336.20 |  |  |  |
| 4394-71 | FGF8 | P55075 | 0 | 546.76 | 485.65 | 188.30 | 4683.10 | 0.223 | 1.86E-02 | 4.50E-02 |
|  |  |  | 1 | 668.81 | 745.16 | 186.30 | 11270.10 |  |  |  |
| 4559-64 | KYNU | Q16719 | 0 | 1169.28 | 406.91 | 410.50 | 2786.60 | 0.155 | 1.86E-02 | 4.51E-02 |
|  |  |  | 1 | 1344.05 | 636.38 | 393.60 | 7348.40 |  |  |  |
| 3073-51 | IL18BP | O95998 | 0 | 11858.73 | 3323.88 | 4989.10 | 20325.40 | -0.157 | 1.88E-02 | 4.52E-02 |
|  |  |  | 1 | 11061.92 | 4346.80 | 514.20 | 38948.00 |  |  |  |
| 4840-73 | CSF3 | P09919 | 0 | 1429.14 | 1088.55 | 871.40 | 11576.70 | -0.175 | 1.87E-02 | 4.52E-02 |
|  |  |  | 1 | 1374.07 | 1513.76 | 248.70 | 19338.00 |  |  |  |
| 4156-74 | TGFB2 | P61812 | 0 | 360.86 | 326.50 | 200.40 | 2850.00 | 0.197 | 1.88E-02 | 4.52E-02 |
|  |  |  | 1 | 824.80 | 7248.63 | 215.30 | 125728.80 |  |  |  |
| 3420-21 | CA13 | Q8N1Q1 | 0 | 4170.94 | 4468.20 | 286.30 | 27982.70 | -0.442 | 1.94E-02 | 4.67E-02 |
|  |  |  | 1 | 3712.88 | 3920.91 | 206.40 | 28023.40 |  |  |  |
| 3412-7 | BCL2 | P10415 | 0 | 1663.75 | 655.27 | 834.40 | 3460.80 | 0.174 | 1.97E-02 | 4.72E-02 |
|  |  |  | 1 | 2000.45 | 1528.68 | 754.30 | 20257.70 |  |  |  |
| 3630-27 | CADM3 | Q8N126 | 0 | 1693.13 | 503.30 | 969.30 | 3870.20 | -0.122 | 1.98E-02 | 4.73E-02 |
|  |  |  | 1 | 1637.84 | 1337.21 | 716.60 | 21149.10 |  |  |  |
| 5430-66 | SIRPA | P78324 | 0 | 7366.33 | 4082.56 | 504.80 | 23104.80 | -0.385 | 2.01E-02 | 4.81E-02 |
|  |  |  | 1 | 6206.15 | 5539.23 | 478.30 | 79681.30 |  |  |  |
| 3848-14 | GAPDH | P04406 | 0 | 124696.21 | 74078.17 | 22174.70 | 303263.10 | 0.287 | 2.05E-02 | 4.88E-02 |
|  |  |  | 1 | 160902.43 | 96854.98 | 20638.80 | 457943.60 |  |  |  |
| 4886-3 | CCL7 | P80098 | 0 | 765.38 | 262.43 | 302.10 | 1889.70 | 0.149 | 2.09E-02 | 4.98E-02 |
|  |  |  | 1 | 874.33 | 369.76 | 197.20 | 2990.30 |  |  |  |
| 4278-14 | P4HB | P07237 | 0 | 265.29 | 56.90 | 173.80 | 471.70 | -0.084 | 2.11E-02 | 4.99E-02 |
|  |  |  | 1 | 252.21 | 69.13 | 141.80 | 893.90 |  |  |  |
| 4564-2 | PLXNC1 | O60486 | 0 | 852.36 | 164.21 | 145.30 | 1237.90 | -0.097 | 2.11E-02 | 4.99E-02 |
|  |  |  | 1 | 803.75 | 257.84 | 292.70 | 3933.40 |  |  |  |
| 2687-2 | MIA | Q16674 | 0 | 3721.42 | 931.06 | 1879.40 | 7105.60 | -0.115 | 2.11E-02 | 4.99E-02 |
|  |  |  | 1 | 3618.55 | 3456.41 | 845.90 | 60932.10 |  |  |  |
| 3361-26 | CD207 | Q9UJ71 | 0 | 220.28 | 72.19 | 153.60 | 835.40 | -0.085 | 2.15E-02 | 5.08E-02 |
|  |  |  | 1 | 209.28 | 85.93 | 137.40 | 1398.40 |  |  |  |
| 2640-3 | MST1R | Q04912 | 0 | 605.15 | 183.60 | 400.40 | 1868.50 | -0.093 | 2.15E-02 | 5.08E-02 |
|  |  |  | 1 | 582.85 | 436.64 | 348.60 | 7734.10 |  |  |  |
| 5244-12 | MAPK9 | P45984 | 0 | 1059.78 | 195.19 | 686.20 | 1636.20 | -0.099 | 2.17E-02 | 5.10E-02 |
|  |  |  | 1 | 1019.29 | 462.04 | 276.10 | 7590.50 |  |  |  |
| 5400-52 | LEPR | P48357 | 0 | 2278.90 | 854.88 | 176.10 | 4438.90 | -0.213 | 2.18E-02 | 5.13E-02 |
|  |  |  | 1 | 2001.53 | 1052.15 | 168.70 | 14507.80 |  |  |  |
| 3798-71 | CA9 | Q16790 | 0 | 1676.08 | 725.62 | 745.50 | 4426.80 | 0.150 | 2.24E-02 | 5.26E-02 |
|  |  |  | 1 | 1878.76 | 913.07 | 757.80 | 6739.20 |  |  |  |
| 3643-90 | SLITRK1 | Q96PX8 | 0 | 1798.39 | 502.39 | 1323.00 | 5706.10 | 0.087 | 2.26E-02 | 5.30E-02 |
|  |  |  | 1 | 1924.99 | 569.45 | 1015.50 | 5801.10 |  |  |  |
| 4981-6 | DSC3 | Q14574 | 0 | 590.07 | 1418.93 | 252.40 | 14004.10 | -0.172 | 2.28E-02 | 5.32E-02 |
|  |  |  | 1 | 451.22 | 895.40 | 217.90 | 14242.40 |  |  |  |
| 4428-1 | CHST2 | Q9Y4C5 | 0 | 462.04 | 342.61 | 285.00 | 3192.20 | 0.156 | 2.30E-02 | 5.36E-02 |
|  |  |  | 1 | 543.18 | 691.14 | 260.60 | 11073.30 |  |  |  |
| 4336-2 | SAA1 | P0DJI8 | 0 | 1634.05 | 2130.23 | 124.10 | 10596.20 | 0.468 | 2.30E-02 | 5.36E-02 |
|  |  |  | 1 | 3089.68 | 9129.16 | 123.60 | 124945.50 |  |  |  |
| 5384-67 | BRF1 | Q92994 | 0 | 2210.07 | 1258.53 | 1495.40 | 13247.90 | 0.105 | 2.31E-02 | 5.38E-02 |
|  |  |  | 1 | 2358.01 | 1116.59 | 1562.90 | 16201.90 |  |  |  |
| 3719-2 | CDKN1B | P46527 | 0 | 1437.23 | 2004.25 | 647.80 | 19674.20 | 0.227 | 2.38E-02 | 5.52E-02 |
|  |  |  | 1 | 2179.00 | 9267.95 | 539.90 | 158398.10 |  |  |  |
| 5070-76 | TNFRSF6B | O95407 | 0 | 793.79 | 606.10 | 507.20 | 6619.60 | 0.104 | 2.49E-02 | 5.78E-02 |
|  |  |  | 1 | 832.51 | 349.89 | 482.10 | 4333.90 |  |  |  |
| 2731-29 | POR | P16435 | 0 | 4447.05 | 1937.09 | 1859.00 | 14938.20 | 0.156 | 2.50E-02 | 5.78E-02 |
|  |  |  | 1 | 5108.58 | 2992.79 | 1166.20 | 32944.00 |  |  |  |
| 8470-213 | RNASEH1 | O60930 | 0 | 337.24 | 571.14 | 149.70 | 4880.60 | 0.189 | 2.51E-02 | 5.79E-02 |
|  |  |  | 1 | 353.93 | 449.53 | 157.20 | 6326.50 |  |  |  |
| 3520-58 | TGFB3 | P10600 | 0 | 559.74 | 173.74 | 392.30 | 1944.50 | 0.088 | 2.52E-02 | 5.81E-02 |
|  |  |  | 1 | 599.26 | 189.27 | 350.30 | 1754.70 |  |  |  |
| 5468-67 | IL17RC | Q8NAC3 | 0 | 970.42 | 452.01 | 558.90 | 4876.60 | -0.150 | 2.52E-02 | 5.81E-02 |
|  |  |  | 1 | 1119.90 | 4072.63 | 218.70 | 70686.00 |  |  |  |
| 2752-62 | GDF5 | P43026 | 0 | 2052.12 | 459.13 | 1194.80 | 4482.50 | 0.092 | 2.57E-02 | 5.90E-02 |
|  |  |  | 1 | 2219.63 | 715.73 | 1167.40 | 8075.40 |  |  |  |
| 5090-49 | LILRB1 | Q8NHL6 | 0 | 4806.12 | 1360.97 | 1997.00 | 7599.10 | -0.165 | 2.57E-02 | 5.90E-02 |
|  |  |  | 1 | 4410.42 | 1374.34 | 119.60 | 8219.50 |  |  |  |
| 14151-4 | ISG15 | P05161 | 0 | 2465.43 | 1524.33 | 899.30 | 12776.40 | 0.236 | 2.58E-02 | 5.92E-02 |
|  |  |  | 1 | 3727.79 | 8598.99 | 443.40 | 127190.70 |  |  |  |
| 5301-7 | CCL11 | P51671 | 0 | 12888.63 | 4132.81 | 6769.70 | 34923.50 | 0.148 | 2.73E-02 | 6.23E-02 |
|  |  |  | 1 | 14999.24 | 7488.84 | 2600.80 | 65940.50 |  |  |  |
| 3403-1 | TPSB2 | P20231 | 0 | 4165.80 | 2160.74 | 678.40 | 10206.40 | -0.220 | 2.76E-02 | 6.29E-02 |
|  |  |  | 1 | 3564.71 | 1864.05 | 622.10 | 8876.20 |  |  |  |
| 3232-28 | ACP5 | P13686 | 0 | 7837.45 | 1706.41 | 4489.40 | 12417.20 | 0.092 | 2.76E-02 | 6.29E-02 |
|  |  |  | 1 | 8437.68 | 2200.64 | 4067.40 | 16291.90 |  |  |  |
| 2746-56 | CRLF2 | Q9HC73 | 0 | 1608.44 | 458.98 | 1017.30 | 3250.80 | 0.122 | 2.78E-02 | 6.31E-02 |
|  |  |  | 1 | 1820.97 | 1032.79 | 921.40 | 15080.10 |  |  |  |
| 2877-3 | UBE2I | P63279 | 0 | 2893.37 | 1852.28 | 789.10 | 8472.30 | 0.258 | 2.80E-02 | 6.34E-02 |
|  |  |  | 1 | 3904.46 | 4165.31 | 723.90 | 53735.40 |  |  |  |
| 3452-17 | LCK | P06239 | 0 | 479.37 | 1130.41 | 250.60 | 11643.00 | 0.162 | 2.80E-02 | 6.34E-02 |
|  |  |  | 1 | 545.71 | 1346.60 | 269.60 | 20837.90 |  |  |  |
| 3388-58 | PAK7 | Q9P286 | 0 | 827.52 | 257.73 | 529.40 | 2367.20 | 0.117 | 2.81E-02 | 6.35E-02 |
|  |  |  | 1 | 924.80 | 447.16 | 436.10 | 6271.80 |  |  |  |
| 4355-13 | DAPK2 | Q9UIK4 | 0 | 3033.72 | 979.27 | 1797.70 | 7309.30 | 0.131 | 2.84E-02 | 6.41E-02 |
|  |  |  | 1 | 3430.09 | 1517.81 | 1588.10 | 12029.80 |  |  |  |
| 4915-64 | HBA1 HBB | P69905 P68871 | 0 | 8033.54 | 43631.07 | 479.00 | 325811.10 | 0.263 | 2.85E-02 | 6.43E-02 |
|  |  |  | 1 | 4491.62 | 23228.38 | 639.50 | 284354.20 |  |  |  |
| 4988-49 | FGFR4 | P22455 | 0 | 1384.61 | 1038.61 | 781.70 | 7039.10 | 0.210 | 2.86E-02 | 6.43E-02 |
|  |  |  | 1 | 2309.83 | 8168.13 | 679.40 | 123657.20 |  |  |  |
| 2977-7 | EDAR | Q9UNE0 | 0 | 1675.01 | 1532.37 | 396.00 | 8966.80 | -0.248 | 2.86E-02 | 6.43E-02 |
|  |  |  | 1 | 1386.60 | 1163.96 | 324.30 | 8688.10 |  |  |  |
| 2436-49 | CXCL16 | Q9H2A7 | 0 | 13827.55 | 2402.20 | 8151.70 | 19680.10 | -0.082 | 2.93E-02 | 6.56E-02 |
|  |  |  | 1 | 13217.97 | 3030.64 | 3769.40 | 21483.20 |  |  |  |
| 2598-9 | TNFRSF9 | Q07011 | 0 | 374.76 | 81.24 | 263.60 | 869.30 | 0.086 | 2.95E-02 | 6.60E-02 |
|  |  |  | 1 | 405.52 | 137.72 | 193.50 | 1564.40 |  |  |  |
| 4139-71 | IL6R | P08887 | 0 | 15954.32 | 4239.25 | 6308.20 | 26323.10 | -0.110 | 2.99E-02 | 6.67E-02 |
|  |  |  | 1 | 14911.35 | 4409.81 | 2118.90 | 27970.00 |  |  |  |
| 2603-61 | CD80 | P33681 | 0 | 705.83 | 345.14 | 450.00 | 2551.70 | -0.138 | 3.00E-02 | 6.69E-02 |
|  |  |  | 1 | 819.88 | 3396.09 | 403.20 | 59166.40 |  |  |  |
| 2567-5 | CFI | P05156 | 0 | 48476.04 | 5361.46 | 37511.50 | 62978.90 | -0.041 | 3.01E-02 | 6.70E-02 |
|  |  |  | 1 | 47124.80 | 5019.10 | 22847.10 | 63700.60 |  |  |  |
| 4785-30 | SERPINA6 | P08185 | 0 | 845.87 | 391.08 | 584.30 | 4345.50 | 0.142 | 3.09E-02 | 6.87E-02 |
|  |  |  | 1 | 1066.93 | 1427.17 | 475.90 | 17382.00 |  |  |  |
| 3360-50 | CHEK2 | O96017 | 0 | 360.32 | 523.40 | 147.60 | 3749.50 | 0.305 | 3.10E-02 | 6.89E-02 |
|  |  |  | 1 | 954.54 | 5186.68 | 145.00 | 81021.40 |  |  |  |
| 9215-117 | USP25 | Q9UHP3 | 0 | 1480.40 | 689.30 | 863.90 | 5766.90 | 0.128 | 3.14E-02 | 6.95E-02 |
|  |  |  | 1 | 1658.53 | 1149.59 | 884.50 | 14982.50 |  |  |  |
| 2945-25 | ESR1 | P03372 | 0 | 1191.51 | 2300.42 | 656.50 | 23764.20 | 0.135 | 3.19E-02 | 7.05E-02 |
|  |  |  | 1 | 1161.50 | 866.79 | 679.40 | 10069.50 |  |  |  |
| 4910-21 | PLA2G1B | P04054 | 0 | 1209.91 | 2926.82 | 476.20 | 29890.80 | -0.143 | 3.20E-02 | 7.06E-02 |
|  |  |  | 1 | 874.25 | 480.07 | 321.30 | 4954.30 |  |  |  |
| 3622-33 | LGMN | Q99538 | 0 | 3028.78 | 461.17 | 2125.90 | 5058.60 | 0.064 | 3.23E-02 | 7.11E-02 |
|  |  |  | 1 | 3196.14 | 749.68 | 2114.70 | 10109.70 |  |  |  |
| 5363-51 | SEMA3E | O15041 | 0 | 3264.10 | 1220.32 | 726.10 | 6932.30 | -0.154 | 3.25E-02 | 7.16E-02 |
|  |  |  | 1 | 2967.02 | 1292.91 | 712.60 | 9929.70 |  |  |  |
| 5364-7 | SET | Q01105 | 0 | 655.07 | 98.24 | 488.70 | 936.00 | 0.056 | 3.28E-02 | 7.20E-02 |
|  |  |  | 1 | 682.08 | 107.30 | 289.40 | 1148.90 |  |  |  |
| 10342-55 | PIAS4 | Q8N2W9 | 0 | 972.91 | 604.97 | 572.50 | 5398.90 | 0.106 | 3.30E-02 | 7.24E-02 |
|  |  |  | 1 | 1023.99 | 434.48 | 542.60 | 5065.60 |  |  |  |
| 13105-7 | SNAP25 | P60880 | 0 | 2572.75 | 4336.32 | 933.50 | 38768.00 | -0.184 | 3.32E-02 | 7.28E-02 |
|  |  |  | 1 | 2177.24 | 5913.27 | 682.90 | 100737.50 |  |  |  |
| 3284-75 | BGN | P21810 | 0 | 1926.16 | 772.28 | 458.50 | 4469.60 | -0.171 | 3.40E-02 | 7.42E-02 |
|  |  |  | 1 | 1787.14 | 979.76 | 400.30 | 8694.50 |  |  |  |
| 8468-19 | KLK3 | P07288 | 0 | 3430.14 | 3146.26 | 292.80 | 13146.90 | -0.360 | 3.46E-02 | 7.55E-02 |
|  |  |  | 1 | 2537.33 | 2664.30 | 297.10 | 21388.20 |  |  |  |
| 3066-12 | LGALS3 | P17931 | 0 | 23543.37 | 5177.50 | 11358.20 | 34822.20 | 0.086 | 3.52E-02 | 7.68E-02 |
|  |  |  | 1 | 25189.68 | 8885.07 | 9631.40 | 145138.70 |  |  |  |
| 5858-6 | YWHAZ | P63104 | 0 | 180259.66 | 71969.06 | 23966.90 | 344749.20 | -0.237 | 3.53E-02 | 7.68E-02 |
|  |  |  | 1 | 170844.48 | 88890.91 | 18200.00 | 410706.00 |  |  |  |
| 10346-5 | STAT3 | P40763 | 0 | 2290.53 | 1844.31 | 465.60 | 8813.10 | 0.309 | 3.54E-02 | 7.68E-02 |
|  |  |  | 1 | 3255.97 | 3028.07 | 409.50 | 17312.00 |  |  |  |
| 2975-19 | CTGF | P29279 | 0 | 1815.69 | 913.59 | 805.90 | 7888.10 | 0.196 | 3.55E-02 | 7.69E-02 |
|  |  |  | 1 | 3447.41 | 18045.72 | 844.80 | 302428.90 |  |  |  |
| 3730-81 | TNFRSF4 | P43489 | 0 | 736.78 | 187.50 | 469.90 | 1597.00 | 0.097 | 3.66E-02 | 7.92E-02 |
|  |  |  | 1 | 811.39 | 401.49 | 477.00 | 4700.20 |  |  |  |
| 3472-40 | BIRC5 | O15392 | 0 | 667.65 | 172.51 | 438.50 | 1739.10 | -0.089 | 3.69E-02 | 7.97E-02 |
|  |  |  | 1 | 692.96 | 1332.63 | 388.00 | 23581.50 |  |  |  |
| 5255-22 | PDE4D | Q08499 | 0 | 1746.74 | 845.49 | 1047.30 | 8143.90 | -0.102 | 3.70E-02 | 7.97E-02 |
|  |  |  | 1 | 1626.03 | 782.94 | 996.30 | 9680.70 |  |  |  |
| 3366-51 | ECM1 | Q16610 | 0 | 20139.72 | 5064.88 | 9661.40 | 35761.60 | 0.078 | 3.70E-02 | 7.97E-02 |
|  |  |  | 1 | 21162.07 | 4487.47 | 7045.00 | 33665.20 |  |  |  |
| 4276-10 | PEBP1 | P30086 | 0 | 8037.13 | 3743.01 | 2648.40 | 18542.50 | 0.188 | 3.71E-02 | 7.98E-02 |
|  |  |  | 1 | 9794.69 | 6284.92 | 1836.50 | 48756.60 |  |  |  |
| 3591-51 | CDH6 | P55285 | 0 | 604.89 | 167.17 | 408.00 | 1725.30 | -0.087 | 3.73E-02 | 8.00E-02 |
|  |  |  | 1 | 580.40 | 290.10 | 293.30 | 4824.00 |  |  |  |
| 3644-5 | DKKL1 | Q9UK85 | 0 | 3586.02 | 686.87 | 2602.70 | 5965.70 | -0.083 | 3.81E-02 | 8.16E-02 |
|  |  |  | 1 | 3469.30 | 1365.24 | 1766.00 | 18857.40 |  |  |  |
| 2637-77 | MRC1 | P22897 | 0 | 12084.80 | 2907.67 | 6497.70 | 20864.90 | 0.092 | 3.82E-02 | 8.17E-02 |
|  |  |  | 1 | 12973.98 | 3507.95 | 3057.80 | 28685.20 |  |  |  |
| 9191-8 | TFF2 | Q03403 | 0 | 11189.85 | 4318.67 | 4584.40 | 33909.10 | -0.172 | 3.84E-02 | 8.19E-02 |
|  |  |  | 1 | 10668.32 | 5733.88 | 650.50 | 46595.70 |  |  |  |
| 3151-6 | IL2RA | P01589 | 0 | 1311.81 | 996.74 | 836.00 | 8945.80 | 0.125 | 3.84E-02 | 8.20E-02 |
|  |  |  | 1 | 1476.69 | 2386.20 | 482.60 | 41241.50 |  |  |  |
| 4543-65 | COL23A1 | Q86Y22 | 0 | 661.76 | 381.80 | 220.30 | 1830.30 | 0.207 | 3.85E-02 | 8.20E-02 |
|  |  |  | 1 | 812.37 | 533.87 | 241.90 | 2707.10 |  |  |  |
| 8469-41 | IGFBP2 | P18065 | 0 | 763.15 | 428.28 | 212.90 | 2098.50 | 0.157 | 3.95E-02 | 8.37E-02 |
|  |  |  | 1 | 831.27 | 407.58 | 292.90 | 2378.90 |  |  |  |
| 2943-5 | CYP3A4 | P08684 | 0 | 22468.61 | 12387.93 | 6623.00 | 59067.50 | -0.214 | 3.94E-02 | 8.37E-02 |
|  |  |  | 1 | 20708.95 | 13362.51 | 4984.10 | 69289.60 |  |  |  |
| 2960-66 | CFP | P27918 | 0 | 119512.18 | 17697.54 | 58561.00 | 167195.30 | -0.183 | 3.96E-02 | 8.38E-02 |
|  |  |  | 1 | 111697.49 | 25921.86 | 30.40 | 169303.60 |  |  |  |
| 5229-90 | IMPDH1 | P20839 | 0 | 2366.83 | 2201.98 | 781.30 | 18087.20 | 0.197 | 3.97E-02 | 8.40E-02 |
|  |  |  | 1 | 2633.52 | 1827.26 | 598.00 | 14065.40 |  |  |  |
| 2748-3 | INHBA | P08476 | 0 | 4311.41 | 1263.12 | 1576.50 | 8870.60 | -0.170 | 3.99E-02 | 8.41E-02 |
|  |  |  | 1 | 4115.39 | 1845.13 | 290.20 | 19070.30 |  |  |  |
| 3381-24 | LYN | P07948 | 0 | 46934.39 | 31786.49 | 3611.30 | 133228.20 | -0.333 | 3.98E-02 | 8.41E-02 |
|  |  |  | 1 | 44804.29 | 34453.93 | 2748.40 | 136117.80 |  |  |  |
| 5947-90 | THPO | P40225 | 0 | 332.01 | 145.41 | 128.70 | 1195.20 | -0.142 | 4.02E-02 | 8.46E-02 |
|  |  |  | 1 | 316.80 | 231.78 | 101.30 | 2933.20 |  |  |  |
| 3373-5 | GZMH | P20718 | 0 | 291.87 | 144.79 | 202.70 | 1308.30 | 0.116 | 4.05E-02 | 8.51E-02 |
|  |  |  | 1 | 340.40 | 387.94 | 212.10 | 5541.10 |  |  |  |
| 5030-52 | SIRT2 | Q8IXJ6 | 0 | 9575.77 | 8895.60 | 4542.50 | 63696.50 | 0.170 | 4.20E-02 | 8.81E-02 |
|  |  |  | 1 | 12100.58 | 22582.74 | 4265.60 | 290004.10 |  |  |  |
| 5918-5 | PSME1 | Q06323 | 0 | 1626.52 | 804.84 | 781.00 | 6326.30 | 0.159 | 4.23E-02 | 8.86E-02 |
|  |  |  | 1 | 1991.77 | 2722.59 | 694.10 | 45646.90 |  |  |  |
| 5068-54 | CRTAM | O95727 | 0 | 1104.86 | 921.95 | 550.90 | 8975.10 | 0.184 | 4.27E-02 | 8.87E-02 |
|  |  |  | 1 | 1496.64 | 3074.21 | 470.80 | 41653.20 |  |  |  |
| 2806-49 | ACVR1B | P36896 | 0 | 312.31 | 112.47 | 213.70 | 1069.70 | 0.119 | 4.25E-02 | 8.87E-02 |
|  |  |  | 1 | 367.54 | 311.12 | 162.80 | 3268.00 |  |  |  |
| 5392-73 | FAS | P25445 | 0 | 2004.55 | 392.39 | 1169.40 | 3056.00 | -0.081 | 4.26E-02 | 8.87E-02 |
|  |  |  | 1 | 1922.13 | 569.92 | 891.40 | 7257.60 |  |  |  |
| 4905-63 | COTL1 | Q14019 | 0 | 2615.09 | 508.40 | 997.90 | 3809.90 | -0.105 | 4.26E-02 | 8.87E-02 |
|  |  |  | 1 | 2544.96 | 1407.58 | 823.50 | 20732.30 |  |  |  |
| 3173-49 | NAAA | Q02083 | 0 | 9032.89 | 6423.03 | 2244.10 | 51792.50 | -0.208 | 4.25E-02 | 8.87E-02 |
|  |  |  | 1 | 7905.62 | 4851.62 | 399.20 | 40305.20 |  |  |  |
| 2449-1 | PLA2G5 | P39877 | 0 | 377.16 | 86.82 | 257.80 | 760.80 | 0.083 | 4.32E-02 | 8.96E-02 |
|  |  |  | 1 | 406.23 | 165.29 | 252.00 | 2734.10 |  |  |  |
| 5903-91 | HSPA8 | P11142 | 0 | 2162.46 | 875.26 | 1325.10 | 9610.50 | 0.118 | 4.35E-02 | 9.01E-02 |
|  |  |  | 1 | 2491.61 | 1945.29 | 1143.40 | 27105.70 |  |  |  |
| 3184-25 | F7 | P08709 | 0 | 639.21 | 159.40 | 295.90 | 1512.50 | -0.077 | 4.37E-02 | 9.03E-02 |
|  |  |  | 1 | 602.70 | 131.87 | 260.20 | 1329.40 |  |  |  |
| 3635-76 | OCIAD1 | Q9NX40 | 0 | 1074.88 | 202.16 | 821.60 | 2319.50 | 0.083 | 4.38E-02 | 9.04E-02 |
|  |  |  | 1 | 1196.37 | 954.19 | 688.70 | 16634.40 |  |  |  |
| 4212-5 | EPS15L1 | Q9UBC2 | 0 | 707.90 | 432.27 | 299.70 | 2896.10 | 0.178 | 4.40E-02 | 9.07E-02 |
|  |  |  | 1 | 811.42 | 474.79 | 292.30 | 3080.20 |  |  |  |
| 3489-9 | CNTF | P26441 | 0 | 231.48 | 31.22 | 159.70 | 359.30 | -0.078 | 4.57E-02 | 9.41E-02 |
|  |  |  | 1 | 228.54 | 121.35 | 127.50 | 1620.80 |  |  |  |
| 2889-37 | CTF1 | Q16619 | 0 | 811.09 | 284.30 | 598.70 | 3328.40 | -0.074 | 4.63E-02 | 9.52E-02 |
|  |  |  | 1 | 774.50 | 297.81 | 385.20 | 4634.30 |  |  |  |
| 5125-6 | SIGLEC14 | Q08ET2 | 0 | 21205.56 | 8133.93 | 8466.60 | 53285.90 | -0.255 | 4.64E-02 | 9.53E-02 |
|  |  |  | 1 | 19920.29 | 8751.84 | 259.50 | 53392.10 |  |  |  |
| 2841-13 | FRZB | Q92765 | 0 | 3656.52 | 710.22 | 2395.90 | 6654.20 | -0.080 | 4.67E-02 | 9.57E-02 |
|  |  |  | 1 | 3522.68 | 1089.61 | 1891.90 | 10727.80 |  |  |  |
| 5308-89 | ELANE | P08246 | 0 | 1507.58 | 9140.50 | 320.00 | 91538.80 | 0.176 | 4.68E-02 | 9.57E-02 |
|  |  |  | 1 | 683.46 | 683.16 | 318.70 | 7375.60 |  |  |  |
| 9197-4 | LGALS9 | O00182 | 0 | 1879.89 | 1105.77 | 1002.60 | 10869.10 | 0.123 | 4.72E-02 | 9.63E-02 |
|  |  |  | 1 | 2115.09 | 1832.02 | 649.80 | 25226.60 |  |  |  |
| 4136-40 | IL17D | Q8TAD2 | 0 | 283.87 | 189.30 | 193.80 | 2000.10 | 0.101 | 4.75E-02 | 9.69E-02 |
|  |  |  | 1 | 318.23 | 418.68 | 191.10 | 7252.80 |  |  |  |
| 3206-4 | LYVE1 | Q9Y5Y7 | 0 | 397.17 | 276.21 | 230.70 | 2911.50 | -0.088 | 4.92E-02 | 1.00E-01 |
|  |  |  | 1 | 364.23 | 236.54 | 210.40 | 4200.20 |  |  |  |
| 3654-27 | BMPER | Q8N8U9 | 0 | 696.34 | 1646.54 | 384.20 | 16930.50 | 0.143 | 4.96E-02 | 1.01E-01 |
|  |  |  | 1 | 740.02 | 1338.88 | 382.40 | 19159.90 |  |  |  |
| 5098-79 | KLRF1 | Q9NZS2 | 0 | 426.74 | 236.59 | 308.40 | 2622.10 | 0.118 | 5.01E-02 | 1.02E-01 |
|  |  |  | 1 | 520.48 | 811.45 | 290.00 | 10610.30 |  |  |  |
| 3796-79 | ANGPTL4 | Q9BY76 | 0 | 5647.47 | 3099.29 | 3227.80 | 30044.40 | 0.110 | 5.02E-02 | 1.02E-01 |
|  |  |  | 1 | 6075.84 | 3219.35 | 3098.00 | 43395.60 |  |  |  |
| 14121-24 | TNFRSF10D | Q9UBN6 | 0 | 1134.21 | 208.55 | 792.60 | 2080.10 | 0.083 | 5.06E-02 | 1.02E-01 |
|  |  |  | 1 | 1252.27 | 785.90 | 481.40 | 13383.40 |  |  |  |
| 5813-58 | EPO | P01588 | 0 | 1586.60 | 683.39 | 439.70 | 4100.60 | -0.189 | 5.08E-02 | 1.03E-01 |
|  |  |  | 1 | 1609.37 | 1854.10 | 247.90 | 26479.80 |  |  |  |
| 2997-8 | JAM2 | P57087 | 0 | 10535.41 | 1438.58 | 6676.50 | 14552.80 | -0.067 | 5.24E-02 | 1.06E-01 |
|  |  |  | 1 | 10275.75 | 3615.10 | 4919.00 | 59051.50 |  |  |  |
| 2685-21 | IGFBP5 | P24593 | 0 | 2147.19 | 265.73 | 1502.80 | 2742.80 | -0.059 | 5.32E-02 | 1.07E-01 |
|  |  |  | 1 | 2091.33 | 558.23 | 995.80 | 9359.20 |  |  |  |
| 4534-10 | PRSS22 | Q9GZN4 | 0 | 2146.18 | 546.79 | 1118.30 | 4250.60 | 0.108 | 5.33E-02 | 1.07E-01 |
|  |  |  | 1 | 2382.77 | 843.17 | 306.90 | 7098.30 |  |  |  |
| 3346-72 | AURKB | Q96GD4 | 0 | 885.45 | 238.85 | 662.60 | 2333.40 | -0.068 | 5.35E-02 | 1.07E-01 |
|  |  |  | 1 | 850.65 | 310.70 | 501.30 | 4934.30 |  |  |  |
| 2728-62 | LAMA1 LAMB1 LAMC1 | P25391 P07942 P11047 | 0 | 2830.30 | 708.26 | 1358.80 | 4774.40 | 0.090 | 5.37E-02 | 1.08E-01 |
|  |  |  | 1 | 3053.63 | 995.89 | 1015.40 | 12318.50 |  |  |  |
| 2585-2 | PRL | P01236 | 0 | 1805.29 | 726.17 | 971.20 | 6911.40 | -0.107 | 5.45E-02 | 1.09E-01 |
|  |  |  | 1 | 1694.26 | 690.26 | 463.40 | 5178.80 |  |  |  |
| 5035-7 | TYMS | P04818 | 0 | 484.76 | 200.35 | 293.20 | 2003.60 | 0.165 | 5.50E-02 | 1.10E-01 |
|  |  |  | 1 | 752.56 | 1735.39 | 255.00 | 18761.40 |  |  |  |
| 4774-62 | FAM107B | Q9H098 | 0 | 2963.27 | 4052.07 | 1124.30 | 42740.60 | -0.103 | 5.54E-02 | 1.11E-01 |
|  |  |  | 1 | 2530.98 | 1089.94 | 755.80 | 14317.00 |  |  |  |
| 3806-55 | EPHA5 | P54756 | 0 | 829.06 | 185.32 | 334.90 | 1323.10 | -0.143 | 5.60E-02 | 1.12E-01 |
|  |  |  | 1 | 913.68 | 1500.70 | 259.40 | 21247.50 |  |  |  |
| 4984-83 | ESD | P10768 | 0 | 3155.25 | 947.93 | 961.80 | 5940.10 | -0.139 | 5.82E-02 | 1.16E-01 |
|  |  |  | 1 | 3143.40 | 3121.76 | 498.60 | 51059.70 |  |  |  |
| 7660-21 | TPM4 | P67936 | 0 | 108161.06 | 63270.63 | 3490.40 | 292481.00 | -0.361 | 5.92E-02 | 1.18E-01 |
|  |  |  | 1 | 108836.58 | 74741.05 | 1086.80 | 351898.40 |  |  |  |
| 4217-49 | HSD17B10 | Q99714 | 0 | 3994.70 | 3734.21 | 571.30 | 18693.70 | 0.307 | 6.03E-02 | 1.19E-01 |
|  |  |  | 1 | 5501.85 | 4963.76 | 524.50 | 23928.50 |  |  |  |
| 4769-10 | OLFM4 | Q6UX06 | 0 | 190.25 | 44.54 | 140.30 | 436.10 | 0.071 | 6.02E-02 | 1.19E-01 |
|  |  |  | 1 | 203.07 | 68.74 | 136.20 | 694.40 |  |  |  |
| 4192-10 | AKR1A1 | P14550 | 0 | 5644.72 | 2511.33 | 2291.70 | 12734.40 | 0.156 | 6.06E-02 | 1.20E-01 |
|  |  |  | 1 | 6700.07 | 4872.47 | 1732.20 | 63494.90 |  |  |  |
| 2794-60 | SOD1 | P00441 | 0 | 1424.14 | 3275.96 | 614.00 | 33626.90 | 0.143 | 6.11E-02 | 1.20E-01 |
|  |  |  | 1 | 1352.34 | 1181.80 | 515.50 | 17910.50 |  |  |  |
| 5478-50 | FOLH1 | Q04609 | 0 | 752.04 | 549.56 | 289.90 | 4851.20 | -0.194 | 6.10E-02 | 1.20E-01 |
|  |  |  | 1 | 1132.22 | 7148.62 | 235.70 | 123646.90 |  |  |  |
| 5464-52 | GRB2 | P62993 | 0 | 39806.78 | 25165.12 | 2186.70 | 112339.80 | -0.302 | 6.12E-02 | 1.21E-01 |
|  |  |  | 1 | 38777.85 | 28304.45 | 487.30 | 124968.50 |  |  |  |
| 4563-61 | PLCG1 | P19174 | 0 | 617.22 | 366.43 | 390.90 | 4104.30 | -0.088 | 6.24E-02 | 1.23E-01 |
|  |  |  | 1 | 595.46 | 494.07 | 253.60 | 7882.90 |  |  |  |
| 2696-87 | PSPN | O60542 | 0 | 359.91 | 125.68 | 160.40 | 952.20 | -0.105 | 6.28E-02 | 1.23E-01 |
|  |  |  | 1 | 341.31 | 196.84 | 117.90 | 3089.90 |  |  |  |
| 3049-61 | PRSS1 | P07477 | 0 | 9567.89 | 3848.24 | 2577.40 | 26944.00 | -0.141 | 6.34E-02 | 1.24E-01 |
|  |  |  | 1 | 8971.94 | 5202.72 | 427.90 | 57232.30 |  |  |  |
| 2734-49 | NCR2 | O95944 | 0 | 401.17 | 97.03 | 301.10 | 1144.50 | 0.063 | 6.36E-02 | 1.24E-01 |
|  |  |  | 1 | 421.22 | 105.75 | 281.80 | 1343.90 |  |  |  |
| 5231-79 | PCSK9 | Q8NBP7 | 0 | 784.77 | 203.28 | 403.70 | 1341.00 | -0.083 | 6.37E-02 | 1.24E-01 |
|  |  |  | 1 | 743.11 | 208.63 | 355.30 | 1803.50 |  |  |  |
| 3860-7 | PSMA6 | P60900 | 0 | 1087.84 | 583.60 | 369.60 | 2614.20 | 0.175 | 6.41E-02 | 1.25E-01 |
|  |  |  | 1 | 1317.95 | 1351.40 | 391.90 | 21216.00 |  |  |  |
| 5093-47 | JAG2 | Q9Y219 | 0 | 461.30 | 267.64 | 270.40 | 1857.60 | 0.150 | 6.53E-02 | 1.27E-01 |
|  |  |  | 1 | 567.18 | 573.46 | 236.00 | 6472.30 |  |  |  |
| 3416-2 | BTK | Q06187 | 0 | 20292.10 | 17388.36 | 613.90 | 64982.70 | -0.411 | 6.53E-02 | 1.27E-01 |
|  |  |  | 1 | 21100.71 | 20412.09 | 178.50 | 102820.40 |  |  |  |
| 3676-15 | IGF2R | P11717 | 0 | 39117.04 | 7233.69 | 27099.70 | 61915.90 | -0.065 | 6.56E-02 | 1.27E-01 |
|  |  |  | 1 | 37658.54 | 8135.48 | 12658.50 | 63485.40 |  |  |  |
| 2869-68 | PRKCD | Q05655 | 0 | 5865.85 | 3711.23 | 2729.60 | 20962.90 | 0.150 | 6.60E-02 | 1.28E-01 |
|  |  |  | 1 | 6554.74 | 4110.35 | 2623.60 | 29853.40 |  |  |  |
| 3056-11 | KLRK1 | P26718 | 0 | 789.58 | 1002.07 | 403.70 | 9755.30 | 0.136 | 6.67E-02 | 1.29E-01 |
|  |  |  | 1 | 1053.44 | 5153.36 | 429.60 | 89782.80 |  |  |  |
| 2851-63 | C5 | P01031 | 0 | 13307.04 | 5467.80 | 2847.20 | 32988.20 | -0.129 | 6.66E-02 | 1.29E-01 |
|  |  |  | 1 | 12149.22 | 5120.12 | 2391.00 | 42038.50 |  |  |  |
| 5861-78 | HAAO | P46952 | 0 | 5478.41 | 2081.44 | 1906.20 | 16273.30 | 0.112 | 6.72E-02 | 1.30E-01 |
|  |  |  | 1 | 6073.39 | 3127.10 | 2313.00 | 32204.20 |  |  |  |
| 3807-1 | FGF23 | Q9GZV9 | 0 | 790.85 | 778.59 | 349.10 | 7048.90 | 0.183 | 6.79E-02 | 1.31E-01 |
|  |  |  | 1 | 1251.96 | 5296.18 | 247.50 | 90641.50 |  |  |  |
| 4493-92 | IL11 | P20809 | 0 | 381.32 | 281.73 | 234.10 | 2279.90 | 0.128 | 6.88E-02 | 1.32E-01 |
|  |  |  | 1 | 705.63 | 5345.96 | 248.60 | 92924.30 |  |  |  |
| 2747-3 | ULBP3 | Q9BZM4 | 0 | 1028.91 | 449.87 | 400.70 | 2087.60 | 0.143 | 6.97E-02 | 1.34E-01 |
|  |  |  | 1 | 1197.77 | 916.35 | 390.70 | 12304.40 |  |  |  |
| 4535-50 | BST1 | Q10588 | 0 | 10166.50 | 4272.18 | 557.60 | 23237.40 | -0.198 | 7.04E-02 | 1.35E-01 |
|  |  |  | 1 | 8909.51 | 4109.63 | 471.00 | 25089.40 |  |  |  |
| 10337-83 | CEBPB | P17676 | 0 | 1241.41 | 1142.89 | 780.20 | 12363.00 | 0.104 | 7.11E-02 | 1.36E-01 |
|  |  |  | 1 | 1359.76 | 1126.36 | 723.00 | 12145.10 |  |  |  |
| 2631-50 | IL10RB | Q08334 | 0 | 1321.85 | 192.38 | 1029.60 | 2266.30 | -0.076 | 7.17E-02 | 1.37E-01 |
|  |  |  | 1 | 1408.95 | 2734.13 | 814.90 | 48238.90 |  |  |  |
| 9175-48 | DSCAM | O60469 | 0 | 1309.91 | 402.91 | 635.20 | 3228.00 | -0.105 | 7.20E-02 | 1.38E-01 |
|  |  |  | 1 | 1296.36 | 981.65 | 540.80 | 12735.50 |  |  |  |
| 5031-10 | SPTAN1 | Q13813 | 0 | 254.92 | 96.86 | 178.70 | 913.70 | 0.109 | 7.23E-02 | 1.38E-01 |
|  |  |  | 1 | 318.45 | 709.23 | 168.70 | 12321.20 |  |  |  |
| 5246-64 | PDE2A | O00408 | 0 | 7300.35 | 1440.54 | 4979.80 | 12289.80 | 0.090 | 7.30E-02 | 1.39E-01 |
|  |  |  | 1 | 8211.46 | 5492.08 | 3333.70 | 91961.10 |  |  |  |
| 4280-47 | PSMA2 | P25787 | 0 | 605.01 | 84.48 | 445.50 | 884.10 | -0.064 | 7.39E-02 | 1.41E-01 |
|  |  |  | 1 | 592.51 | 206.15 | 267.90 | 3265.00 |  |  |  |
| 13133-73 | LTBP4 | Q8N2S1 | 0 | 1522.86 | 277.22 | 1031.20 | 2346.60 | 0.066 | 7.41E-02 | 1.41E-01 |
|  |  |  | 1 | 1613.54 | 387.60 | 316.40 | 3368.00 |  |  |  |
| 3817-18 | PRKCQ | Q04759 | 0 | 4581.80 | 3286.44 | 1437.20 | 14525.60 | 0.205 | 7.51E-02 | 1.42E-01 |
|  |  |  | 1 | 5395.24 | 3834.16 | 1140.90 | 24964.10 |  |  |  |
| 6647-55 | UBC | P0CG48 | 0 | 5904.32 | 1388.78 | 3737.60 | 12841.30 | -0.085 | 7.75E-02 | 1.47E-01 |
|  |  |  | 1 | 5749.05 | 2325.33 | 1717.20 | 20033.10 |  |  |  |
| 5936-53 | TNF | P01375 | 0 | 614.04 | 147.49 | 403.70 | 1421.70 | 0.115 | 7.81E-02 | 1.48E-01 |
|  |  |  | 1 | 881.10 | 2469.73 | 366.40 | 32856.10 |  |  |  |
| 3905-62 | UBE2N | P61088 | 0 | 8581.32 | 5920.31 | 1934.00 | 26441.00 | 0.231 | 7.95E-02 | 1.50E-01 |
|  |  |  | 1 | 11299.84 | 9614.58 | 1413.50 | 72594.00 |  |  |  |
| 4230-1 | EIF4G2 | P78344 | 0 | 4739.75 | 4271.31 | 193.30 | 20235.70 | -0.326 | 7.99E-02 | 1.51E-01 |
|  |  |  | 1 | 4511.32 | 4140.77 | 144.40 | 20586.30 |  |  |  |
| 2647-66 | GDI2 | P50395 | 0 | 24477.13 | 11375.63 | 9256.80 | 56445.70 | 0.155 | 8.06E-02 | 1.51E-01 |
|  |  |  | 1 | 28869.56 | 16682.91 | 7067.40 | 89160.90 |  |  |  |
| 3209-69 | MEPE | Q9NQ76 | 0 | 296.12 | 72.94 | 226.40 | 754.70 | 0.083 | 8.05E-02 | 1.51E-01 |
|  |  |  | 1 | 345.94 | 559.88 | 223.70 | 9854.80 |  |  |  |
| 2848-2 | WIF1 | Q9Y5W5 | 0 | 3776.89 | 901.03 | 2167.10 | 7189.50 | -0.080 | 8.03E-02 | 1.51E-01 |
|  |  |  | 1 | 3680.39 | 2336.01 | 1286.50 | 41212.20 |  |  |  |
| 7655-11 | NPPB | P16860 | 0 | 13550.14 | 13163.43 | 1339.90 | 68358.90 | -0.274 | 8.07E-02 | 1.51E-01 |
|  |  |  | 1 | 12245.55 | 15065.51 | 567.70 | 117841.70 |  |  |  |
| 6653-58 | CD47 | Q08722 | 0 | 986.41 | 419.73 | 483.50 | 2204.40 | 0.145 | 8.14E-02 | 1.52E-01 |
|  |  |  | 1 | 1211.42 | 1279.86 | 432.40 | 14548.70 |  |  |  |
| 2652-15 | PLAUR | Q03405 | 0 | 11508.37 | 2636.93 | 6003.80 | 18273.20 | 0.099 | 8.18E-02 | 1.53E-01 |
|  |  |  | 1 | 13006.72 | 8695.91 | 2812.80 | 145474.20 |  |  |  |
| 3608-12 | DPP7 | Q9UHL4 | 0 | 1758.56 | 491.59 | 1218.90 | 4408.80 | 0.083 | 8.25E-02 | 1.54E-01 |
|  |  |  | 1 | 2027.77 | 3172.15 | 1168.40 | 55915.50 |  |  |  |
| 2849-49 | AIF1 | P55008 | 0 | 3216.62 | 754.92 | 2062.60 | 6325.70 | 0.094 | 8.27E-02 | 1.54E-01 |
|  |  |  | 1 | 3609.22 | 1763.68 | 943.10 | 19403.40 |  |  |  |
| 3222-11 | SEMA3A | Q14563 | 0 | 2291.00 | 822.56 | 1421.20 | 7112.90 | 0.112 | 8.31E-02 | 1.55E-01 |
|  |  |  | 1 | 2875.93 | 5782.59 | 1308.10 | 97488.60 |  |  |  |
| 2666-53 | DCN | P07585 | 0 | 3956.57 | 722.10 | 2354.50 | 7671.20 | -0.059 | 8.51E-02 | 1.58E-01 |
|  |  |  | 1 | 3832.49 | 970.75 | 1591.70 | 14542.40 |  |  |  |
| 2760-2 | FAM107A | O95990 | 0 | 360.20 | 262.12 | 240.50 | 2905.20 | -0.067 | 8.54E-02 | 1.59E-01 |
|  |  |  | 1 | 335.94 | 156.86 | 208.90 | 2321.50 |  |  |  |
| 5792-8 | AFP | P02771 | 0 | 804.37 | 729.70 | 481.00 | 7506.80 | -0.089 | 8.58E-02 | 1.59E-01 |
|  |  |  | 1 | 729.32 | 486.45 | 435.40 | 6761.80 |  |  |  |
| 2986-49 | CXCL3 CXCL2 | P19876 P19875 | 0 | 1096.81 | 940.46 | 178.00 | 6940.10 | 0.215 | 8.67E-02 | 1.60E-01 |
|  |  |  | 1 | 1346.16 | 1659.12 | 167.00 | 23708.20 |  |  |  |
| 3867-49 | SEZ6L2 | Q6UXD5 | 0 | 2099.99 | 1491.07 | 492.90 | 10813.20 | 0.192 | 8.66E-02 | 1.60E-01 |
|  |  |  | 1 | 2696.59 | 4699.41 | 509.00 | 72566.60 |  |  |  |
| 4956-2 | EREG | O14944 | 0 | 1253.93 | 1360.59 | 524.70 | 12766.10 | 0.149 | 8.67E-02 | 1.60E-01 |
|  |  |  | 1 | 1980.03 | 11399.07 | 528.80 | 197690.10 |  |  |  |
| 4342-10 | ICAM1 | P05362 | 0 | 4892.73 | 1894.96 | 1535.30 | 11217.40 | 0.144 | 8.71E-02 | 1.60E-01 |
|  |  |  | 1 | 5758.91 | 4157.45 | 1403.50 | 50218.60 |  |  |  |
| 5248-68 | PPIF | P30405 | 0 | 15835.51 | 12395.39 | 453.50 | 66335.80 | -0.361 | 8.70E-02 | 1.60E-01 |
|  |  |  | 1 | 16522.41 | 14483.66 | 269.50 | 78861.30 |  |  |  |
| 13088-397 | BTC | P35070 | 0 | 722.03 | 823.76 | 467.50 | 8747.40 | 0.095 | 8.82E-02 | 1.62E-01 |
|  |  |  | 1 | 744.98 | 492.35 | 361.70 | 7064.90 |  |  |  |
| 5082-51 | IL1RAPL2 | Q9NP60 | 0 | 1061.28 | 333.21 | 767.40 | 2584.00 | 0.105 | 8.92E-02 | 1.64E-01 |
|  |  |  | 1 | 1307.85 | 1973.61 | 773.10 | 30096.00 |  |  |  |
| 5452-71 | ASGR1 | P07306 | 0 | 1401.63 | 573.39 | 510.70 | 4966.00 | -0.123 | 8.95E-02 | 1.64E-01 |
|  |  |  | 1 | 1342.04 | 684.14 | 436.00 | 4702.10 |  |  |  |
| 14123-34 | C10orf54 | Q9H7M9 | 0 | 577.82 | 208.09 | 367.40 | 2141.90 | 0.079 | 8.99E-02 | 1.64E-01 |
|  |  |  | 1 | 620.06 | 268.18 | 372.30 | 2828.20 |  |  |  |
| 13101-60 | SOST | Q9BQB4 | 0 | 43609.00 | 18932.78 | 9463.30 | 125222.20 | -0.154 | 9.11E-02 | 1.67E-01 |
|  |  |  | 1 | 41481.00 | 23701.99 | 2051.40 | 155197.50 |  |  |  |
| 2447-7 | PLA2G2E | Q9NZK7 | 0 | 293.89 | 38.65 | 206.70 | 442.40 | 0.062 | 9.38E-02 | 1.71E-01 |
|  |  |  | 1 | 317.09 | 131.74 | 218.60 | 1850.90 |  |  |  |
| 3832-51 | PTK6 | Q13882 | 0 | 320.50 | 218.07 | 201.60 | 1830.10 | 0.107 | 9.43E-02 | 1.72E-01 |
|  |  |  | 1 | 354.22 | 324.38 | 195.60 | 4309.90 |  |  |  |
| 3170-6 | METAP2 | P50579 | 0 | 56848.19 | 29284.12 | 19210.60 | 130934.70 | 0.172 | 9.47E-02 | 1.72E-01 |
|  |  |  | 1 | 68304.14 | 39549.32 | 15807.30 | 174545.80 |  |  |  |
| 3033-57 | LGALS2 | P05162 | 0 | 1180.42 | 4248.12 | 470.30 | 43105.50 | 0.151 | 9.65E-02 | 1.75E-01 |
|  |  |  | 1 | 1420.89 | 7470.67 | 451.40 | 127585.10 |  |  |  |
| 3201-49 | KLK5 | Q9Y337 | 0 | 842.38 | 6208.65 | 140.50 | 62294.90 | -0.127 | 9.65E-02 | 1.75E-01 |
|  |  |  | 1 | 222.58 | 198.77 | 118.60 | 2824.60 |  |  |  |
| 3497-13 | IFNA2 | P01563 | 0 | 159.70 | 31.57 | 107.60 | 371.20 | -0.059 | 9.69E-02 | 1.76E-01 |
|  |  |  | 1 | 155.56 | 46.62 | 88.40 | 500.50 |  |  |  |
| 3854-24 | NACA | Q13765 | 0 | 8978.42 | 6539.32 | 1991.50 | 35863.80 | 0.198 | 9.84E-02 | 1.78E-01 |
|  |  |  | 1 | 11031.92 | 8534.93 | 1822.50 | 55903.50 |  |  |  |
| 11102-22 | REG4 | Q9BYZ8 | 0 | 2386.05 | 969.16 | 805.00 | 6190.70 | -0.112 | 9.85E-02 | 1.78E-01 |
|  |  |  | 1 | 2230.16 | 1006.58 | 357.00 | 8536.20 |  |  |  |
| 4318-12 | PTPN6 | P29350 | 0 | 8657.41 | 6602.06 | 1285.50 | 39005.10 | -0.237 | 9.87E-02 | 1.78E-01 |
|  |  |  | 1 | 8101.71 | 7374.43 | 829.40 | 69067.90 |  |  |  |
| 5822-22 | GDNF | P39905 | 0 | 438.94 | 153.50 | 292.00 | 1459.00 | 0.103 | 1.00E-01 | 1.80E-01 |
|  |  |  | 1 | 575.61 | 1576.04 | 244.10 | 27113.10 |  |  |  |
| 4481-34 | C4A C4B | P0C0L4 P0C0L5 | 0 | 144486.88 | 24711.94 | 80248.60 | 193140.90 | -0.059 | 1.01E-01 | 1.82E-01 |
|  |  |  | 1 | 139788.80 | 28792.50 | 53663.00 | 219195.50 |  |  |  |
| 3594-6 | CTSE | P14091 | 0 | 540.39 | 311.78 | 342.60 | 2699.30 | 0.111 | 1.03E-01 | 1.86E-01 |
|  |  |  | 1 | 641.93 | 794.58 | 326.00 | 11324.70 |  |  |  |
| 3453-87 | LYN | P07948 | 0 | 10102.68 | 8210.21 | 697.10 | 35829.00 | -0.285 | 1.04E-01 | 1.87E-01 |
|  |  |  | 1 | 9994.29 | 8833.95 | 576.00 | 48455.50 |  |  |  |
| 2949-6 | PLA2G10 | O15496 | 0 | 732.16 | 273.87 | 523.70 | 2816.00 | -0.068 | 1.05E-01 | 1.87E-01 |
|  |  |  | 1 | 703.34 | 281.75 | 451.70 | 3001.80 |  |  |  |
| 2992-59 | IL17RA | Q96F46 | 0 | 879.93 | 371.51 | 348.40 | 3316.30 | -0.096 | 1.05E-01 | 1.87E-01 |
|  |  |  | 1 | 822.89 | 367.13 | 294.30 | 4171.50 |  |  |  |
| 9204-33 | POMC | P01189 | 0 | 1005.93 | 375.25 | 353.60 | 2631.00 | -0.132 | 1.04E-01 | 1.87E-01 |
|  |  |  | 1 | 986.68 | 650.52 | 142.70 | 8348.40 |  |  |  |
| 4533-76 | ADAMTS15 | Q8TE58 | 0 | 457.69 | 84.96 | 332.50 | 1016.00 | 0.072 | 1.06E-01 | 1.89E-01 |
|  |  |  | 1 | 513.80 | 418.40 | 322.20 | 5181.50 |  |  |  |
| 5312-49 | APOE | P02649 | 0 | 294416.68 | 34946.40 | 211623.60 | 377999.90 | -0.035 | 1.07E-01 | 1.89E-01 |
|  |  |  | 1 | 287798.03 | 37151.39 | 157903.70 | 424056.30 |  |  |  |
| 3418-12 | CAMK1D | Q8IU85 | 0 | 2902.98 | 3457.63 | 1795.10 | 36928.70 | -0.080 | 1.06E-01 | 1.89E-01 |
|  |  |  | 1 | 2692.23 | 3102.75 | 1648.50 | 53874.30 |  |  |  |
| 5921-58 | S100A7 | P31151 | 0 | 346.88 | 336.58 | 153.90 | 3139.80 | -0.139 | 1.06E-01 | 1.89E-01 |
|  |  |  | 1 | 331.67 | 418.42 | 116.70 | 4495.10 |  |  |  |
| 2775-54 | IL17F | Q96PD4 | 0 | 852.43 | 854.27 | 447.40 | 9110.70 | -0.091 | 1.07E-01 | 1.90E-01 |
|  |  |  | 1 | 775.59 | 388.52 | 331.80 | 4069.10 |  |  |  |
| 3405-6 | UFC1 | Q9Y3C8 | 0 | 23396.61 | 10967.67 | 6635.50 | 50206.70 | -0.152 | 1.08E-01 | 1.91E-01 |
|  |  |  | 1 | 22261.62 | 11851.57 | 2214.20 | 62003.90 |  |  |  |
| 5440-26 | TNNI2 | P48788 | 0 | 12950.56 | 20167.18 | 2201.00 | 145858.00 | -0.185 | 1.08E-01 | 1.92E-01 |
|  |  |  | 1 | 9772.35 | 8020.77 | 907.30 | 68741.70 |  |  |  |
| 4129-72 | CFB | P00751 | 0 | 30000.57 | 4217.38 | 21720.40 | 46065.20 | -0.041 | 1.09E-01 | 1.93E-01 |
|  |  |  | 1 | 29250.19 | 4882.39 | 14820.30 | 60265.30 |  |  |  |
| 9173-21 | PGM1 | P36871 | 0 | 830.69 | 1051.60 | 420.30 | 10111.30 | -0.097 | 1.10E-01 | 1.95E-01 |
|  |  |  | 1 | 692.10 | 593.72 | 379.00 | 10174.70 |  |  |  |
| 10361-25 | OAS1 | P00973 | 0 | 654.59 | 1614.52 | 283.90 | 16271.90 | 0.150 | 1.11E-01 | 1.96E-01 |
|  |  |  | 1 | 1045.68 | 6708.59 | 324.70 | 113706.60 |  |  |  |
| 3708-62 | A2M | P01023 | 0 | 16706.44 | 6078.18 | 8318.70 | 40671.30 | 0.076 | 1.11E-01 | 1.96E-01 |
|  |  |  | 1 | 17296.38 | 5070.96 | 8063.20 | 45472.10 |  |  |  |
| 5104-57 | NCR1 | O76036 | 0 | 2710.74 | 1463.49 | 1559.30 | 14834.90 | -0.097 | 1.11E-01 | 1.96E-01 |
|  |  |  | 1 | 2619.93 | 2103.86 | 831.40 | 33067.50 |  |  |  |
| 5089-11 | IL7R | P16871 | 0 | 362.72 | 261.13 | 226.60 | 2449.80 | -0.089 | 1.12E-01 | 1.97E-01 |
|  |  |  | 1 | 336.32 | 226.60 | 179.80 | 3192.10 |  |  |  |
| 2605-49 | TNFRSF8 | P28908 | 0 | 1166.97 | 353.63 | 722.30 | 3243.10 | 0.084 | 1.15E-01 | 2.01E-01 |
|  |  |  | 1 | 1295.92 | 945.63 | 538.10 | 15597.90 |  |  |  |
| 3617-80 | HGFAC | Q04756 | 0 | 3717.36 | 1105.65 | 82.60 | 6117.60 | -0.124 | 1.15E-01 | 2.02E-01 |
|  |  |  | 1 | 3412.80 | 1081.71 | 63.00 | 7373.40 |  |  |  |
| 4130-71 | FGF6 | P10767 | 0 | 554.41 | 530.87 | 196.50 | 4992.50 | 0.162 | 1.16E-01 | 2.02E-01 |
|  |  |  | 1 | 967.25 | 5950.02 | 194.60 | 103074.90 |  |  |  |
| 4542-24 | CLU | P10909 | 0 | 1321.53 | 179.58 | 932.60 | 2052.40 | -0.062 | 1.16E-01 | 2.03E-01 |
|  |  |  | 1 | 1352.67 | 1481.63 | 756.40 | 26329.30 |  |  |  |
| 4301-58 | TK1 | P04183 | 0 | 557.68 | 115.61 | 415.70 | 1246.40 | -0.054 | 1.17E-01 | 2.04E-01 |
|  |  |  | 1 | 543.23 | 155.17 | 208.20 | 2186.40 |  |  |  |
| 5475-10 | PRKCB | P05771 | 0 | 16460.69 | 14141.99 | 452.20 | 65173.40 | -0.336 | 1.21E-01 | 2.10E-01 |
|  |  |  | 1 | 17763.05 | 17187.36 | 313.00 | 79551.10 |  |  |  |
| 2616-23 | ERBB2 | P04626 | 0 | 510.98 | 281.45 | 354.30 | 3089.60 | 0.094 | 1.21E-01 | 2.11E-01 |
|  |  |  | 1 | 588.36 | 615.47 | 332.60 | 9094.10 |  |  |  |
| 2505-49 | GFRA3 | O60609 | 0 | 1267.45 | 2654.28 | 710.80 | 27300.80 | 0.083 | 1.22E-01 | 2.12E-01 |
|  |  |  | 1 | 1110.22 | 415.92 | 570.30 | 3702.10 |  |  |  |
| 3032-11 | CGA FSHB | P01215 P01225 | 0 | 2860.10 | 3439.35 | 91.50 | 14291.70 | -0.265 | 1.22E-01 | 2.12E-01 |
|  |  |  | 1 | 2120.60 | 2261.69 | 94.40 | 11623.70 |  |  |  |
| 5526-53 | TNFRSF18 | Q9Y5U5 | 0 | 1226.54 | 3136.43 | 418.70 | 31703.90 | -0.120 | 1.25E-01 | 2.16E-01 |
|  |  |  | 1 | 920.15 | 666.29 | 257.20 | 6444.00 |  |  |  |
| 4900-8 | C3 | P01024 | 0 | 1460.96 | 6796.76 | 243.10 | 68421.10 | -0.171 | 1.25E-01 | 2.17E-01 |
|  |  |  | 1 | 913.31 | 1882.51 | 145.40 | 25796.30 |  |  |  |
| 3379-29 | PRKCI | P41743 | 0 | 3385.04 | 1897.63 | 1224.20 | 10264.10 | 0.145 | 1.27E-01 | 2.19E-01 |
|  |  |  | 1 | 3907.86 | 2712.86 | 969.80 | 30470.40 |  |  |  |
| 5915-58 | PEX5 | P50542 | 0 | 307.95 | 119.42 | 183.10 | 738.70 | 0.109 | 1.28E-01 | 2.21E-01 |
|  |  |  | 1 | 368.57 | 364.30 | 133.30 | 3990.20 |  |  |  |
| 3010-53 | TSLP | Q969D9 | 0 | 474.09 | 161.58 | 262.10 | 1204.40 | 0.102 | 1.29E-01 | 2.22E-01 |
|  |  |  | 1 | 574.81 | 865.90 | 259.50 | 13385.50 |  |  |  |
| 3059-50 | TNFSF13B | Q9Y275 | 0 | 1612.94 | 717.73 | 832.40 | 6033.20 | 0.095 | 1.29E-01 | 2.22E-01 |
|  |  |  | 1 | 1780.21 | 1142.30 | 444.00 | 14340.10 |  |  |  |
| 4392-54 | FGF12 | P61328 | 0 | 233.38 | 34.69 | 175.60 | 355.90 | 0.054 | 1.31E-01 | 2.26E-01 |
|  |  |  | 1 | 249.12 | 99.94 | 165.30 | 1224.70 |  |  |  |
| 3457-57 | POSTN | Q15063 | 0 | 6674.94 | 1737.72 | 3478.30 | 14962.60 | -0.079 | 1.32E-01 | 2.26E-01 |
|  |  |  | 1 | 6467.71 | 2196.98 | 1948.60 | 16562.30 |  |  |  |
| 4423-77 | BCL2L1 | Q07817 | 0 | 2055.42 | 1121.00 | 705.50 | 7437.50 | -0.137 | 1.32E-01 | 2.26E-01 |
|  |  |  | 1 | 2083.62 | 3249.99 | 418.40 | 55191.50 |  |  |  |
| 10367-62 | IL12A IL12B | P29459 P29460 | 0 | 377.67 | 370.37 | 222.10 | 3743.80 | 0.111 | 1.32E-01 | 2.27E-01 |
|  |  |  | 1 | 435.97 | 518.71 | 209.60 | 5061.80 |  |  |  |
| 2838-53 | MMP17 | Q9ULZ9 | 0 | 1054.45 | 266.08 | 695.00 | 2130.60 | 0.072 | 1.34E-01 | 2.29E-01 |
|  |  |  | 1 | 1140.22 | 468.03 | 664.70 | 3786.90 |  |  |  |
| 3875-62 | ARID3A | Q99856 | 0 | 818.87 | 584.06 | 322.20 | 3924.80 | 0.133 | 1.34E-01 | 2.29E-01 |
|  |  |  | 1 | 918.69 | 910.54 | 316.40 | 13501.20 |  |  |  |
| 14135-3 | RXFP1 | Q9HBX9 | 0 | 469.42 | 353.56 | 317.40 | 3864.50 | 0.076 | 1.34E-01 | 2.29E-01 |
|  |  |  | 1 | 500.10 | 359.19 | 295.90 | 4174.40 |  |  |  |
| 10344-334 | IL10RA | Q13651 | 0 | 1402.38 | 199.25 | 975.10 | 2039.80 | -0.043 | 1.35E-01 | 2.30E-01 |
|  |  |  | 1 | 1372.98 | 310.13 | 787.80 | 4776.50 |  |  |  |
| 3422-4 | CDC2 CCNB1 | P06493 P14635 | 0 | 773.94 | 159.03 | 547.30 | 1318.80 | 0.077 | 1.35E-01 | 2.30E-01 |
|  |  |  | 1 | 901.33 | 1177.05 | 496.40 | 18478.60 |  |  |  |
| 3374-49 | HCK | P08631 | 0 | 224.81 | 85.51 | 156.80 | 775.70 | -0.059 | 1.36E-01 | 2.31E-01 |
|  |  |  | 1 | 215.14 | 72.80 | 131.40 | 863.10 |  |  |  |
| 3052-8 | FASLG | P48023 | 0 | 946.96 | 2977.31 | 295.80 | 30274.70 | -0.139 | 1.37E-01 | 2.32E-01 |
|  |  |  | 1 | 760.45 | 1376.42 | 238.80 | 19071.70 |  |  |  |
| 5129-12 | SCARF1 | Q14162 | 0 | 9553.88 | 2431.40 | 5129.20 | 16773.90 | 0.076 | 1.37E-01 | 2.32E-01 |
|  |  |  | 1 | 10312.31 | 3611.78 | 4710.10 | 27685.30 |  |  |  |
| 10363-13 | SMAD3 | P84022 | 0 | 23545.23 | 13630.40 | 8347.90 | 60556.40 | -0.162 | 1.37E-01 | 2.32E-01 |
|  |  |  | 1 | 22240.61 | 13366.00 | 4216.70 | 60626.20 |  |  |  |
| 5798-3 | BID | P55957 | 0 | 2340.52 | 1393.47 | 847.90 | 7777.60 | 0.146 | 1.38E-01 | 2.33E-01 |
|  |  |  | 1 | 2751.29 | 2322.04 | 737.90 | 29835.60 |  |  |  |
| 2969-11 | ACVRL1 | P37023 | 0 | 1972.89 | 3070.52 | 1091.90 | 32169.60 | 0.104 | 1.40E-01 | 2.37E-01 |
|  |  |  | 1 | 2696.35 | 12767.25 | 1128.20 | 221007.30 |  |  |  |
| 9169-14 | SUMO3 | P55854 | 0 | 4230.06 | 2810.14 | 1015.80 | 11530.40 | 0.176 | 1.42E-01 | 2.38E-01 |
|  |  |  | 1 | 5165.59 | 4076.98 | 932.20 | 25107.20 |  |  |  |
| 5508-62 | CTSD | P07339 | 0 | 982.37 | 412.77 | 535.20 | 3418.90 | 0.096 | 1.41E-01 | 2.38E-01 |
|  |  |  | 1 | 1128.79 | 1297.43 | 519.20 | 20691.80 |  |  |  |
| 3473-78 | MPL | P40238 | 0 | 296.69 | 561.12 | 166.20 | 5750.70 | -0.082 | 1.42E-01 | 2.38E-01 |
|  |  |  | 1 | 241.12 | 158.19 | 149.20 | 2280.20 |  |  |  |
| 4487-1 | FGF7 | P21781 | 0 | 2111.72 | 735.08 | 1199.80 | 3865.80 | 0.100 | 1.44E-01 | 2.41E-01 |
|  |  |  | 1 | 2514.38 | 3890.02 | 914.70 | 66607.10 |  |  |  |
| 2917-3 | TNFSF11 | O14788 | 0 | 712.79 | 1218.37 | 330.90 | 12655.00 | -0.109 | 1.45E-01 | 2.43E-01 |
|  |  |  | 1 | 728.39 | 2227.81 | 207.90 | 38433.20 |  |  |  |
| 4160-49 | MMP2 | P08253 | 0 | 8474.34 | 1771.95 | 5501.80 | 14186.10 | -0.060 | 1.46E-01 | 2.44E-01 |
|  |  |  | 1 | 8353.19 | 4883.77 | 1807.40 | 87474.30 |  |  |  |
| 3858-5 | ACP1 | P24666 | 0 | 6840.24 | 6707.47 | 966.60 | 34142.90 | 0.234 | 1.46E-01 | 2.45E-01 |
|  |  |  | 1 | 8295.56 | 8239.71 | 747.90 | 42750.80 |  |  |  |
| 4978-54 | DBNL | Q9UJU6 | 0 | 4446.56 | 3366.07 | 2038.10 | 31417.70 | 0.129 | 1.49E-01 | 2.49E-01 |
|  |  |  | 1 | 5030.36 | 3517.89 | 1597.80 | 32362.90 |  |  |  |
| 4719-58 | PDIA3 | P30101 | 0 | 4829.84 | 2358.82 | 2148.70 | 11005.10 | 0.131 | 1.51E-01 | 2.52E-01 |
|  |  |  | 1 | 5665.75 | 3580.52 | 1774.60 | 22245.50 |  |  |  |
| 4540-11 | CBX5 | P45973 | 0 | 448.25 | 60.76 | 325.60 | 669.90 | -0.043 | 1.51E-01 | 2.52E-01 |
|  |  |  | 1 | 440.59 | 107.17 | 274.10 | 1498.00 |  |  |  |
| 4545-53 | DNAJC19 | Q96DA6 | 0 | 969.23 | 1606.74 | 554.90 | 15999.10 | 0.098 | 1.52E-01 | 2.53E-01 |
|  |  |  | 1 | 1184.73 | 5662.22 | 523.50 | 98794.10 |  |  |  |
| 3815-14 | IL12RB2 | Q99665 | 0 | 1208.46 | 1132.40 | 676.80 | 11486.40 | 0.125 | 1.53E-01 | 2.54E-01 |
|  |  |  | 1 | 1833.76 | 5633.40 | 629.30 | 65234.00 |  |  |  |
| 5346-24 | CPNE1 | Q99829 | 0 | 5515.86 | 4589.85 | 227.60 | 23446.30 | 0.243 | 1.53E-01 | 2.55E-01 |
|  |  |  | 1 | 7055.98 | 7129.48 | 207.90 | 57379.30 |  |  |  |
| 4389-2 | DHH | O43323 | 0 | 903.12 | 487.07 | 284.50 | 2280.70 | 0.146 | 1.54E-01 | 2.55E-01 |
|  |  |  | 1 | 1359.18 | 5452.74 | 305.50 | 94056.00 |  |  |  |
| 5228-25 | KIF23 | Q02241 | 0 | 1380.68 | 735.70 | 540.20 | 3777.00 | 0.142 | 1.54E-01 | 2.55E-01 |
|  |  |  | 1 | 1711.31 | 2028.12 | 424.90 | 30131.50 |  |  |  |
| 2961-1 | PROC | P04070 | 0 | 1595.65 | 271.58 | 853.70 | 2236.40 | -0.057 | 1.55E-01 | 2.56E-01 |
|  |  |  | 1 | 1628.87 | 1895.09 | 683.30 | 33954.20 |  |  |  |
| 2985-35 | CXCL1 | P09341 | 0 | 5252.17 | 4077.30 | 2208.70 | 25628.30 | 0.121 | 1.57E-01 | 2.59E-01 |
|  |  |  | 1 | 5509.50 | 3575.69 | 1827.50 | 22386.80 |  |  |  |
| 5006-71 | MAPK13 | O15264 | 0 | 738.70 | 699.21 | 410.70 | 7112.30 | 0.096 | 1.57E-01 | 2.59E-01 |
|  |  |  | 1 | 847.60 | 1652.74 | 250.80 | 28344.60 |  |  |  |
| 4913-78 | CCL16 | O15467 | 0 | 38729.70 | 10839.31 | 15113.50 | 57675.70 | -0.084 | 1.57E-01 | 2.59E-01 |
|  |  |  | 1 | 37122.44 | 12894.91 | 3867.00 | 102591.10 |  |  |  |
| 5077-28 | EPHB2 | P29323 | 0 | 21098.36 | 4978.59 | 11086.70 | 34427.00 | -0.073 | 1.59E-01 | 2.62E-01 |
|  |  |  | 1 | 20503.04 | 6281.49 | 5475.40 | 48623.90 |  |  |  |
| 5467-15 | HSP90AB1 | P08238 | 0 | 54160.40 | 24461.43 | 22122.90 | 114788.10 | 0.116 | 1.59E-01 | 2.62E-01 |
|  |  |  | 1 | 60441.31 | 29642.70 | 17452.60 | 164694.00 |  |  |  |
| 2741-22 | SIGLEC6 | O43699 | 0 | 270.13 | 72.44 | 168.50 | 484.70 | -0.065 | 1.61E-01 | 2.65E-01 |
|  |  |  | 1 | 261.64 | 90.15 | 127.70 | 804.20 |  |  |  |
| 2900-53 | CCL14 | Q16627 | 0 | 6356.31 | 2381.63 | 2492.70 | 13739.90 | 0.096 | 1.62E-01 | 2.66E-01 |
|  |  |  | 1 | 6898.89 | 2929.14 | 1811.30 | 17825.10 |  |  |  |
| 3065-65 | FGF5 | P12034 | 0 | 772.46 | 1019.45 | 416.80 | 9622.10 | 0.120 | 1.63E-01 | 2.67E-01 |
|  |  |  | 1 | 1084.29 | 4421.73 | 376.40 | 68563.30 |  |  |  |
| 3202-28 | KREMEN2 | Q8NCW0 | 0 | 4161.66 | 2018.31 | 1616.60 | 9701.10 | 0.119 | 1.63E-01 | 2.67E-01 |
|  |  |  | 1 | 4685.13 | 2766.70 | 1420.50 | 30404.20 |  |  |  |
| 5014-49 | NCK1 | P16333 | 0 | 1496.38 | 1162.40 | 760.00 | 11344.50 | 0.116 | 1.64E-01 | 2.68E-01 |
|  |  |  | 1 | 1868.81 | 3067.37 | 684.50 | 35148.40 |  |  |  |
| 3003-29 | NCR3 | O14931 | 0 | 599.42 | 110.65 | 378.50 | 1062.90 | -0.080 | 1.64E-01 | 2.68E-01 |
|  |  |  | 1 | 803.80 | 3940.22 | 302.60 | 68599.80 |  |  |  |
| 14143-8 | HIST2H2BE | Q16778 | 0 | 14833.26 | 8007.08 | 2266.40 | 46683.10 | 0.139 | 1.66E-01 | 2.71E-01 |
|  |  |  | 1 | 17431.53 | 11644.18 | 1261.30 | 71575.00 |  |  |  |
| 4282-3 | RAN | P62826 | 0 | 12232.15 | 7385.45 | 1006.90 | 31428.20 | -0.215 | 1.66E-01 | 2.71E-01 |
|  |  |  | 1 | 13343.33 | 16401.70 | 333.20 | 240111.90 |  |  |  |
| 2805-6 | ACE2 | Q9BYF1 | 0 | 657.90 | 222.45 | 382.30 | 2204.60 | 0.074 | 1.68E-01 | 2.72E-01 |
|  |  |  | 1 | 719.17 | 430.30 | 314.30 | 5638.40 |  |  |  |
| 2247-20 | PROK1 | P58294 | 0 | 223.21 | 205.95 | 137.80 | 1647.50 | 0.070 | 1.67E-01 | 2.72E-01 |
|  |  |  | 1 | 222.40 | 104.45 | 118.40 | 1057.70 |  |  |  |
| 3627-71 | MMEL1 | Q495T6 | 0 | 1331.70 | 906.34 | 953.30 | 9873.20 | 0.067 | 1.68E-01 | 2.72E-01 |
|  |  |  | 1 | 1434.45 | 1699.16 | 810.70 | 29401.10 |  |  |  |
| 5002-76 | MMP14 | P50281 | 0 | 1655.50 | 3626.97 | 567.00 | 36541.80 | 0.154 | 1.69E-01 | 2.74E-01 |
|  |  |  | 1 | 2427.70 | 7445.18 | 446.90 | 71750.40 |  |  |  |
| 4929-55 | SHBG | P04278 | 0 | 3801.70 | 2080.75 | 1158.00 | 16083.70 | 0.101 | 1.70E-01 | 2.75E-01 |
|  |  |  | 1 | 3976.37 | 1817.67 | 1184.40 | 16415.80 |  |  |  |
| 4922-13 | CCL19 | Q99731 | 0 | 577.77 | 242.93 | 276.90 | 1797.30 | 0.112 | 1.73E-01 | 2.79E-01 |
|  |  |  | 1 | 736.20 | 1060.79 | 141.60 | 10924.80 |  |  |  |
| 4874-3 | ANG | P03950 | 0 | 3833.79 | 948.95 | 1469.60 | 7467.90 | -0.059 | 1.73E-01 | 2.79E-01 |
|  |  |  | 1 | 3691.97 | 906.08 | 563.70 | 8912.40 |  |  |  |
| 4883-56 | INS | P01308 | 0 | 268.83 | 330.91 | 81.30 | 3083.00 | -0.138 | 1.73E-01 | 2.79E-01 |
|  |  |  | 1 | 265.51 | 732.11 | 64.60 | 12498.80 |  |  |  |
| 5259-2 | MAP3K7 TAB1 | O43318 Q15750 | 0 | 2784.64 | 1881.24 | 1318.20 | 9550.10 | 0.113 | 1.74E-01 | 2.80E-01 |
|  |  |  | 1 | 2984.71 | 1828.58 | 1281.70 | 11907.70 |  |  |  |
| 9190-7 | CD63 | P08962 | 0 | 490.38 | 124.69 | 351.60 | 1247.90 | 0.068 | 1.74E-01 | 2.80E-01 |
|  |  |  | 1 | 543.32 | 360.44 | 323.60 | 4269.50 |  |  |  |
| 2443-10 | FGF8 | P55075 | 0 | 930.15 | 256.26 | 662.40 | 2842.20 | 0.058 | 1.74E-01 | 2.80E-01 |
|  |  |  | 1 | 996.98 | 523.22 | 631.60 | 8178.90 |  |  |  |
| 2796-62 | FGA FGB FGG | P02671 P02675 P02679 | 0 | 169548.40 | 23984.58 | 131469.60 | 237231.40 | 0.089 | 1.76E-01 | 2.82E-01 |
|  |  |  | 1 | 185245.94 | 27067.38 | 803.80 | 266928.60 |  |  |  |
| 4471-50 | TGM3 | Q08188 | 0 | 702.35 | 1161.32 | 99.60 | 6319.70 | 0.239 | 1.76E-01 | 2.83E-01 |
|  |  |  | 1 | 1124.33 | 2406.33 | 85.30 | 23489.10 |  |  |  |
| 2771-35 | IGFBP1 | P08833 | 0 | 722.52 | 705.38 | 121.30 | 3658.50 | 0.175 | 1.79E-01 | 2.87E-01 |
|  |  |  | 1 | 811.56 | 834.70 | 120.90 | 6903.70 |  |  |  |
| 14146-92 | HIST1H3A | P68431 | 0 | 1763.70 | 1145.41 | 584.50 | 9174.20 | 0.101 | 1.80E-01 | 2.87E-01 |
|  |  |  | 1 | 1918.54 | 1263.59 | 537.70 | 9593.10 |  |  |  |
| 4151-6 | PLG | P00747 | 0 | 4498.83 | 678.82 | 2668.00 | 7006.50 | -0.037 | 1.80E-01 | 2.88E-01 |
|  |  |  | 1 | 4403.40 | 838.51 | 2663.40 | 11837.00 |  |  |  |
| 3413-50 | BCL2A1 | Q16548 | 0 | 918.33 | 164.62 | 658.30 | 1374.80 | 0.064 | 1.82E-01 | 2.89E-01 |
|  |  |  | 1 | 1033.27 | 1007.51 | 606.00 | 16480.60 |  |  |  |
| 2635-61 | LAYN | Q6UX15 | 0 | 1705.09 | 305.87 | 1062.80 | 2341.30 | -0.060 | 1.82E-01 | 2.89E-01 |
|  |  |  | 1 | 1697.33 | 846.68 | 698.30 | 13861.70 |  |  |  |
| 2602-2 | ANGPT2 | O15123 | 0 | 198.86 | 63.19 | 121.70 | 471.40 | -0.073 | 1.82E-01 | 2.89E-01 |
|  |  |  | 1 | 203.95 | 287.83 | 101.20 | 5013.10 |  |  |  |
| 2773-50 | IL10 | P22301 | 0 | 259.65 | 254.77 | 164.30 | 2615.60 | -0.079 | 1.82E-01 | 2.89E-01 |
|  |  |  | 1 | 244.77 | 230.70 | 147.60 | 3149.50 |  |  |  |
| 5489-18 | STIP1 | P31948 | 0 | 12472.05 | 7812.57 | 3339.30 | 36115.30 | 0.154 | 1.84E-01 | 2.92E-01 |
|  |  |  | 1 | 14968.69 | 11098.39 | 2956.50 | 88447.70 |  |  |  |
| 3874-8 | UBE2L3 | P68036 | 0 | 2246.38 | 1423.40 | 615.00 | 6255.90 | 0.145 | 1.86E-01 | 2.94E-01 |
|  |  |  | 1 | 2636.08 | 2288.29 | 566.50 | 29780.10 |  |  |  |
| 2625-53 | HSP90AA1 HSP90AB1 | P07900 P08238 | 0 | 11512.69 | 7420.55 | 4038.30 | 32288.20 | 0.152 | 1.87E-01 | 2.95E-01 |
|  |  |  | 1 | 13956.20 | 11143.48 | 2625.30 | 76708.00 |  |  |  |
| 5542-22 | NRP1 | O14786 | 0 | 2509.40 | 426.47 | 1382.90 | 3618.70 | -0.047 | 1.87E-01 | 2.95E-01 |
|  |  |  | 1 | 2450.23 | 519.24 | 1125.30 | 4025.30 |  |  |  |
| 3397-7 | PTPN11 | Q06124 | 0 | 7274.51 | 4018.24 | 2339.00 | 18857.60 | -0.129 | 1.87E-01 | 2.95E-01 |
|  |  |  | 1 | 6966.79 | 4312.89 | 788.60 | 23666.80 |  |  |  |
| 4547-59 | FLRT1 | Q9NZU1 | 0 | 1190.80 | 464.53 | 567.80 | 2435.20 | 0.096 | 1.88E-01 | 2.97E-01 |
|  |  |  | 1 | 1348.01 | 1045.05 | 529.70 | 16243.50 |  |  |  |
| 3340-53 | THBS4 | P35443 | 0 | 510.54 | 128.93 | 244.80 | 927.60 | -0.072 | 1.91E-01 | 3.01E-01 |
|  |  |  | 1 | 510.93 | 322.69 | 202.30 | 4193.10 |  |  |  |
| 3593-72 | CASP3 | P42574 | 0 | 13364.43 | 10566.92 | 700.00 | 58150.60 | -0.243 | 1.92E-01 | 3.02E-01 |
|  |  |  | 1 | 14433.82 | 12677.21 | 302.60 | 64293.00 |  |  |  |
| 2642-4 | PAFAH1B2 | P68402 | 0 | 7492.84 | 3672.30 | 2649.30 | 15782.00 | 0.110 | 1.92E-01 | 3.02E-01 |
|  |  |  | 1 | 8239.40 | 4492.01 | 2245.90 | 46944.70 |  |  |  |
| 3310-62 | FCGR2B | P31994 | 0 | 2816.23 | 1928.67 | 658.80 | 8994.40 | 0.150 | 1.94E-01 | 3.03E-01 |
|  |  |  | 1 | 3222.51 | 2413.29 | 707.20 | 14491.80 |  |  |  |
| 3865-53 | RPS6KA5 | O75582 | 0 | 312.20 | 428.51 | 119.60 | 4356.40 | 0.114 | 1.94E-01 | 3.03E-01 |
|  |  |  | 1 | 323.05 | 321.41 | 100.40 | 5065.90 |  |  |  |
| 5459-33 | CST1 | P01037 | 0 | 1681.70 | 834.33 | 332.30 | 5518.60 | -0.106 | 1.94E-01 | 3.03E-01 |
|  |  |  | 1 | 1606.85 | 929.84 | 410.20 | 7144.10 |  |  |  |
| 5885-55 | EIF4H | Q15056 | 0 | 7575.46 | 7201.55 | 193.10 | 44490.70 | -0.245 | 1.95E-01 | 3.05E-01 |
|  |  |  | 1 | 7665.61 | 7365.46 | 135.70 | 48067.20 |  |  |  |
| 4258-15 | PA2G4 | Q9UQ80 | 0 | 2818.41 | 1869.41 | 431.70 | 7810.40 | 0.173 | 1.96E-01 | 3.05E-01 |
|  |  |  | 1 | 3707.62 | 4191.43 | 426.90 | 57653.70 |  |  |  |
| 2833-20 | KLK4 | Q9Y5K2 | 0 | 409.70 | 561.23 | 275.00 | 5896.60 | 0.077 | 1.98E-01 | 3.09E-01 |
|  |  |  | 1 | 442.52 | 747.36 | 264.50 | 11914.90 |  |  |  |
| 8463-2 | SOD3 | P08294 | 0 | 779.66 | 671.13 | 366.90 | 5293.20 | -0.091 | 2.00E-01 | 3.12E-01 |
|  |  |  | 1 | 761.55 | 1190.22 | 287.10 | 18880.70 |  |  |  |
| 3516-60 | CXCL12 | P48061 | 0 | 5654.26 | 1273.40 | 1957.80 | 8701.70 | 0.058 | 2.01E-01 | 3.12E-01 |
|  |  |  | 1 | 5916.34 | 1505.72 | 1929.00 | 11410.20 |  |  |  |
| 4979-34 | DPT | Q07507 | 0 | 5868.75 | 1515.28 | 3398.00 | 12249.80 | -0.063 | 2.02E-01 | 3.13E-01 |
|  |  |  | 1 | 5762.66 | 2924.36 | 1670.90 | 48710.30 |  |  |  |
| 3684-78 | MSR1 | P21757 | 0 | 1032.11 | 258.37 | 745.20 | 2140.10 | 0.053 | 2.02E-01 | 3.14E-01 |
|  |  |  | 1 | 1090.81 | 476.01 | 616.90 | 7149.80 |  |  |  |
| 3324-51 | LY9 | Q9HBG7 | 0 | 2312.15 | 797.81 | 320.80 | 4179.90 | -0.138 | 2.04E-01 | 3.16E-01 |
|  |  |  | 1 | 2336.17 | 1215.34 | 100.10 | 7110.70 |  |  |  |
| 3081-70 | ULBP1 | Q9BZM6 | 0 | 638.30 | 704.85 | 304.70 | 5271.10 | 0.099 | 2.05E-01 | 3.17E-01 |
|  |  |  | 1 | 646.33 | 472.51 | 314.80 | 4161.20 |  |  |  |
| 5347-59 | CCNB1 | P14635 | 0 | 594.50 | 473.17 | 409.30 | 4582.30 | 0.081 | 2.07E-01 | 3.19E-01 |
|  |  |  | 1 | 659.97 | 728.12 | 335.30 | 9259.60 |  |  |  |
| 3359-11 | CDK8 CCNC | P49336 P24863 | 0 | 1956.00 | 1002.56 | 951.00 | 9551.80 | -0.084 | 2.08E-01 | 3.21E-01 |
|  |  |  | 1 | 1877.13 | 999.94 | 820.40 | 12844.20 |  |  |  |
| 3485-28 | B2M | P61769 | 0 | 2272.06 | 648.85 | 966.00 | 5100.60 | 0.056 | 2.09E-01 | 3.23E-01 |
|  |  |  | 1 | 2372.20 | 723.41 | 1315.00 | 5550.50 |  |  |  |
| 5007-1 | MAPK14 | Q16539 | 0 | 3146.07 | 2061.31 | 977.70 | 9138.70 | 0.147 | 2.13E-01 | 3.28E-01 |
|  |  |  | 1 | 3792.36 | 2875.27 | 776.60 | 16297.70 |  |  |  |
| 3600-2 | CHIT1 | Q13231 | 0 | 3042.49 | 1706.02 | 528.30 | 10652.40 | 0.122 | 2.17E-01 | 3.34E-01 |
|  |  |  | 1 | 3485.38 | 3653.38 | 436.80 | 54466.70 |  |  |  |
| 4132-27 | FST | P19883 | 0 | 1041.88 | 307.40 | 636.10 | 2233.60 | 0.072 | 2.18E-01 | 3.35E-01 |
|  |  |  | 1 | 1153.26 | 776.49 | 542.60 | 10796.80 |  |  |  |
| 13125-45 | VTN | P04004 | 0 | 63843.17 | 21805.97 | 21559.60 | 106540.20 | -0.088 | 2.18E-01 | 3.35E-01 |
|  |  |  | 1 | 60647.29 | 21805.69 | 9270.90 | 119770.80 |  |  |  |
| 2891-1 | CD22 | P20273 | 0 | 1014.78 | 231.37 | 726.50 | 2234.60 | 0.049 | 2.19E-01 | 3.35E-01 |
|  |  |  | 1 | 1068.96 | 401.41 | 691.10 | 5221.70 |  |  |  |
| 5494-52 | SNRPF | P62306 | 0 | 3150.48 | 396.03 | 2290.40 | 4375.90 | -0.048 | 2.22E-01 | 3.40E-01 |
|  |  |  | 1 | 3253.63 | 3350.62 | 1616.00 | 58527.50 |  |  |  |
| 5262-57 | BCAR3 | O75815 | 0 | 1862.96 | 733.16 | 1190.90 | 6701.00 | -0.051 | 2.23E-01 | 3.41E-01 |
|  |  |  | 1 | 1797.84 | 789.77 | 1034.20 | 11288.30 |  |  |  |
| 5272-55 | SHC1 | P29353 | 0 | 14753.34 | 9825.67 | 4307.80 | 45034.70 | 0.134 | 2.28E-01 | 3.49E-01 |
|  |  |  | 1 | 16907.18 | 11079.55 | 2858.00 | 53550.50 |  |  |  |
| 9213-24 | FTCD | O95954 | 0 | 19769.96 | 26665.94 | 496.70 | 151510.80 | 0.218 | 2.28E-01 | 3.49E-01 |
|  |  |  | 1 | 21641.64 | 34968.42 | 531.60 | 437677.10 |  |  |  |
| 14139-16 | NRG4 | Q8WWG1 | 0 | 1002.31 | 549.68 | 645.20 | 5122.70 | -0.058 | 2.30E-01 | 3.51E-01 |
|  |  |  | 1 | 940.93 | 319.73 | 434.80 | 3278.50 |  |  |  |
| 4566-24 | RSPO2 | Q6UXX9 | 0 | 617.33 | 921.98 | 328.60 | 8289.10 | 0.089 | 2.32E-01 | 3.53E-01 |
|  |  |  | 1 | 865.27 | 5719.12 | 315.60 | 99515.50 |  |  |  |
| 3845-51 | DYNLRB1 | Q9NP97 | 0 | 3423.85 | 3103.56 | 466.80 | 20693.20 | 0.184 | 2.34E-01 | 3.56E-01 |
|  |  |  | 1 | 4565.05 | 4406.79 | 473.70 | 22098.40 |  |  |  |
| 3903-49 | SNX4 | O95219 | 0 | 17345.19 | 12814.06 | 2503.80 | 47474.00 | -0.196 | 2.35E-01 | 3.57E-01 |
|  |  |  | 1 | 17907.12 | 14046.69 | 1425.70 | 57380.30 |  |  |  |
| 5349-69 | DLL1 | O00548 | 0 | 2419.47 | 464.59 | 1573.30 | 3634.60 | -0.050 | 2.37E-01 | 3.59E-01 |
|  |  |  | 1 | 2414.48 | 1286.57 | 1143.20 | 21634.20 |  |  |  |
| 5807-77 | CD70 | P32970 | 0 | 341.32 | 153.68 | 246.70 | 1301.00 | 0.078 | 2.38E-01 | 3.61E-01 |
|  |  |  | 1 | 403.67 | 490.75 | 197.30 | 5691.80 |  |  |  |
| 5004-69 | MAPK11 | Q15759 | 0 | 806.45 | 1419.99 | 418.80 | 14737.60 | -0.083 | 2.38E-01 | 3.61E-01 |
|  |  |  | 1 | 720.99 | 729.30 | 207.10 | 10464.00 |  |  |  |
| 4134-4 | HBEGF | Q99075 | 0 | 399.22 | 155.90 | 255.50 | 1489.90 | 0.065 | 2.40E-01 | 3.64E-01 |
|  |  |  | 1 | 458.92 | 739.50 | 218.70 | 12780.30 |  |  |  |
| 5102-55 | MICB | Q29980 | 0 | 1159.04 | 427.47 | 365.30 | 2874.20 | -0.082 | 2.46E-01 | 3.71E-01 |
|  |  |  | 1 | 1145.11 | 667.99 | 289.60 | 8081.90 |  |  |  |
| 2623-54 | Human-virus | P03129 | 0 | 532.15 | 251.31 | 276.70 | 2185.40 | 0.066 | 2.47E-01 | 3.72E-01 |
|  |  |  | 1 | 552.45 | 220.65 | 248.10 | 1683.60 |  |  |  |
| 13114-50 | LUM | P51884 | 0 | 1684.46 | 259.36 | 1076.30 | 2359.90 | 0.037 | 2.49E-01 | 3.75E-01 |
|  |  |  | 1 | 1742.27 | 352.16 | 927.70 | 3208.60 |  |  |  |
| 4224-7 | SYNCRIP | O60506 | 0 | 2832.09 | 517.44 | 1732.20 | 4560.10 | 0.056 | 2.49E-01 | 3.75E-01 |
|  |  |  | 1 | 3058.21 | 1087.03 | 873.50 | 11207.70 |  |  |  |
| 5897-58 | GRP | P07492 | 0 | 456.68 | 233.11 | 323.20 | 2344.00 | 0.085 | 2.52E-01 | 3.80E-01 |
|  |  |  | 1 | 657.83 | 1977.69 | 261.30 | 24466.00 |  |  |  |
| 4557-61 | KIRREL3 | Q8IZU9 | 0 | 346.53 | 39.91 | 268.80 | 538.70 | -0.049 | 2.54E-01 | 3.81E-01 |
|  |  |  | 1 | 377.97 | 699.52 | 227.00 | 12299.00 |  |  |  |
| 2607-54 | CRLF1 CLCF1 | O75462 Q9UBD9 | 0 | 540.36 | 158.85 | 370.40 | 1574.90 | -0.062 | 2.54E-01 | 3.81E-01 |
|  |  |  | 1 | 569.59 | 727.66 | 289.50 | 11901.50 |  |  |  |
| 3399-31 | STAB2 | Q8WWQ8 | 0 | 940.15 | 130.48 | 707.90 | 1263.40 | 0.038 | 2.55E-01 | 3.82E-01 |
|  |  |  | 1 | 982.18 | 269.95 | 446.50 | 3920.70 |  |  |  |
| 14129-1 | IFNA7 | P01567 | 0 | 341.46 | 179.54 | 222.20 | 1491.90 | 0.088 | 2.57E-01 | 3.83E-01 |
|  |  |  | 1 | 437.85 | 789.98 | 174.10 | 10960.70 |  |  |  |
| 3447-64 | CXCL8 | P10145 | 0 | 1060.67 | 363.81 | 755.20 | 3500.90 | 0.063 | 2.57E-01 | 3.83E-01 |
|  |  |  | 1 | 1174.42 | 804.04 | 638.20 | 9761.40 |  |  |  |
| 2950-57 | IGFBP4 | P22692 | 0 | 7592.45 | 1782.65 | 5297.10 | 20218.90 | -0.036 | 2.57E-01 | 3.83E-01 |
|  |  |  | 1 | 7396.57 | 1607.89 | 4691.30 | 22461.40 |  |  |  |
| 3022-4 | CTLA4 | P16410 | 0 | 178.41 | 329.23 | 70.90 | 3035.80 | -0.115 | 2.57E-01 | 3.83E-01 |
|  |  |  | 1 | 181.79 | 531.41 | 45.80 | 6561.80 |  |  |  |
| 5882-34 | EEF1B2 | P24534 | 0 | 898.02 | 510.12 | 327.70 | 3485.50 | 0.104 | 2.59E-01 | 3.85E-01 |
|  |  |  | 1 | 1037.14 | 1104.26 | 318.70 | 16940.40 |  |  |  |
| 4238-4 | LIN7B | Q9HAP6 | 0 | 473.34 | 56.39 | 342.20 | 628.60 | 0.041 | 2.59E-01 | 3.85E-01 |
|  |  |  | 1 | 506.05 | 281.26 | 225.00 | 4059.50 |  |  |  |
| 2999-6 | LSAMP | Q13449 | 0 | 1752.85 | 350.40 | 769.10 | 2562.80 | 0.041 | 2.62E-01 | 3.90E-01 |
|  |  |  | 1 | 1808.62 | 379.02 | 520.50 | 3269.40 |  |  |  |
| 5252-33 | PDE11A | Q9HCR9 | 0 | 3789.70 | 1651.95 | 1858.70 | 8419.00 | 0.094 | 2.64E-01 | 3.91E-01 |
|  |  |  | 1 | 4361.01 | 2709.74 | 1360.40 | 19676.20 |  |  |  |
| 3055-54 | IL4R | P24394 | 0 | 286.20 | 168.91 | 158.00 | 1364.80 | -0.068 | 2.64E-01 | 3.91E-01 |
|  |  |  | 1 | 277.16 | 214.41 | 114.80 | 2963.20 |  |  |  |
| 5441-67 | TNNI3 | P19429 | 0 | 708.51 | 419.97 | 438.10 | 3875.10 | -0.071 | 2.65E-01 | 3.92E-01 |
|  |  |  | 1 | 720.42 | 790.85 | 295.30 | 10692.90 |  |  |  |
| 4138-25 | IL20 | Q9NYY1 | 0 | 406.17 | 128.47 | 327.00 | 1610.80 | 0.034 | 2.66E-01 | 3.94E-01 |
|  |  |  | 1 | 415.20 | 100.70 | 263.40 | 1514.80 |  |  |  |
| 2972-57 | BMP7 | P18075 | 0 | 814.00 | 275.94 | 493.80 | 1836.60 | -0.058 | 2.67E-01 | 3.94E-01 |
|  |  |  | 1 | 800.95 | 434.89 | 246.00 | 5666.30 |  |  |  |
| 4851-25 | IL1A | P01583 | 0 | 741.17 | 340.89 | 491.70 | 2467.40 | 0.070 | 2.71E-01 | 4.00E-01 |
|  |  |  | 1 | 828.66 | 669.23 | 292.20 | 6491.40 |  |  |  |
| 2708-54 | TNFSF18 | Q9UNG2 | 0 | 703.91 | 198.37 | 548.20 | 2444.80 | -0.042 | 2.71E-01 | 4.00E-01 |
|  |  |  | 1 | 710.36 | 595.85 | 357.40 | 10562.20 |  |  |  |
| 5230-99 | HMGCR | P04035 | 0 | 485.72 | 389.71 | 283.40 | 3414.30 | 0.086 | 2.73E-01 | 4.02E-01 |
|  |  |  | 1 | 637.07 | 1619.28 | 268.10 | 21470.80 |  |  |  |
| 3362-61 | CHRDL1 | Q9BU40 | 0 | 2954.11 | 625.74 | 1763.60 | 4455.60 | 0.050 | 2.73E-01 | 4.02E-01 |
|  |  |  | 1 | 3117.54 | 914.73 | 946.90 | 7978.90 |  |  |  |
| 3389-7 | SERPINA5 | P05154 | 0 | 100873.01 | 19785.46 | 442.80 | 135064.40 | -0.064 | 2.74E-01 | 4.03E-01 |
|  |  |  | 1 | 92298.51 | 16053.78 | 7734.10 | 133865.90 |  |  |  |
| 5934-1 | FTH1 FTL | P02794 P02792 | 0 | 16772.83 | 12233.36 | 1095.50 | 57006.50 | 0.153 | 2.75E-01 | 4.04E-01 |
|  |  |  | 1 | 19710.46 | 16819.60 | 1136.80 | 128433.70 |  |  |  |
| 2754-50 | C3 | P01024 | 0 | 86914.32 | 49317.45 | 39258.40 | 462914.20 | -0.080 | 2.75E-01 | 4.04E-01 |
|  |  |  | 1 | 85338.26 | 46836.34 | 8300.10 | 354264.50 |  |  |  |
| 4553-65 | HDGFRP2 | Q7Z4V5 | 0 | 951.52 | 513.94 | 658.40 | 5185.80 | 0.065 | 2.77E-01 | 4.05E-01 |
|  |  |  | 1 | 1244.47 | 4769.71 | 642.70 | 83193.30 |  |  |  |
| 4140-3 | IL7 | P13232 | 0 | 174.46 | 26.53 | 143.10 | 311.10 | 0.050 | 2.77E-01 | 4.05E-01 |
|  |  |  | 1 | 195.22 | 165.54 | 126.70 | 2066.90 |  |  |  |
| 2201-17 | COL18A1 | P39060 | 0 | 77158.25 | 14647.36 | 44086.00 | 119228.80 | 0.041 | 2.76E-01 | 4.05E-01 |
|  |  |  | 1 | 79850.65 | 17246.51 | 14704.50 | 132056.90 |  |  |  |
| 5249-31 | STK17B | O94768 | 0 | 5773.51 | 1747.17 | 3540.30 | 18868.70 | 0.060 | 2.79E-01 | 4.07E-01 |
|  |  |  | 1 | 6380.30 | 3732.84 | 2442.20 | 32420.00 |  |  |  |
| 5223-59 | GCKR | Q14397 | 0 | 669.24 | 431.55 | 439.20 | 3418.20 | -0.053 | 2.81E-01 | 4.10E-01 |
|  |  |  | 1 | 623.66 | 321.31 | 397.80 | 4721.10 |  |  |  |
| 2870-29 | RAC1 | P63000 | 0 | 56550.12 | 36284.00 | 6269.30 | 152519.80 | -0.164 | 2.82E-01 | 4.11E-01 |
|  |  |  | 1 | 60280.27 | 44574.05 | 3920.80 | 192597.50 |  |  |  |
| 8465-52 | CTSH | P09668 | 0 | 1080.23 | 413.10 | 155.30 | 3047.10 | 0.092 | 2.83E-01 | 4.12E-01 |
|  |  |  | 1 | 1262.51 | 1410.92 | 164.60 | 23589.90 |  |  |  |
| 5462-62 | FCN3 | O75636 | 0 | 1233.41 | 1477.09 | 73.20 | 7311.20 | 0.263 | 2.84E-01 | 4.13E-01 |
|  |  |  | 1 | 1699.74 | 2204.24 | 45.50 | 14723.70 |  |  |  |
| 2714-78 | AIMP1 | Q12904 | 0 | 4385.67 | 1066.93 | 2787.70 | 9054.20 | -0.044 | 2.86E-01 | 4.15E-01 |
|  |  |  | 1 | 4302.12 | 1295.19 | 1631.80 | 12566.70 |  |  |  |
| 3122-6 | DIABLO | Q9NR28 | 0 | 5066.20 | 999.41 | 3447.60 | 12366.90 | -0.045 | 2.87E-01 | 4.17E-01 |
|  |  |  | 1 | 5096.00 | 2634.22 | 1522.70 | 41109.80 |  |  |  |
| 3827-22 | PAK6 | Q9NQU5 | 0 | 15000.26 | 12660.94 | 672.90 | 48891.50 | 0.206 | 2.94E-01 | 4.26E-01 |
|  |  |  | 1 | 20939.70 | 17710.81 | 433.60 | 81816.80 |  |  |  |
| 13116-25 | CD177 | Q8N6Q3 | 0 | 1123.45 | 1234.09 | 153.80 | 7392.10 | -0.126 | 2.96E-01 | 4.28E-01 |
|  |  |  | 1 | 1001.04 | 1097.28 | 120.30 | 11072.80 |  |  |  |
| 4141-79 | CXCL10 | P02778 | 0 | 3032.64 | 2382.84 | 892.10 | 21762.50 | 0.090 | 2.99E-01 | 4.32E-01 |
|  |  |  | 1 | 3581.69 | 6658.35 | 992.80 | 108075.70 |  |  |  |
| 2612-5 | EIF5 | P55010 | 0 | 1307.73 | 440.39 | 760.60 | 2818.20 | 0.060 | 3.00E-01 | 4.32E-01 |
|  |  |  | 1 | 1413.55 | 949.51 | 612.20 | 13026.60 |  |  |  |
| 2982-82 | LGALS4 | P56470 | 0 | 809.27 | 182.96 | 535.90 | 1501.60 | 0.044 | 3.00E-01 | 4.33E-01 |
|  |  |  | 1 | 855.86 | 409.59 | 409.10 | 5942.80 |  |  |  |
| 5307-12 | F9 | P00740 | 0 | 8969.10 | 1445.36 | 4474.90 | 12395.00 | 0.030 | 3.00E-01 | 4.33E-01 |
|  |  |  | 1 | 9137.95 | 1403.35 | 3092.40 | 14641.00 |  |  |  |
| 5250-53 | IMPDH2 | P12268 | 0 | 3212.65 | 982.97 | 1938.70 | 7128.40 | 0.059 | 3.04E-01 | 4.35E-01 |
|  |  |  | 1 | 3467.38 | 1518.71 | 1751.30 | 13051.20 |  |  |  |
| 2474-54 | APCS | P02743 | 0 | 42946.23 | 8208.10 | 17334.00 | 61366.30 | 0.033 | 3.03E-01 | 4.35E-01 |
|  |  |  | 1 | 43758.08 | 7767.07 | 20740.20 | 70759.20 |  |  |  |
| 5115-31 | RELT | Q969Z4 | 0 | 2623.35 | 716.34 | 1186.80 | 5278.80 | -0.056 | 3.04E-01 | 4.35E-01 |
|  |  |  | 1 | 2574.71 | 863.39 | 317.10 | 6053.40 |  |  |  |
| 3509-1 | CCL15 | Q16663 | 0 | 5331.86 | 2003.77 | 2702.40 | 14048.50 | -0.060 | 3.03E-01 | 4.35E-01 |
|  |  |  | 1 | 5178.19 | 2096.48 | 1936.00 | 17146.20 |  |  |  |
| 13123-3 | FLRT3 | Q9NZU0 | 0 | 4622.02 | 1446.93 | 797.20 | 8797.20 | -0.093 | 3.04E-01 | 4.35E-01 |
|  |  |  | 1 | 4642.92 | 1971.48 | 312.00 | 10255.60 |  |  |  |
| 3440-7 | GZMA | P12544 | 0 | 3601.17 | 1051.26 | 1435.10 | 7669.40 | -0.062 | 3.05E-01 | 4.36E-01 |
|  |  |  | 1 | 3565.44 | 1509.49 | 928.50 | 13090.90 |  |  |  |
| 10351-51 | IRF1 | P10914 | 0 | 4540.87 | 28368.00 | 1020.30 | 285071.50 | -0.092 | 3.07E-01 | 4.39E-01 |
|  |  |  | 1 | 2032.48 | 5120.83 | 945.80 | 79891.40 |  |  |  |
| 3390-72 | PIK3CA PIK3R1 | P42336 P27986 | 0 | 949.67 | 418.62 | 365.80 | 2228.30 | -0.081 | 3.07E-01 | 4.39E-01 |
|  |  |  | 1 | 978.61 | 1115.86 | 281.80 | 18111.70 |  |  |  |
| 5024-67 | RB1 | P06400 | 0 | 2341.66 | 2252.42 | 1443.60 | 23051.90 | -0.060 | 3.08E-01 | 4.40E-01 |
|  |  |  | 1 | 2163.64 | 1541.49 | 745.20 | 24021.70 |  |  |  |
| 4429-51 | CHST6 | Q9GZX3 | 0 | 350.26 | 108.40 | 222.40 | 1234.30 | 0.043 | 3.10E-01 | 4.41E-01 |
|  |  |  | 1 | 366.11 | 134.74 | 235.10 | 1340.50 |  |  |  |
| 4693-72 | HIBADH | P31937 | 0 | 901.67 | 1398.95 | 462.90 | 14442.30 | 0.082 | 3.12E-01 | 4.43E-01 |
|  |  |  | 1 | 998.77 | 1477.08 | 281.50 | 21491.60 |  |  |  |
| 3873-51 | TPO | P07202 | 0 | 2042.76 | 420.28 | 1638.30 | 5551.10 | 0.035 | 3.12E-01 | 4.43E-01 |
|  |  |  | 1 | 2132.43 | 685.42 | 1240.90 | 7962.00 |  |  |  |
| 3486-58 | FGF1 | P05230 | 0 | 216.53 | 49.39 | 153.40 | 555.20 | -0.048 | 3.12E-01 | 4.43E-01 |
|  |  |  | 1 | 228.52 | 300.21 | 100.90 | 5173.90 |  |  |  |
| 4551-72 | ADGRG5 | Q8IZF4 | 0 | 785.50 | 594.01 | 506.30 | 5723.20 | -0.057 | 3.14E-01 | 4.46E-01 |
|  |  |  | 1 | 957.19 | 4322.85 | 310.10 | 75349.30 |  |  |  |
| 3795-6 | ADAM9 | Q13443 | 0 | 3162.83 | 15317.83 | 819.80 | 154695.70 | 0.079 | 3.15E-01 | 4.47E-01 |
|  |  |  | 1 | 2125.50 | 4459.56 | 608.10 | 67628.80 |  |  |  |
| 5957-30 | SST | P61278 | 0 | 1751.06 | 259.55 | 1365.20 | 3170.60 | 0.040 | 3.16E-01 | 4.47E-01 |
|  |  |  | 1 | 1877.58 | 1055.11 | 495.80 | 15568.70 |  |  |  |
| 4959-2 | AGR2 | O95994 | 0 | 912.56 | 188.93 | 738.70 | 2419.20 | 0.041 | 3.19E-01 | 4.50E-01 |
|  |  |  | 1 | 994.75 | 926.76 | 631.20 | 15851.30 |  |  |  |
| 4914-10 | CGA CGB | P01215 P01233 | 0 | 3823.70 | 5207.66 | 396.10 | 26609.70 | -0.182 | 3.19E-01 | 4.50E-01 |
|  |  |  | 1 | 3336.21 | 5120.36 | 242.50 | 35548.40 |  |  |  |
| 10356-21 | JUN | P05412 | 0 | 6644.51 | 2072.96 | 4403.10 | 23295.00 | 0.039 | 3.20E-01 | 4.51E-01 |
|  |  |  | 1 | 6849.85 | 2058.79 | 2729.20 | 22964.50 |  |  |  |
| 5060-62 | CD274 | Q9NZQ7 | 0 | 1496.00 | 2136.82 | 419.00 | 20893.40 | 0.108 | 3.26E-01 | 4.58E-01 |
|  |  |  | 1 | 1905.60 | 3408.35 | 301.20 | 30586.00 |  |  |  |
| 4490-65 | IL3RA | P26951 | 0 | 1653.70 | 977.95 | 539.60 | 5393.90 | 0.100 | 3.26E-01 | 4.58E-01 |
|  |  |  | 1 | 1913.22 | 2013.56 | 476.80 | 30804.50 |  |  |  |
| 9170-24 | IL17A | Q16552 | 0 | 512.97 | 413.63 | 301.10 | 3773.50 | 0.055 | 3.26E-01 | 4.58E-01 |
|  |  |  | 1 | 532.49 | 756.97 | 282.80 | 13214.20 |  |  |  |
| 3623-84 | LY86 | O95711 | 0 | 4991.66 | 1013.10 | 3495.60 | 8148.50 | 0.038 | 3.26E-01 | 4.58E-01 |
|  |  |  | 1 | 5217.08 | 1681.47 | 3467.30 | 19623.40 |  |  |  |
| 4393-3 | FGF16 | O43320 | 0 | 3163.94 | 2076.21 | 690.10 | 8286.30 | 0.121 | 3.27E-01 | 4.59E-01 |
|  |  |  | 1 | 3918.55 | 5617.85 | 583.00 | 88377.20 |  |  |  |
| 3091-70 | AURKA | O14965 | 0 | 1583.64 | 1450.66 | 808.50 | 11713.70 | 0.088 | 3.28E-01 | 4.60E-01 |
|  |  |  | 1 | 2005.08 | 4489.05 | 757.50 | 67297.80 |  |  |  |
| 3897-61 | PDXP | Q96GD0 | 0 | 4872.08 | 3341.49 | 972.40 | 14092.60 | 0.123 | 3.29E-01 | 4.60E-01 |
|  |  |  | 1 | 5776.36 | 4939.55 | 834.60 | 43494.50 |  |  |  |
| 2906-55 | IL4 | P05112 | 0 | 592.93 | 124.77 | 414.80 | 911.40 | 0.042 | 3.32E-01 | 4.65E-01 |
|  |  |  | 1 | 629.49 | 328.32 | 377.50 | 5553.50 |  |  |  |
| 4292-5 | NAPA | P54920 | 0 | 10863.01 | 7573.75 | 2354.70 | 31827.30 | 0.128 | 3.33E-01 | 4.65E-01 |
|  |  |  | 1 | 13252.63 | 11523.74 | 1371.50 | 108830.20 |  |  |  |
| 3494-71 | FGF17 | O60258 | 0 | 245.45 | 122.37 | 182.10 | 1434.50 | -0.035 | 3.34E-01 | 4.66E-01 |
|  |  |  | 1 | 238.90 | 93.00 | 155.20 | 1325.60 |  |  |  |
| 4420-7 | ADAM12 | O43184 | 0 | 610.78 | 434.13 | 412.70 | 3956.70 | 0.061 | 3.35E-01 | 4.67E-01 |
|  |  |  | 1 | 816.87 | 3294.55 | 403.80 | 56188.60 |  |  |  |
| 3646-7 | TEC | P42680 | 0 | 5786.51 | 4341.33 | 857.70 | 19285.40 | -0.136 | 3.35E-01 | 4.67E-01 |
|  |  |  | 1 | 5703.44 | 4539.27 | 781.00 | 21685.90 |  |  |  |
| 8447-11 | GHRL | Q9UBU3 | 0 | 1538.45 | 4295.27 | 578.80 | 43869.10 | -0.070 | 3.36E-01 | 4.67E-01 |
|  |  |  | 1 | 1256.34 | 2613.98 | 390.10 | 44871.90 |  |  |  |
| 2823-7 | COMMD7 | Q86VX2 | 0 | 2386.37 | 1018.48 | 1030.50 | 6371.20 | 0.070 | 3.41E-01 | 4.74E-01 |
|  |  |  | 1 | 2603.74 | 1350.43 | 648.20 | 10192.90 |  |  |  |
| 5132-71 | IL27RA | Q6UWB1 | 0 | 1524.48 | 676.51 | 662.80 | 6912.80 | -0.044 | 3.42E-01 | 4.75E-01 |
|  |  |  | 1 | 1451.41 | 395.37 | 628.00 | 3121.50 |  |  |  |
| 3396-54 | REN | P00797 | 0 | 1740.52 | 1436.21 | 303.20 | 9042.30 | 0.119 | 3.44E-01 | 4.77E-01 |
|  |  |  | 1 | 2034.95 | 2340.20 | 200.30 | 18700.80 |  |  |  |
| 3061-61 | CTSB | P07858 | 0 | 2156.55 | 484.13 | 1180.10 | 3440.30 | -0.041 | 3.44E-01 | 4.77E-01 |
|  |  |  | 1 | 2133.58 | 749.34 | 1239.10 | 9745.70 |  |  |  |
| 3356-50 | CA7 | P43166 | 0 | 399.39 | 182.29 | 250.20 | 1538.30 | 0.058 | 3.45E-01 | 4.77E-01 |
|  |  |  | 1 | 492.85 | 1104.22 | 254.40 | 17340.50 |  |  |  |
| 4203-50 | CFL1 | P23528 | 0 | 10135.36 | 4153.29 | 3765.60 | 23097.00 | 0.094 | 3.48E-01 | 4.81E-01 |
|  |  |  | 1 | 12033.14 | 6945.08 | 2126.80 | 41269.20 |  |  |  |
| 3181-50 | CTSS | P25774 | 0 | 1038.92 | 236.68 | 600.50 | 1924.40 | 0.036 | 3.48E-01 | 4.81E-01 |
|  |  |  | 1 | 1068.39 | 251.25 | 554.60 | 1926.50 |  |  |  |
| 3034-1 | GFAP | P14136 | 0 | 1233.58 | 4621.37 | 418.70 | 45976.90 | -0.080 | 3.49E-01 | 4.81E-01 |
|  |  |  | 1 | 911.02 | 2586.66 | 396.00 | 32782.80 |  |  |  |
| 3800-71 | CKB | P12277 | 0 | 1019.76 | 1428.49 | 438.20 | 12287.60 | 0.093 | 3.50E-01 | 4.82E-01 |
|  |  |  | 1 | 1295.58 | 3349.82 | 339.60 | 43184.10 |  |  |  |
| 5487-7 | SLAMF7 | Q9NQ25 | 0 | 55593.87 | 25206.76 | 4781.70 | 167991.80 | -0.103 | 3.52E-01 | 4.84E-01 |
|  |  |  | 1 | 55076.64 | 33916.65 | 945.00 | 296831.30 |  |  |  |
| 3808-76 | FGFR2 | P21802 | 0 | 396.96 | 430.29 | 208.70 | 3039.20 | 0.086 | 3.55E-01 | 4.88E-01 |
|  |  |  | 1 | 526.36 | 1577.59 | 196.10 | 19128.60 |  |  |  |
| 3341-33 | ABL1 | P00519 | 0 | 179.24 | 108.01 | 113.40 | 1136.80 | 0.056 | 3.56E-01 | 4.90E-01 |
|  |  |  | 1 | 205.56 | 346.23 | 111.70 | 5828.20 |  |  |  |
| 5490-53 | SPOCK1 | Q08629 | 0 | 3396.61 | 1177.61 | 1875.30 | 6723.50 | 0.063 | 3.59E-01 | 4.93E-01 |
|  |  |  | 1 | 3780.43 | 2328.36 | 1532.70 | 26211.40 |  |  |  |
| 3309-2 | FCGR2A | P12318 | 0 | 2855.78 | 2107.30 | 45.60 | 7557.80 | 0.261 | 3.60E-01 | 4.93E-01 |
|  |  |  | 1 | 3303.86 | 2451.37 | 43.60 | 15347.80 |  |  |  |
| 3887-90 | KPNB1 | Q14974 | 0 | 8897.44 | 6606.92 | 654.60 | 32493.50 | -0.154 | 3.60E-01 | 4.94E-01 |
|  |  |  | 1 | 9623.20 | 8107.02 | 167.40 | 48595.20 |  |  |  |
| 12060-28 | GDF11 | O95390 | 0 | 1026.47 | 301.77 | 651.70 | 2509.10 | -0.041 | 3.61E-01 | 4.94E-01 |
|  |  |  | 1 | 1008.67 | 345.71 | 425.80 | 3550.50 |  |  |  |
| 4995-16 | HPGD | P15428 | 0 | 1484.60 | 339.60 | 989.90 | 2507.50 | 0.049 | 3.62E-01 | 4.95E-01 |
|  |  |  | 1 | 1616.93 | 823.25 | 849.30 | 7486.70 |  |  |  |
| 3078-1 | PGF | P49763 | 0 | 528.92 | 1228.16 | 272.80 | 12536.40 | -0.056 | 3.65E-01 | 4.98E-01 |
|  |  |  | 1 | 428.19 | 509.21 | 180.10 | 8674.90 |  |  |  |
| 4994-178 | HNRNPK | P61978 | 0 | 367.13 | 407.03 | 207.20 | 4132.90 | 0.068 | 3.65E-01 | 4.98E-01 |
|  |  |  | 1 | 469.28 | 1725.51 | 153.10 | 29822.60 |  |  |  |
| 8459-10 | BMP6 | P22004 | 0 | 21160.71 | 6027.05 | 8956.00 | 40212.90 | -0.057 | 3.66E-01 | 4.99E-01 |
|  |  |  | 1 | 20953.40 | 7585.22 | 1037.20 | 53722.40 |  |  |  |
| 2704-74 | TNFRSF13B | O14836 | 0 | 1283.18 | 1001.24 | 947.40 | 10927.40 | 0.045 | 3.68E-01 | 5.01E-01 |
|  |  |  | 1 | 1434.32 | 3146.18 | 841.50 | 55317.70 |  |  |  |
| 4815-25 | TXNDC12 | O95881 | 0 | 3367.49 | 1441.46 | 1903.30 | 15220.30 | 0.041 | 3.69E-01 | 5.01E-01 |
|  |  |  | 1 | 3435.91 | 1269.05 | 1353.70 | 19378.90 |  |  |  |
| 2993-1 | IL18RAP | O95256 | 0 | 367.52 | 193.77 | 248.80 | 1679.00 | -0.047 | 3.69E-01 | 5.01E-01 |
|  |  |  | 1 | 390.21 | 842.07 | 229.60 | 14754.80 |  |  |  |
| 3005-5 | PTPN1 | P18031 | 0 | 2861.78 | 1027.38 | 1645.70 | 6873.10 | 0.058 | 3.71E-01 | 5.03E-01 |
|  |  |  | 1 | 3144.52 | 2021.22 | 942.50 | 27597.60 |  |  |  |
| 5204-13 | PSME3 | P61289 | 0 | 473.52 | 1227.99 | 238.30 | 12446.60 | 0.058 | 3.72E-01 | 5.03E-01 |
|  |  |  | 1 | 385.41 | 237.83 | 221.80 | 2693.10 |  |  |  |
| 5460-60 | DDX19B | Q9UMR2 | 0 | 6377.84 | 4019.40 | 1452.00 | 18635.80 | -0.112 | 3.72E-01 | 5.03E-01 |
|  |  |  | 1 | 6499.19 | 4606.28 | 1195.60 | 28766.20 |  |  |  |
| 2998-53 | JAM3 | Q9BX67 | 0 | 6247.77 | 3166.43 | 2601.90 | 14245.70 | 0.080 | 3.72E-01 | 5.04E-01 |
|  |  |  | 1 | 6921.25 | 4270.96 | 1858.30 | 29535.40 |  |  |  |
| 5867-60 | ARG1 | P05089 | 0 | 1474.85 | 2734.45 | 465.50 | 17191.30 | -0.082 | 3.73E-01 | 5.04E-01 |
|  |  |  | 1 | 1172.37 | 1773.19 | 286.30 | 22876.10 |  |  |  |
| 5130-67 | SCARF2 | Q96GP6 | 0 | 529.96 | 1297.08 | 236.20 | 12738.70 | 0.081 | 3.75E-01 | 5.06E-01 |
|  |  |  | 1 | 876.07 | 7547.09 | 219.10 | 130645.10 |  |  |  |
| 3538-26 | DDC | P20711 | 0 | 279.30 | 51.03 | 198.30 | 453.20 | -0.044 | 3.75E-01 | 5.06E-01 |
|  |  |  | 1 | 292.57 | 250.59 | 172.50 | 3590.50 |  |  |  |
| 4964-67 | ERAP1 | Q9NZ08 | 0 | 81409.05 | 31946.71 | 17521.20 | 173891.80 | -0.077 | 3.79E-01 | 5.10E-01 |
|  |  |  | 1 | 78829.26 | 32633.58 | 3749.20 | 189006.30 |  |  |  |
| 3394-81 | PLK1 | P53350 | 0 | 216.15 | 66.75 | 157.60 | 740.00 | 0.037 | 3.80E-01 | 5.11E-01 |
|  |  |  | 1 | 225.20 | 86.67 | 138.80 | 945.70 |  |  |  |
| 3853-56 | MDH1 | P40925 | 0 | 30305.09 | 12410.09 | 13845.10 | 64485.30 | 0.067 | 3.83E-01 | 5.14E-01 |
|  |  |  | 1 | 33033.58 | 16960.17 | 9967.00 | 116672.40 |  |  |  |
| 8488-33 | ITGA2B ITGB3 | P08514 P05106 | 0 | 3137.04 | 2362.56 | 1210.40 | 16403.40 | -0.087 | 3.83E-01 | 5.14E-01 |
|  |  |  | 1 | 3305.65 | 4158.01 | 861.10 | 47871.20 |  |  |  |
| 2619-72 | TACSTD2 | P09758 | 0 | 670.41 | 1004.71 | 333.60 | 9684.70 | -0.062 | 3.84E-01 | 5.15E-01 |
|  |  |  | 1 | 630.26 | 1287.68 | 295.80 | 21254.20 |  |  |  |
| 2755-8 | C3 | P01024 | 0 | 185631.77 | 37122.31 | 107896.60 | 395737.90 | -0.041 | 3.84E-01 | 5.15E-01 |
|  |  |  | 1 | 184084.72 | 42341.27 | 21878.80 | 308596.00 |  |  |  |
| 13103-125 | CSH1 CSH2 | P0DML2 P0DML3 | 0 | 3124.37 | 1830.09 | 1254.30 | 9939.00 | 0.071 | 3.85E-01 | 5.15E-01 |
|  |  |  | 1 | 3421.57 | 4138.13 | 1218.60 | 68831.90 |  |  |  |
| 5091-28 | LILRB2 | Q8N423 | 0 | 11931.31 | 2707.90 | 4814.10 | 19957.70 | -0.041 | 3.87E-01 | 5.17E-01 |
|  |  |  | 1 | 11752.97 | 3292.08 | 1788.40 | 30573.20 |  |  |  |
| 10362-35 | MYC | P01106 | 0 | 628.08 | 536.84 | 370.90 | 4527.50 | 0.064 | 3.91E-01 | 5.22E-01 |
|  |  |  | 1 | 670.13 | 699.15 | 278.80 | 7295.80 |  |  |  |
| 3441-64 | GSK3A GSK3B | P49840 P49841 | 0 | 24563.04 | 16777.36 | 3709.20 | 62362.70 | -0.130 | 3.91E-01 | 5.22E-01 |
|  |  |  | 1 | 26184.14 | 19963.70 | 2635.40 | 82806.60 |  |  |  |
| 5117-14 | ROBO3 | Q96MS0 | 0 | 485.11 | 283.64 | 327.90 | 2374.90 | 0.047 | 3.93E-01 | 5.23E-01 |
|  |  |  | 1 | 518.53 | 510.69 | 313.50 | 7830.70 |  |  |  |
| 3316-58 | SERPIND1 | P05546 | 0 | 3677.13 | 736.24 | 1857.80 | 5829.50 | -0.028 | 3.93E-01 | 5.23E-01 |
|  |  |  | 1 | 3594.29 | 646.63 | 1475.00 | 5624.90 |  |  |  |
| 3799-11 | CA3 | P07451 | 0 | 3033.39 | 2021.45 | 912.20 | 14417.90 | -0.074 | 3.93E-01 | 5.23E-01 |
|  |  |  | 1 | 3049.38 | 3712.82 | 779.40 | 57913.00 |  |  |  |
| 2764-20 | FGF9 | P31371 | 0 | 590.36 | 124.99 | 411.30 | 1153.40 | -0.038 | 3.96E-01 | 5.26E-01 |
|  |  |  | 1 | 595.59 | 271.44 | 340.60 | 3008.20 |  |  |  |
| 3723-1 | NPPB | P16860 | 0 | 588.26 | 388.62 | 368.70 | 4004.50 | 0.052 | 3.97E-01 | 5.28E-01 |
|  |  |  | 1 | 644.91 | 826.94 | 341.90 | 13479.30 |  |  |  |
| 2859-69 | HDAC8 | Q9BY41 | 0 | 2706.96 | 834.36 | 1658.00 | 7344.90 | -0.043 | 4.01E-01 | 5.32E-01 |
|  |  |  | 1 | 2675.19 | 1040.57 | 1365.30 | 11845.00 |  |  |  |
| 3026-5 | CAST | P20810 | 0 | 8508.84 | 13024.24 | 3041.00 | 135640.30 | 0.056 | 4.02E-01 | 5.32E-01 |
|  |  |  | 1 | 8455.72 | 10826.69 | 2600.10 | 185723.70 |  |  |  |
| 5488-74 | SRC | P12931 | 0 | 77966.87 | 56512.05 | 2729.60 | 238109.70 | -0.160 | 4.02E-01 | 5.32E-01 |
|  |  |  | 1 | 85985.97 | 62956.24 | 1342.70 | 248414.10 |  |  |  |
| 4912-17 | GOT1 | P17174 | 0 | 3058.47 | 828.82 | 1423.20 | 5870.60 | 0.038 | 4.03E-01 | 5.32E-01 |
|  |  |  | 1 | 3148.93 | 905.24 | 767.70 | 8006.90 |  |  |  |
| 3317-33 | HTRA2 | O43464 | 0 | 5628.69 | 1835.18 | 1737.50 | 11752.10 | -0.060 | 4.03E-01 | 5.32E-01 |
|  |  |  | 1 | 5646.09 | 2535.33 | 549.50 | 28032.50 |  |  |  |
| 2970-60 | AREG | P15514 | 0 | 4255.04 | 2882.09 | 857.50 | 11441.20 | 0.107 | 4.05E-01 | 5.33E-01 |
|  |  |  | 1 | 5064.87 | 4609.01 | 720.60 | 42915.80 |  |  |  |
| 13113-7 | SPP1 | P10451 | 0 | 4660.05 | 2077.55 | 598.00 | 11368.90 | -0.100 | 4.05E-01 | 5.33E-01 |
|  |  |  | 1 | 4979.56 | 4153.86 | 206.90 | 38980.30 |  |  |  |
| 8450-36 | ADCYAP1 | P18509 | 0 | 276.32 | 125.37 | 172.10 | 1226.80 | 0.055 | 4.06E-01 | 5.35E-01 |
|  |  |  | 1 | 367.96 | 1297.20 | 145.80 | 22425.40 |  |  |  |
| 4294-16 | SPHK1 | Q9NYA1 | 0 | 16763.43 | 13553.78 | 911.60 | 49802.40 | -0.157 | 4.09E-01 | 5.38E-01 |
|  |  |  | 1 | 18493.81 | 15491.43 | 730.10 | 56691.40 |  |  |  |
| 3182-38 | ENTPD1 | P49961 | 0 | 1972.80 | 2946.77 | 1325.90 | 31083.90 | -0.042 | 4.10E-01 | 5.39E-01 |
|  |  |  | 1 | 1913.64 | 3742.03 | 735.60 | 65653.00 |  |  |  |
| 9180-6 | IFNGR2 | P38484 | 0 | 565.81 | 266.63 | 374.30 | 2911.90 | 0.048 | 4.11E-01 | 5.39E-01 |
|  |  |  | 1 | 643.80 | 887.93 | 369.50 | 13651.50 |  |  |  |
| 5927-4 | WISP3 | O95389 | 0 | 2009.96 | 1111.91 | 1222.20 | 10217.10 | -0.037 | 4.13E-01 | 5.41E-01 |
|  |  |  | 1 | 1920.68 | 822.25 | 802.90 | 11174.40 |  |  |  |
| 5356-2 | MIF | P14174 | 0 | 1462.35 | 526.64 | 807.20 | 3135.80 | 0.069 | 4.17E-01 | 5.46E-01 |
|  |  |  | 1 | 2065.71 | 7104.21 | 656.00 | 121846.20 |  |  |  |
| 5131-15 | TNFRSF19 | Q9NS68 | 0 | 1107.26 | 372.19 | 580.30 | 2855.90 | -0.042 | 4.18E-01 | 5.47E-01 |
|  |  |  | 1 | 1086.26 | 401.04 | 224.20 | 3799.10 |  |  |  |
| 4569-52 | SORCS2 | Q96PQ0 | 0 | 2573.48 | 13231.13 | 324.00 | 127734.90 | -0.121 | 4.18E-01 | 5.47E-01 |
|  |  |  | 1 | 1535.64 | 6517.02 | 258.20 | 98460.80 |  |  |  |
| 4314-12 | DCTPP1 | Q9H773 | 0 | 11663.34 | 3172.02 | 3372.00 | 24391.70 | -0.076 | 4.19E-01 | 5.47E-01 |
|  |  |  | 1 | 11955.15 | 4138.96 | 326.50 | 25848.90 |  |  |  |
| 13129-40 | LDLR | P01130 | 0 | 233.03 | 408.88 | 130.10 | 4248.80 | 0.061 | 4.24E-01 | 5.52E-01 |
|  |  |  | 1 | 301.28 | 1263.62 | 126.50 | 21480.60 |  |  |  |
| 4440-15 | FCRL3 | Q96P31 | 0 | 457.46 | 151.64 | 325.50 | 1622.60 | 0.047 | 4.23E-01 | 5.52E-01 |
|  |  |  | 1 | 571.21 | 1350.62 | 300.30 | 21921.40 |  |  |  |
| 3438-10 | FSTL3 | O95633 | 0 | 8782.08 | 2236.21 | 3774.10 | 15005.50 | -0.037 | 4.23E-01 | 5.52E-01 |
|  |  |  | 1 | 8629.66 | 2476.56 | 1524.40 | 20274.40 |  |  |  |
| 5359-65 | PIM1 | P11309 | 0 | 969.39 | 376.71 | 720.50 | 3488.30 | 0.037 | 4.24E-01 | 5.52E-01 |
|  |  |  | 1 | 1011.07 | 562.50 | 599.30 | 8134.50 |  |  |  |
| 4396-54 | IFNL1 | Q8IU54 | 0 | 413.38 | 176.63 | 209.00 | 1573.50 | 0.052 | 4.30E-01 | 5.58E-01 |
|  |  |  | 1 | 456.65 | 441.65 | 194.90 | 5878.30 |  |  |  |
| 3766-51 | STX1A | Q16623 | 0 | 382.48 | 43.99 | 293.70 | 581.70 | 0.028 | 4.30E-01 | 5.58E-01 |
|  |  |  | 1 | 401.68 | 149.78 | 265.40 | 1723.00 |  |  |  |
| 4135-84 | IGHE IGK@ IGL@ | P01854 | 0 | 4824.04 | 15911.67 | 140.30 | 133247.30 | 0.188 | 4.32E-01 | 5.60E-01 |
|  |  |  | 1 | 5481.49 | 13413.98 | 94.10 | 137885.90 |  |  |  |
| 11096-57 | N6AMT1 | Q9Y5N5 | 0 | 4195.62 | 2611.66 | 1342.40 | 12610.30 | 0.090 | 4.32E-01 | 5.60E-01 |
|  |  |  | 1 | 4947.36 | 3879.09 | 1137.10 | 23274.20 |  |  |  |
| 4122-12 | DDR1 | Q08345 | 0 | 349.73 | 74.33 | 243.60 | 740.50 | -0.047 | 4.32E-01 | 5.60E-01 |
|  |  |  | 1 | 534.75 | 3039.62 | 193.60 | 52179.80 |  |  |  |
| 4145-58 | NTF3 | P20783 | 0 | 278.59 | 144.64 | 108.10 | 1480.70 | 0.040 | 4.34E-01 | 5.62E-01 |
|  |  |  | 1 | 282.64 | 107.99 | 121.70 | 1100.80 |  |  |  |
| 5034-79 | PRSS2 | P07478 | 0 | 3918.62 | 2131.33 | 809.60 | 12282.20 | 0.070 | 4.39E-01 | 5.66E-01 |
|  |  |  | 1 | 4234.16 | 3326.54 | 566.80 | 36949.80 |  |  |  |
| 3292-75 | CD48 | P09326 | 0 | 719.61 | 129.23 | 430.70 | 1179.30 | -0.028 | 4.39E-01 | 5.66E-01 |
|  |  |  | 1 | 717.76 | 253.72 | 399.70 | 4198.30 |  |  |  |
| 2632-5 | IL12RB1 | P42701 | 0 | 642.70 | 190.45 | 450.60 | 1708.20 | -0.042 | 4.40E-01 | 5.67E-01 |
|  |  |  | 1 | 846.34 | 4011.41 | 392.00 | 69972.70 |  |  |  |
| 3291-30 | FCER2 | P06734 | 0 | 5428.26 | 1915.58 | 2409.60 | 10926.00 | -0.055 | 4.40E-01 | 5.67E-01 |
|  |  |  | 1 | 5445.73 | 2630.05 | 1280.90 | 18678.20 |  |  |  |
| 3866-7 | SBDS | Q9Y3A5 | 0 | 22217.77 | 18103.77 | 1747.30 | 71142.50 | 0.133 | 4.41E-01 | 5.68E-01 |
|  |  |  | 1 | 28691.48 | 23863.29 | 1821.80 | 111560.80 |  |  |  |
| 2778-10 | IL22 | Q9GZX6 | 0 | 1175.09 | 772.79 | 527.90 | 6696.30 | 0.067 | 4.43E-01 | 5.70E-01 |
|  |  |  | 1 | 1721.22 | 6619.61 | 479.60 | 104602.50 |  |  |  |
| 3070-1 | IL2 | P60568 | 0 | 1785.51 | 790.90 | 541.20 | 8077.80 | 0.050 | 4.45E-01 | 5.70E-01 |
|  |  |  | 1 | 1898.19 | 1071.71 | 346.00 | 14946.90 |  |  |  |
| 2715-25 | EPOR | P19235 | 0 | 447.15 | 179.72 | 293.70 | 1479.00 | 0.045 | 4.45E-01 | 5.70E-01 |
|  |  |  | 1 | 491.77 | 471.77 | 258.80 | 6124.90 |  |  |  |
| 3187-52 | CRISP3 | P54108 | 0 | 355.58 | 353.38 | 230.10 | 3041.30 | 0.043 | 4.45E-01 | 5.70E-01 |
|  |  |  | 1 | 343.86 | 170.46 | 222.50 | 1819.80 |  |  |  |
| 4148-49 | PAPPA | Q13219 | 0 | 20176.01 | 8373.73 | 8014.60 | 54619.60 | 0.056 | 4.48E-01 | 5.73E-01 |
|  |  |  | 1 | 21588.10 | 10395.93 | 2922.10 | 71570.60 |  |  |  |
| 4128-27 | CCL24 | O00175 | 0 | 409.62 | 505.47 | 289.70 | 5326.90 | 0.037 | 4.54E-01 | 5.81E-01 |
|  |  |  | 1 | 393.93 | 197.27 | 242.90 | 1872.30 |  |  |  |
| 2843-13 | SPINT2 | O43291 | 0 | 1389.27 | 433.92 | 726.20 | 2781.70 | 0.045 | 4.55E-01 | 5.82E-01 |
|  |  |  | 1 | 1483.42 | 818.81 | 405.70 | 12502.20 |  |  |  |
| 13089-6 | HIF1A | Q16665 | 0 | 607.25 | 361.62 | 339.20 | 3483.40 | 0.037 | 4.56E-01 | 5.82E-01 |
|  |  |  | 1 | 611.90 | 252.62 | 228.10 | 2588.80 |  |  |  |
| 5114-65 | PRLR | P16471 | 0 | 1670.29 | 2855.36 | 928.00 | 29107.60 | -0.050 | 4.60E-01 | 5.86E-01 |
|  |  |  | 1 | 1465.77 | 1148.20 | 835.30 | 9575.30 |  |  |  |
| 3363-31 | CSK | P41240 | 0 | 17039.90 | 14715.72 | 618.10 | 60402.00 | -0.156 | 4.60E-01 | 5.86E-01 |
|  |  |  | 1 | 20594.60 | 20321.57 | 403.30 | 102248.10 |  |  |  |
| 5879-51 | DCTN2 | Q13561 | 0 | 1288.16 | 227.74 | 882.60 | 1955.20 | -0.020 | 4.62E-01 | 5.88E-01 |
|  |  |  | 1 | 1267.99 | 209.85 | 868.60 | 2396.10 |  |  |  |
| 4491-4 | IL5RA | Q01344 | 0 | 2086.10 | 837.42 | 690.90 | 4670.70 | -0.057 | 4.65E-01 | 5.91E-01 |
|  |  |  | 1 | 2103.70 | 1211.06 | 493.20 | 12618.80 |  |  |  |
| 3404-51 | TPSG1 | Q9NRR2 | 0 | 204.62 | 56.86 | 140.30 | 556.50 | -0.037 | 4.67E-01 | 5.94E-01 |
|  |  |  | 1 | 207.39 | 101.95 | 113.60 | 878.40 |  |  |  |
| 5178-5 | PDE7A | Q13946 | 0 | 498.10 | 246.78 | 312.30 | 2599.00 | 0.035 | 4.69E-01 | 5.94E-01 |
|  |  |  | 1 | 522.28 | 406.57 | 219.40 | 6610.60 |  |  |  |
| 4876-32 | F9 | P00740 | 0 | 12672.46 | 1957.21 | 6479.30 | 16151.30 | 0.021 | 4.68E-01 | 5.94E-01 |
|  |  |  | 1 | 12832.41 | 1925.28 | 4347.70 | 20614.00 |  |  |  |
| 3427-63 | CSNK2A1 | P68400 | 0 | 671.10 | 543.02 | 378.70 | 5708.30 | -0.046 | 4.69E-01 | 5.94E-01 |
|  |  |  | 1 | 650.90 | 494.53 | 324.00 | 7390.60 |  |  |  |
| 4916-2 | IGHD IGK@ IGL@ | P01880 | 0 | 6841.00 | 10226.17 | 156.10 | 76910.80 | -0.169 | 4.69E-01 | 5.94E-01 |
|  |  |  | 1 | 8156.56 | 16063.46 | 99.80 | 105800.70 |  |  |  |
| 3115-64 | MAPK1 | P28482 | 0 | 10619.27 | 7545.38 | 2744.10 | 30541.90 | 0.103 | 4.72E-01 | 5.97E-01 |
|  |  |  | 1 | 12851.77 | 10374.54 | 926.40 | 57491.80 |  |  |  |
| 11513-92 | CD38 | P28907 | 0 | 788.51 | 251.08 | 505.10 | 2414.10 | -0.039 | 4.74E-01 | 5.98E-01 |
|  |  |  | 1 | 844.11 | 1209.46 | 342.40 | 20511.80 |  |  |  |
| 4957-1 | RPSA | P08865 | 0 | 1884.95 | 1566.43 | 1107.90 | 16629.10 | 0.047 | 4.75E-01 | 5.99E-01 |
|  |  |  | 1 | 2303.43 | 7156.43 | 834.60 | 124547.20 |  |  |  |
| 4976-57 | CRK | P46108 | 0 | 8808.12 | 4621.44 | 1223.90 | 24369.10 | -0.082 | 4.77E-01 | 6.02E-01 |
|  |  |  | 1 | 9053.21 | 5299.89 | 329.80 | 33027.80 |  |  |  |
| 10370-21 | STAT1 | P42224 | 0 | 17683.80 | 13156.40 | 4410.90 | 80420.60 | 0.102 | 4.78E-01 | 6.02E-01 |
|  |  |  | 1 | 21182.89 | 16238.04 | 1563.30 | 82058.20 |  |  |  |
| 3634-5 | OPCML | Q14982 | 0 | 3521.01 | 486.35 | 2702.90 | 5294.60 | -0.022 | 4.79E-01 | 6.02E-01 |
|  |  |  | 1 | 3520.22 | 950.97 | 2232.80 | 12922.00 |  |  |  |
| 2962-50 | PTHLH | P12272 | 0 | 1089.60 | 364.70 | 515.70 | 3462.20 | -0.034 | 4.79E-01 | 6.02E-01 |
|  |  |  | 1 | 1094.12 | 682.01 | 477.50 | 10450.80 |  |  |  |
| 14147-50 | IFNG | P01579 | 0 | 842.16 | 196.79 | 457.00 | 1685.10 | -0.056 | 4.79E-01 | 6.02E-01 |
|  |  |  | 1 | 1216.55 | 3996.50 | 243.20 | 52208.60 |  |  |  |
| 4359-87 | DYRK3 | O43781 | 0 | 2512.40 | 391.20 | 1883.00 | 4262.00 | 0.034 | 4.80E-01 | 6.03E-01 |
|  |  |  | 1 | 2958.43 | 6088.53 | 1526.60 | 106489.00 |  |  |  |
| 3421-54 | TNFSF8 | P32971 | 0 | 2774.40 | 968.88 | 1623.00 | 9943.50 | -0.037 | 4.85E-01 | 6.08E-01 |
|  |  |  | 1 | 2747.97 | 940.25 | 723.60 | 9626.70 |  |  |  |
| 11098-1 | PDXK | O00764 | 0 | 463.18 | 384.02 | 99.20 | 3949.80 | 0.052 | 4.90E-01 | 6.13E-01 |
|  |  |  | 1 | 473.10 | 218.65 | 84.50 | 1895.30 |  |  |  |
| 3074-6 | LBP | P18428 | 0 | 100676.63 | 18303.23 | 54470.10 | 171422.80 | -0.024 | 4.90E-01 | 6.13E-01 |
|  |  |  | 1 | 99630.14 | 20827.21 | 32039.40 | 192619.30 |  |  |  |
| 3831-21 | PTEN | P60484 | 0 | 958.74 | 1274.17 | 311.20 | 13062.70 | -0.057 | 4.90E-01 | 6.13E-01 |
|  |  |  | 1 | 907.16 | 854.56 | 345.60 | 9392.10 |  |  |  |
| 3469-74 | RPS6KA3 | P51812 | 0 | 982.05 | 789.42 | 231.60 | 5531.10 | 0.085 | 4.92E-01 | 6.14E-01 |
|  |  |  | 1 | 1148.61 | 1088.05 | 221.70 | 9365.90 |  |  |  |
| 9188-119 | CXCL9 | Q07325 | 0 | 923.49 | 1365.23 | 290.00 | 10754.80 | 0.067 | 4.93E-01 | 6.14E-01 |
|  |  |  | 1 | 893.39 | 916.53 | 167.20 | 10262.50 |  |  |  |
| 4961-17 | ANXA2 | P07355 | 0 | 8396.91 | 3095.44 | 3514.10 | 21956.10 | 0.057 | 4.94E-01 | 6.14E-01 |
|  |  |  | 1 | 9517.94 | 5656.38 | 1023.80 | 39573.00 |  |  |  |
| 3082-9 | ULBP2 | Q9BZM5 | 0 | 501.70 | 931.74 | 271.70 | 9289.80 | 0.056 | 4.94E-01 | 6.14E-01 |
|  |  |  | 1 | 529.86 | 924.39 | 234.60 | 11181.50 |  |  |  |
| 9178-30 | NRG1 | Q02297 | 0 | 223.26 | 95.72 | 145.90 | 884.60 | 0.041 | 4.93E-01 | 6.14E-01 |
|  |  |  | 1 | 246.84 | 289.67 | 93.80 | 4839.10 |  |  |  |
| 9171-11 | CSRP3 | P50461 | 0 | 3957.24 | 3957.45 | 1543.40 | 39907.30 | 0.048 | 4.96E-01 | 6.16E-01 |
|  |  |  | 1 | 3826.47 | 1643.99 | 974.50 | 11661.90 |  |  |  |
| 3195-50 | GNLY | P22749 | 0 | 2692.45 | 1092.77 | 989.20 | 6251.90 | -0.050 | 5.00E-01 | 6.21E-01 |
|  |  |  | 1 | 2707.08 | 1421.71 | 786.10 | 10773.80 |  |  |  |
| 2836-68 | LCN2 | P80188 | 0 | 21991.42 | 9363.36 | 8335.80 | 56820.40 | 0.046 | 5.01E-01 | 6.21E-01 |
|  |  |  | 1 | 23015.56 | 10836.76 | 5254.30 | 105826.70 |  |  |  |
| 2888-49 | C7 | P10643 | 0 | 1578.49 | 336.23 | 814.20 | 2586.60 | 0.027 | 5.01E-01 | 6.21E-01 |
|  |  |  | 1 | 1628.04 | 546.86 | 728.90 | 8490.80 |  |  |  |
| 3613-62 | FCN1 | O00602 | 0 | 1751.19 | 648.69 | 728.70 | 5036.30 | 0.043 | 5.04E-01 | 6.24E-01 |
|  |  |  | 1 | 1859.38 | 904.92 | 666.70 | 9724.50 |  |  |  |
| 10364-6 | SMAD2 | Q15796 | 0 | 21576.02 | 18360.68 | 3458.70 | 71278.90 | -0.113 | 5.06E-01 | 6.26E-01 |
|  |  |  | 1 | 22988.54 | 19727.67 | 1754.60 | 84765.70 |  |  |  |
| 4834-61 | EPHA2 | P29317 | 0 | 3249.20 | 915.54 | 1630.40 | 6516.50 | -0.042 | 5.08E-01 | 6.28E-01 |
|  |  |  | 1 | 3280.13 | 1278.28 | 277.20 | 10771.00 |  |  |  |
| 3522-57 | VIP | P01282 | 0 | 454.34 | 255.69 | 213.20 | 2456.00 | 0.045 | 5.10E-01 | 6.30E-01 |
|  |  |  | 1 | 483.17 | 415.73 | 207.40 | 6816.00 |  |  |  |
| 3872-2 | TPT1 | P13693 | 0 | 19748.29 | 13756.32 | 2793.50 | 53864.20 | 0.096 | 5.11E-01 | 6.31E-01 |
|  |  |  | 1 | 24423.91 | 19898.56 | 2765.40 | 92584.30 |  |  |  |
| 3414-40 | BMX | P51813 | 0 | 437.67 | 199.75 | 285.70 | 1533.30 | 0.043 | 5.14E-01 | 6.33E-01 |
|  |  |  | 1 | 521.19 | 927.10 | 258.70 | 13310.50 |  |  |  |
| 4992-49 | GRN | P28799 | 0 | 28318.95 | 4968.66 | 15852.50 | 40720.70 | -0.022 | 5.20E-01 | 6.40E-01 |
|  |  |  | 1 | 28068.73 | 5807.63 | 11091.70 | 45971.80 |  |  |  |
| 5225-50 | CSNK2A1 CSNK2B | P68400 P67870 | 0 | 12179.17 | 8698.16 | 1562.90 | 41326.90 | 0.087 | 5.26E-01 | 6.47E-01 |
|  |  |  | 1 | 13888.12 | 9774.60 | 1184.60 | 42653.10 |  |  |  |
| 2774-10 | IL16 | Q14005 | 0 | 351.97 | 51.59 | 263.70 | 664.60 | 0.020 | 5.27E-01 | 6.48E-01 |
|  |  |  | 1 | 364.01 | 125.48 | 219.40 | 1955.30 |  |  |  |
| 5834-18 | IL9 | P15248 | 0 | 319.09 | 172.36 | 171.60 | 1582.90 | 0.044 | 5.29E-01 | 6.49E-01 |
|  |  |  | 1 | 359.66 | 429.75 | 173.80 | 5748.10 |  |  |  |
| 4561-65 | BIRC7 | Q96CA5 | 0 | 135.75 | 29.64 | 102.40 | 294.40 | 0.033 | 5.29E-01 | 6.49E-01 |
|  |  |  | 1 | 164.00 | 380.53 | 94.10 | 6542.60 |  |  |  |
| 5265-12 | GRAP2 | O75791 | 0 | 643.54 | 480.32 | 420.90 | 4690.60 | 0.037 | 5.30E-01 | 6.49E-01 |
|  |  |  | 1 | 657.37 | 484.46 | 385.30 | 5865.40 |  |  |  |
| 5094-62 | AMICA1 | Q86YT9 | 0 | 702.06 | 1218.66 | 369.00 | 12337.90 | -0.041 | 5.32E-01 | 6.51E-01 |
|  |  |  | 1 | 606.15 | 483.60 | 363.70 | 4815.10 |  |  |  |
| 3554-24 | ADIPOQ | Q15848 | 0 | 3541.48 | 1653.18 | 348.00 | 10313.20 | -0.046 | 5.33E-01 | 6.52E-01 |
|  |  |  | 1 | 3360.80 | 1522.07 | 1165.60 | 11153.30 |  |  |  |
| 3172-28 | ARSB | P15848 | 0 | 1507.70 | 3628.08 | 831.40 | 37309.20 | -0.047 | 5.34E-01 | 6.52E-01 |
|  |  |  | 1 | 1658.96 | 6437.63 | 559.30 | 104010.50 |  |  |  |
| 8446-4 | ADCYAP1 | P18509 | 0 | 894.62 | 2564.57 | 207.40 | 20852.60 | -0.079 | 5.40E-01 | 6.59E-01 |
|  |  |  | 1 | 947.76 | 4512.28 | 111.00 | 74454.40 |  |  |  |
| 9176-3 | MUC1 | P15941 | 0 | 1500.45 | 1894.23 | 755.60 | 19812.70 | -0.045 | 5.40E-01 | 6.59E-01 |
|  |  |  | 1 | 1492.26 | 1582.42 | 455.70 | 16434.20 |  |  |  |
| 5066-134 | CD300C | Q08708 | 0 | 4730.06 | 1286.42 | 2681.10 | 9729.50 | -0.028 | 5.41E-01 | 6.59E-01 |
|  |  |  | 1 | 4664.82 | 1346.88 | 2196.90 | 12007.60 |  |  |  |
| 3836-51 | UFM1 | P61960 | 0 | 5418.80 | 3598.58 | 980.20 | 16989.70 | -0.082 | 5.44E-01 | 6.62E-01 |
|  |  |  | 1 | 5883.93 | 4614.89 | 342.40 | 28259.50 |  |  |  |
| 3797-1 | CDH2 | P19022 | 0 | 2340.98 | 2517.36 | 1147.20 | 18346.20 | -0.042 | 5.45E-01 | 6.62E-01 |
|  |  |  | 1 | 2064.37 | 1523.96 | 1110.10 | 16913.50 |  |  |  |
| 4982-54 | PI3 | P19957 | 0 | 13231.16 | 5592.18 | 3991.30 | 32645.90 | -0.044 | 5.45E-01 | 6.62E-01 |
|  |  |  | 1 | 13025.52 | 6099.97 | 3393.00 | 38231.90 |  |  |  |
| 5264-65 | CALR | P27797 | 0 | 2746.30 | 1408.81 | 1794.40 | 11907.90 | -0.037 | 5.47E-01 | 6.64E-01 |
|  |  |  | 1 | 2968.31 | 5183.41 | 1666.60 | 88055.20 |  |  |  |
| 3825-18 | MAPK8 | P45983 | 0 | 2164.94 | 1983.82 | 536.40 | 16815.00 | -0.075 | 5.48E-01 | 6.64E-01 |
|  |  |  | 1 | 2226.70 | 2479.88 | 386.80 | 29182.60 |  |  |  |
| 13097-11 | BCL2L2 | Q92843 | 0 | 650.69 | 121.10 | 429.80 | 1243.30 | 0.026 | 5.49E-01 | 6.65E-01 |
|  |  |  | 1 | 684.59 | 281.18 | 201.20 | 3589.20 |  |  |  |
| 2831-29 | KLK11 | Q9UBX7 | 0 | 1269.86 | 448.89 | 405.70 | 3434.30 | 0.035 | 5.50E-01 | 6.65E-01 |
|  |  |  | 1 | 1302.41 | 476.04 | 346.30 | 4272.10 |  |  |  |
| 2878-66 | YES1 | P07947 | 0 | 1439.24 | 543.06 | 745.90 | 3308.40 | 0.039 | 5.50E-01 | 6.66E-01 |
|  |  |  | 1 | 1512.77 | 646.34 | 551.80 | 3799.00 |  |  |  |
| 4162-54 | TF | P02787 | 0 | 228879.25 | 30518.24 | 160885.00 | 325160.50 | 0.013 | 5.55E-01 | 6.70E-01 |
|  |  |  | 1 | 230948.37 | 30353.79 | 147061.30 | 345926.70 |  |  |  |
| 4309-59 | TPI1 | P60174 | 0 | 12219.45 | 8061.17 | 3262.80 | 38830.90 | 0.077 | 5.55E-01 | 6.70E-01 |
|  |  |  | 1 | 14720.35 | 12242.79 | 2057.50 | 95746.60 |  |  |  |
| 4137-57 | IL25 | Q9H293 | 0 | 337.80 | 139.48 | 228.60 | 1371.10 | 0.038 | 5.58E-01 | 6.73E-01 |
|  |  |  | 1 | 410.78 | 758.25 | 185.30 | 11476.70 |  |  |  |
| 4271-75 | PFDN5 | Q99471 | 0 | 403.96 | 96.35 | 250.90 | 938.40 | -0.027 | 5.62E-01 | 6.77E-01 |
|  |  |  | 1 | 421.52 | 406.03 | 170.50 | 6898.30 |  |  |  |
| 5017-19 | PRDX5 | P30044 | 0 | 582.41 | 103.35 | 433.30 | 1083.60 | -0.032 | 5.63E-01 | 6.78E-01 |
|  |  |  | 1 | 681.29 | 1199.91 | 377.30 | 13165.80 |  |  |  |
| 5801-72 | NGF | P01138 | 0 | 1163.46 | 1645.25 | 409.30 | 11707.90 | -0.061 | 5.65E-01 | 6.80E-01 |
|  |  |  | 1 | 1149.28 | 2821.29 | 249.50 | 41815.30 |  |  |  |
| 3803-10 | CST5 | P28325 | 0 | 11449.26 | 5952.50 | 2457.80 | 31362.60 | 0.053 | 5.71E-01 | 6.86E-01 |
|  |  |  | 1 | 12402.38 | 8085.07 | 560.60 | 67475.60 |  |  |  |
| 3813-3 | FYN | P06241 | 0 | 6798.67 | 8369.00 | 1002.20 | 44537.70 | 0.091 | 5.77E-01 | 6.92E-01 |
|  |  |  | 1 | 7377.30 | 7740.72 | 846.20 | 40133.00 |  |  |  |
| 4460-8 | PDPK1 | O15530 | 0 | 1666.75 | 1771.51 | 208.40 | 7433.90 | -0.098 | 5.77E-01 | 6.92E-01 |
|  |  |  | 1 | 1760.50 | 2288.28 | 108.30 | 29071.10 |  |  |  |
| 3057-55 | WISP1 | O95388 | 0 | 5389.23 | 1227.09 | 3325.00 | 9755.40 | 0.028 | 5.78E-01 | 6.93E-01 |
|  |  |  | 1 | 5827.30 | 4070.81 | 2846.50 | 48735.40 |  |  |  |
| 5245-40 | PRKAA2 PRKAB2 PRKAG1 | P54646 O43741 P54619 | 0 | 5950.57 | 5171.95 | 1154.00 | 28567.70 | 0.072 | 5.80E-01 | 6.94E-01 |
|  |  |  | 1 | 6423.86 | 5140.24 | 1178.70 | 28055.60 |  |  |  |
| 5084-154 | IL17RB | Q9NRM6 | 0 | 1532.54 | 1173.74 | 752.80 | 8603.70 | -0.038 | 5.80E-01 | 6.94E-01 |
|  |  |  | 1 | 1471.99 | 1393.61 | 588.10 | 22535.10 |  |  |  |
| 5065-8 | CD83 | Q01151 | 0 | 385.54 | 303.64 | 200.30 | 3090.60 | 0.035 | 5.81E-01 | 6.94E-01 |
|  |  |  | 1 | 401.23 | 342.41 | 171.70 | 4251.10 |  |  |  |
| 3822-54 | MAPKAPK3 | Q16644 | 0 | 2042.50 | 1381.14 | 417.70 | 6860.50 | 0.073 | 5.82E-01 | 6.95E-01 |
|  |  |  | 1 | 2492.23 | 2176.35 | 316.70 | 11875.60 |  |  |  |
| 5316-54 | F2 | P00734 | 0 | 162767.42 | 25487.43 | 102404.40 | 222951.30 | -0.015 | 5.83E-01 | 6.95E-01 |
|  |  |  | 1 | 161070.31 | 24615.99 | 76775.00 | 241468.80 |  |  |  |
| 4322-28 | AMN | Q9BXJ7 | 0 | 165.40 | 40.55 | 115.40 | 420.10 | 0.035 | 5.84E-01 | 6.96E-01 |
|  |  |  | 1 | 199.33 | 283.01 | 102.70 | 3444.10 |  |  |  |
| 5437-63 | FABP3 | P05413 | 0 | 26961.05 | 12706.61 | 11147.10 | 99177.90 | 0.041 | 5.91E-01 | 7.03E-01 |
|  |  |  | 1 | 28333.49 | 14045.61 | 4534.00 | 106888.00 |  |  |  |
| 5328-33 | EGFR | P00533 | 0 | 328.44 | 401.77 | 164.50 | 4044.70 | 0.047 | 5.92E-01 | 7.04E-01 |
|  |  |  | 1 | 422.78 | 1280.66 | 168.30 | 20480.40 |  |  |  |
| 3629-60 | CDC42BPB | Q9Y5S2 | 0 | 1788.89 | 2596.60 | 1030.60 | 22034.70 | 0.029 | 5.95E-01 | 7.07E-01 |
|  |  |  | 1 | 1655.50 | 1973.53 | 885.30 | 34852.70 |  |  |  |
| 3657-74 | PPP3R1 | P63098 | 0 | 8707.65 | 1295.07 | 6353.60 | 12542.60 | 0.019 | 5.97E-01 | 7.08E-01 |
|  |  |  | 1 | 9005.85 | 2674.70 | 4193.50 | 29626.00 |  |  |  |
| 4468-21 | SPHK2 | Q9NRA0 | 0 | 713.18 | 497.38 | 483.20 | 4584.30 | -0.031 | 5.96E-01 | 7.08E-01 |
|  |  |  | 1 | 930.79 | 4777.21 | 423.20 | 83297.50 |  |  |  |
| 14128-121 | IFNA10 | P01566 | 0 | 400.99 | 177.51 | 229.90 | 1405.80 | -0.042 | 5.99E-01 | 7.10E-01 |
|  |  |  | 1 | 724.50 | 4526.21 | 148.80 | 72968.00 |  |  |  |
| 5280-68 | SLC25A18 | Q9H1K4 | 0 | 987.33 | 248.53 | 694.00 | 3053.30 | 0.021 | 6.02E-01 | 7.12E-01 |
|  |  |  | 1 | 1033.71 | 546.49 | 438.70 | 8320.20 |  |  |  |
| 5451-1 | ALCAM | Q13740 | 0 | 19704.78 | 2949.83 | 10677.20 | 25219.00 | -0.018 | 6.02E-01 | 7.12E-01 |
|  |  |  | 1 | 19690.70 | 4115.77 | 4402.80 | 33135.80 |  |  |  |
| 5888-29 | EIF5A | P63241 | 0 | 42783.49 | 18444.35 | 10349.50 | 79585.10 | -0.051 | 6.02E-01 | 7.12E-01 |
|  |  |  | 1 | 44275.34 | 22485.11 | 5080.30 | 101939.90 |  |  |  |
| 5261-13 | ABL2 | P42684 | 0 | 2089.55 | 737.74 | 1509.90 | 8010.30 | 0.021 | 6.03E-01 | 7.12E-01 |
|  |  |  | 1 | 2144.51 | 965.39 | 1372.60 | 14744.80 |  |  |  |
| 2944-66 | NBL1 | P41271 | 0 | 1331.46 | 534.38 | 410.70 | 3672.60 | 0.043 | 6.04E-01 | 7.13E-01 |
|  |  |  | 1 | 1540.41 | 1886.32 | 406.90 | 30088.70 |  |  |  |
| 2769-3 | Human-virus | P18093 | 0 | 204.67 | 265.91 | 134.60 | 2784.00 | -0.027 | 6.05E-01 | 7.13E-01 |
|  |  |  | 1 | 194.14 | 208.71 | 122.10 | 3424.20 |  |  |  |
| 5256-86 | PDE5A | O76074 | 0 | 27357.68 | 21012.66 | 1117.40 | 94425.90 | -0.091 | 6.09E-01 | 7.17E-01 |
|  |  |  | 1 | 30599.79 | 24673.86 | 1452.10 | 100891.50 |  |  |  |
| 4304-18 | LCORL | Q8N3X6 | 0 | 261.09 | 89.90 | 198.00 | 1105.20 | -0.027 | 6.12E-01 | 7.20E-01 |
|  |  |  | 1 | 303.69 | 659.49 | 157.90 | 8578.90 |  |  |  |
| 14115-34 | ADM | P35318 | 0 | 2103.60 | 392.27 | 1405.50 | 4241.80 | 0.017 | 6.16E-01 | 7.25E-01 |
|  |  |  | 1 | 2148.73 | 545.41 | 1332.40 | 7243.70 |  |  |  |
| 4209-60 | VTA1 | Q9NP79 | 0 | 42021.82 | 31794.98 | 5708.10 | 115389.30 | -0.078 | 6.18E-01 | 7.25E-01 |
|  |  |  | 1 | 44309.35 | 33842.50 | 3345.40 | 128000.60 |  |  |  |
| 3007-7 | SIGLEC9 | Q9Y336 | 0 | 16797.44 | 14914.07 | 178.20 | 56276.50 | -0.182 | 6.18E-01 | 7.25E-01 |
|  |  |  | 1 | 14617.96 | 13812.94 | 161.80 | 55063.10 |  |  |  |
| 2682-68 | HSPD1 | P10809 | 0 | 20930.18 | 18363.97 | 1689.00 | 118772.10 | 0.087 | 6.20E-01 | 7.26E-01 |
|  |  |  | 1 | 27118.97 | 24969.36 | 996.20 | 159711.20 |  |  |  |
| 4452-9 | LRRTM1 | Q86UE6 | 0 | 155.00 | 192.35 | 97.20 | 1917.20 | 0.042 | 6.19E-01 | 7.26E-01 |
|  |  |  | 1 | 422.19 | 3611.40 | 87.70 | 55454.60 |  |  |  |
| 4721-54 | TFF3 | Q07654 | 0 | 15095.43 | 10591.45 | 5047.50 | 86953.60 | -0.029 | 6.22E-01 | 7.28E-01 |
|  |  |  | 1 | 14220.18 | 5836.44 | 2213.00 | 66085.00 |  |  |  |
| 4807-13 | COL8A1 | P27658 | 0 | 1579.55 | 2234.55 | 549.60 | 13846.90 | -0.057 | 6.23E-01 | 7.28E-01 |
|  |  |  | 1 | 1809.96 | 6588.72 | 351.80 | 100941.90 |  |  |  |
| 4165-2 | TG | P01266 | 0 | 426.71 | 241.58 | 322.10 | 2484.10 | 0.022 | 6.24E-01 | 7.29E-01 |
|  |  |  | 1 | 439.04 | 280.07 | 289.30 | 3169.20 |  |  |  |
| 2946-52 | CFD | P00746 | 0 | 1139.25 | 370.23 | 733.50 | 4241.00 | -0.020 | 6.25E-01 | 7.30E-01 |
|  |  |  | 1 | 1127.13 | 326.79 | 355.50 | 4418.90 |  |  |  |
| 2781-63 | CCL4L1 | Q8NHW4 | 0 | 633.16 | 371.58 | 248.90 | 3380.60 | 0.047 | 6.28E-01 | 7.31E-01 |
|  |  |  | 1 | 1254.94 | 7624.19 | 132.60 | 122599.60 |  |  |  |
| 4909-68 | LGALS8 | O00214 | 0 | 530.13 | 155.26 | 339.60 | 1595.80 | 0.023 | 6.28E-01 | 7.31E-01 |
|  |  |  | 1 | 579.85 | 729.95 | 233.60 | 12626.60 |  |  |  |
| 4792-51 | Human-virus | Q70626 | 0 | 999.98 | 307.56 | 566.20 | 1992.30 | -0.024 | 6.29E-01 | 7.31E-01 |
|  |  |  | 1 | 995.99 | 437.58 | 460.70 | 6172.70 |  |  |  |
| 3583-54 | ARSA | P15289 | 0 | 1862.16 | 4230.78 | 795.70 | 42714.60 | -0.025 | 6.28E-01 | 7.31E-01 |
|  |  |  | 1 | 1433.51 | 457.05 | 628.20 | 5248.00 |  |  |  |
| 3479-71 | PRSS3 | P35030 | 0 | 495.79 | 160.23 | 355.60 | 1740.70 | -0.026 | 6.29E-01 | 7.31E-01 |
|  |  |  | 1 | 546.16 | 1010.10 | 240.80 | 17584.30 |  |  |  |
| 5020-50 | PGK1 | P00558 | 0 | 3368.95 | 1769.20 | 1127.20 | 9853.20 | 0.045 | 6.30E-01 | 7.32E-01 |
|  |  |  | 1 | 3843.43 | 4359.09 | 567.20 | 68567.80 |  |  |  |
| 5108-72 | NOTCH3 | Q9UM47 | 0 | 738.30 | 226.68 | 416.20 | 2033.60 | -0.021 | 6.35E-01 | 7.36E-01 |
|  |  |  | 1 | 735.67 | 292.01 | 379.90 | 3435.00 |  |  |  |
| 3401-8 | PTPN2 | P17706 | 0 | 383.15 | 162.15 | 292.70 | 1902.20 | -0.016 | 6.36E-01 | 7.37E-01 |
|  |  |  | 1 | 374.59 | 89.89 | 252.30 | 1014.60 |  |  |  |
| 2839-2 | TNFSF4 | P23510 | 0 | 416.35 | 920.79 | 219.40 | 9427.00 | -0.030 | 6.37E-01 | 7.37E-01 |
|  |  |  | 1 | 391.50 | 1190.09 | 198.30 | 20841.70 |  |  |  |
| 4220-39 | FER | P16591 | 0 | 3943.51 | 4730.77 | 222.90 | 21510.10 | -0.094 | 6.39E-01 | 7.39E-01 |
|  |  |  | 1 | 4132.92 | 4304.05 | 143.80 | 24475.00 |  |  |  |
| 2860-19 | KPNA2 | P52292 | 0 | 285.86 | 47.16 | 203.30 | 449.30 | -0.020 | 6.44E-01 | 7.44E-01 |
|  |  |  | 1 | 302.68 | 340.16 | 147.30 | 6015.50 |  |  |  |
| 2695-25 | PECAM1 | P16284 | 0 | 1158.67 | 244.34 | 926.80 | 2607.00 | 0.021 | 6.46E-01 | 7.46E-01 |
|  |  |  | 1 | 1458.72 | 5179.86 | 827.40 | 90778.90 |  |  |  |
| 3847-56 | ETHE1 | O95571 | 0 | 4782.50 | 1575.03 | 2472.70 | 14044.20 | -0.026 | 6.50E-01 | 7.50E-01 |
|  |  |  | 1 | 5051.34 | 4675.05 | 1981.60 | 65579.90 |  |  |  |
| 3534-14 | CD40LG | P29965 | 0 | 646.17 | 1793.20 | 216.10 | 18246.80 | 0.039 | 6.57E-01 | 7.57E-01 |
|  |  |  | 1 | 548.44 | 662.53 | 198.20 | 7606.90 |  |  |  |
| 5036-50 | TNFAIP6 | P98066 | 0 | 1394.27 | 432.09 | 735.30 | 3159.50 | -0.024 | 6.57E-01 | 7.57E-01 |
|  |  |  | 1 | 1398.79 | 587.99 | 354.40 | 7263.60 |  |  |  |
| 11101-18 | TLR4 | O00206 | 0 | 514.90 | 602.26 | 252.00 | 5674.30 | -0.041 | 6.57E-01 | 7.57E-01 |
|  |  |  | 1 | 796.06 | 4866.17 | 199.00 | 83239.00 |  |  |  |
| 3728-52 | SCT | P09683 | 0 | 259.87 | 79.19 | 179.50 | 815.10 | -0.021 | 6.63E-01 | 7.62E-01 |
|  |  |  | 1 | 268.42 | 182.43 | 167.60 | 2660.30 |  |  |  |
| 2809-25 | ADAMTS4 | O75173 | 0 | 220.50 | 319.62 | 139.10 | 3366.70 | -0.020 | 6.68E-01 | 7.66E-01 |
|  |  |  | 1 | 201.43 | 156.10 | 117.80 | 2565.70 |  |  |  |
| 10990-21 | LRRK2 | Q5S007 | 0 | 2475.30 | 338.15 | 1882.10 | 3582.60 | -0.020 | 6.67E-01 | 7.66E-01 |
|  |  |  | 1 | 2704.05 | 3788.28 | 1020.70 | 65584.50 |  |  |  |
| 5315-22 | TNNT2 | P45379 | 0 | 4855.30 | 3592.00 | 2358.80 | 22038.50 | 0.029 | 6.69E-01 | 7.67E-01 |
|  |  |  | 1 | 4750.72 | 2573.03 | 2186.00 | 24727.60 |  |  |  |
| 5939-42 | TNFSF12 | O43508 | 0 | 790.77 | 329.97 | 386.40 | 3387.00 | 0.026 | 6.70E-01 | 7.67E-01 |
|  |  |  | 1 | 857.39 | 737.05 | 243.00 | 10315.20 |  |  |  |
| 2855-49 | MAPK3 | P27361 | 0 | 43656.66 | 28014.19 | 11437.00 | 113075.50 | -0.055 | 6.74E-01 | 7.71E-01 |
|  |  |  | 1 | 46288.35 | 31884.93 | 6875.90 | 136206.10 |  |  |  |
| 4453-83 | LRRTM3 | Q86VH5 | 0 | 259.26 | 224.21 | 160.20 | 2331.60 | 0.023 | 6.74E-01 | 7.71E-01 |
|  |  |  | 1 | 258.90 | 152.60 | 148.10 | 1790.40 |  |  |  |
| 3305-6 | DLL4 | Q9NR61 | 0 | 487.07 | 163.61 | 279.70 | 1777.40 | -0.022 | 6.75E-01 | 7.72E-01 |
|  |  |  | 1 | 515.18 | 648.93 | 246.30 | 11277.90 |  |  |  |
| 5193-51 | KRAS | P01116 | 0 | 1493.84 | 642.18 | 979.00 | 6620.10 | 0.019 | 6.77E-01 | 7.72E-01 |
|  |  |  | 1 | 1498.31 | 436.23 | 484.10 | 3872.50 |  |  |  |
| 5019-16 | UCHL1 | P09936 | 0 | 2078.07 | 1019.76 | 952.90 | 4806.00 | -0.034 | 6.77E-01 | 7.72E-01 |
|  |  |  | 1 | 2152.89 | 1919.94 | 800.10 | 27677.50 |  |  |  |
| 5013-2 | CLIC1 | O00299 | 0 | 9085.31 | 7506.72 | 1448.90 | 32254.40 | -0.071 | 6.77E-01 | 7.72E-01 |
|  |  |  | 1 | 10565.12 | 10223.50 | 813.00 | 49439.70 |  |  |  |
| 5021-13 | PPA1 | Q15181 | 0 | 34585.23 | 17072.63 | 12976.50 | 154814.00 | -0.039 | 6.79E-01 | 7.73E-01 |
|  |  |  | 1 | 35931.05 | 19124.90 | 1940.10 | 184969.30 |  |  |  |
| 5076-53 | EPHA10 | Q5JZY3 | 0 | 476.40 | 452.09 | 318.60 | 4754.30 | -0.022 | 6.82E-01 | 7.76E-01 |
|  |  |  | 1 | 457.60 | 270.33 | 219.20 | 3051.80 |  |  |  |
| 5015-15 | PLA2G7 | Q13093 | 0 | 938.88 | 214.95 | 524.90 | 1778.30 | 0.019 | 6.87E-01 | 7.81E-01 |
|  |  |  | 1 | 988.41 | 666.11 | 461.80 | 11322.80 |  |  |  |
| 2730-58 | MICA | Q29983 | 0 | 508.72 | 350.13 | 80.50 | 2618.60 | -0.045 | 6.87E-01 | 7.81E-01 |
|  |  |  | 1 | 516.79 | 723.14 | 69.60 | 12102.20 |  |  |  |
| 5085-18 | IL20RA | Q9UHF4 | 0 | 310.21 | 116.40 | 198.30 | 1202.40 | 0.022 | 6.88E-01 | 7.81E-01 |
|  |  |  | 1 | 353.22 | 555.98 | 201.50 | 8941.70 |  |  |  |
| 6649-51 | NTN1 | O95631 | 0 | 656.29 | 314.49 | 341.60 | 2984.40 | 0.027 | 6.93E-01 | 7.85E-01 |
|  |  |  | 1 | 761.12 | 1322.91 | 282.00 | 21715.30 |  |  |  |
| 3432-21 | EPHA3 | P29320 | 0 | 211.45 | 310.79 | 120.70 | 3134.80 | -0.029 | 6.94E-01 | 7.86E-01 |
|  |  |  | 1 | 214.86 | 435.34 | 96.00 | 7140.90 |  |  |  |
| 4435-66 | ENPP7 | Q6UWV6 | 0 | 8362.88 | 5703.14 | 1798.50 | 36217.10 | 0.042 | 6.95E-01 | 7.87E-01 |
|  |  |  | 1 | 8805.57 | 6416.82 | 1049.60 | 47282.00 |  |  |  |
| 5355-69 | TNFSF14 | O43557 | 0 | 802.19 | 668.24 | 453.50 | 4218.80 | 0.028 | 6.97E-01 | 7.88E-01 |
|  |  |  | 1 | 956.42 | 3676.74 | 396.60 | 64002.70 |  |  |  |
| 4987-17 | FCAR | P24071 | 0 | 284.45 | 181.46 | 182.20 | 1548.40 | 0.021 | 6.98E-01 | 7.89E-01 |
|  |  |  | 1 | 288.86 | 268.12 | 175.20 | 4532.50 |  |  |  |
| 3499-77 | IL17B | Q9UHF5 | 0 | 169.83 | 21.68 | 129.90 | 241.80 | 0.015 | 7.00E-01 | 7.89E-01 |
|  |  |  | 1 | 178.11 | 76.70 | 110.80 | 863.50 |  |  |  |
| 2278-61 | TIMP2 | P16035 | 0 | 454.42 | 66.96 | 309.70 | 678.60 | 0.011 | 7.01E-01 | 7.89E-01 |
|  |  |  | 1 | 461.92 | 98.75 | 303.90 | 1051.80 |  |  |  |
| 4967-1 | C1QBP | Q07021 | 0 | 4862.40 | 621.44 | 3892.80 | 8826.00 | -0.010 | 7.01E-01 | 7.89E-01 |
|  |  |  | 1 | 4882.79 | 1205.41 | 3570.50 | 15746.20 |  |  |  |
| 2609-59 | CST3 | P01034 | 0 | 2816.97 | 594.27 | 1470.50 | 5295.60 | -0.014 | 7.00E-01 | 7.89E-01 |
|  |  |  | 1 | 2804.60 | 661.17 | 1422.10 | 5735.50 |  |  |  |
| 2599-51 | TNFSF9 | P41273 | 0 | 501.78 | 786.53 | 280.40 | 7909.60 | -0.025 | 6.99E-01 | 7.89E-01 |
|  |  |  | 1 | 587.21 | 2866.08 | 225.50 | 49970.60 |  |  |  |
| 2665-26 | TNFRSF17 | Q02223 | 0 | 9356.83 | 2335.61 | 4707.90 | 15170.50 | 0.020 | 7.03E-01 | 7.90E-01 |
|  |  |  | 1 | 9709.66 | 3466.29 | 2398.10 | 37144.40 |  |  |  |
| 2634-2 | IL2RG | P31785 | 0 | 4492.30 | 3015.59 | 959.00 | 11965.30 | 0.046 | 7.08E-01 | 7.95E-01 |
|  |  |  | 1 | 4999.92 | 3941.35 | 930.90 | 36305.90 |  |  |  |
| 2381-52 | C5 | P01031 | 0 | 7977.79 | 1182.26 | 5897.40 | 15632.60 | 0.008 | 7.10E-01 | 7.97E-01 |
|  |  |  | 1 | 8017.78 | 1027.28 | 3900.40 | 11186.20 |  |  |  |
| 4159-130 | CFH | P08603 | 0 | 67434.32 | 8145.19 | 41852.70 | 86404.00 | -0.007 | 7.14E-01 | 8.01E-01 |
|  |  |  | 1 | 67013.74 | 7507.52 | 47113.10 | 113762.50 |  |  |  |
| 4986-59 | PTK2 | Q05397 | 0 | 386.26 | 198.00 | 247.80 | 1496.50 | 0.019 | 7.17E-01 | 8.03E-01 |
|  |  |  | 1 | 398.47 | 292.78 | 185.20 | 3539.50 |  |  |  |
| 3152-57 | TNFRSF1B | P20333 | 0 | 14149.03 | 3897.69 | 6924.10 | 28044.40 | -0.018 | 7.20E-01 | 8.06E-01 |
|  |  |  | 1 | 14491.20 | 10607.72 | 6309.10 | 182980.80 |  |  |  |
| 14132-21 | HHLA2 | Q9UM44 | 0 | 1680.48 | 2308.27 | 751.40 | 21065.50 | -0.038 | 7.21E-01 | 8.06E-01 |
|  |  |  | 1 | 2084.09 | 5030.10 | 591.80 | 53813.80 |  |  |  |
| 3495-15 | CXCL6 | P80162 | 0 | 2310.23 | 3075.89 | 593.10 | 29549.00 | 0.038 | 7.24E-01 | 8.09E-01 |
|  |  |  | 1 | 2764.56 | 8781.79 | 459.50 | 147265.80 |  |  |  |
| 8485-7 | KEAP1 | Q14145 | 0 | 563.60 | 763.92 | 312.00 | 7408.80 | -0.020 | 7.30E-01 | 8.15E-01 |
|  |  |  | 1 | 511.66 | 361.31 | 280.80 | 4436.70 |  |  |  |
| 3194-36 | GP6 | Q9HCN6 | 0 | 13982.66 | 8846.58 | 1963.70 | 41642.50 | 0.042 | 7.33E-01 | 8.18E-01 |
|  |  |  | 1 | 16018.89 | 12198.61 | 2093.50 | 66030.00 |  |  |  |
| 4272-46 | GPI | P06744 | 0 | 27368.37 | 13892.36 | 10818.80 | 63642.90 | -0.031 | 7.34E-01 | 8.18E-01 |
|  |  |  | 1 | 27685.84 | 14650.54 | 3299.20 | 83867.60 |  |  |  |
| 3820-68 | MAPKAPK2 | P49137 | 0 | 16308.98 | 11559.07 | 2291.20 | 42630.30 | -0.055 | 7.34E-01 | 8.18E-01 |
|  |  |  | 1 | 18617.95 | 14601.10 | 1532.30 | 62556.10 |  |  |  |
| 2913-1 | CCL23 | P55773 | 0 | 3463.63 | 909.45 | 1703.30 | 5979.60 | -0.016 | 7.36E-01 | 8.18E-01 |
|  |  |  | 1 | 3458.39 | 1074.71 | 1417.90 | 8713.10 |  |  |  |
| 4862-63 | BMPR2 | Q13873 | 0 | 593.23 | 268.04 | 393.80 | 2322.00 | 0.022 | 7.37E-01 | 8.19E-01 |
|  |  |  | 1 | 697.95 | 1147.64 | 362.90 | 17770.30 |  |  |  |
| 2968-61 | TNFSF15 | O95150 | 0 | 991.89 | 414.42 | 320.80 | 3373.20 | 0.025 | 7.40E-01 | 8.22E-01 |
|  |  |  | 1 | 1234.65 | 4008.60 | 328.20 | 70056.50 |  |  |  |
| 4903-72 | PPP3CA PPP3R1 | Q08209 P63098 | 0 | 2729.02 | 1886.08 | 628.30 | 9434.50 | 0.046 | 7.42E-01 | 8.23E-01 |
|  |  |  | 1 | 3317.84 | 2949.56 | 492.80 | 18793.10 |  |  |  |
| 3311-27 | FCGR3B | O75015 | 0 | 1702.90 | 424.45 | 941.20 | 3131.00 | 0.015 | 7.42E-01 | 8.23E-01 |
|  |  |  | 1 | 1741.85 | 510.13 | 739.80 | 4005.40 |  |  |  |
| 5360-9 | AKT2 | P31751 | 0 | 18937.10 | 13529.87 | 3010.40 | 54990.40 | -0.044 | 7.47E-01 | 8.28E-01 |
|  |  |  | 1 | 19763.69 | 13935.39 | 2453.70 | 55988.60 |  |  |  |
| 5005-4 | MAPK12 | P53778 | 0 | 1100.31 | 429.06 | 487.00 | 2896.50 | 0.026 | 7.49E-01 | 8.28E-01 |
|  |  |  | 1 | 1465.28 | 4714.22 | 467.20 | 79696.70 |  |  |  |
| 5018-68 | PRDX6 | P30041 | 0 | 787.34 | 507.77 | 204.00 | 2203.30 | -0.040 | 7.48E-01 | 8.28E-01 |
|  |  |  | 1 | 1070.95 | 4753.90 | 129.60 | 82539.50 |  |  |  |
| 3415-61 | IBSP | P21815 | 0 | 4595.09 | 1847.16 | 1533.30 | 12528.00 | 0.024 | 7.50E-01 | 8.29E-01 |
|  |  |  | 1 | 4926.74 | 3866.04 | 1308.90 | 46662.10 |  |  |  |
| 5226-36 | CSNK2A2 CSNK2B | P19784 P67870 | 0 | 700.93 | 463.28 | 210.10 | 2183.80 | 0.036 | 7.52E-01 | 8.31E-01 |
|  |  |  | 1 | 812.66 | 1064.59 | 169.60 | 13650.40 |  |  |  |
| 3357-67 | CDK2 CCNA2 | P24941 P20248 | 0 | 353.26 | 111.44 | 263.60 | 1124.30 | 0.016 | 7.53E-01 | 8.31E-01 |
|  |  |  | 1 | 409.33 | 900.31 | 160.90 | 15694.40 |  |  |  |
| 13095-51 | REG1A | P05451 | 0 | 552.60 | 580.39 | 195.30 | 5986.00 | 0.021 | 7.59E-01 | 8.36E-01 |
|  |  |  | 1 | 536.84 | 288.09 | 198.50 | 2443.40 |  |  |  |
| 2649-77 | ICAM3 | P32942 | 0 | 1073.98 | 3164.90 | 594.10 | 32319.60 | 0.019 | 7.61E-01 | 8.38E-01 |
|  |  |  | 1 | 944.51 | 1970.58 | 533.20 | 25957.50 |  |  |  |
| 3881-49 | DYNLL1 | P63167 | 0 | 726.49 | 2570.71 | 263.30 | 25777.60 | 0.030 | 7.62E-01 | 8.39E-01 |
|  |  |  | 1 | 600.21 | 1286.11 | 257.50 | 17973.90 |  |  |  |
| 3480-7 | DUSP3 | P51452 | 0 | 2593.84 | 2008.84 | 387.20 | 8313.90 | -0.044 | 7.63E-01 | 8.39E-01 |
|  |  |  | 1 | 2835.81 | 2408.12 | 385.10 | 11422.40 |  |  |  |
| 4549-78 | FUT5 | Q11128 | 0 | 9366.18 | 3661.13 | 3149.00 | 28051.70 | -0.024 | 7.64E-01 | 8.39E-01 |
|  |  |  | 1 | 9702.11 | 5005.31 | 954.10 | 33876.80 |  |  |  |
| 4786-58 | CMPK1 | P30085 | 0 | 6416.71 | 1373.79 | 4633.20 | 15417.20 | 0.011 | 7.69E-01 | 8.44E-01 |
|  |  |  | 1 | 6555.44 | 1824.66 | 2496.40 | 16834.10 |  |  |  |
| 13131-5 | HK1 | P19367 | 0 | 1623.84 | 816.23 | 1017.70 | 6904.90 | -0.014 | 7.71E-01 | 8.44E-01 |
|  |  |  | 1 | 1598.46 | 732.78 | 585.30 | 9294.10 |  |  |  |
| 11094-104 | CLC | Q05315 | 0 | 677.71 | 209.61 | 476.00 | 1438.40 | -0.016 | 7.72E-01 | 8.44E-01 |
|  |  |  | 1 | 711.09 | 559.61 | 412.90 | 7297.80 |  |  |  |
| 5133-17 | TGFBR2 | P37173 | 0 | 1712.63 | 2175.45 | 729.80 | 14054.80 | -0.030 | 7.72E-01 | 8.44E-01 |
|  |  |  | 1 | 2507.85 | 14471.21 | 382.20 | 246777.20 |  |  |  |
| 5202-4 | PPID | Q08752 | 0 | 32601.31 | 28512.20 | 1470.10 | 108554.30 | -0.064 | 7.71E-01 | 8.44E-01 |
|  |  |  | 1 | 42864.24 | 40590.30 | 362.80 | 165122.00 |  |  |  |
| 5033-27 | TPM1 | P09493 | 0 | 1200.11 | 997.52 | 818.90 | 10412.00 | 0.018 | 7.73E-01 | 8.45E-01 |
|  |  |  | 1 | 1293.26 | 1991.48 | 634.70 | 32397.60 |  |  |  |
| 3072-4 | IL13 | P35225 | 0 | 1020.69 | 913.99 | 636.10 | 8077.20 | 0.017 | 7.75E-01 | 8.46E-01 |
|  |  |  | 1 | 1017.24 | 837.43 | 604.80 | 8462.70 |  |  |  |
| 4261-55 | PON1 | P27169 | 0 | 252.43 | 211.95 | 151.70 | 1676.60 | 0.014 | 7.77E-01 | 8.47E-01 |
|  |  |  | 1 | 239.19 | 122.59 | 160.10 | 1646.20 |  |  |  |
| 3208-2 | MATN3 | O15232 | 0 | 574.53 | 91.86 | 452.60 | 1060.70 | -0.009 | 7.76E-01 | 8.47E-01 |
|  |  |  | 1 | 578.48 | 147.00 | 245.50 | 1831.70 |  |  |  |
| 3313-21 | FCN2 | Q15485 | 0 | 1272.20 | 316.31 | 664.50 | 2244.10 | -0.012 | 7.82E-01 | 8.51E-01 |
|  |  |  | 1 | 1263.74 | 333.35 | 647.40 | 2732.30 |  |  |  |
| 2915-6 | PCNA | P12004 | 0 | 310.88 | 698.50 | 165.50 | 6812.10 | -0.018 | 7.81E-01 | 8.51E-01 |
|  |  |  | 1 | 245.33 | 211.44 | 155.60 | 3083.20 |  |  |  |
| 4464-10 | SIGLEC1 | Q9BZZ2 | 0 | 205.27 | 425.00 | 99.60 | 3311.80 | -0.024 | 7.87E-01 | 8.56E-01 |
|  |  |  | 1 | 478.92 | 5701.90 | 66.20 | 98893.80 |  |  |  |
| 2865-77 | KAT6A | Q92794 | 0 | 1554.53 | 347.16 | 1147.60 | 3984.80 | -0.010 | 7.89E-01 | 8.57E-01 |
|  |  |  | 1 | 1574.78 | 708.10 | 598.80 | 12226.30 |  |  |  |
| 5491-12 | SPOCK2 | Q92563 | 0 | 943.10 | 248.86 | 537.80 | 1850.10 | 0.011 | 7.90E-01 | 8.57E-01 |
|  |  |  | 1 | 953.29 | 267.47 | 429.40 | 3028.30 |  |  |  |
| 2953-31 | CGA LHB | P01215 P01229 | 0 | 1806.17 | 1672.99 | 302.00 | 10354.90 | -0.030 | 7.90E-01 | 8.57E-01 |
|  |  |  | 1 | 1698.45 | 2529.93 | 190.40 | 41897.80 |  |  |  |
| 11105-171 | ENO1 | P06733 | 0 | 129459.88 | 79659.77 | 38167.00 | 308422.10 | 0.032 | 7.93E-01 | 8.60E-01 |
|  |  |  | 1 | 143818.27 | 96121.25 | 15669.00 | 456929.00 |  |  |  |
| 3392-68 | AKT1 AKT2 AKT3 | P31749 P31751 Q9Y243 | 0 | 7790.62 | 5511.40 | 1677.10 | 25019.20 | 0.034 | 7.98E-01 | 8.64E-01 |
|  |  |  | 1 | 8950.18 | 7172.50 | 1385.80 | 34679.70 |  |  |  |
| 3681-87 | KLK14 | Q9P0G3 | 0 | 9300.16 | 1278.33 | 6345.80 | 13445.20 | -0.009 | 7.99E-01 | 8.64E-01 |
|  |  |  | 1 | 9385.99 | 2207.01 | 3143.30 | 25355.10 |  |  |  |
| 2979-8 | CXCL5 | P42830 | 0 | 666.90 | 461.41 | 446.90 | 3808.30 | 0.016 | 8.02E-01 | 8.67E-01 |
|  |  |  | 1 | 883.17 | 4231.90 | 385.00 | 73765.40 |  |  |  |
| 3168-8 | ADAMTS5 | Q9UNA0 | 0 | 492.60 | 137.70 | 279.70 | 1403.40 | 0.016 | 8.03E-01 | 8.67E-01 |
|  |  |  | 1 | 734.79 | 3806.09 | 236.50 | 66000.60 |  |  |  |
| 5128-53 | SLAMF6 | Q96DU3 | 0 | 540.78 | 336.03 | 288.60 | 2801.20 | -0.016 | 8.03E-01 | 8.67E-01 |
|  |  |  | 1 | 555.12 | 528.58 | 147.40 | 7523.70 |  |  |  |
| 5340-24 | CASP10 | Q92851 | 0 | 4773.65 | 4368.30 | 3213.10 | 45464.70 | 0.012 | 8.07E-01 | 8.71E-01 |
|  |  |  | 1 | 4656.06 | 3222.01 | 2726.10 | 53756.50 |  |  |  |
| 4998-50 | JAK2 | O60674 | 0 | 126439.91 | 25993.02 | 50990.30 | 187408.60 | 0.009 | 8.09E-01 | 8.72E-01 |
|  |  |  | 1 | 127457.44 | 27431.21 | 39202.20 | 222350.10 |  |  |  |
| 4188-1 | AKR7A2 | O43488 | 0 | 7066.17 | 5708.42 | 669.90 | 23705.90 | 0.043 | 8.10E-01 | 8.72E-01 |
|  |  |  | 1 | 9186.68 | 9079.56 | 429.00 | 68136.40 |  |  |  |
| 13107-9 | LYPD3 | O95274 | 0 | 1183.61 | 367.03 | 714.90 | 3647.10 | -0.011 | 8.11E-01 | 8.72E-01 |
|  |  |  | 1 | 1189.38 | 412.91 | 356.70 | 4287.80 |  |  |  |
| 2618-10 | ERBB4 | Q15303 | 0 | 198.54 | 99.43 | 125.30 | 935.50 | -0.017 | 8.14E-01 | 8.75E-01 |
|  |  |  | 1 | 479.75 | 4629.27 | 120.30 | 80024.00 |  |  |  |
| 4234-8 | IL1RL1 | Q01638 | 0 | 5132.57 | 2503.47 | 1436.70 | 15479.50 | 0.017 | 8.19E-01 | 8.76E-01 |
|  |  |  | 1 | 5254.94 | 3069.66 | 1461.90 | 24709.60 |  |  |  |
| 2753-2 | C1QA C1QB C1QC | P02745 P02746 P02747 | 0 | 44136.42 | 7680.46 | 20259.10 | 77806.20 | -0.009 | 8.18E-01 | 8.76E-01 |
|  |  |  | 1 | 44289.00 | 8886.22 | 2647.00 | 101824.60 |  |  |  |
| 3628-3 | MAP2K2 | P36507 | 0 | 1652.47 | 426.02 | 598.60 | 2920.70 | -0.011 | 8.18E-01 | 8.76E-01 |
|  |  |  | 1 | 1653.99 | 478.52 | 571.20 | 3171.60 |  |  |  |
| 5352-11 | TNFRSF14 | Q92956 | 0 | 692.11 | 343.75 | 445.80 | 3822.80 | -0.013 | 8.16E-01 | 8.76E-01 |
|  |  |  | 1 | 734.27 | 866.69 | 346.60 | 14270.90 |  |  |  |
| 4832-75 | TNFRSF10A | O00220 | 0 | 2072.45 | 2270.90 | 1045.70 | 16206.50 | -0.016 | 8.18E-01 | 8.76E-01 |
|  |  |  | 1 | 1823.17 | 1193.55 | 996.30 | 16232.00 |  |  |  |
| 8484-24 | LEP | P41159 | 0 | 12606.67 | 10467.89 | 2010.60 | 59539.20 | 0.029 | 8.19E-01 | 8.77E-01 |
|  |  |  | 1 | 12975.57 | 11749.96 | 1269.80 | 102406.80 |  |  |  |
| 4993-16 | GSTA3 | Q16772 | 0 | 316.82 | 353.72 | 148.30 | 3391.60 | 0.023 | 8.21E-01 | 8.78E-01 |
|  |  |  | 1 | 520.02 | 2289.01 | 121.10 | 29735.10 |  |  |  |
| 2952-75 | IGF1 | P05019 | 0 | 1022.66 | 247.09 | 580.60 | 1750.10 | -0.012 | 8.22E-01 | 8.78E-01 |
|  |  |  | 1 | 1163.29 | 2601.26 | 476.20 | 45770.90 |  |  |  |
| 3204-2 | LTA4H | P09960 | 0 | 1443.05 | 561.52 | 607.00 | 4181.40 | -0.022 | 8.23E-01 | 8.78E-01 |
|  |  |  | 1 | 1878.89 | 2974.62 | 456.90 | 32139.80 |  |  |  |
| 7624-19 | ANK2 | Q01484 | 0 | 843.92 | 1514.48 | 417.60 | 10575.40 | 0.020 | 8.26E-01 | 8.79E-01 |
|  |  |  | 1 | 904.77 | 2474.72 | 293.50 | 37827.90 |  |  |  |
| 4482-66 | C5 C6 | P01031 P13671 | 0 | 576.99 | 79.82 | 436.70 | 1024.00 | -0.005 | 8.25E-01 | 8.79E-01 |
|  |  |  | 1 | 575.63 | 80.07 | 288.80 | 983.20 |  |  |  |
| 13111-79 | BCL6 | P41182 | 0 | 1691.61 | 359.73 | 1110.50 | 3003.20 | -0.014 | 8.26E-01 | 8.79E-01 |
|  |  |  | 1 | 2275.08 | 9217.84 | 703.30 | 160475.10 |  |  |  |
| 4920-10 | LYZ | P61626 | 0 | 106767.52 | 19820.54 | 66593.70 | 153244.20 | 0.008 | 8.28E-01 | 8.80E-01 |
|  |  |  | 1 | 107851.93 | 22216.11 | 38327.70 | 205005.20 |  |  |  |
| 3143-3 | CD4 | P01730 | 0 | 416.17 | 191.60 | 295.70 | 1586.00 | -0.008 | 8.28E-01 | 8.80E-01 |
|  |  |  | 1 | 404.57 | 112.27 | 257.90 | 1038.40 |  |  |  |
| 11071-1 | IL5 | P05113 | 0 | 302.30 | 136.79 | 181.60 | 990.70 | -0.015 | 8.32E-01 | 8.83E-01 |
|  |  |  | 1 | 342.73 | 559.32 | 149.30 | 8873.00 |  |  |  |
| 2668-70 | CAPN1 CAPNS1 | P07384 P04632 | 0 | 66033.60 | 34112.66 | 19521.10 | 146560.40 | 0.022 | 8.33E-01 | 8.83E-01 |
|  |  |  | 1 | 71312.12 | 41592.95 | 10533.90 | 213264.60 |  |  |  |
| 5846-24 | NOG | Q13253 | 0 | 3171.26 | 850.81 | 2033.40 | 7132.40 | 0.011 | 8.33E-01 | 8.83E-01 |
|  |  |  | 1 | 3275.26 | 1190.11 | 640.70 | 9861.40 |  |  |  |
| 2737-22 | NOV | P48745 | 0 | 763.09 | 217.50 | 571.50 | 2031.10 | 0.008 | 8.36E-01 | 8.85E-01 |
|  |  |  | 1 | 790.81 | 557.92 | 379.50 | 9588.20 |  |  |  |
| 13093-6 | SECTM1 | Q8WVN6 | 0 | 1409.11 | 280.90 | 908.00 | 2591.90 | -0.008 | 8.37E-01 | 8.85E-01 |
|  |  |  | 1 | 1450.35 | 977.20 | 953.70 | 17116.40 |  |  |  |
| 14156-33 | YWHAB | P31946 | 0 | 31425.33 | 24575.86 | 4546.20 | 103346.50 | 0.030 | 8.39E-01 | 8.87E-01 |
|  |  |  | 1 | 36025.82 | 30041.99 | 3568.30 | 137207.20 |  |  |  |
| 3443-61 | HIPK3 | Q9H422 | 0 | 766.76 | 879.77 | 338.70 | 5433.30 | 0.021 | 8.47E-01 | 8.95E-01 |
|  |  |  | 1 | 1002.83 | 2882.29 | 312.60 | 40232.20 |  |  |  |
| 3391-10 | PIK3CG | P48736 | 0 | 2125.74 | 432.12 | 1351.30 | 3693.50 | -0.009 | 8.49E-01 | 8.96E-01 |
|  |  |  | 1 | 2182.38 | 1069.62 | 738.30 | 17908.50 |  |  |  |
| 2853-68 | CHEK1 | O14757 | 0 | 624.39 | 123.17 | 427.80 | 1193.90 | 0.007 | 8.50E-01 | 8.96E-01 |
|  |  |  | 1 | 634.87 | 189.86 | 374.90 | 2395.70 |  |  |  |
| 10365-132 | IL12B IL23A | P29460 Q9NPF7 | 0 | 1698.65 | 810.32 | 557.10 | 5407.80 | -0.016 | 8.56E-01 | 9.02E-01 |
|  |  |  | 1 | 1865.12 | 2551.23 | 344.10 | 42726.00 |  |  |  |
| 4889-82 | WNT7A | O00755 | 0 | 317.42 | 168.09 | 201.40 | 1449.50 | 0.013 | 8.57E-01 | 9.03E-01 |
|  |  |  | 1 | 743.98 | 7535.42 | 167.10 | 130803.60 |  |  |  |
| 3339-33 | THBS2 | P35442 | 0 | 20440.64 | 9495.79 | 6584.50 | 69223.70 | 0.015 | 8.59E-01 | 9.04E-01 |
|  |  |  | 1 | 22103.53 | 15258.05 | 1882.30 | 133550.80 |  |  |  |
| 3437-80 | FLT3 | P36888 | 0 | 6800.96 | 4481.93 | 1930.90 | 20812.90 | 0.021 | 8.62E-01 | 9.06E-01 |
|  |  |  | 1 | 7474.58 | 5491.72 | 1500.10 | 35879.80 |  |  |  |
| 5138-50 | TNFRSF12A | Q9NP84 | 0 | 201.72 | 142.34 | 114.50 | 1198.10 | 0.012 | 8.62E-01 | 9.06E-01 |
|  |  |  | 1 | 211.98 | 222.79 | 99.60 | 2764.10 |  |  |  |
| 5063-12 | CD244 | Q9BZW8 | 0 | 220.93 | 188.80 | 143.10 | 2015.30 | 0.011 | 8.69E-01 | 9.11E-01 |
|  |  |  | 1 | 272.82 | 883.51 | 118.30 | 14879.20 |  |  |  |
| 2638-12 | CSF1R | P07333 | 0 | 209.38 | 54.04 | 135.50 | 564.50 | -0.007 | 8.68E-01 | 9.11E-01 |
|  |  |  | 1 | 210.11 | 57.44 | 114.00 | 563.90 |  |  |  |
| 6151-18 | MAP2K3 | P46734 | 0 | 3157.74 | 1470.03 | 1497.40 | 7479.40 | 0.015 | 8.70E-01 | 9.12E-01 |
|  |  |  | 1 | 3422.06 | 2070.80 | 1075.50 | 11423.60 |  |  |  |
| 2654-19 | TNFRSF1A | P19438 | 0 | 1579.45 | 393.21 | 757.90 | 3076.50 | 0.009 | 8.72E-01 | 9.14E-01 |
|  |  |  | 1 | 1847.40 | 4495.46 | 845.50 | 78906.30 |  |  |  |
| 3343-1 | ACY1 | Q03154 | 0 | 6801.06 | 4185.87 | 1471.40 | 28957.10 | -0.014 | 8.74E-01 | 9.15E-01 |
|  |  |  | 1 | 6911.52 | 4532.20 | 1640.80 | 34661.60 |  |  |  |
| 2597-8 | VEGFA | P15692 | 0 | 13050.04 | 1938.90 | 8908.60 | 21195.10 | -0.005 | 8.76E-01 | 9.15E-01 |
|  |  |  | 1 | 13204.24 | 3324.71 | 5151.00 | 45787.80 |  |  |  |
| 4925-54 | MMP13 | P45452 | 0 | 781.20 | 370.72 | 481.90 | 2767.30 | -0.011 | 8.76E-01 | 9.15E-01 |
|  |  |  | 1 | 883.30 | 1588.55 | 383.60 | 25868.10 |  |  |  |
| 5028-59 | CD163 | Q86VB7 | 0 | 3957.53 | 1291.13 | 1637.40 | 12114.20 | -0.007 | 8.87E-01 | 9.24E-01 |
|  |  |  | 1 | 3968.20 | 1238.09 | 1011.20 | 8277.60 |  |  |  |
| 10372-18 | STAT6 | P42226 | 0 | 651.78 | 382.48 | 303.40 | 2648.90 | -0.011 | 8.86E-01 | 9.24E-01 |
|  |  |  | 1 | 668.20 | 531.29 | 250.70 | 7204.30 |  |  |  |
| 4480-59 | C3 | P01024 | 0 | 17016.64 | 9760.24 | 2604.90 | 53682.10 | -0.020 | 8.87E-01 | 9.24E-01 |
|  |  |  | 1 | 19054.46 | 13156.42 | 64.50 | 99317.30 |  |  |  |
| 4407-10 | MST1 | P26927 | 0 | 7226.99 | 3256.52 | 607.90 | 13901.30 | -0.016 | 8.88E-01 | 9.25E-01 |
|  |  |  | 1 | 7709.73 | 9834.91 | 87.70 | 167894.60 |  |  |  |
| 3504-58 | HAMP | P81172 | 0 | 15164.74 | 9829.09 | 82.40 | 43053.50 | -0.026 | 8.94E-01 | 9.30E-01 |
|  |  |  | 1 | 16946.12 | 13230.70 | 124.10 | 85358.80 |  |  |  |
| 4146-58 | NTF4 | P34130 | 0 | 250.17 | 90.26 | 159.30 | 701.10 | 0.007 | 8.97E-01 | 9.32E-01 |
|  |  |  | 1 | 266.98 | 259.56 | 135.80 | 4172.60 |  |  |  |
| 10336-3 | STUB1 | Q9UNE7 | 0 | 1828.87 | 1269.74 | 561.70 | 6610.80 | -0.015 | 8.96E-01 | 9.32E-01 |
|  |  |  | 1 | 1940.18 | 1788.20 | 460.10 | 19706.30 |  |  |  |
| 4179-57 | YWHAB YWHAE YWHAG YWHAH YWHAQ YWHAZ SFN | P31946 P62258 P61981 Q04917 P27348 P63104 P31947 | 0 | 7800.70 | 6944.44 | 1209.00 | 28335.20 | 0.020 | 8.99E-01 | 9.32E-01 |
|  |  |  | 1 | 8870.01 | 8230.28 | 1006.00 | 32686.60 |  |  |  |
| 2785-15 | CCL8 | P80075 | 0 | 251.72 | 39.29 | 184.30 | 419.20 | 0.004 | 8.99E-01 | 9.32E-01 |
|  |  |  | 1 | 256.93 | 76.32 | 149.80 | 863.40 |  |  |  |
| 2939-10 | ARTN | Q5T4W7 | 0 | 602.65 | 235.15 | 381.60 | 2182.40 | -0.006 | 9.04E-01 | 9.37E-01 |
|  |  |  | 1 | 628.71 | 495.08 | 253.00 | 5617.50 |  |  |  |
| 4240-31 | PKM2 | P14618 | 0 | 72430.46 | 55628.04 | 7827.10 | 239025.60 | -0.021 | 9.04E-01 | 9.37E-01 |
|  |  |  | 1 | 88695.59 | 75839.37 | 3515.00 | 318974.30 |  |  |  |
| 4306-4 | TKT | P29401 | 0 | 56292.40 | 28448.54 | 17073.70 | 138942.30 | 0.012 | 9.06E-01 | 9.38E-01 |
|  |  |  | 1 | 61433.00 | 36161.96 | 11325.60 | 175774.30 |  |  |  |
| 3445-53 | IL15RA | Q13261 | 0 | 1153.68 | 616.85 | 818.20 | 6608.10 | 0.005 | 9.09E-01 | 9.38E-01 |
|  |  |  | 1 | 1154.94 | 507.39 | 635.70 | 5971.00 |  |  |  |
| 3336-50 | TFPI | P10646 | 0 | 73029.52 | 14049.54 | 34067.80 | 116449.80 | 0.004 | 9.11E-01 | 9.38E-01 |
|  |  |  | 1 | 73468.34 | 15417.58 | 20918.90 | 141717.70 |  |  |  |
| 2761-49 | FGF18 | O76093 | 0 | 401.24 | 174.98 | 271.00 | 1558.20 | -0.007 | 9.10E-01 | 9.38E-01 |
|  |  |  | 1 | 486.25 | 1494.69 | 231.90 | 25812.60 |  |  |  |
| 14157-21 | YWHAE | P62258 | 0 | 1105.42 | 1327.01 | 446.00 | 13995.60 | -0.007 | 9.10E-01 | 9.38E-01 |
|  |  |  | 1 | 1037.49 | 431.97 | 288.90 | 3957.30 |  |  |  |
| 5271-5 | RAC3 | P60763 | 0 | 10590.86 | 4774.10 | 2976.30 | 22636.10 | -0.011 | 9.09E-01 | 9.38E-01 |
|  |  |  | 1 | 11451.24 | 9548.47 | 2903.80 | 130479.40 |  |  |  |
| 2768-56 | HPX | P02790 | 0 | 1179.03 | 604.16 | 222.80 | 3774.90 | -0.011 | 9.08E-01 | 9.38E-01 |
|  |  |  | 1 | 1170.54 | 612.66 | 138.30 | 3704.00 |  |  |  |
| 3347-9 | ADRBK1 | P25098 | 0 | 11805.63 | 9075.18 | 1406.80 | 33753.30 | -0.018 | 9.11E-01 | 9.38E-01 |
|  |  |  | 1 | 13515.64 | 11097.76 | 951.20 | 50118.00 |  |  |  |
| 5196-7 | NMT1 | P30419 | 0 | 4600.76 | 2703.97 | 1206.30 | 11140.10 | 0.013 | 9.12E-01 | 9.38E-01 |
|  |  |  | 1 | 5232.87 | 4428.95 | 958.70 | 44386.40 |  |  |  |
| 3868-8 | SGTA | O43765 | 0 | 5745.95 | 4143.94 | 1022.80 | 19183.20 | 0.015 | 9.18E-01 | 9.44E-01 |
|  |  |  | 1 | 6810.81 | 5617.53 | 319.00 | 34161.90 |  |  |  |
| 3828-54 | PDGFC | Q9NRA1 | 0 | 1372.17 | 8089.84 | 293.30 | 81319.50 | -0.010 | 9.19E-01 | 9.44E-01 |
|  |  |  | 1 | 657.07 | 978.04 | 223.00 | 10421.70 |  |  |  |
| 8458-111 | SNCA | P37840 | 0 | 14680.24 | 6233.29 | 4364.70 | 29302.80 | -0.009 | 9.22E-01 | 9.45E-01 |
|  |  |  | 1 | 15498.01 | 7869.05 | 2103.70 | 45693.00 |  |  |  |
| 5810-25 | TDGF1 | P13385 | 0 | 1511.45 | 1401.20 | 306.80 | 5599.40 | -0.016 | 9.22E-01 | 9.45E-01 |
|  |  |  | 1 | 1597.19 | 1905.06 | 129.40 | 20475.30 |  |  |  |
| 5870-23 | BAD | Q92934 | 0 | 13257.64 | 9033.95 | 2688.70 | 34485.40 | 0.012 | 9.24E-01 | 9.46E-01 |
|  |  |  | 1 | 14259.39 | 10185.64 | 2129.40 | 56415.10 |  |  |  |
| 3045-72 | PTN | P21246 | 0 | 1810.15 | 595.03 | 1045.90 | 4463.40 | 0.005 | 9.24E-01 | 9.46E-01 |
|  |  |  | 1 | 1844.91 | 784.27 | 678.60 | 9365.80 |  |  |  |
| 5486-73 | ICAM2 | P13598 | 0 | 1957.56 | 338.03 | 1412.70 | 3320.50 | -0.003 | 9.27E-01 | 9.47E-01 |
|  |  |  | 1 | 1992.31 | 718.18 | 1162.70 | 10946.10 |  |  |  |
| 3888-8 | ING1 | Q9UK53 | 0 | 28894.00 | 23032.02 | 4714.00 | 99734.90 | -0.013 | 9.27E-01 | 9.47E-01 |
|  |  |  | 1 | 30857.82 | 24142.28 | 2243.00 | 119402.80 |  |  |  |
| 3844-2 | PPIA | P62937 | 0 | 151082.77 | 55788.08 | 42677.30 | 298179.90 | -0.007 | 9.32E-01 | 9.51E-01 |
|  |  |  | 1 | 157100.07 | 68557.36 | 42470.80 | 372271.80 |  |  |  |
| 5864-10 | ALDOA | P04075 | 0 | 96421.31 | 34706.52 | 41243.20 | 222785.90 | 0.005 | 9.37E-01 | 9.54E-01 |
|  |  |  | 1 | 99890.06 | 42236.54 | 30925.20 | 269699.40 |  |  |  |
| 3053-49 | FLT3LG | P49771 | 0 | 565.24 | 263.71 | 327.50 | 2133.60 | 0.005 | 9.36E-01 | 9.54E-01 |
|  |  |  | 1 | 658.54 | 1503.59 | 291.20 | 25714.70 |  |  |  |
| 5410-53 | CDH15 | P55291 | 0 | 4690.48 | 926.59 | 3460.20 | 10556.10 | 0.003 | 9.36E-01 | 9.54E-01 |
|  |  |  | 1 | 4751.18 | 1154.37 | 1777.40 | 9960.30 |  |  |  |
| 5238-26 | PPIE | Q9UNP9 | 0 | 2013.46 | 1364.68 | 657.20 | 6781.10 | 0.009 | 9.43E-01 | 9.59E-01 |
|  |  |  | 1 | 2289.74 | 1955.44 | 342.70 | 18836.80 |  |  |  |
| 4250-23 | NSFL1C | Q9UNZ2 | 0 | 557.04 | 470.62 | 98.00 | 3844.60 | -0.009 | 9.44E-01 | 9.60E-01 |
|  |  |  | 1 | 628.90 | 911.52 | 65.10 | 14112.50 |  |  |  |
| 3898-5 | PSMD7 | P51665 | 0 | 330.87 | 89.28 | 211.10 | 723.60 | -0.003 | 9.46E-01 | 9.62E-01 |
|  |  |  | 1 | 341.50 | 184.51 | 181.50 | 2273.40 |  |  |  |
| 4970-55 | CA2 | P00918 | 0 | 456.35 | 1389.04 | 184.80 | 13955.20 | 0.005 | 9.57E-01 | 9.71E-01 |
|  |  |  | 1 | 415.39 | 1596.56 | 151.80 | 27605.60 |  |  |  |
| 3192-3 | CNDP2 | Q96KP4 | 0 | 1575.30 | 523.40 | 991.60 | 3530.40 | -0.003 | 9.57E-01 | 9.71E-01 |
|  |  |  | 1 | 1581.38 | 538.12 | 799.80 | 3758.00 |  |  |  |
| 4973-18 | BIRC3 | Q13489 | 0 | 306.42 | 154.04 | 203.00 | 1371.90 | -0.003 | 9.62E-01 | 9.76E-01 |
|  |  |  | 1 | 323.85 | 356.96 | 197.00 | 4657.60 |  |  |  |
| 5134-52 | HAVCR2 | Q8TDQ0 | 0 | 12007.79 | 3246.70 | 5668.70 | 22789.80 | -0.002 | 9.63E-01 | 9.76E-01 |
|  |  |  | 1 | 12190.32 | 4148.74 | 4064.00 | 32479.00 |  |  |  |
| 5350-14 | GPC6 | Q9Y625 | 0 | 2103.65 | 489.72 | 1445.20 | 5430.10 | -0.002 | 9.66E-01 | 9.78E-01 |
|  |  |  | 1 | 2120.25 | 650.17 | 1425.70 | 8397.30 |  |  |  |
| 4963-19 | ARPP19 | P56211 | 0 | 3730.02 | 2592.38 | 900.70 | 10799.30 | -0.006 | 9.68E-01 | 9.79E-01 |
|  |  |  | 1 | 4067.39 | 3121.59 | 600.30 | 16235.40 |  |  |  |
| 5061-27 | ICOSLG | O75144 | 0 | 2283.08 | 1617.40 | 899.00 | 10163.80 | 0.004 | 9.70E-01 | 9.81E-01 |
|  |  |  | 1 | 3036.95 | 6559.96 | 698.20 | 82287.10 |  |  |  |
| 11067-13 | BGLAP | P02818 | 0 | 1808.18 | 1106.75 | 793.70 | 9381.70 | -0.003 | 9.71E-01 | 9.81E-01 |
|  |  |  | 1 | 2169.85 | 3918.46 | 734.40 | 57507.60 |  |  |  |
| 4965-27 | ATP5B | P06576 | 0 | 2578.38 | 2805.92 | 1615.70 | 29796.00 | -0.002 | 9.74E-01 | 9.83E-01 |
|  |  |  | 1 | 2521.02 | 1852.97 | 777.40 | 27677.90 |  |  |  |
| 14120-2 | RNF43 | Q68DV7 | 0 | 1324.70 | 581.83 | 848.10 | 6288.80 | 0.002 | 9.76E-01 | 9.84E-01 |
|  |  |  | 1 | 1432.18 | 1287.56 | 502.50 | 18439.80 |  |  |  |
| 13094-75 | RSPO3 | Q9BXY4 | 0 | 1758.49 | 576.01 | 1123.60 | 6624.70 | -0.001 | 9.77E-01 | 9.85E-01 |
|  |  |  | 1 | 1795.06 | 785.42 | 634.50 | 12502.90 |  |  |  |
| 3809-1 | FGFR3 | P22607 | 0 | 1087.05 | 614.31 | 716.20 | 6128.00 | -0.001 | 9.84E-01 | 9.91E-01 |
|  |  |  | 1 | 1283.87 | 4129.20 | 606.90 | 72242.10 |  |  |  |
| 14150-7 | IL36A | Q9UHA7 | 0 | 2475.62 | 2402.48 | 377.10 | 15861.00 | 0.002 | 9.85E-01 | 9.91E-01 |
|  |  |  | 1 | 2317.25 | 1863.52 | 355.80 | 18797.00 |  |  |  |
| 4436-1 | ENTPD3 | O75355 | 0 | 1334.04 | 281.90 | 956.20 | 2942.60 | 0.001 | 9.86E-01 | 9.91E-01 |
|  |  |  | 1 | 1350.63 | 444.04 | 838.90 | 6929.70 |  |  |  |
| 14127-240 | IFNB1 | P01574 | 0 | 719.44 | 817.93 | 348.10 | 7386.10 | -0.001 | 9.87E-01 | 9.91E-01 |
|  |  |  | 1 | 792.64 | 1350.22 | 285.80 | 18217.30 |  |  |  |
| 14149-9 | IL36B | Q9NZH7 | 0 | 669.31 | 428.08 | 444.40 | 3528.00 | -0.001 | 9.90E-01 | 9.94E-01 |
|  |  |  | 1 | 862.46 | 3203.60 | 331.10 | 54645.00 |  |  |  |
| 4155-3 | TNC | P24821 | 0 | 23544.97 | 7012.09 | 12145.60 | 45927.60 | -0.001 | 9.93E-01 | 9.96E-01 |
|  |  |  | 1 | 24239.57 | 9647.00 | 5929.80 | 70256.20 |  |  |  |
| 2732-58 | NANOG | Q9H9S0 | 0 | 217.60 | 203.56 | 134.90 | 1967.00 | 0.001 | 9.95E-01 | 9.97E-01 |
|  |  |  | 1 | 348.65 | 2307.82 | 120.30 | 39919.40 |  |  |  |
| 3835-11 | TLR2 | O60603 | 0 | 599.33 | 116.78 | 352.20 | 1140.10 | 0.000 | 9.97E-01 | 9.98E-01 |
|  |  |  | 1 | 624.43 | 289.82 | 271.20 | 3275.70 |  |  |  |
| 4930-21 | STC1 | P52823 | 0 | 2667.63 | 735.47 | 1407.70 | 5728.00 | 0.000 | 9.98E-01 | 9.98E-01 |
|  |  |  | 1 | 2733.55 | 1016.27 | 705.40 | 8492.60 |  |  |  |
| 6152-111 | TP53 | P04637 | 0 | 1128.30 | 391.35 | 865.60 | 4389.20 | 0.000 | 9.98E-01 | 9.98E-01 |
|  |  |  | 1 | 1128.51 | 336.99 | 729.00 | 3940.00 |  |  |  |

p Values are shown as exponentiated values.

**Table S2.** Operating characteristics of all models in the test set for the IPF versus control multivariable modelling.

| Model | Accuracy | Kappa | Sensitivity | Specificity | | Positive predictive value | Negative predictive value |
| --- | --- | --- | --- | --- | --- | --- | --- |
| PLS | 1 | 1 | 1 | | 1 | 1 | 1 |
| GLMN | 0.99 | 0.97 | 0.96 | | 1 | 1 | 0.99 |
| FDA | 0.99 | 0.97 | 1 | | 0.99 | 0.96 | 1 |
| SVM | 0.99 | 0.97 | 0.96 | | 1 | 1 | 0.99 |
| KNN | 0.91 | 0.73 | 0.64 | | 1 | 1 | 0.89 |
| RPART | 0.89 | 0.69 | 0.68 | | 0.96 | 0.85 | 0.9 |
| RF | 0.96 | 0.89 | 0.84 | | 1 | 1 | 0.95 |
| GBM | 1 | 1 | 1 | | 1 | 1 | 1 |

PLS, partial least squares; GLMN, penalised logistic regression; FDA, flexible discriminant analysis; SVM, support vector machines; KNN, K-nearest neighbours; RPART, recursive partitioning - single tree; RF, random forest; GBM, gradient boosted machine.

**Table S3.** Proteins designated as among the most influential in at least two of the eight multivariable models.

| Gene | Aptamer ID | Protein | Frequency |
| --- | --- | --- | --- |
| MMP3 | 2788-55 | Stromelysin-1 | 8 |
| SFN | 4829-43 | 14-3-3 protein sigma | 8 |
| ICAM5 | 5124-69 | Intercellular adhesion molecule 5 | 8 |
| OLR1 | 3636-37 | Oxidized low-density lipoprotein receptor 1 | 7 |
| APOA1 | 2750-3 | Apolipoprotein A-I | 6 |
| C1R | 3285-23 | Complement C1r subcomponent | 6 |
| ADSL | 5023-23 | Adenylosuccinate lyase | 6 |
| SHH | 2743-5 | Sonic hedgehog protein | 5 |
| GPD1 | 11081-1 | Glycerol-3-phosphate dehydrogenase [NAD(+)], cytoplasmic | 5 |
| GDF15 | 4374-45 | Growth/differentiation factor 15 | 4 |
| CAPG | 4968-50 | Macrophage-capping protein | 4 |
| VWF | 3050-7 | d | 3 |
| CCL18 | 3044-3 | C-C motif chemokine 18 | 2 |

**Table S4.** Proteins significantly associated with FVC % predicted (unadjusted and adjusted for anti-fibrotic treatment).

| APTAMER | ENTREZGENESYMBOL | Difference in disease severity per unit change in log2RFU | Uncorrected  p Value (treatment unadjusted) | FDR corrected  p Value (treatment unadjusted) | Difference in disease severity per unit change in log2RFU | Uncorrected  p Value (treatment adjusted) | FDR corrected  p Value (treatment adjusted) |
| --- | --- | --- | --- | --- | --- | --- | --- |
| 5124-69 | ICAM5 | -6.49 | 7.74E-05 | 1.96E-02 | -6.49 | 9.11E-05 | 1.71E-02 |
| 3708-62 | A2M | -9.32 | 1.11E-04 | 1.96E-02 | -9.42 | 1.03E-04 | 1.71E-02 |
| 2578-67 | CCL2 | -5.82 | 1.23E-04 | 1.96E-02 | -5.83 | 1.34E-04 | 1.94E-02 |
| 5852-6 | S100A12 | -5.15 | 2.26E-04 | 2.46E-02 | -5.17 | 2.19E-04 | 2.60E-02 |
| 4567-82 | SH2D1A | -5.63 | 3.16E-04 | 2.82E-02 | -5.62 | 3.56E-04 | 3.09E-02 |
| 5116-62 | ROBO2 | -11.47 | 2.84E-04 | 2.82E-02 | -11.44 | 3.20E-04 | 2.98E-02 |
| 3216-2 | PIGR | -5.13 | 5.60E-04 | 3.66E-02 | -5.11 | 6.59E-04 | 4.09E-02 |
| 3470-1 | SELE | -5.78 | 5.89E-04 | 3.66E-02 | -5.76 | 6.96E-04 | 4.09E-02 |
| 4297-62 | SPON1 | -7.69 | 7.65E-04 | 3.99E-02 | -7.67 | 8.33E-04 | 4.19E-02 |
| 3317-33 | HTRA2 | 6.34 | 7.55E-06 | 6.55E-03 | 6.32 | 8.99E-06 | 7.25E-03 |
| 9171-11 | CSRP3 | 6.37 | 6.99E-05 | 1.96E-02 | 6.36 | 7.70E-05 | 1.71E-02 |
| 3642-4 | CD84 | 6.00 | 3.84E-04 | 2.82E-02 | 5.98 | 4.12E-04 | 3.10E-02 |
| 2686-67 | IGFBP6 | 9.89 | 1.02E-03 | 4.17E-02 | 9.86 | 1.09E-03 | 4.50E-02 |
| 2962-50 | PTHLH | 7.26 | 9.73E-04 | 4.17E-02 | 7.27 | 9.96E-04 | 4.50E-02 |
| 5482-61 | RBP4 | 9.45 | 1.71E-03 | 5.07E-02 | 9.85 | 1.25E-03 | 4.55E-02 |

p Values are shown as exponentiated values.

**Table S5.** Proteins significantly associated with DLco % predicted (unadjusted and adjusted for anti-fibrotic treatment).

| APTAMER | ENTREZGENESYMBOL | Difference in disease severity per unit change in log2RFU | Uncorrected  p Value (treatment unadjusted) | FDR corrected  p Value (treatment unadjusted) | Difference in disease severity per unit change in log2RFU | Uncorrected  p Value (treatment adjusted) | FDR corrected   p Value (treatment adjusted) |
| --- | --- | --- | --- | --- | --- | --- | --- |
| 3487-32 | CXCL13 | -5.26 | 8.33E-11 | 1.09E-07 | -5.27 | 1.05E-10 | 1.37E-07 |
| 3216-2 | PIGR | -7.62 | 5.05E-10 | 3.29E-07 | -7.67 | 5.80E-10 | 3.78E-07 |
| 4297-62 | SPON1 | -11.50 | 1.04E-09 | 4.54E-07 | -11.49 | 1.30E-09 | 5.67E-07 |
| 5124-69 | ICAM5 | -7.98 | 4.82E-09 | 1.57E-06 | -8.01 | 5.77E-09 | 1.88E-06 |
| 4968-50 | CAPG | -6.39 | 3.73E-08 | 9.72E-06 | -6.39 | 4.13E-08 | 1.08E-05 |
| 4413-3 | SLPI | -10.02 | 5.11E-07 | 1.11E-04 | -10.10 | 5.92E-07 | 1.29E-04 |
| 8464-31 | RSPO4 | -6.74 | 1.23E-06 | 2.29E-04 | -6.75 | 1.32E-06 | 2.47E-04 |
| 4234-8 | IL1RL1 | -5.32 | 2.04E-05 | 3.32E-03 | -5.34 | 2.06E-05 | 3.36E-03 |
| 3213-65 | NID1 | -8.90 | 1.29E-04 | 1.40E-02 | -8.99 | 1.37E-04 | 1.49E-02 |
| 3447-64 | CXCL8 | -5.70 | 1.96E-04 | 1.71E-02 | -5.68 | 2.21E-04 | 1.81E-02 |
| 4374-45 | GDF15 | -5.23 | 3.01E-04 | 2.31E-02 | -5.21 | 3.39E-04 | 2.60E-02 |
| 5116-62 | ROBO2 | -9.23 | 5.41E-04 | 3.43E-02 | -9.26 | 5.54E-04 | 3.20E-02 |
| 4721-54 | TFF3 | -5.50 | 6.31E-04 | 3.43E-02 | -5.50 | 7.24E-04 | 3.78E-02 |
| 2999-6 | LSAMP | -8.49 | 6.12E-04 | 3.43E-02 | -8.50 | 6.89E-04 | 3.74E-02 |
| 4930-21 | STC1 | -5.48 | 8.58E-04 | 4.02E-02 | -5.48 | 9.91E-04 | 4.37E-02 |
| 10342-55 | PIAS4 | -6.28 | 9.89E-04 | 4.30E-02 | -6.35 | 9.23E-04 | 4.30E-02 |
| 2888-49 | C7 | -7.22 | 1.14E-03 | 4.60E-02 | -7.30 | 1.07E-03 | 4.50E-02 |
| 2637-77 | MRC1 | -6.62 | 1.14E-03 | 4.60E-02 | -6.59 | 1.30E-03 | 4.99E-02 |
| 2973-15 | CD36 | 6.24 | 1.59E-04 | 1.59E-02 | 6.23 | 1.71E-04 | 1.59E-02 |
| 3616-3 | GNS | 5.79 | 1.79E-04 | 1.67E-02 | 5.85 | 1.66E-04 | 1.59E-02 |
| 3340-53 | THBS4 | 5.49 | 4.55E-04 | 3.30E-02 | 5.49 | 5.07E-04 | 3.20E-02 |
| 3538-26 | DDC | 5.81 | 6.61E-04 | 3.45E-02 | 6.00 | 5.06E-04 | 3.20E-02 |
| 3581-53 | AHSG | 12.04 | 8.93E-04 | 4.02E-02 | 12.17 | 8.62E-04 | 4.17E-02 |
| 5103-30 | CD200R1 | 6.00 | 1.38E-03 | 5.30E-02 | 6.09 | 1.29E-03 | 4.99E-02 |

p Values are shown as exponentiated values.

**Table S6.** Proteins significantly associated with composite physiologic index (unadjusted and adjusted for anti-fibrotic treatment).

| APTAMER | ENTREZGENESYMBOL | Difference in disease severity per unit change in log2RFU | Uncorrected  p Value (treatment unadjusted) | FDR corrected  p Value (treatment unadjusted) | Difference in disease severity per unit change in log2RFU | Uncorrected  p Value (treatment adjusted) | FDR corrected  p Value (treatment adjusted) |
| --- | --- | --- | --- | --- | --- | --- | --- |
| 4297-62 | SPON1 | 9.57 | 9.81E-11 | 4.27E-08 | 9.59 | 1.13E-10 | 4.93E-08 |
| 3216-2 | PIGR | 6.28 | 6.93E-11 | 4.27E-08 | 6.32 | 8.03E-11 | 4.93E-08 |
| 5124-69 | ICAM5 | 6.85 | 1.53E-10 | 5E-08 | 6.88 | 1.78E-10 | 5.82E-08 |
| 4968-50 | CAPG | 5.38 | 3.71E-09 | 9.67E-07 | 5.38 | 3.98E-09 | 1.04E-06 |
| 4413-3 | SLPI | 8.14 | 2.17E-07 | 4.72E-05 | 8.24 | 2.22E-07 | 4.82E-05 |
| 5116-62 | ROBO2 | 9.42 | 6.44E-06 | 1.05E-03 | 9.41 | 7.48E-06 | 1.22E-03 |
| 3708-62 | A2M | 6.67 | 3.06E-05 | 3.32E-03 | 6.73 | 2.9E-05 | 3.16E-03 |
| 2381-52 | C5 | 11.98 | 2.48E-04 | 1.62E-02 | 12.18 | 2.71E-04 | 1.69E-02 |
| 2999-6 | LSAMP | 7.07 | 2.88E-04 | 1.71E-02 | 7.09 | 3.29E-04 | 1.95E-02 |
| 3060-43 | C9 | 8.52 | 4.02E-04 | 2.10E-02 | 8.50 | 4.60E-04 | 2.40E-02 |
| 3581-53 | AHSG | -10.16 | 3.72E-04 | 2.10E-02 | -10.16 | 4.11E-04 | 2.24E-02 |
| 2637-77 | MRC1 | 5.35 | 8.60E-04 | 3.52E-02 | 5.35 | 9.31E-04 | 3.57E-02 |
| 3213-65 | NID1 | 6.04 | 1.02E-03 | 3.79E-02 | 6.20 | 8.86E-04 | 3.57E-02 |
| 5412-53 | CD27 | -7.59 | 1.12E-03 | 4.00E-02 | -7.59 | 1.16E-03 | 4.06E-02 |
| 2888-49 | C7 | 5.50 | 1.69E-03 | 5.00E-02 | 5.58 | 1.54E-03 | 4.07E-02 |

p Values are shown as exponentiated values.
